# A Single‐Stranded DNA‐Encoded Chemical Library Based on a Stereoisomeric Scaffold Enables Ligand Discovery by Modular Assembly of Building Blocks

**DOI:** 10.1002/advs.202001970

**Published:** 2020-10-14

**Authors:** Gabriele Bassi, Nicholas Favalli, Miriam Vuk, Marco Catalano, Adriano Martinelli, Anika Trenner, Antonio Porro, Su Yang, Chuin Lean Tham, Mustafa Moroglu, Wyatt W. Yue, Stuart J. Conway, Peter K. Vogt, Alessandro A. Sartori, Jörg Scheuermann, Dario Neri

**Affiliations:** ^1^ Department of Chemistry and Applied Biosciences ETH Zürich Zürich 8092 Switzerland; ^2^ Institute of Molecular Cancer Research University of Zürich Zürich 8006 Switzerland; ^3^ Scripps Research Institute Department of Molecular Medicine La Jolla CA 92037 USA; ^4^ Structural Genomic Consortium (SGC) Nuffield Department of Medicine University of Oxford Oxford OX1 2JD UK; ^5^ Department of Chemistry Chemistry Research Laboratory University of Oxford Mansfield Road Oxford OX1 3TA UK

**Keywords:** Human alpha‐aminoadipic semialdehyde synthase (AASS), affinity maturation, cyclic‐AMP response element binding protein (CREBBP), DNA‐encoded chemical libraries (DEL), drug discovery, encoded self‐assembling chemical libraries (ESAC), FANCD2‐associated nuclease 1 (FAN‐1), phosphatidylinositol 3‐kinase (PI3K)

## Abstract

A versatile and Lipinski‐compliant DNA‐encoded library (DEL), comprising 366 600 glutamic acid derivatives coupled to oligonucleotides serving as amplifiable identification barcodes is designed, constructed, and characterized. The GB‐DEL library, constructed in single‐stranded DNA format, allows de novo identification of specific binders against several pharmaceutically relevant proteins. Moreover, hybridization of the single‐stranded DEL with a set of known protein ligands of low to medium affinity coupled to a complementary DNA strand results in self‐assembled selectable chemical structures, leading to the identification of affinity‐matured compounds.

## Graphical abstract

A single‐stranded DNA‐encoded chemical library (DEL) of 366 600 compounds, based on a stereoisomeric scaffold and on a single‐stranded DNA format, allows the isolation of ligands against multiple protein targets. The library can also be hybridized to a complementary DNA strand equipped with a protein binder, in order to generate affinity‐matured ligands.

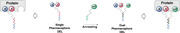

## 1 Introduction

The identification of small organic ligands to protein targets is crucial for many areas of chemical, biological, and pharmaceutical research. High‐quality organic ligands are fundamental for the discovery of novel drugs,^[^ ^1^ ^]^ of affinity‐chromatography supports^[^ ^2^ ^]^ and of tool compounds for the interrogation of biological processes.^[^ ^3^ ^]^

Protein ligands can often be discovered using conventional high‐throughput screening (HTS) of compound collections, but these procedures can be expensive,^[^ ^4^ ^]^ require complex logistics and are typically limited to the analysis of a few hundred thousand compounds.^[^ ^5^ ^]^ The encoding of chemical compounds with DNA fragments serving as amplifiable identification barcodes, first postulated by Brenner and Lerner,^[^ ^6^ ^]^ is growing in popularity as a ligand discovery methodology, both in industry and in academia. DNA‐encoded chemical libraries (DELs) do not require dedicated logistics for the handling of library members, since the DNA tag allows the facile identification of binding molecules by PCR amplification and DNA sequencing.^[^ ^7^ ^]^

In most cases, DELs are interrogated by affinity‐capture on target proteins of interest, immobilized on a solid support,^[^ ^8^ ^]^ but alternative methodologies (e.g., those based on photocrosslinking, on capillary electrophoresis or on interaction‐dependent PCR procedures) have been proposed.^[^ ^9^ ^]^

In principle, highly complex DELs can be constructed by the iterative assembly of multiple sets of building blocks.^[^ ^8^, ^10^ ^]^ However, the incorporation of three or more chemical moieties may result in molecular structures that violate Lipinski's rules of five and which may be too large for pharmaceutical applications.^[^ ^11^ ^]^ In some cases, DEL‐derived binders comprising multiple building blocks may be reduced in size by retaining only those structural elements that contribute most of the binding energy.^[^ ^12^ ^]^ Alternatively, DELs can be constructed using two sets of building blocks, yielding “hits” which can be subsequently optimized by medicinal chemistry.^[^ ^8^, ^10^, ^13^ ^]^

Here, we describe the construction of a novel versatile DEL based on two sets of building blocks, coupled to a central stereo‐defined 4‐azido‐5‐methoxy‐5‐oxopentanoic acid. The GB‐DEL library, whose members were typically smaller than 500 Da, was screened against multiple targets, allowing the discovery of novel binders which were compatible with Lipinksi's rule of five. While most DELs described so far had featured DNA in double‐stranded format,^[^ ^9^, ^14^ ^]^ we used single‐stranded DNA barcodes in order to enable modular lead expansion procedures by DNA self‐assembly. The results of this article illustrate how novel protein ligands can efficiently be discovered in single‐stranded format and how ligands can be conveniently assembled with a single‐stranded DEL yielding dual‐display affinity maturation libraries. This approach may practically be used in iterative assembly and selection steps allowing ligand discovery and subsequent affinity maturation.

## 2 Results

### 2.1 Library Construction

**Figure 1:**
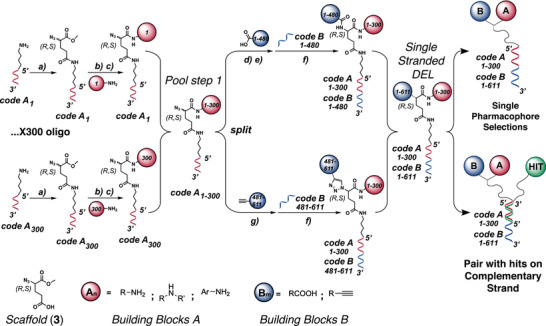
Schematic representation of the synthetical steps involved in GB‐DEL formation. The two sets of building blocks, A and B, and the corresponding encoding DNA portions are color‐coded in red and blue, respectively. The first set of building blocks A was conjugated to DNA via reverse amide bond. The building blocks B were linked to the library either by amide bond formation or CuAAC click reaction. a) Amide bond formation of three hundred different oligonucleotides (described as CodeA) with 3 (EDC/S‐NHS, DIPEA, r.t. 30’, in TEA buffer 50 × 10^−3^ m, pH = 10, 37 °C); b) deprotection of scaffold's methyl ester (LiOH 200 × 10^−3^ m); c) reverse amide bond formation (On‐DNA‐COOH, 500 × 10^−3^ m DMTMM in MOPS 100 × 10^−3^ m, 1 m NaCl, pH = 7) of each of the three hundred different scaffold‐oligo conjugates with an amine (building blocks A). Each of the CodeA‐building block A conjugates was individually purified by HPLC and pooled in equimolar amount to form pool step 1. The pool step 1 was split in 611 wells. d) Wells 1–480 underwent Staudinger reduction (30 × 10^−3^ m TCEP in 500 × 10^−3^ m TRIS, pH = 7.4), e) coupling to a carboxylic acid and f) encoding with CodeB 1–480 by splint ligation (Ligase buffer, NEB T‐4 DNA ligase (f)); g) CuAAC click reaction (250 × 10^−3^ m Borate buffer, pH = 9.5, 5 × 10^−3^ m CuSO_4_ mq H_2_O, 5 × 10^−3^ m sodium ascorbate in H_2_O) of N_3_‐pool step 1 (wells 481–611) with alkynes (10 × 10^−3^ m) and subsequent splint ligation (f)). After pooling and purification by RP‐HPLC, the single stranded DEL was used directly in single pharmacophore selections or paired to a complementary oligonucleotide displaying a known protein binding moiety “HIT” to perform 2+1 dual pharmacophore affinity maturation selections.

The first step for the synthesis of our library (termed GB‐DEL) was performed by coupling the 4‐azido‐5‐methoxy‐5‐oxopentanoic acid (3) onto 300 distinct amino‐tagged oligonucleotides (**Figure** **1**).

We used a 1:1 mixture of the *R* and *S* stereoisomers, with the aim to assess the contribution of chirality to protein recognition at the hit validation stage. The glutamic acid derivatives were deprotected and allowed to react with 299 amines (191 primary, 36 secondary and 72 (hetero) aromatic amines), plus one code reserved for ammonia, serving as a control (“building blocks A”). The resulting conjugates were individually HPLC‐purified, prior to pooling and splitting for the second coupling step with “building blocks B”. At this stage, we converted 480 aliquots of the mixture (each containing the 300 building block A conjugates) into the corresponding primary amines which were later reacted with 480 carboxylic acids. In parallel, 131 aliquots of *α*‐azido glutamic acid derivatives were reacted with terminal alkynes using copper(I)‐catalyzed alkyne azide cycloaddition (CuAAC), using a previously optimized procedure.^[^ ^15^ ^]^ The second reactions step was encoded by splint ligation^[^ ^13c^ ^]^ with 611 oligonucleotides, yielding a library comprising 2 (stereoisomers) x 300 (building block A derivatives) x 611 (building block B derivatives) = 366 600 compounds (Figure 1 and Figures S1–S10, Supporting Information). Compounds were eventually pooled and HPLC‐purified. GB‐DEL in single‐stranded DNA format could be used as such for protein selections (Streptavidin, CAIX, FAN1, AASS, PI3K), or hybridized to complementary oligonucleotide derivatives bearing a protein‐binding moiety for affinity maturation procedures (CREBBP, HSA, CAIX, tyrosinase, and trypsin) (Figures S11–S18, Supporting Information). Specific binders with dissociation constant in the single‐digit micromolar or in the submicromolar range were isolated, as illustrated in the following sections.

### 2.2 Selection Experiments against Protein Targets using the Library in Single‐Stranded DNA Format

#### 2.2.1 DEL Selections against Streptavidin and CAIX

**Figure 2:**
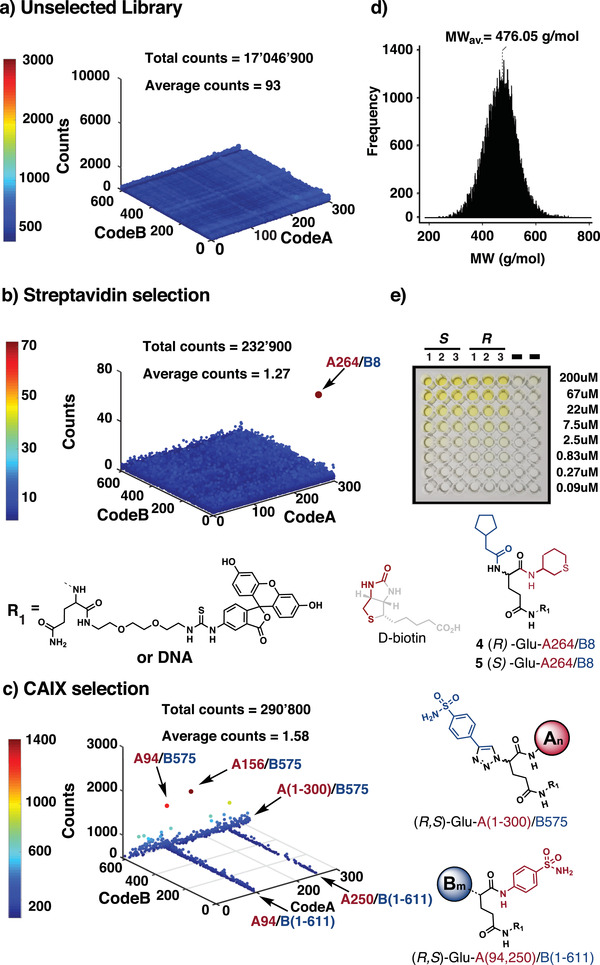
a) (left) High‐throughput DNA sequencing (HTDS) plot of unselected library GB‐DEL. The *x*,*y*,*z* axes of the tridimensional plot represent CodeA/CodeB and sequence counts of the GB‐DEL. Total sequence counts (TCs) = 17 046 900, average sequence counts (ACs) = 93. b) HTDS plot of streptavidin selections (TCs = 232’900, ACs = 1.27). The arrow indicates the most enriched CodeA/CodeB combination (A264/B8) which was re‐synthetized off‐DNA (right panel compounds 4, 5). c) HTDS plot of CAIX selections. TCs = 290 800, ACs = 1.58. (Right) Aromatic sulfonamide derivatives were included in the library as positive controls: A94, A250, and B575. Indicated by the arrows are the two most enriched building block combinations, A94/B575 and A156/B575, whose molecular structures and enrichment factors can be found in Table S5 (Supporting Information). d) Histogram of GB‐DEL final molecules’ molecular weights distribution. On the *x* axis molecular weights for the CodeA/CodeB combinations, on the *y* axis molecular weight frequency. The mean of the Gaussian distribution corresponds to MW 476.05 g mol^−1^. e) Enzyme‐linked immunosorbent assay (ELISA) of compound 4, 5 against streptavidin. The measurements have been performed in triplicate (*n* = 3). The Kd values ± standard error for compound 4 and 5 are 37 ± 2 and 42 ± 3 × 10^−6^ m, respectively.

Library aliquots containing 10^7^ copies of each compound were used for direct library selection. In total, more than 10 000 such aliquots were available. Affinity capture experiments with proteins immobilized on magnetic beads were typically performed in triplicate, using previously described procedures.^[^ ^7a^ ^]^ For clarity sake, only one fingerprint is shown in the main body of the article, but replicate selection results can be found in Figures S24–S30 (Supporting Information). We characterized the unselected library by high‐throughput DNA sequencing and observed a uniform distribution of sequence counts for the various library members, thus providing confidence about the proper execution of the various mixing steps during library synthesis (**Figure** **2**a). The majority of library compounds had molecular weights <500 Da (Figure 2d) and were compliant with Lipinski's rule of five (Tables S1 and S2, Supporting Information).^[^ ^11^, ^16^ ^]^

Since most selections were performed using biotinylated protein targets, we conducted selections on “empty” streptavidin‐coated magnetic beads. Only one slightly enriched compound was found which was structurally reminiscent of the high affinity streptavidin binder biotin (Figure 2e, compounds 4, 5). The compounds displayed a weak interaction with streptavidin, with dissociation constants Kd = 37 ± 2 and 42 ± 3 × 10^−6^ m, respectively, which were measured by a small molecule ELISA procedure, recently described by our group.^[^ ^17^ ^]^ Selections were also performed against human carbonic anhydrase IX, a tumor‐associated antigen and a marker of hypoxia.^[^ ^18^ ^]^ As expected, aromatic sulfonamides derivatives were found to be preferentially enriched (with enrichment factors (EF) up to 969‐fold), as revealed by the lines at coordinates A94, A250, and B575 of the fingerprints in 3D display mode (Figure 2c). For all subsequent selections, screening results were displayed in the more compact pseudo‐2D representation.^[^ ^19^ ^]^

#### 2.2.2 DEL Selections against FAN1, AASS, and PI3K

**Figure 3:**
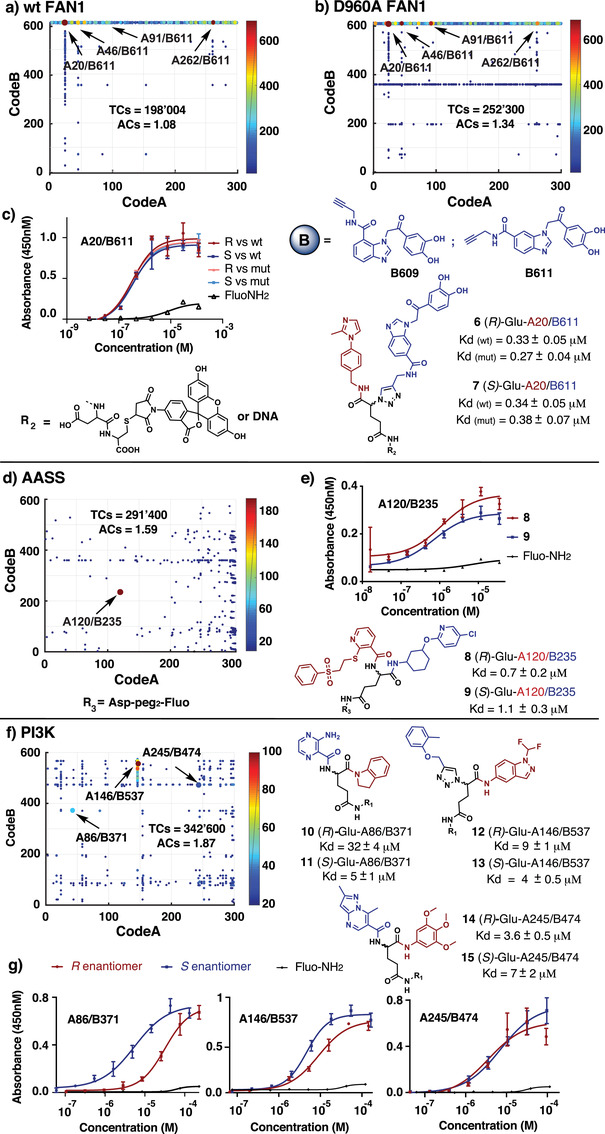
High‐throughput DNA sequencing plots of a) wt FAN1 (TCs = 198 004, ACs = 1.08); b) D960A FAN1 (TCs = 252 500, ACs = 1.34); d) AASS (TCs = 291 400, ACs = 1.59) and f) PI3K (TCs = 342 600, ACs = 1.87). The *x*,*y* plane of the bidimensional plots represent Code A and Code B, respectively. The jet scale and the dots color code indicate code sequence counts. The arrows indicate Code A/Code B combinations selected for re‐synthesis (compounds 6–15), the colors, red and blue, indicate building block A and B, respectively. c,e,g) ELISA binding assays of compounds against their protein targets. c) Compounds 6–7 against wt and D960A FAN1; e) compounds 8–9 against AASS; g) compounds 10–15 against PI3K; on the *x* axis concentration (m), on the *y* axis absorbance at 450 nm. Red and blue curves indicate *R* and *S* stereoisomer, respectively. Kd measurements have been performed in triplicate (*n* = 3). Error bars on the curve indicate standard deviation of the three measurements. The Kd values ± standard error for compound 6–15 are given next to the chemical structures. Structures of CodeA/CodeB combinations selectively enriched in the FAN1 selection (A46/B611, A91/B611, and A262/B611) can be found in Table S5 (Supporting Information).

We performed selections against proteins of pharmaceutical interest (**Figure** **3**). FANCD2‐associated nuclease 1 (FAN1) is DNA repair nuclease implicated in the resolution of inter‐strand crosslinks (ICLs) and in protective responses at stalled replication forks.^[^ ^20^ ^]^ Moreover, it has been reported that FAN1 is critical to maintain genome integrity in BRCA2‐deficient cells.^[^ ^21^ ^]^ Therefore, small‐molecule FAN1 inhibitors may also represent a promising strategy for treatment of BRCA‐mutated carriers. Based on the canSAR cancer drug discovery platform and using the chemical properties and bioactivity of small molecules annotated in ChEMBL database, it has been estimated that FAN1 is among 107 DNA damage response (DDR) factors predicted to be druggable.^[^ ^22^ ^]^

Selections were performed both on His_6_‐tagged wild‐type FAN1 and on a nuclease‐dead variant (D960A) carrying a mutation in the metal‐coordinating active site (Figure S31a,b, Supporting Information).^[^ ^23^ ^]^ A preferential enrichment of two lines, corresponding to building blocks B609 and B611 (two regioisomers of catechol derivatives) was observed (Figure 3a,b). The building block combination (A20/B611) inspired the synthesis of the two stereoisomers 6 and 7, which, however, bound to both versions of FAN1 with comparable affinities (Kd = 0.27 ± 0.04 × 10^−6^ m; Figure 3c), most likely to a remote epitope, as they do not inhibit FAN1 enzymatic activity. Indeed, partial inhibition of FAN1 5’Flap endonuclease activity by A20/B611 was seen only in the 100 × 10^−6^ m range (Figure S31,c, Supporting Information).

Human alpha‐aminoadipic semialdehyde synthase (AASS) catalyzes the first two steps of lysine catabolism. Inhibition of AASS is proposed as a substrate reduction therapy^[^ ^24^ ^]^ for an autosomal recessive neonatal seizure disorder, pyridoxine‐dependent epilepsy (PDE), caused by mutations in the ALDH7A1 gene encoding the enzyme downstream of AASS.^[^ ^25^ ^]^ Selection experiments against the saccharopine dehydrogenase domain of AASS revealed a preferential enrichment of the A120/B235 building block pairs (Figure 3d). Conversion of this chemical information into the stereoisomeric compounds 8 and 9 resulted in an apparent dissociation constant in the submicromolar concentration range (Kd = 0.7 ± 0.2 × 10^−6^ m for compound 8) (Figure 3e).

The p110*α*/p85*α* dimeric^[^ ^26^ ^]^ isoform of PI3K (phosphatidylinositol 3‐kinase) was the target of selections described in (Figure 3f). The regulatory subunit p85*α* combines with the catalytic subunits p110*α*/P85*α* to form the PI3K complex. Three structurally related pairs of building blocks (A86/B371, A146/B537, and A245/B474), with nitrogen‐containing heterocycles, were among the most enriched features in the fingerprints (Figure 3g). Binding assays performed with compounds 10–15 revealed a strong influence of the stereocenter on the binding affinity for the A86/B371 combination (≈10‐fold difference between R and S stereoisomers). A lower impact of the stereocenter was observed for A146/B537 and no difference in binding affinity was seen for the two A245/B474 enantiomers (Figure 3g).

### 2.3 Library Expansion by 2+1 Self‐Assembly with Complementary Oligonucleotide Derivatives

**Figure 4:**
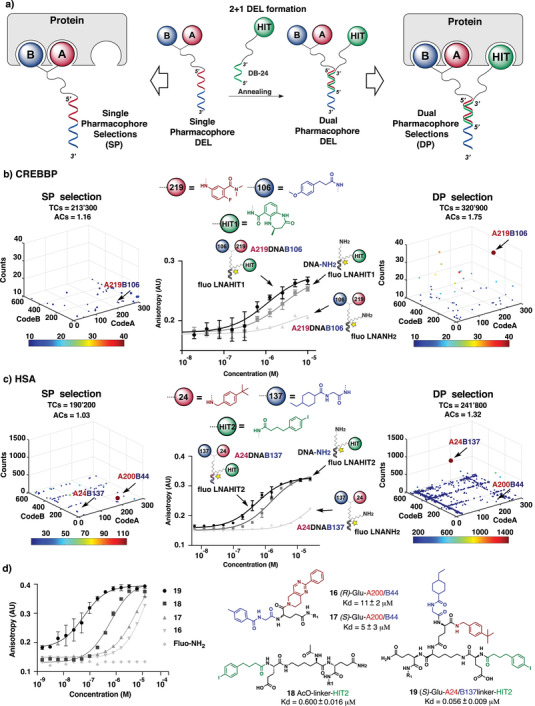
a) Schematic representation of 2+1 DEL formation (middle): The single pharmacophore DEL (GB‐DEL) is annealed to a partially complementary oligonucleotide (DB‐24) bearing a protein‐binding moiety (“HIT”). Left and right parts represent the selection process in single pharmacophore (SP) and in dual pharmacophore 2+1 (DP) mode, respectively. b) High‐throughput DNA sequencing plots (HTDS) of selections against the CREBBP bromodomain in SP format (TCs = 213 300, ACs = 1.16, left graph) and DP format bearing DB‐24‐HIT1 (TCs = 320 900, ACs = 1.75, right graph). The *x*,*y*,*z* axes of the tridimensional plot represent CodeA/CodeB and sequence counts of the GB‐DEL. The arrows indicate the A109/B206 combination (Enrichment factor (EF) SP selection = 3, EF DP selection = 22) selected for affinity maturation of HIT1. (Middle): On‐DNA fluorescence polarization (FP) of affinity maturated hit against CREBBP: A219, B106, HIT1 displayed on 12‐mer DNA/fluo‐LNA hetero‐duplex (black curve), only HIT1 displayed on 12‐mer DNA/fluo‐LNA hetero‐duplex (dark grey) and A219/B106 displayed on 12‐mer DNA/fluo‐LNA hetero‐duplex (light grey). c) HTDS plots of selections against human serum albumin in SP format (TCs = 190 200 ACs = 1.03, left graph) and DP format bearing DB‐24‐HIT2 (TCs = 241 800, ACs = 1.32, right graph). The *x*,*y*,*z* axes of the tridimensional plot represent CodeA/CodeB and sequence counts of the GB‐DEL. The arrows indicate the combination A24/B137 (Enrichment factor (EF) SP selection = 3, EF DP selection = 1119) selected for affinity maturation and A200/ B244 combination selected for de novo discovery EF SP selection = 112, respectively. EF has been calculated according to Equations S1–S3 in Tables S5 and S6 (Supporting Information). (Middle): On‐DNA fluorescence polarization (FP) with HSA: A24/B137/ HIT2 displayed on 12‐mer DNA/fluo‐LNA hetero‐duplex (black curve), only HIT2 displayed on 12‐mer DNA/fluo‐LNA hetero‐duplex (dark grey) and only A24/B137 (light grey). d) Off‐DNA fluorescence polarization of HSA hits. All the Kd measurements have been performed in triplicate (*n* = 3). Error bars on the curve indicate standard deviation of the three measurements. The Kd values ± standard error for compound 16–19 are given next to the chemical structures.

The single‐stranded DNA format of the GB‐DEL allowed the facile annealing with chemically modified complementary DNA strands, carrying a protein‐binding moiety (“hit”) at the 3’ end. This feature allowed us to compare selections performed with the unmodified GB‐DEL and with the self‐assembly products, featuring three sets of building blocks (**Figure** **4**a). We paired the GB‐DEL with a complementary DNA strand, carrying hits of intermediate affinity, in order to discover combinations of building blocks which could enable an affinity maturation procedure, using multiple proteins as targets.

CREBBP is a bromodomain‐containing transcriptional co‐regulator, which regulates a number ^[^ ^27^ ^]^of key intracellular processes.^[^ ^28^ ^]^ CREBBP selections featured a benzodiazepinone (HIT1) on the complementary DNA strand. This chemical moiety had previously been described as a 32 × 10^−6^ m binder.^[^ ^29^ ^]^ A preferential enrichment of the synergistic building blocks A219/B106 was observed in the selection with three sets of building blocks (“dual‐pharmacophore library”), while a substantially lower enrichment was seen using the unmodified GB‐DEL library (“single‐pharmacophore library”) (Figure 4b). Affinity measurements performed by fluorescence polarization with DNA‐LNA hybrids^[^ ^13^, ^30^ ^]^ revealed a binding increase for the triple combination of building blocks, while the A219/B106 moiety had an undetectable interaction with the protein at concentrations below 10 × 10^−6^ m. Similar findings have recently been reported for other dual‐pharmacophore systems.^[^ ^13^, ^30^, ^31^ ^]^

HSA, human serum albumin, is the most abundant protein in serum which has been used as target for pharmacokinetic modulation strategies.^[^ ^32^ ^]^ For HSA selections, the GB‐DEL was paired with a 4‐(*p*‐iodophenyl)butanoic acid moiety (HIT2) that we had previously described as a portable HSA binder.^[^ ^13d^ ^]^ A striking potentiation of the A24/B137 was observed, while the A200/B44 combination had similar enrichments in both single‐ and dual‐pharmacophore format (Figure 4c). Similar findings were observed for affinity‐maturation selections against other protein targets, including the tumor‐associated antigens CAIX and tyrosinase^[^ ^18^, ^33^ ^]^ (Figure S32, Supporting Information). In order to confirm that the binding potentiation seen “on DNA” could be translated into small organic ligands devoid of any DNA component, we resynthesized the A24/B137/HIT2 and A200/B44 HSA hits as small organic molecules 16–19 (Figure 4). The best binding combination from the “on DNA” experiments of Figure 4c also gave the best binding results “off DNA,” with an apparent dissociation constant Kd for compound 19 of 56 ± 9 × 10^−9^ m (Figure 4d).

## 3 Conclusion

We have synthesized a novel DNA‐encoded chemical library (GB‐DEL), using a glutamic acid derivative as scaffold for the simultaneous display of two sets of building blocks. The library was used to isolate and characterize small molecule ligands against multiple target proteins. The strategic choice to encode the GB‐DEL with DNA in single‐stranded format opens the possibility to perform selection schemes based on photo‐crosslinking (e.g., by hybridization with oligonucleotide‐photosensitizer conjugates^[^ ^9^, ^34^ ^]^ and to implement affinity‐maturation with oligonucleotide‐hit conjugates (Figure 4). Some of the hit compounds isolated from the GB‐DEL may be useful in practice and may deserve additional medicinal chemistry optimization.

We have discovered two novel binders of FAN1, a versatile DNA nuclease involved in ICL repair and replication stress response. The two molecules (compounds 6 and 7) bound with high affinity (Kd in the 300 × 10^−9^ m range) to both wt and nuclease‐dead FAN1 variants (amino acids 373–1008) and exhibited only poor endonuclease inhibitory activity, suggesting that the binding site might map to a different portion of the protein. Besides the C‐terminal nuclease domain, other structural regions of the FAN1 fragment used in the selections include the SAP (SAF‐A/B, Acinus and PIAS) motif and the TPR (tetratricopeptide repeat) module responsible for homo‐dimerization and DNA‐binding.

We have also isolated ligands 8 and 9 which bound tightly to AASS (alpha‐aminoadipic semialdehyde synthase), an enzyme involved in the major lysine degradation pathway^[^ ^35^ ^]^ implicated as therapy for pyridoxine‐dependent epilepsy (PDE). The pathogenic driver of PDE is the accumulation of *α*−aminoadipate semialdehyde (AASA; a substrate for ALDH7A1)^[^ ^36^ ^]^ and of its cyclic form L‐Δ1‐piperidine‐6 carboxylate (P6C). P6C reacts with pyridoxal 5’‐phosphate and reduces the availability of this essential cofactor for normal cellular functions.^[^ ^37^ ^]^ The inhibition of AASS, upstream of the ALDH7A1 pathway, may allow to reduce AASA/P6C levels and thus serve as substrate reduction therapy for the treatment of PDE.^[^ ^24b^ ^]^

PI3K selections have revealed an intriguing contribution of the library scaffold's chirality for protein recognition, with a ten‐fold improved affinity for compound 11 over its stereoisomer 10. The protein target is a member of the lipid kinases family, which regulates signaling pathways important for cell proliferation, adhesion, survival, and motility.^[^ ^38^ ^]^ The PIK3CA gene, coding for the p110*α* subunit, is frequently mutated in cancer ^[^ ^39^ ^]^ and represents an attractive target for the development of small molecule inhibitors, some of which are currently in preclinical evaluation and some are approved therapeutics.^[^ ^40^ ^]^

The GB‐DEL features DNA in single‐stranded DNA format, which may facilitate its hybridization with chemically modified complementary oligonucleotides. This feature may be useful for a number of applications, including the execution of DEL selections with photocrosslinkers.^[^ ^9a^ ^]^ Small ligands isolated from DELs often display suboptimal kinetic dissociation constants and may be lost during washing procedures in the screening experiments. The display of a photocrosslinker on the complementary strand may help stabilize complex formation and facilitate the recovery of preferential binders.^[^ ^9^, ^41^ ^]^

The stable non‐covalent assembly of complementary DNA structures also facilitates the lead expansion of hit compounds. Our group had previously described the use of encoded self‐assembling chemical (ESAC) libraries as an avenue for the discovery of synergistic pairs of building blocks that may interact with the cognate target in a bidentate fashion and provide an affinity gain through the “chelate effect”.^[^ ^13^, ^42^ ^]^ The affinity maturation strategy described in this article represents an extension of the ESAC strategy, leading to the identification of three chemical moieties which may synergistically interact with the target protein of choice. The procedure was exemplified for the affinity maturation of ligands for the CREBBP bromodomain and HSA. Compound re‐synthesis in the absence of DNA, combined with a judicious linker optimization, may lead to an additional gain in binding affinity (e.g., Kd = 56 ± 9 × 10^−9^ m for the HSA binder). Indeed, the molecular features of the linker may positively or negatively affect the affinity constant of the resulting bidentate ligand.^[^ ^30^ ^]^

In spite of its relatively small size (366 600 compounds), the GB‐DEL generated hits against multiple target proteins of pharmaceutical interest. The glutamic acid scaffold provided a directional display of functional group pairs which could synergistically interact with the cognate protein. The reactions used for library synthesis proceeded with excellent conversion yields,^[^ ^10a^ ^]^ contributing to the overall quality and reproducibility of screening experiments which were conducted in triplicate. The single‐stranded DNA format used for library encoding is likely to find a broader applicability in the future, not only because of its versatility but also because it often yields better selection results compared to the double‐stranded DNA format in terms of ligand recovery and signal‐to‐noise ratios.^[^ ^43^ ^]^

## Conflict of Interest

D.N. is co‐founder and shareholder of Philochem AG (http://www.philochem.com), a company active in the field of DNA‐encoded chemical libraries.

## Supporting information

Supporting Information
