# Supplementary material for: A Single‐Stranded DNA‐Encoded Chemical Library Based on a Stereoisomeric Scaffold Enables Ligand Discovery by Modular Assembly of Building Blocks
Source: Adv Sci (Weinh). 2020 Oct 14;7(22):2001970. doi: 10.1002/advs.202001970 (PMC7675038; doi:10.1002/advs.202001970)

© 2020 Wiley-VCH GmbH

ADVANCED SCIENCE
Open Access

Supporting Information

for *Adv. Sci.,* DOI: 10.1002/advs.202001970

A single-stranded DNA-encoded chemical library based on a stereoisomeric scaffold enables ligand discovery by modular assembly of building blocks

Gabriele Bassi, Nicholas Favalli, Miriam Vuk, Marco Catalano, Adriano Martinelli, Anika Trenner, Antonio Porro, Su Yang, Chuin Lean Tham, Mustafa Moroglu, Wyatt W. Yue, Stuart J. Conway, Peter K. Vogt, Alessandro A. Sartori, Jörg Scheuermann, Dario Neri**

Supporting Information

"A single-stranded DNA-encoded chemical library based on a stereoisomeric scaffold enables ligand discovery by modular assembly of building blocks"

Gabriele Bassi[1], Nicholas Favalli[1], Miriam Vuk[1], Marco Catalano[1], Adriano Martinelli[1], Anika Trenner[2], Antonio Porro[2], Su Yang[3], Chuin Lean Tham[4], Mustafa Moroglu[5], Wyatt W. Yue[4], Stuart J. Conway[5], Peter K. Vogt[3], Alessandro A. Sartori[2], Jörg Scheuermann*[1], Dario Neri*[1]

AFFILIATIONS
1) Department of Chemistry and Applied Biosciences, Swiss Federal Institute of Technology (ETH Zürich), Zürich, Switzerland 2) Institute of Molecular Cancer Research, University of Zürich, Switzerland 3) Scripps Research Institute, Department of Molecular Medicine, La Jolla, California 92037, USA. 4) Structural Genomic Consortium (SGC), Nuffield Department of Medicine, University of Oxford, UK 5) Department of Chemistry, Chemistry Research Laboratory, University of Oxford, Mansfield Road, Oxford, OX1 3TA, UK.

*) Corresponding Authors
J.S.: Tel: +41-44-6337774; e-mail: joerg.scheuermann@pharma.ethz.ch
D.N.: Tel: +41-44-6337401; e-mail: dario.neri@pharma.ethz.ch

Keywords:
DNA-encoded chemical libraries, encoded self-assembling chemical libraries, FAN-1 (FANCD2-associated nuclease 1), AASS (Human alpha-aminoadipic semialdehyde synthase), PI3K (phosphatidylinositol 3-kinase), CREBBP (cyclic-AMP response element binding protein.

1. List of abbreviations....................5
2. General Remarks and procedures....................7
3. Synthesis of Glu-DEL scaffold (Sc)....................10
3.1. Synthesis of compound 2: (R/S) 5-(tert-butyl) 1-methyl 2-azido pentanedioate.................... 10
3.2. Synthesis of compound 3: (*R*+*S*) 4-azido-5-methoxy-5-oxopentanoic acid.................... 10
4. Library synthesis, characterization and purification....................11
4.1. General strategies for oligonucleotides conjugation.................... 11
4.1.1. on-DNA amide bond formation (50 nmol scale)....................11
4.1.2. on-DNA amide bond (0.3nmol scale)....................11
4.1.3. On-DNA methylester deprotection....................11
4.1.4. on-DNA Reverse amide bond reactions (acid attached on-DNA) for primary and secondary amines....................12
4.1.5. on-DNA Reverse amide bond reactions (Acid attached on-DNA) for aromatic derivatives....................12
4.1.6. on-DNA Staudinger reduction....................13
4.1.7. on-DNA CuAAC click reaction....................13
4.2. Library Step 1.................... 14
4.2.1. Generation and purification of three hundred CodeA -N3-Sc-CONHR....................14
4.2.2. LC characterization of pool step 1....................21
4.3. Library step 2..................... 22
4.3.1. Staudinger reduction of library step 1 pool....................22
4.3.2. Pool step 1 splint ligation with Code B oligonucleotides (611 reactions)....................23
4.3.3. Test reactions with building blocks B.....................25
4.3.4. Encoded pool step 1 reactions with building blocks B (611 reactions)....................26
4.3.5. HPLC purification of step 2 (pool step 2)....................40
4.3.6. 2 % Agarose gel of pool step 2 after HPLC purification....................41
4.4. Library design.................... 41
5. Dual pharmacophore 2+1 affinity maturation libraries....................42
5.1. Synthesis and characterization of known ligands (HIT 1-5) to protein targets.................... 42
5.1.1. CREBBP ligand HIT1....................43
5.1.2. Human serum albumin (HSA) ligand HIT2....................48
5.1.3. Carbonic anhydrase IX ligand HIT3....................48
5.1.4. Tyrosinase ligand HIT4....................48
5.1.5. Synthesis of trypsin ligand HIT5....................49
5.2. Conjugation of HITs to 24mer DNA (DB24).................... 50
5.3. Formation of dual pharmacophore 2+1 affinity maturation libraries.................... 52
6. Affinity selections....................54
6.1. Selection procedure.................... 54
6.2. Proteins for affinity selections.................... 54
6.2.1. CAIX....................55
6.2.2. CREBBP bromodomain....................55
6.2.3. PI3K....................56
6.2.4. Streptavidin....................56
6.2.5. AASS....................57
6.2.6. Trypsin....................58
6.2.7. D960A FAN-1.....................59
6.2.8. wt FAN-1....................60
6.2.9. Human serum albumin....................60

6.2.10. Tyrosinase 60
6.2.11. Protein biotinylation. 60

6.3. PCR amplification and sequencing 60

6.4. 2BB single pharmacophore selections replicates 62
6.4.1. Naïve library 62
6.4.2. Streptavidin fingerprints 62
6.4.3. CAIX fingerprints 63
6.4.4. D960A FAN1 fingerprints 63
6.4.5. AASSA fingerprints 64
6.4.6. PI3K fingerprints 64
6.4.7. Albumin fingerprints 65
6.5. Enrichment factors calculation 65

7. Nuclease Activity assay 69

8. Selection experiments with affinity maturation dual pharmacophore 2+1 DELs 70

9. Single pharmacophore library: re-synthesis of selection hits 74

9.1. Solid phase synthesis 74
9.1.1. General procedure of Fmoc Deprotection 74
9.1.2. General procedure of amino-acid loading 74
9.1.3. TNBS test 74
9.1.4. General procedure of amide coupling 74
9.1.5. General procedure of diazo-transfer. 75
9.1.6. General procedure of CuAAC. 75
9.1.7. General procedure of Alloc protective group deprotection 75
9.1.8. General procedure of resin cleavage. 75
9.1.9. Reverse amide bond formation (COOH on resin). 76

9.2. Linker synthesis 76
9.2.1. Synthesis of Resin-(S)-FmocNH-glu(OtBu)-COOAllyl R2 76
9.2.2. Synthesis of FITC-labelled negative control (Fluo-NH_2) 76
9.3. Synthesis of streptavidin binder: (S)-COOH-Glu/(R,S)-Glu-A8/B264 77

9.4. Synthesis of FAN-1 binders: (*S*)-COOH-glu/(*R,S*)-A20/B611 79
9.5. Synthesis of AASSA binders: (*S*)-COOH-glu/(*R,S*)-A120/B235 81

9.6. Synthesis of wt-PI3K binders 83
9.6.1. Synthesis of (S)-COOH-glu/(R,S)-A86/B371 83
9.6.2. Synthesis of (*S*)-COOH-glu/(*R,S*)-A146/B537 84
9.6.3. Synthesis of (*S*)-COOH-glu/(*R,S*)-A245/B474 86

9.7. Synthesis of HSA binder 87
9.7.1. (S)-COOH-glu/(R,S)-A200/B44 87

10. Dual pharmacophore libraries: re-synthesis of affinity maturation hits 90

10.1. On-DNA Dual Pharmacophore hits re-synthesis 90
10.1.1. CREBBP 2+1 affinity maturation binder 90
10.1.2. HSA 2+1 affinity maturation binder 92

10.2. Off-DNA dual pharmacophore hits re-synthesis 93
10.2.1. Synthesis of compound 18 allyl ester 93
10.2.2. Synthesis of compound 19 allyl ester 95

11. Hit Validation 97

11.1. Synthesis of fluorescently labelled ligands 97

12. References 98

13. Appendix I: NMR data 99
14. Appendix II: LC-MS data 117

1. List of abbreviations

AASS: Human alpha-aminoadipic semialdehyde synthase

AC: average counts (AC = total counts / library size);

Boc = tert-Butyloxycarbonyl;

CAIX: Carbonic Anhydrase IX;

CREBBP: cAMP-response element binding protein;

CuAAC: Copper-Catalyzed Azide-Alkyne cycloaddition;

DCM: dichloromethane;

DIPEA: *N*, *N*'-diisopropylethylamine;

DMF: *N*, *N*'-dimethylformamide;

DMSO: dimethyl sulfoxide;

EDC: 1-ethyl-3- (3-dimethylaminopropyl) carbodiimide;

EF: enrichment factor;

ELISA: enzyme-linked immunosorbent assay;

FA: formic acid;

FAN 1: FANCD2-associated nuclease 1;

FITC: Fluorescein isothiocyanate isomer I;

Fmoc: 9-fluorenylmethyloxycarbonyl;

FP: fluorescence polarization;

HATU: (1-[Bis(dimethylamino)methylene]-1H-1,2,3-triazolo[4,5-b]pyridinium 3-oxide hexafluorophosphate;

HSA: Human Serum Albumin;

$ImSO_3N_3$ = Imidazole-1-sulphonyl-azide;

PAGE: polyacrylamide gel electrophoresis;

PB: protein buffer;

PBS: phosphate buffered saline;

PI3K: Phosphoinositide 3-kinases

Pip : Piperidine;

RP: reverse phase

sNHS: *N*-hydroxysulfosuccinimide sodium salt;

TCEP-HCl: tris(2-carboxyethyl)phosphine hydrochloride;

TEA: triethylamine;

TEAA: triethylammonium acetate;

TFA: trifluoroacetic acid;

TMB: 3,3′,5,5′-Tetramethylbenzidine;

Tris-HCl: tris(hydroxylmethyl) aminomethane hydrochloride;

wt-PI3K: *wildtype* p110α/p85α PI3K;

2. General Remarks and procedures

Reagents: All compounds and chemical reagents were obtained from Sigma-Aldrich, TCI Europe, Enamine or ABCR, and used without further purification. DNA and LNA™ starting materials were purchased from IBA Life sciences.

Water was purified with a Millipore Milli-Q system (Merck). Ligation buffer, DNA-ligase™ and high-fidelity Phusion™ DNA polymerase were purchased from New England Biolabs. PCR purification and gel extraction kits were purchased from Qiagen. All gel images were captured by a Bio-Rad Chemidoc image system. Rink amide resin was purchased from Sigma-Aldrich.

Purification: Preparative reversed-phase HPLC for the DNA and LNA conjugates was performed on a Waters Alliance HT RP-HPLC with PDA-UV detector, using a Synergi 4 µm, polar-RP 150 × 10 mm C18 column using a gradient of eluent A (TEAA 100 mM) and eluent B (TEAA 100 mM in 80% ACN). Semi preparative reversed-phase high-pressure liquid chromatography (HPLC) for the compounds was performed on a Waters Alliance HT RP-HPLC with PDA-UV detector, on Synergi 4 uM polar-RP 80 (150X10mm) column with mQ Millipore water 0.1%Formic Acid (buffer A) and Acetonitrile 0.1% formic acid (Buffer B) as mobile phase. Proton (^{1}H) nuclear magnetic resonance (NMR) spectra were record on a Bruker AV400 (400 MHz). Carbon (^{13}C) NMR spectra were recorded on a Bruker AV400 (100 MHz) spectrometer. Shifts are given in ppm using residual solvent as the internal standard. Coupling constants (*J*) are reported in Hz with the following abbreviations used to indicate splitting: s = singlet, d = doublet, t = triplet, dd = doublet of doublets, m = multiplet. Analytical LC traces were registered using a Xevo G2-XS QTof LC-MS (Waters).

Statistical analysis: All the affinity constant values have been calculated from triplicate measurements (n = 3) and the results are presented as mean ± standard error. Standard deviation, mean and standard error on mean have been fitted using Graphpad Prism 8.

Small molecule enzyme-linked immunosorbent assay (ELISA)

The proteins FAN-1, AASSA, PI3K (100 µL/well, 200 nM) were incubated overnight at 4°C on a F8 maxisorp (Thermo fisher scientific) plate. The protein was blocked by the addition of 4% milk in PBS (200 µL/well, 30 min (RT)) then washed with PB (3x, 200 µL/well). The immobilized protein was incubated for 30 minutes in the dark with serial dilutions of FITC-labelled

compound and washed with PB (3x, 200 µL/well). A solution of 200 nM anti-fluorescein antibody (IgG1)[1] in 2% milk-PB was added to each well (100 µL/well) and incubated for additional 30 minutes in the dark. The resulting complex was washed with PB (3x, 200 µL/well) and incubated for additional 30 minutes with Protein A-HRP (1µg/mL in 2% Milk-PB, 100µL/well). The wells were then washed with PB 0.1% Tween (3x, 200 uL/ well) and with PB (3x, 200 uL/ well). The substrate (3,3′,5,5′-Tetramethylbenzidine, TMB) was added (100 µL /well) and developed in the dark for 1-5 minutes. The reaction was stopped by adding 50 µL of 1 M sulphuric acid. The absorbance was measured on a Spectramax paradigm multimode plate reader (Molecular Devices) at 620-650nm and 450nm. All the experiments were performed in triplicate and the resulting data was statistically evaluated using Prism 8. The Kd values were obtained by fitting log concentration vs. anisotropy.

Fluorescence Polarization Assays (On-DNA)

A12-LNA and B12-DNA conjugated strands were annealed as 1 µM solutions in 1:1.5 ratio in PB (pH-7.4). The annealing was performed at 65°C for 4 minutes and then left cooling down at RT for ~1 hour. The dilution series were prepared in a non-binding black 384-well microplate (Greiner Bio One). In a final volume of 30 µL, 10 nM of the fluorophore-labelled double stranded DNA-LNA hybrids were incubated with a 1:1 dilution series of protein HSA (initial concentration 50 µM), and CREBBP (initial concentration 10 uM) in PBS pH-7.4 for 2 hours at 37°C. The fluorescence anisotropy was measured on a Spectramax Paradigm multimode plate reader (Molecular Devices). Experiments were performed in triplicate and the anisotropy values fitted using Graphpad Prism 8.

Fluorescence Polarization Assays (Off-DNA)

The protein dilution series was prepared in a non-binding black 384-well microplate (Greiner Bio One). Proteins were serially diluted in PB buffer. In a final volume of 30 uL, 25 nM of the fluorescently labelled small molecules were incubated at a 1:1 ratio with HSA (25 uM) for 30 minutes (RT) in the dark. The fluorescence anisotropy was measured on a Spectramax Paradigm multimode plate reader (Molecular Devices). Experiments were performed in triplicate and the anisotropy values fitted using Graphpad Prism 8.

3. Synthesis of Glu-DEL scaffold (Sc)

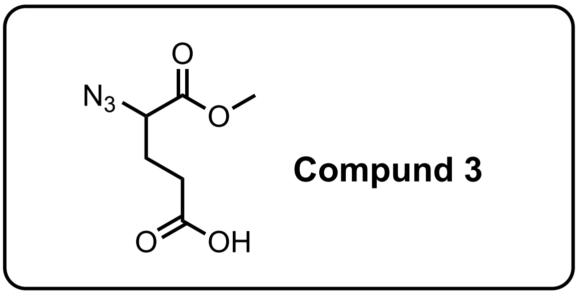

3.1. Synthesis of compound 2: (R/S) 5-(tert-butyl) 1-methyl 2-azido pentanedioate

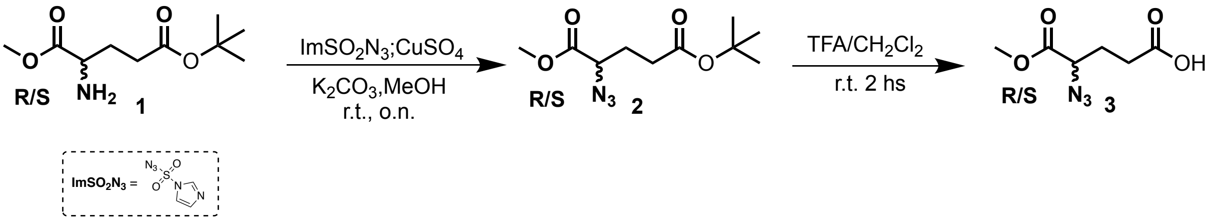

Supplementary figure 1: Schematic representation of scaffold's synthesis (**3)**

An equimolar mixture of the commercially available R and S 5-(tert-butyl) 1-methyl glutamate was prepared. 662g (2.62 mmol) of the mixture was dissolved in dry methanol (10 mL) and Imidazole-1-sulphonyl-azide hydrochloride (**$ImSO_2N_3$**, 850 mg, 4 mmol), potassium carbonate anhydrous (1.17 g, 8.5 mmol) and anhydrous copper sulfate (25 mg, 0.013 mmol) were added to the reaction, the resulting mixture was stirred at RT for 24 hours. The reaction crude was filtered, concentrated under reduced pressure and the products were extracted with ethyl acetate. The organic layers were dried over Mg_2SO_4 and concentrated. Flash chromatography Hexane/AcOEt 9:1 (Rf=0.25) yielded product **2**.

3.2. Synthesis of compound 3: (*R*+*S*) 4-azido-5-methoxy-5-oxopentanoic acid

Compound **2** was dissolved in a solution 9:1 CF_3COOH/CH_2Cl_2 and left under stirring for 2 hours. The mixture was evaporated, suspended in MeOH and filtered. The crude product was purified by RP-HPLC to afford compound **3**. **^{1}H NMR** (400 MHz, Chloroform-d) δ 4.05 (dd, J = 8.7, 5.1 Hz, 1H), 3.82 (s, 3H), 2.73 – 2.45 (m, 2H), 2.28 – 1.88 (m, 2H). **^{13}C NMR** (101 MHz, DMSO) δ 173.81, 170.81, 60.93, 52.82, 30.03, 26.57. **MS** calcd: (m/z) $[M^+H^+]$ $C_6H_9N_3O_4$ = 187.0593.

4. Library synthesis, characterization and purification

4.1. General strategies for oligonucleotides conjugation

4.1.1. on-DNA amide bond formation (50 nmol scale)

In a 1.5 mL Eppendorf tube 42 uL (100 mM DMSO, 85eq.) of the desired carboxylic acid were mixed for 30 minutes with 90uL DMSO, 20 uL (S-NHS 100 mM DMSO/H_2O 2:1, 40eq.), 42 uL (EDC 100 mM DMSO, 84 eq.). In a separate well 50 uL DNA (1 nmol/uL, 1eq.) in H_2O were mixed with 50 uL of TEA buffer (100 mM, pH=10). The activated carboxylic acid solution was added to the oligo solution and stirred at room temperature over-night. The reacted DNA was precipitated by adding 30 uL (NaCl 5 M) and 30 uL (AcOH/$NaAcO^-$ 3 M, pH= 4.7) followed by 900 uL EtOH absolute. The precipitated DNA was kept at -20°C for 4hours and centrifuged a 4°C for 30mins at 16.1 krpm. The supernatant was discarded and the pellet dried under vacuum.

4.1.2. on-DNA amide bond (0.3nmol scale)

In a 96-well plate, 8 uL DNA (0.3 nmol) in TEA (50 mM pH= 10) were added to each well. The carboxylic acid solution was prepared separately by adding to 10 uL (60 mM carboxylic acid), 4.7 uL (EDC 100 mM/S-NHS 100mM, 2.1:1, followed by stirring for 30 minutes. The activated carboxylic acid solution was added to the oligo solution and stirred over-night. The reacted DNA was precipitated by adding 2.5uL (NaCl 5M) and 2.5uL (AcOH/AcO^- 3M pH=4.7) followed by 100uL EtOH. The precipitated DNA was kept at -20°C for 4 hours and centrifuged at 4°C for 30 mins at 16.1 krpm. The supernatant was discarded and the pellet dried under vacuum.

4.1.3. On-DNA methylester deprotection

In a 2 mL Eppendorf tube, DNA (50 nmol) was dissolved in 250 uL H_2O, 250 uL LiOH (200 mM) were added and the resulting mixture was stirred at 25°C for 4 h. The reaction was monitored by LC-MS. After the reaction was completed, LiOH was quenched by adding 16 uL AcOH (3

M). The reacted DNA was precipitated by adding 50 uL 5 M NaCl and 1375 uL EtOH. The precipitated DNA was kept at -20°C for 4 hours and centrifuged at 4°C for 30 mins at 16.1 krpm. The supernatant was discarded and the pellet dried in a speed vacuum machine.

4.1.4. on-DNA Reverse amide bond reactions (acid attached on-DNA) for primary and secondary amines

In a 2 mL Eppendorf tube DNA (50 nmol, 1 eq) was dissolved in 40 µL H_2O. 137 µL of MOPS buffer (100 mM MOPS, 1 M NaCl, pH= 7.0) was added, followed by 30 µL of DMT-MM (500 mM in MOPS buffer, 300eq). The resulting mixture was stirred at 30 °C for 30 min. 75 µL (100 mM, 150 eq) of the desired amine was added to the activation solution and stirred at 30°C for 16 hours. The reacted DNA was precipitated by adding 28 uL 5 M NaCl and 775 uL EtOH. The precipitated DNA was kept at -20°C for 4hours and centrifuged at 4°C for 30mins at 16.1 krpm. The supernatant was discarded and the pellet dried in a speed vacuum machine.

4.1.5. on-DNA Reverse amide bond reactions (Acid attached on-DNA) for aromatic derivatives

In a 2 mL Eppendorf tube DNA (50 nmol, 1 eq) was dissolved in 40 µL H_2O. 137 µL of MOPS buffer (100 mM MOPS, 1 M NaCl, pH= 7.0) was added, followed by 30 µL of DMT-MM (500 mM in MOPS buffer, 300 eq). The resulting mixture was stirred at 45 °C for 30 min. 75 µL (100 mM, 150 eq) of the desired amine were added to the activation solution and stirred at 45°C for 16 hours. The reacted DNA was precipitated by adding 28 uL 5 M NaCl and 775 uL EtOH. The precipitated DNA was kept at -20°C for 4 hours and centrifuged at 4°C for 30 mins at 16.1 krpm. The supernatant was discarded and the pellet dried in a speed vacuum machine.

4.1.6. on-DNA Staudinger reduction

In a 2 mL Eppendorf tube DNA (1-100 nmol,1 eq) was dissolved in 100 µL of TCEP buffer (30 mM TCEP, in 500 mM TRIS*HCl, pH= 7.4). The resulting mixture was stirred at 30 °C for 4 hours. Reaction completion was monitored by LC-MS. The reacted DNA was precipitated by adding 25 uL 5 M NaCl and 375 uL EtOH. The precipitated DNA was kept at -20°C for 4hours and centrifuged a 4°C for 30 mins at 16.1 krpm. The supernatant was discarded and the pellet dried under vacuum.

4.1.7. on-DNA CuAAC click reaction

In a 96-well plate to 2.5 uL DNA (0.3 nmol), 30 uL (Borate buffer pH= 9.5) followed by 2.5 uL $CuSO_4$ (5 mM in H_2O, 42 eq) and 10 uL of the desired alkyne (10 mM in DMSO, 100 eq) were added. The reaction was then started by the addition of 2.5 uL sodium ascorbate (5 mM in H_2O, 42 eq) and stirred at room temperature overnight. The reacted DNA was precipitated by adding 12 uL 5 M NaCl and 240 uL EtOH. The precipitated DNA was kept at -20°C for 4 hours and centrifuged a 4°C for 30 mins at 16.1 krpm. The supernatant was discarded and the pellet dried under vacuum.

4.2. Library Step 1

4.2.1. Generation and purification of three hundred CodeA -N3-Sc-CONHR

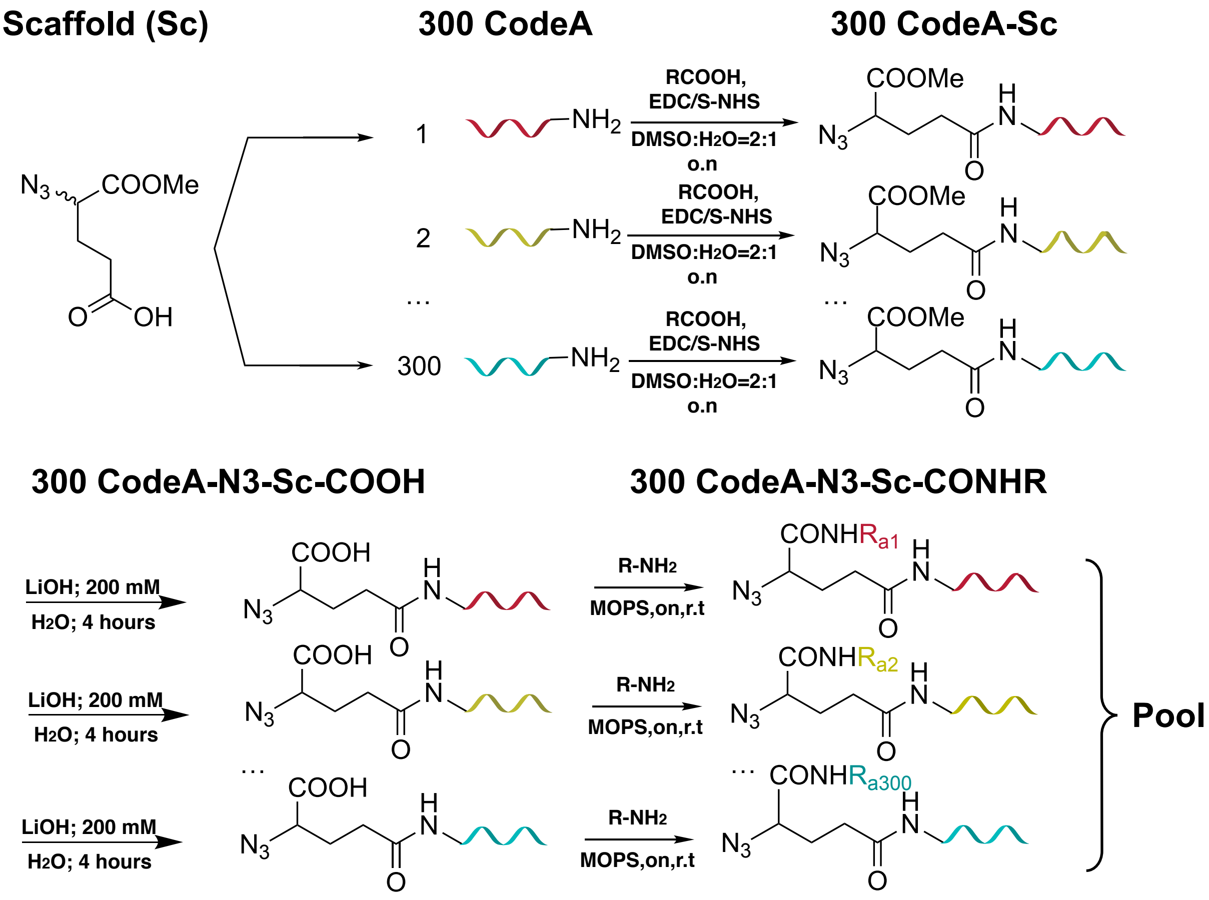

Supplementary figure 2: Schematic representation of the synthetical steps involved in the formation of three hundred unique **CodeA-N3-Sc-CONHR.**

A 100 mM stock solution of compound **3** was prepared and reacted with three hundred different oligonucleotides following the general procedure **4.1.1** to yield three hundred **CodeA-Sc** derivatives**.** A 43mer-C6-NH_2 oligonucleotide was used for the reactions (NH2-C6-5'-GGAGCTTCTGAATTCTGTGTGTGCTGXXXXXXCGAGCGTCAGGCAGC-3') bearing the XXXXXX coding region**.**

Each Eppendorf tube was then subsequently treated with LiOH to remove the methyl ester following the procedure described in **4.1.3.** The three hundred different **CodeA-N_3-Sc-COOH** were reacted with three hundred different amines following protocol **4.1.4** in case of aliphatic primary and secondary amines. Conditions reported in section **4.1.5** were used for aromatic amines. After individual RP HPLC, lyophilization, precipitation, centrifugation, solvent removal

and drying, the encoded three hundred **CodeA-N$_3$-Sc-CONHR** were obtained in variable yields. A detailed list of the three hundred **CodeA-N$_3$-Sc-CONHR** is reported in **supplementary table 1**. The conjugates were pooled in equimolar amounts and dried under reduced pressure.

Supplementary table 1: Building block A (amines) used for library construction.

CdId	Structure	M.W.	Formula	MW calculated	MW obs.	LogP
1	NCC1=CC=CS1	113.18	C5H7NS	14'328.28	14'332	1.01
2	COC1=CC=C(OC)C(CN)=C1	167.21	C9H13NO2	14'440.31	14'444	0.78
3	NCCCSCC1=CC=C(F)C=C1	199.29	C10H14FNS	14'414.39	14'418	2.15
4	NCC1(CC1)C1=CC=CC=C1	147.22	C10H13N	14'378.32	14'382	1.67
5	CC(C)(CN)CN1CCCCCC1	184.33	C11H24N2	14'376.33	14'380	1.72
6	C1CCNCC1	85.15	C5H11N	14'310.25	14'314	0.66
7	NC1CCCCCC1	113.20	C7H15N	14330.20	14'334	1.62
8	COC1=CC(CCN)=C(OC)C=C1	181.24	C10H15NO2	14'421.34	14'425	1.07
9	NCC1CC1	71.12	C4H9N	14'281.12	14'285	0.16
10	CN1N=C(C)C(CN)=C1C	139.20	C7H13N3	14'380.30	14'384	-0.14
11	CCN1CCCC1CN	128.22	C7H16N2	14375.32	14'379	0.22
12	C1CN(CCN1)C1=NC=CC=N1	164.21	C8H12N4	14'374.21	14'378	0.30
13	CC(CN)C1=CC=CC=C1	135.21	C9H13N	14391.31	14'395	1.75
14	NCCN1CCCCC1	128.22	C7H16N2	14377.32	14'381	0.24
15	NCC1CCCCC1	113.20	C7H15N	14'339.20	14'343	1.49
16	NCC1=CC(=CC=C1)C(F)(F)F	175.15	C8H8F3N	14'424.25	14'428	1.98
17	NCC1=CC=C(Cl)C(Cl)=C1	176.04	C7H7Cl2N	14'392.04	14'396	2.31
18	NCC1=C(C=CC=C1)C(F)(F)F	175.15	C8H8F3N	14'390.25	14'394	1.98
19	NCC1=CC=CC(=C1)N1CCOCC1	192.26	C11H16N2O	14'392.26	14'396	0.99
20	CC1=NC=CN1C1=CC=C(CN)C=C1	187.25	C11H13N3	14'451.35	14'455	0.99
21	CC1=NC=CN1C1=C(CN)C=CC=C1	187.25	C11H13N3	14'427.35	14'431	0.99
22	NCC1=CC(F)=C(F)C=C1	143.14	C7H7F2N	14'432.24	14'436	1.38
23	NCCN1CCCC(C1)C1=CC=CC=C1	204.32	C13H20N2	14'460.42	14'464	1.74
24	CC(C)(C)C1=CC=C(CN)C=C1	163.26	C11H17N	14'388.36	14'392	2.64
25	NCC1=CSC(=N1)C1=CC=CS1	196.29	C8H8N2S2	14'461.39	14'465	1.64
26	NCC1=C(C=CC=C1)N1CCCC1	176.26	C11H16N2	14'456.36	14'460	1.61
27	NCC1=CC=C(F)C=C1	125.15	C7H8FN	14'390.25	14'394	1.24
28	NCC1CCN(CC1)C1=NC=CC=N1	192.27	C10H16N4	14'449.37	14'453	0.51
29	NCC1=CC=CN=C1	108.14	C6H8N2	14299.14	14'303	-0.12
30	NCC1=CC=C(N=C1)C1=CC=CC=C1	184.24	C12H12N2	14'400.24	14'404	1.91
31	CC1=NN(N=C1CN)C1=CC=CC=C1	188.23	C10H12N4	14'428.33	14'432	0.64
32	CC(N)C1=CC=CC=C1	121.18	C8H11N	14'321.18	14'325	1.52
33	NCC1(CCOCC1)C1=CC=CS1	197.30	C10H15NOS	14'412.30	14'416	1.22
34	Cl.Cl.NC1CN2CCC1CC2	199.12	C7H16Cl2N2	14'382.20	14'386	-0.25
35	CC1(C)C2CCC1(C)C(N)C2	153.27	C10H19N	14'362.27	14'366	1.88
36	CCN(CC)CCNC	130.24	C7H18N2	14404.34	14'408	0.54
37	CNCC1=CC=CC=C1	121.18	C8H11N	14'392.18	14'396	1.53
38	CN1C=C(CN)C2=CC=CC=C12	160.22	C10H12N2	14'386.22	14'390	1.42
39	CCN1CCNCC1	114.19	C6H14N2	14'364.19	14'368	0.01
40	CCOC(=O)N1CCNCC1	158.20	C7H14N2O2	14'414.20	14'418	-0.14
41	C[C@H](N)C(C)(C)C	101.19	C6H15N	14'341.19	14'345	1.41
42	C1CC2=C(CN1)C=CC=C2	133.19	C9H11N	14'349.19	14'353	1.57
43	NCC1CCCO1	101.15	C5H11NO	14'326.15	14'330	-0.21
44	NC1CCCC2=C1C=CC=C2	147.22	C10H13N	14'347.22	14'351	2.09
45	NC(C1=CC=CC=C1)C1=CC=CC=C1	183.25	C13H13N	14'408.25	14'412	2.88
46	CN(CC1=C(N)C=CC=C1)C1CCCCC1	218.34	C14H22N2	14'468.44	14'472	2.88

47	NCCC1=CC(=CC=C1)C(F)(F)F	189.18	C9H10F3N	14'420.28	14'424	2.27
48	NCCC1=CC(Br)=CC=C1	200.08	C8H10BrN	14'426.08	14'430	2.16
49	CC1=CC=CC=C1CCN	135.21	C9H13N	14'366.31	14'370	1.90
50	NC1=CC=C(OC2=CC=CC=C2)C=C1	185.23	C12H11NO	14'362.23	14'366	2.64
51	NCCC1=CC=CC=C1	121.18	C8H11N	14'386.28	14'390	1.39
52	CC(C)OC1=CC=CC(CCN)=C1	179.26	C11H17NO	14'420.36	14'424	2.00
53	CC1=CC=CC(CCN)=C1	135.21	C9H13N	14'449.31	14'453	1.90
54	Cl.FC(F)(F)C1CCNCC1	189.61	C6H11ClF3N	14'353.15	14'357	1.22
55	Cl.C1CC2CNCC2C1	147.65	C7H14ClN	14'376.29	14'380	0.92
56	O=C(N1CCNCC1)C1=CC=CC=C1	190.25	C11H14N2O	14'446.35	14'450	0.74
57	C1CN(CCN1)C1=CC=NC=C1	163.22	C9H13N3	14378.32	14'382	0.33
58	COC1=CC=C(N)C=C1	123.16	C7H9NO	14'339.16	14'343	0.99
59	COC1=CC=CC=C1N	123.16	C7H9NO	14'378.26	14'382	0.99
60	CC1=CC=CC=C1N	107.16	C7H9N	14'324.16	14'328	1.66
61	NCCS(O)(=O)=O	125.14	C2H7NO3S	14292.14	14'296	-2.61
62	CNCCC1=CC=CC=N1	136.20	C8H12N2	14'375.30	14'379	0.63
63	CN(C)C(=O)N1CCNCC1	157.22	C7H15N3O	14'342.22	14'346	-1.01
64	FC(F)(F)C1CCCNC1	153.15	C6H10F3N	14'375.25	14'379	1.22
65	Cl.NC1C2CC3CC(C2)C[C@@H]1C3	187.71	C10H18ClN	14'438.35	14'442	1.55
66	Cl.NC1CC2=CC=CC=C2C1	169.65	C9H12ClN	14'414.29	14'418	1.49
67	Cl.N#CC1CCNCC1	146.62	C6H11ClN2	14'334.26	14'338	-0.06
68	C1CC(CN1)C1=CC=CC=C1	147.22	C10H13N	14'412.32	14'416	1.71
69	C1CN(CCN1)C1=CC=CC=N1	163.22	C9H13N3	14'364.22	14'368	0.92
70	NCCC1=CC=C(F)C=C1	139.17	C8H10FN	14'444.27	14'448	1.53
71	CCOCCCN	103.17	C5H13NO	14'383.27	14'387	-0.26
72	NC1=CC=CC=C1	93.13	C6H7N	14'348.23	14'352	1.14
73	NC1CCCC1	85.15	C5H11N	14'246.15	14'250	0.73
74	FC1=CC2=C(C=C1)C(=NO2)C1CCNCC<br>1	220.25	C12H13FN2O	14'460.35	14'464	1.73
75	CC(=O)NCCN	102.14	C4H10N2O	14309.14	14'313	-1.60
76	CC(C)(C)OC(=O)NCCN	160.22	C7H16N2O2	14'375.32	14'379	0.07
77	Cl.Cl.CCCN1C=NN=C1CN	213.11	C6H14Cl2N4	14518.21	14'522	-0.80
78	CC(C)C1=CSC(CCN)=N1	170.27	C8H14N2S	14'435.37	14'439	1.44
79	NCC1=NOC(=C1)C1=C(Cl)C=CC=C1	208.65	C10H9ClN2O	14'448.75	14'453	1.72
80	CCOC1=C(OCC)C=C(CCCN)C=C1	223.32	C13H21NO2	14'439.32	14'443	2.23
81	Cl.Cl.CC1=CN2C=C(CN)N=C2C=C1	234.12	C9H13Cl2N3	14'401.31	14'405	0.48
82	NCC1CCCN(C1)C(=O)C1CC1	182.27	C10H18N2O	14'416.37	14'420	-0.09
83	NCC1=CC=C2OCCOC2=C1	165.19	C9H11NO2	14'430.29	14'434	0.61
84	C1CNC2=C(C1)C=CC=C2	133.19	C9H11N	14'349.19	14'353	1.93
85	COC1=C(CCN)C=CC=C1	151.21	C9H13NO	14'456.31	14'460	1.23
86	C1CC2=CC=CC=C2N1	119.17	C8H9N	14'375.27	14'379	1.49
87	NCCP(O)(O)=O	125.06	C2H8NO3P	14'389.16	14'393	-3.17
88	N.Cl	53.49	ClH4N	14'289.13	14'293	-0.98
89	NC1CCCCC1C1CCCCC1	181.32	C12H23N	14'431.42	14'435	3.14
90	Cl.CN	67.52	CH6ClN	14'262.16	14'266	-0.63
91	NC(=O)C1=CC=C(N)C=C1	136.15	C7H8N2O	14'336.15	14'340	-0.01
92	NC1=CC(OC2=CC=CC=C2)=CC=C1	185.23	C12H11NO	14'465.33	14'469	2.64
93	NC1C(O)CC2=CC=CC=C12	149.19	C9H11NO	14'358.29	14'362	0.57
94	NC1=CC=C(C=C1)S(N)(=O)=O	172.20	C6H8N2O2S	14'413.30	14'417	-0.25
95	CC1=CC(N)=CC=C1Br	186.05	C7H8BrN	14'467.15	14'471	2.43
96	NC1=CC=C(C=C1)S(O)(=O)=O	173.19	C6H7NO3S	14'349.19	14'353	0.10
97	NC1=CC=CC(=C1)C(F)(F)F	161.13	C7H6F3N	14'410.23	14'414	2.02
98	CC1=CC=C(N)C=C1F	125.15	C7H8FN	14'357.15	14'361	1.80
99	COC1=CC=C(CN)C=C1	137.18	C8H11NO	14'353.18	14'357	0.94
100	NCC1=CC=CC=C1	107.16	C7H9N	14'323.16	14'327	1.10
101	NCC1(CCSC1)N1CCOCC1	202.32	C9H18N2OS	14'372.32	14'376	-0.26
102	NC1=C(OCC(F)F)C=CC=C1	173.16	C8H9F2NO	14'462.26	14'466	1.45
103	CC1=CC=C(N)C=C1	107.16	C7H9N	14381.26	14'385	1.66
104	NC1=CC(=CC=C1)C#C	117.15	C8H7N	14317.15	14'321	1.30
105	NOC(C1=CC=CC=C1)(C1=CC=CC=C1)<br>C1=CC=CC=C1	275.35	C19H17NO	14524.45	14'528	4.79

106	NC1(CCCCC1)C#C	123.20	C8H13N	14413.30	14'417	1.33
107	NC1=CC2=C(SC=N2)C=C1	150.20	C7H6N2S	14'406.30	14'410	1.28
108	NC1=C(CN2C=NC=N2)C=CC=C1	174.21	C9H10N4	14'448.31	14'452	0.61
109	CC1=NN(C2CCCNC2)C(C)=N1	180.26	C9H16N4	14'454.36	14'458	0.42
110	Cl.N[C@@H]1CCCN(C1)C(=O)CCC(F)(F)F	260.69	C9H16ClF3N2O	14'474.33	14'478	0.42
111	CNCC#C	69.11	C4H7N	14375.21	14'379	0.04
112	CC1=NC(OC2CCNCC2)=CC=C1	192.26	C11H16N2O	14'432.26	14'436	0.97
113	NC1CCN(CC1)C(=O)C1=C(F)C=CC=C1	222.26	C12H15FN2O	14'496.36	14'500	0.63
114	NS(=O)(=O)C1=C2CCNCC2=CC=C1	212.27	C9H12N2O2S	14'427.27	14'431	0.07
115	CC1=C2OC(=NC2=CC=C1)C1CCNCC1	216.28	C13H16N2O	14'521.38	14'525	2.06
116	CN1C=C(C=N1)N1CCCC(N)C1	180.26	C9H16N4	14'437.26	14'441	0.18
117	NC1CCN(CC2=NC3=C(N2)C=CC=C3)CC1	230.32	C13H18N4	14'470.32	14'474	0.33
118	CN1CCC2(CCNCC2)C1=O	168.24	C9H16N2O	14'433.24	14'437	-0.40
119	C(C1=NN=C2CNCCN12)C1=CC=CC=C1	214.27	C12H14N4	14'478.37	14'482	0.54
120	ClC1=CN=C(OC2CCCNC2)C=C1	212.68	C10H13ClN2O	14'406.68	14'411	1.90
121	COC1=CC(=CC(OC)=C1)N1CC(N)CC1=O	236.27	C12H16N2O3	14'428.27	14'432	-0.04
122	CC1=NN(C(C)=N1)C1=C(N)C=CC=C1	188.23	C10H12N4	14'493.33	14'497	1.26
123	NC(CC(N)=O)C1CCCCC1	170.26	C9H18N2O	14'371.26	14'375	0.46
124	NC1CCN(C1)C(=O)COC1=CC=CC=C1	220.27	C12H16N2O2	14'445.27	14'449	0.09
125	C1CC1C1=NNC(=C1)N1CCNCC1	192.27	C10H16N4	14'393.27	14'397	0.85
126	CN1C=C(CN2CCNCC2)C=N1	180.26	C9H16N4	14'430.36	14'434	-0.19
127	N[C@@H]1CCC(=O)N[C@H]1C1=CC=CC=C1	190.25	C11H14N2O	14'415.35	14'419	0.47
128	CC1=C(N)C=C(C=C1)C1=NC=CN=C1	185.23	C11H11N3	14'442.33	14'446	1.26
129	CN1C=C(C=N1)[C@H]1OCCC[C@@H]1N	181.24	C9H15N3O	14'486.34	14'490	0.01
130	CN1N=CC=C1N1CCCC(N)C1	180.26	C9H16N4	14390.26	14'394	0.27
131	O=C1C=CC=CN1CCN1CCNCC1	207.28	C11H17N3O	14'454.38	14'458	-0.28
132	Cl.Cl.NC1CCN2CCCCC2C1	227.17	C9H20Cl2N2	14452.27	14'456	0.31
133	NC1CCN(CC1)C1=C(C=CC=C1)C(N)=O	219.29	C12H17N3O	14'435.29	14'439	0.14
134	C1CN(CCN1)C1=C2C=CC=CC2=NC=C1	213.28	C13H15N3	14'469.38	14'473	1.70
135	CC1CCCC(N)C1C	127.23	C8H17N	14319.23	14'323	1.83
136	Cl.Cl.C1CNC(C1)C1=NN=C2C=CC=CN12	261.15	C10H14Cl2N4	14'468.33	14'472	0.05
137	CNC(=O)C1=CC(N)=C(OC)C=C1	180.21	C9H12N2O2	14'421.31	14'425	0.06
138	NC1=CC(CN2CCNC(=O)C2)=CC=C1	205.26	C11H15N3O	14'467.36	14'471	-0.19
139	NCCC1=CC=CS1	127.21	C6H9NS	14414.31	14'418	1.30
140	FC(F)(F)COC1CCCNC1	183.17	C7H12F3NO	14'448.27	14'452	1.18
141	CN1CCN(C)C(=O)C2=C1C=C(N)C=C2	205.26	C11H15N3O	14'429.36	14'433	0.38
142	NCC1CCCN2CCCCC12	168.28	C10H20N2	14'433.10	14'437	0.81
143	CC(CN)(C1=CC=CC=C1)C1=CC=CC=C1	211.31	C15H17N	14412.31	14'416	3.26
144	CC1=NC2=C(C=CC=C2)C(=C1)C(=O)N1CCNCC1	255.32	C15H17N3O	14'487.32	14'491	1.02
145	NC1CCCN(C1)C(=O)CC1=CC=CC=C1	218.30	C13H18N2O	14'475.40	14'479	0.92
146	NC1=CC2=C(C=C1)N(N=C2)C(F)F	183.16	C8H7F2N3	14'439.26	14'443	1.52
147	CN1C=C(CN2CCNCC2)C=N1	180.26	C9H16N4	14'371.26	14'375	-0.19
148	CN1C=C(C(N)=O)C(=N1)C1CCCNC1	208.27	C10H16N4O	14'448.37	14'452	-0.36
149	NC1CCC2=C(C1)N=C(N2)C1CCCCC1	219.33	C13H21N3	14'475.43	14'479	1.84
150	CN1C=CN=C1N1CCC(N)CC1	180.26	C9H16N4	14'412.26	14'416	0.08
151	O=C(N1CCNCC1)C1=C2C=CC=CC2=NN1	230.27	C12H14N4O	14'455.37	14'459	0.33
152	Cl.Cl.O=C(N1CCNCC1)C1=CC=NC=C1	264.15	C10H15Cl2N3O	14'398.15	14'402	-0.48
153	N[C@H]1CCO[C@@H]1C1=CN=CC=C1	164.21	C9H12N2O	14'405.31	14'409	-0.16
154	COC1=C2CCNCC2=CC=C1	163.22	C10H13NO	14'385.32	14'389	1.41
155	Cl.NC1(CC1)C1=CC(Cl)=C(Cl)C=C1	238.54	C9H10Cl3N	14'402.00	14'406	2.70
156	NC1CCN(CC2=CC=CC=C2)CC1CO	220.32	C13H20N2O	14'421.32	14'425	0.43

157	CN1CCN(CC1)C1=CC(N)=CC(C)=C1	205.31	C12H19N3	14'437.31	14'441	1.61
158	NC1CCN(CCC2=CC=NC=C2)CC1	205.31	C12H19N3	14427.41	14'431	0.20
159	FC1=CC2=C(C=C1)N=C(O2)C1CCCNC1	220.25	C12H13FN2O	14'445.35	14'449	1.85
160	C1CC(CN1)N1C=NC2=C1CCCC2	191.28	C11H17N3	14'481.38	14'485	0.75
161	CN(C(C)=O)C1=CC(N)=CC=C1	164.21	C9H12N2O	14'445.31	14'449	0.25
162	CC(C)CC1=NN=C2CNCCN12	180.26	C9H16N4	14'413.36	14'417	0.13
163	CC1=CN=CN1C1CCNCC1	165.24	C9H15N3	14'374.34	14'378	-0.08
164	FC1=C(N=CC=C1)N1CCCNCC1	195.24	C10H14FN3	14'452.34	14'456	1.12
165	NCC1=CC=CC=C1N1CCOCC1	192.26	C11H16N2O	14481.36	14'485	0.99
166	CC1=C(OC2CCNC2)C=CC=N1	178.24	C10H14N2O	14'433.34	14'437	0.31
167	NC1=CC(CCN2CCCC2)=CC=C1	190.29	C12H18N2	14'381.29	14'385	1.78
168	O=S(=O)(NC1=CC=CC=C1)C1CCCNC1	240.32	C11H16N2O2S	14'481.42	14'485	0.45
169	FC(F)C1=NC(=NO1)C1CCNCC1	203.19	C8H11F2N3O	14'419.19	14'423	0.81
170	CCN1CC2=C(C1)C=C(N)C=C2	162.24	C10H14N2	14'418.34	14'422	1.19
171	CC1=CN=C(S1)N1CCNCC1	183.27	C8H13N3S	14'383.27	14'387	1.53
172	CC1=NC(CC2CCCNC2)=NO1	181.24	C9H15N3O	14'422.34	14'426	0.79
173	CCN1C=CN=C1[C@H]1OCCC[C@@H]1N	195.27	C10H17N3O	14'402.27	14'406	0.26
174	NC1CN(CC(F)(F)F)C(=O)C1	182.15	C6H9F3N2O	14'438.25	14'442	-0.43
175	CC(=O)NC1=NN(C=C1)C1CCNC1	194.24	C9H14N4O	14'434.34	14'438	-0.44
176	CCC(N)(CC)C#C	111.19	C7H13N	14368.23	14'372	1.34
177	NC1CN(CC2=CC=CC=C2)CCC1CO	220.32	C13H20N2O	14'469.42	14'473	0.55
178	C(C1CCCNC1)N1C=NC=N1	166.23	C8H14N4	14'407.33	14'411	-0.14
179	NC1CCC(CC1)C1=NC=NO1	167.21	C8H13N3O	14'343.21	14'347	0.39
180	NC1CCC2=NN=C(N2C1)C1=CC=CC=C1	214.27	C12H14N4	14'494.27	14'498	0.76
181	NCC#C	55.08	C3H5N	14326.18	14'330	-0.40
182	FC(F)(F)C1=NN=C2CNCCN12	192.15	C6H7F3N4	14'392.15	14'396	-0.17
183	CC1=C(F)C=CC(=C1)C1CNCCO1	195.24	C11H14FNO	14'444.34	14'448	2.03
184	O=C(NC1=NC=CS1)C1CCCNC1	211.28	C9H13N3OS	14'451.38	14'455	-0.49
185	NC1CCN(CC1)C(=O)C1CCC1	182.27	C10H18N2O	14'431.37	14'435	-0.15
186	O=C1CCCCN1C1CCCNC1	182.27	C10H18N2O	14'439.37	14'443	0.19
187	CC1=C(SC=N1)C(=O)N1CCCNCC1	225.31	C10H15N3OS	14'496.31	14'500	-0.28
188	NC1CCN(C1)C(=O)C1=CC(F)=CC=C1	208.24	C11H13FN2O	14'473.34	14'477	0.57
189	CC1=CN=C(S1)C1CCNCC1	182.29	C9H14N2S	14'448.39	14'452	1.50
190	O=C1NC2=C(C=CC=C2)C11CCNC1	188.23	C11H12N2O	14'422.33	14'426	0.74
191	CN(C)C(=O)CC1CCNCC1	170.26	C9H18N2O	14'435.36	14'439	-0.29
192	C1CN(CCN1)C1=CC2=C(C=CC=C2)N=C1	213.28	C13H15N3	14'484.38	14'488	1.70
193	CN1C=CN=C1[C@H]1OCCC[C@@H]1N	181.24	C9H15N3O	14'415.34	14'419	-0.09
194	CNC(=O)C1=NN(C=C1)C1CCCNC1	208.27	C10H16N4O	14'449.37	14'453	-0.04
195	NC1CCC2=NN=C(CC3=CC=CC=C3)N2C1	228.30	C13H16N4	14'453.40	14'457	0.69
196	FC(F)(F)CC(=O)N1CCNCC1	196.17	C7H11F3N2O	14'396.17	14'400	-0.05
197	CC1=CC=CC(N)=C1	107.16	C7H9N	14333.16	14'337	1.66
198	CC1=NC=C(CN2CCNCC2)S1	197.30	C9H15N3S	14'398.30	14'402	0.29
199	N[C@H]1CCC[C@H]1OC1=C(F)C=CC=C1	195.24	C11H14FNO	14'469.34	14'473	2.13
200	C1CC2=C(CN1)C=NC(=N2)C1=CC=CC=C1	211.27	C13H13N3	14'451.37	14'455	2.03
201	NC1=CC(F)=C(C=C1)C1=NN=CO1	179.15	C8H6FN3O	14'395.15	14'399	0.29
202	CC(N)C1CN(CC2=CC=CC=C2)CCO1	220.32	C13H20N2O	14'485.42	14'489	1.38
203	CC1=C(C(C)=NN1)C1=C(N)C=CC=C1	187.25	C11H13N3	14'388.25	14'392	1.43
204	O=C1CCCCN1C1CCNCC1	182.27	C10H18N2O	14'431.37	14'435	-0.27
205	CC1=NNC(=C1)N1CCC(N)CC1	180.26	C9H16N4	14'411.36	14'415	-0.18
206	CC1=NN=C(O1)C1CC2=C(CN1)C=CC=C2	215.26	C12H13N3O	14'439.36	14'443	0.69
207	NC(=O)C1=CN(N=C1C1CCCNC1)C1=CC=CC=C1	270.34	C15H18N4O	14'502.34	14'506	1.30
208	O=C1CCCN1CC1CCNCC1	182.27	C10H18N2O	14'462.37	14'466	-0.25
209	CC1=NNC=C1C1CCNCC1	165.24	C9H15N3	14'422.34	14'426	0.51

210	NC1CN(C2CCCCC2)C(=O)C1	182.27	C10H18N2O	14'431.37	14'435	0.42
211	FC(F)(F)CN1CCCNCC1	182.19	C7H13F3N2	14'472.29	14'476	0.67
212	CC(=O)NC1=CNN=C1C1CCCNC1	208.27	C10H16N4O	14'424.27	14'428	-0.09
213	CC1=NN(CC2CNCCO2)C(C)=C1	195.27	C10H17N3O	14'387.27	14'391	0.21
214	COC(=O)[C@@H](N)CC1=CNC=N1	169.18	C7H11N3O2	14419.28	14'423	-0.87
215	CCN1CC2=C(C1)C(N)=CC=C2	162.24	C10H14N2	14403.34	14'407	1.19
216	CC1=CC(=CC=C1)C(=O)N1CCCNCC1	218.30	C13H18N2O	14'458.40	14'462	1.31
217	CN1C=CN=C1CC1CCCNC1	179.27	C10H17N3	14'475.37	14'479	0.57
218	C(C1CCNC1)N1CCOCC1	170.26	C9H18N2O	14'427.36	14'431	-0.33
219	CN(C)C(=O)C1=C(F)C=CC(N)=C1	182.20	C9H11FN2O	14'456.30	14'460	0.59
220	CC1=CN(CC2CCNCC2)N=C1	179.27	C10H17N3	14'346.27	14'350	1.02
221	FC1=CC=C(C=C1)N1CCCNCC1	194.25	C11H15FN2	14'474.35	14'478	1.75
222	COC(=O)CC[C@H](N)C(=O)OC	175.18	C7H13NO4	14390.28	14'394	-0.64
223	C(OC1CCNCC1)C1CCCCO1	199.29	C11H21NO2	14'479.39	14'483	0.63
224	NC1CCN(C1)C1=C(C=CC=C1)C(N)=O	205.26	C11H15N3O	14'486.36	14'490	0.08
225	C(C1CCOCC1)N1CCNCC1	184.28	C10H20N2O	14'490.38	14'494	0.08
226	N[C@@H]1CCCC[C@H]1OCC1=CC=CC=C1	205.30	C13H19NO	14'397.30	14'401	2.47
227	CS(=O)(=O)NC1=C2CCNCC2=CC=C1	226.29	C10H14N2O2S	14'458.29	14'462	-0.29
228	C(N1CCNCC1)C1=CSC=N1	183.27	C8H13N3S	14399.27	14'403	0.12
229	FC(F)(F)CC1=NN=C2CNCCN12	206.17	C7H9F3N4	14'511.27	14'515	-0.23
230	CN1C2CCC1CC(C2)N1CCNCC1	209.34	C12H23N3	14'434.44	14'438	0.21
231	CN(C)CCN1CCCNCC1	171.29	C9H21N3	14'411.39	14'415	-0.27
232	CC(C)(C)OC(=O)NN	132.16	C5H12N2O2	14372.26	14'376	0.35
233	N[C@H]1CCCO[C@@H]1C1=CC=NC=C1	178.24	C10H14N2O	14'354.24	14'358	0.36
234	CC1=NC=C(C=C1)C1CCNCC1	176.26	C11H16N2	14410.36	14'414	0.99
235	NC1CCN(CC1)C1CCOC1	170.26	C9H18N2O	14361.00	14'365	-0.70
236	C1CCNC1	71.12	C4H9N	14342.22	14'346	0.21
237	CC(C)N1C=C(C=N1)[C@@H]1CNC[C@H]1CO	209.29	C11H19N3O	14514.39	14'518	-0.12
238	NC1CCN(CC2=CNC3=C2C=CC=N3)CC1	230.32	C13H18N4	14422.30	14'426	0.37
239	NCC1=CC=C(C=C1)C#N	132.17	C8H8N2	14413.27	14'417	0.96
240	CN1CCNCC1C1=NC=CN1	166.23	C8H14N4	14'382.23	14'386	-0.45
241	C(OC1CCNC1)C1=NC=CS1	184.26	C8H12N2OS	14'424.36	14'428	0.16
242	C1CC(=CCN1)C1=CC2=C(N1)N=CC=C2	199.26	C12H13N3	14'455.36	14'459	1.14
243	N[C@H]1CC[C@@H](CC1)OC1=CC=C(F)C=C1	209.26	C12H16FNO	14'505.40	14'509	2.26
244	NC1=NN(C(=O)C1)C1=CC=CC=C1	175.19	C9H9N3O	14431.29	14'435	0.45
245	COC1=CC(N)=CC(OC)=C1OC	183.21	C9H13NO3	14'398.31	14'402	0.67
246	Cl.Cl.NCC1=NC=C(C=N1)C1=CC=CC=C1	258.15	C11H13Cl2N3	14457.20	14'461	1.30
247	COC(=O)[C@@H](N)C(C)C	131.18	C6H13NO2	14427.28	14'431	0.45
248	CC1=CN2CCCC(CN)C2=N1	165.24	C9H15N3	14'375.16	14'379	0.16
249	CN(C)CCN1CC2=C(C1)C(N)=CC=C2	205.31	C12H19N3	14414.41	14'418	0.86
250	NC1=CC=C(C=C1)S(N)(=O)=O	172.20	C6H8N2O2S	14'419.30	14'423	-0.25
251	NCCCN1CCCC1=O	142.20	C7H14N2O	14'398.30	14'402	-1.09
252	N[C@@H](CC(O)=O)C(O)=O	133.10	C4H7NO4	14310.10	14'314	-3.50
253	CC[C@H](C)[C@H](N)C(=O)OC	145.20	C7H15NO2	14410.30	14'414	0.90
254	NC1CCN(CC1)C(=O)C1CCC1	182.27	C10H18N2O	14349.27	14'353	-0.15
255	CC(C)(C)OC(=O)NCCN	160.22	C7H16N2O2	14'385.32	14'389	0.07
256	NCC1=CC=C(Br)C=C1F	204.04	C7H7BrFN	14'485.14	14'489	2.01
257	COC1=C(Br)C=C(CCN)C=C1	230.11	C9H12BrNO	14'487.21	14'491	2.00
258	NC1=C(F)C=C2N=COC2=C1	152.13	C7H5FN2O	14353.13	14'357	0.63
259	COC1=C(Br)C=C(CCN)C=C1	230.11	C9H12BrNO	14'461.21	14'465	2.00
260	NC1=CC=C(C=C1)C(=O)NC1CN2CCC1CC2	245.33	C14H19N3O	14470.43	14'474	0.59
261	NC(C1=CC=CC=C1)C1=CC=CC=C1	183.25	C13H13N	14'439.35	14'443	2.88
262	NOCC1=CC=CC=C1	123.16	C7H9NO	14'338.26	14'342	1.36
263	CN(C)CC1=C(N)C=C(C=C1)C(N)=O	193.25	C10H15N3O	14440.35	14'444	-0.06
264	NC1CCCSC1	117.21	C5H11NS	14'326.31	14'330	0.53

265	COC1=C(CN)C=CC=C1	137.18	C8H11NO	14'386.18	14'390	0.94
266	NCC(C1=CC=CC=C1)C1=CC=CC=C1	197.28	C14H15N	14'438.38	14'442	2.89
267	COC1=CC=C(CCN)C=C1	151.21	C9H13NO	14'425.31	14'429	1.23
268	CCC(C)CN	87.17	C5H13N	14'313.17	14'317	1.06
269	NC(CC1=CC=CC=C1)C1=CC=CC=C1	197.28	C14H15N	14'461.38	14'465	3.17
270	FC(F)(F)CN1CCNCC1=O	182.15	C6H9F3N2O	14391.25	14'395	-0.29
271	NC(C#N)C1CCOCC1	140.19	C7H12N2O	14'365.29	14'369	-0.46
272	COC1=CC=CC(CCN)=C1	151.21	C9H13NO	14'357.31	14'361	1.23
273	CC1=CC=CC(CN)=C1	121.18	C8H11N	14'371.28	14'375	1.61
274	COC1=CN=C(N)C=C1	124.14	C6H8N2O	14'404.24	14'408	0.36
275	NCC1=C(Cl)C=CC=C1	141.60	C7H8ClN	14'438.70	14'443	1.70
276	COC(=O)[C@@H](N)CC1=CC=CC=C1	179.22	C10H13NO2	14'419.32	14'423	1.22
277	COC(=O)[C@@H](N)CCSC	163.24	C6H13NO2S	14'437.34	14'441	0.22
278	COC1=CC=C(CN)C=C1	137.18	C8H11NO	14'338.18	14'342	0.94
279	COC(=O)[C@@H](N)CO	119.12	C4H9NO3	14'359.12	14'363	-1.48
280	COC(=O)[C@@H](N)[C@@H](C)O	133.15	C5H11NO3	14'375.15	14'379	-1.06
281	COC(=O)[C@@H](N)CC1=CNC2=C1C=CC=C2	218.26	C12H14N2O2	14'508.26	14'512	1.32
282	COC1=C(CN)C=CC=C1	137.18	C8H11NO	14'353.18	14'357	0.94
283	COC(=O)[C@H](C)N	103.12	C4H9NO2	14'328.22	14'332	-0.43
284	COC1=CC(CN)=CC=C1	137.18	C8H11NO	14'403.28	14'407	0.94
285	COC(=O)[C@@H](N)CC(C)C	145.20	C7H15NO2	14'409.30	14'413	0.82
286	COC(=O)[C@@H]1CCCN1	129.16	C6H11NO2	14'409.26	14'413	0.05
287	COC(=O)[C@@H](N)CC1=CN[C@@H]2NC(CC3=CNC=N3)C(=O)N12	306.33	C13H18N6O3	14'531.10	14'535	-2.04
288	COC(=O)CN	89.09	C3H7NO2	14'360.19	14'364	-1.00
289	COC(=O)CC[C@H](N)C(=O)OC	175.18	C7H13NO4	14'376.18	14'380	-0.64
290	C1CCC2=CN=C(C3CCCNC3)N2C1	205.31	C12H19N3	14'460.41	14'464	1.15
291	COC(=O)[C@@H](N)C(C)C	131.18	C6H13NO2	14'405.28	14'409	0.45
292	FC1=CC2=C(C=C1)N=C(O2)C1CCCNC1	220.25	C12H13FN2O	14'459.35	14'463	1.85
293	COC(=O)C1(N)CCC2=CC=CC=C2C1	205.26	C12H15NO2	14445.36	14'449	1.78
294	N[C@@H](CC1=CC=CC=C1)C(O)=O	165.19	C9H11NO2	14470.29	14'474	-1.18
295	NC1CSCC2=C1C=CC=C2	165.25	C9H11NS	14412.35	14'416	1.60
296	NC1CCN(CC2CCCC2)CC1	182.31	C11H22N2	14408.31	14'412	1.07
297	NC1=C(C=CC=C1)N1CCOCC1	178.24	C10H14N2O	14402.34	14'406	1.03
298	NC1CCCOC1	101.15	C5H11NO	14341.25	14'345	-0.21
299	C[C@H](N)C(O)=O	89.09	C3H7NO2	14304.19	14'308	-2.84
300	CC(C)C[C@H](N)C(O)=O	131.18	C6H13NO2	14371.28	14'375	-1.59

4.2.2. LC characterization of pool step 1

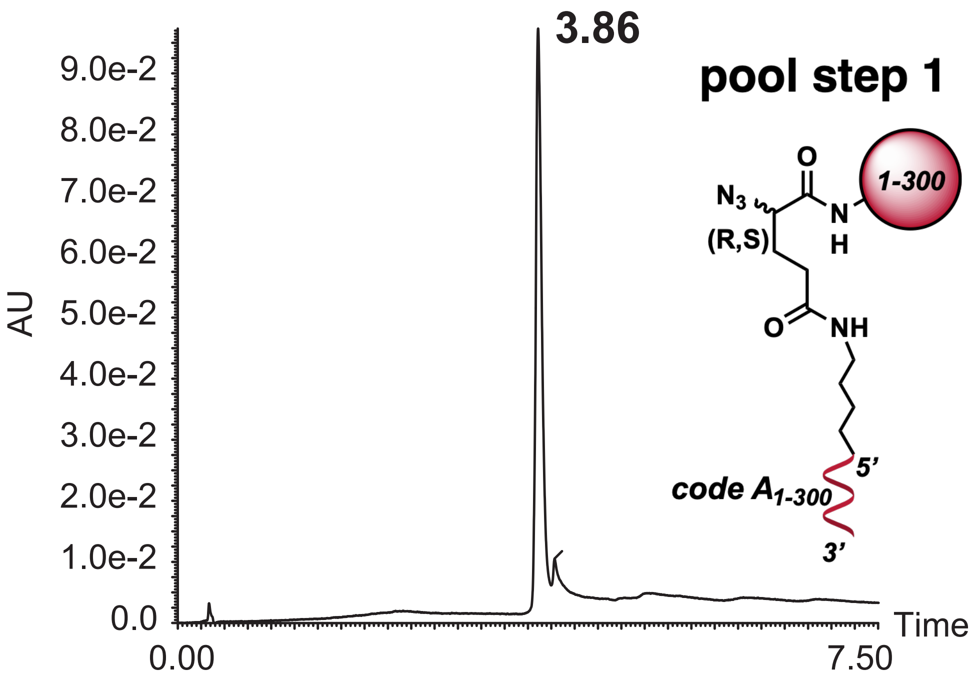

Supplementary figure 3: UPLC of the pooled three hundred oligo-conjugates formed in step 1.

4.3. Library step 2.

4.3.1. Staudinger reduction of library step 1 pool

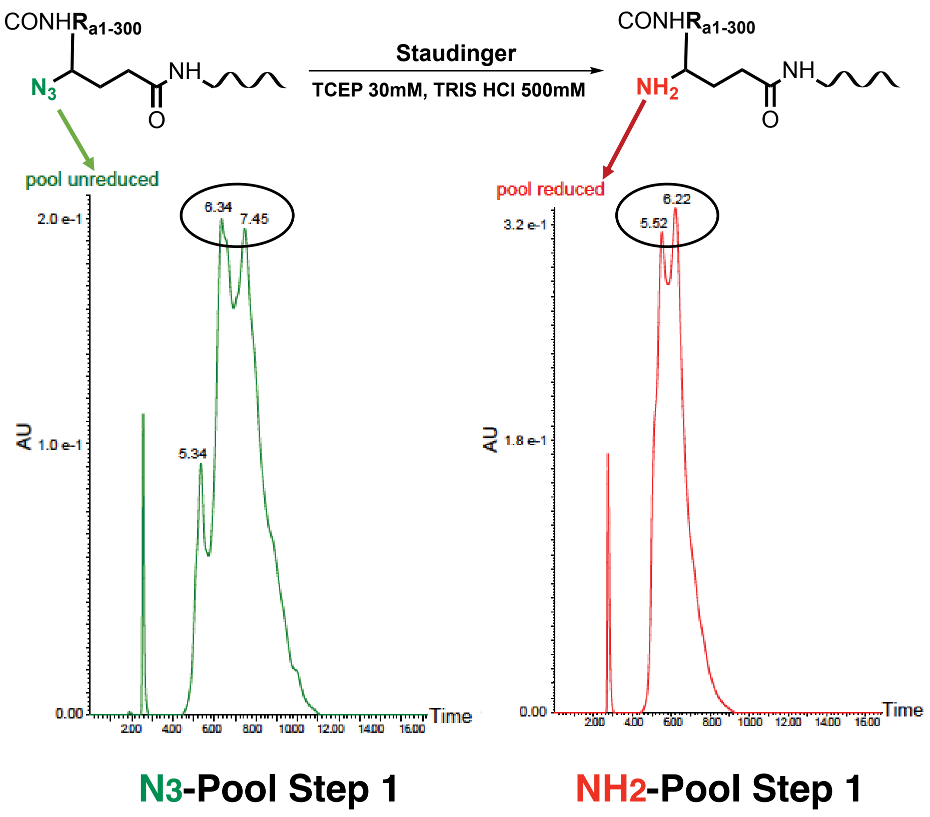

Supplementary figure 4: RP-HPLC profile of the **pool step 1** before Staudinger reduction (green) and after reduction (red). The retention time shift (circled in black in the two chromatograms), confirms reduction had occurred.

In a 2 mL Eppendorf tube **pool step 1** (200 nmol, 1 eq) was dissolved in 200 µL of TCEP (30 mM, in 500 mM TRIS*HCl pH= 7.4). The resulting mixture was stirred at 30 °C for 4 hours. The reacted DNA was precipitated by adding 50 uL (NaCl 5 M) and 800 uL EtOH absolute. The precipitated DNA was kept at -20°C for 4 hours and centrifuged at 4°C for 30 mins at 16.1 krpm. The supernatant was discarded and the pellet dried under vacuum.

4.3.2. Pool step 1 splint ligation with Code B oligonucleotides (611 reactions)

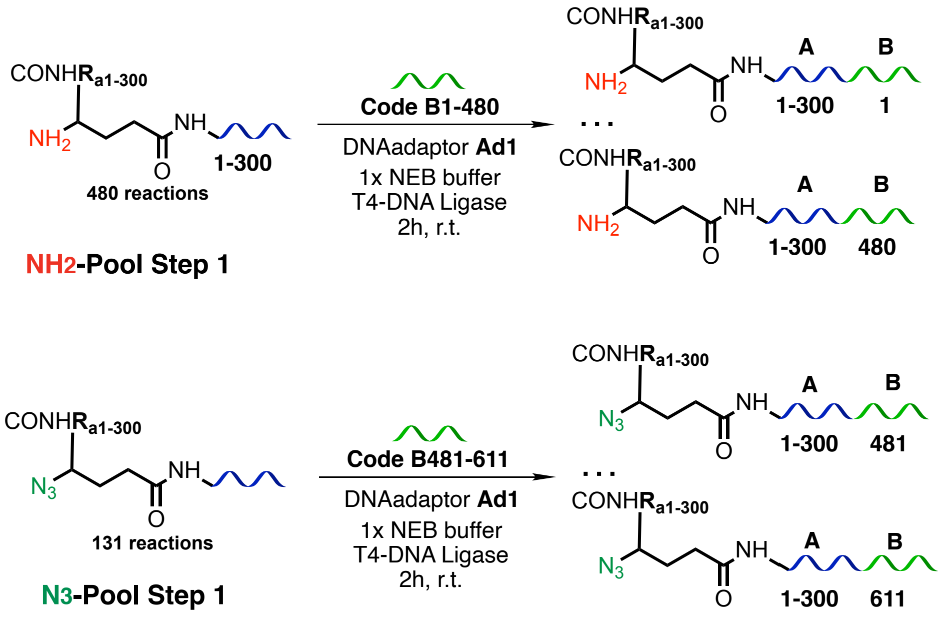

Supplementary figure 5: Strategy used for the splint ligation of 611 different **codeB** to the nascent library

To **NH_2-pool step 1** (0.3 nmol, 1 eq, wells 1-497) and **N_3-pool step 1** (0.3 nmol, wells 498-611) in pellet form were added 4.6 uL of 10x NEB buffer (500 mM Tris-HCl, 100 mM $MgCl_2$, 100 mM Dithiothreitol, 10 mM ATP, pH= 7.5), 2 uL chimeric DNA/RNA adaptor **Ad1**: 5'-CAGCACACAGGCTGCCTGACG-3' (0.75 nmol, 0.375 mM in H_2O, 2.5 eq) and 6 uL of corresponding **code B** 5'-CGCGGTACCCTGAGCXXXXXXGTCGTGTGTC-3' (0.6 nmol, 2 eq). The 611 resulting mixtures were heated at 75°C for 10 min and then cooled down to room temperature for 15 min. Finally, 1 uL of T-4 DNA ligase 400 U/mL was added to each well. The reaction mixtures were kept at room temperature for two hours. The reactions were then heated at 65°C for 10 minutes to denature the T4 DNA ligase. The ligated DNA products were dried under reduced pressure.

The purification conditions after splint ligation on the pool were optimized in a model reaction using **N_3-pool step 1** and **code B_1**. Results are shown in **supplementary figure 6 .**

a)

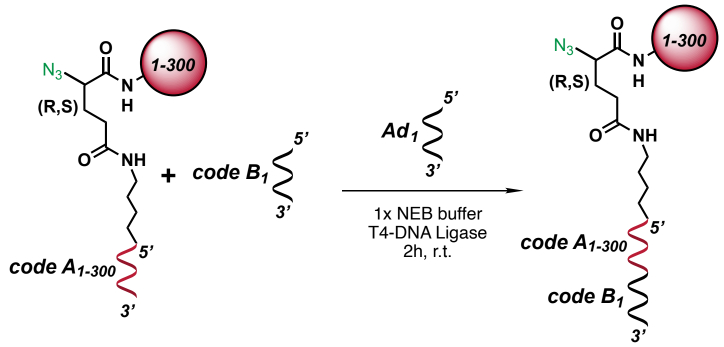

N3
(R,S)
1-300
code A1-300
5'
3'
+
code B1
Ad1
1x NEB buffer
T4-DNA Ligase
2h, r.t.

b)

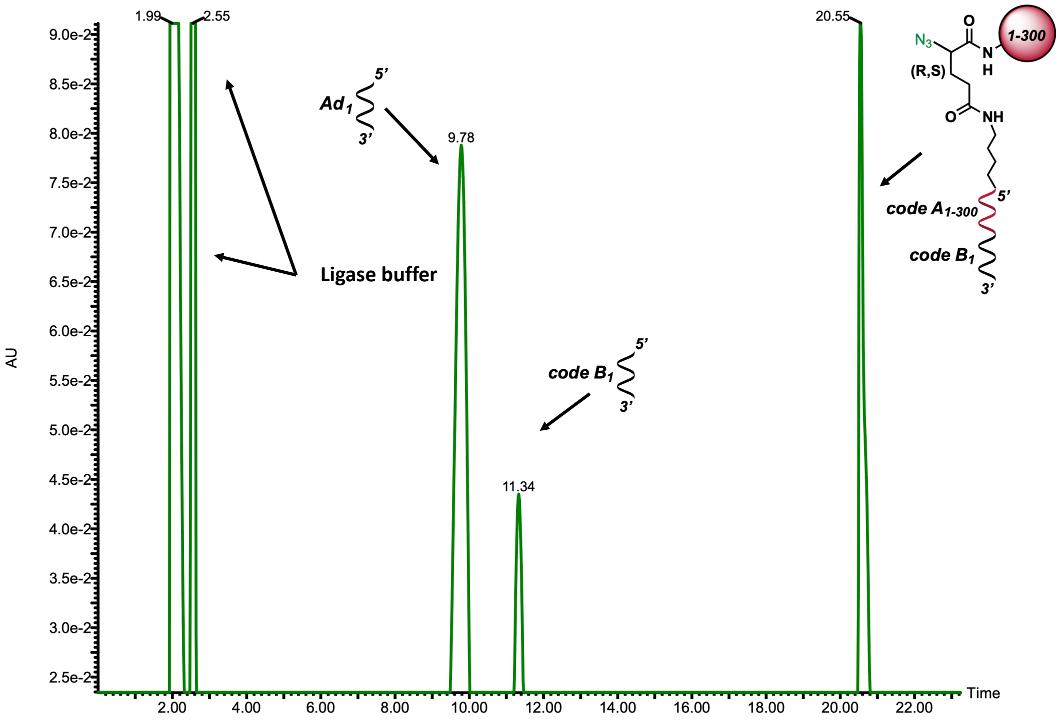

1.99
2.55
9.78
11.34
20.55
Ligase buffer
Ad1
code B1
code A1-300
1-300
AU
Time

c)

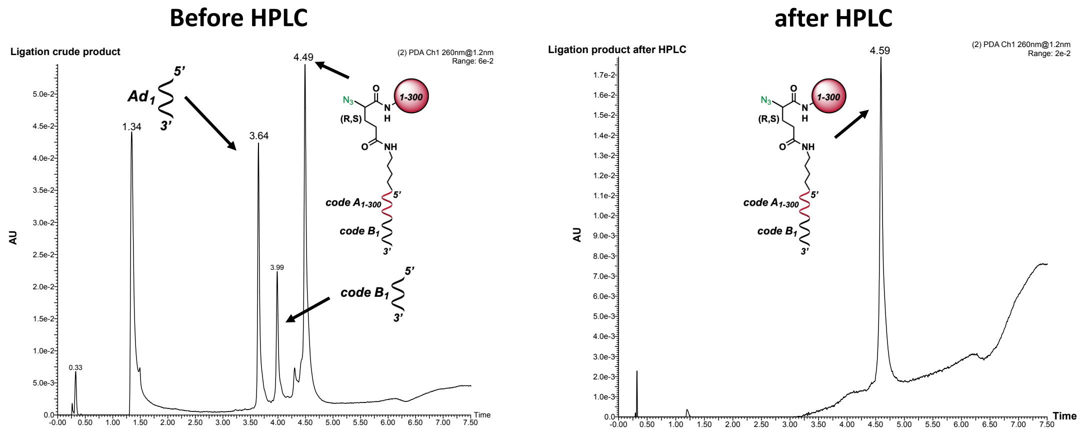

Before HPLC
after HPLC
Ligation crude product
Ligation product after HPLC
1.34
3.64
3.99
4.49
0.33
4.59
(2) PDA Ch1 260nm@1.2nm
Range: 6e-2
Range: 2e-2
Ad1
code B1
code A1-300
1-300
AU
Time

Supplementary figure 6: a) Model ligation on N_3**-pool step 1** and **Code B_1** used to test purification of the ligation product. **b)** RP-HPLC profile (graph in green) of the reaction crude registered at λ=260 nm. The ligation product was isolated from the unreacted **Code B_1** as confirmed by UPLC profile before and after RP-HPLC (**c**).

4.3.3. Test reactions with building blocks B.

All building blocks **B** used for library construction were individually tested and selected interrogating their reactivity toward the **Sc-NH_2** or **Sc-N_3** using model reactions on a chemically modified oligonucleotide (**std-1**:5'-C6-amino-GGAGCTTCTGAATT-3'). The model **oligo-Sc** conjugate was obtained via coupling of the scaffold to **std-1** using conditions **4.1.1**, methyl ester deprotection (**4.1.3**) and reverse amide bond formation with cyclohexylamine (**4.1.4**). The **std-1-scaffold** oligo-conjugate was used with the azide moiety for the test reactions with alkynes as described in **4.1.7** and after on-DNA Staudinger reduction for the model reactions with carboxylic acids (**4.1.2**). Results are reported in **Supplementary figure 7.**

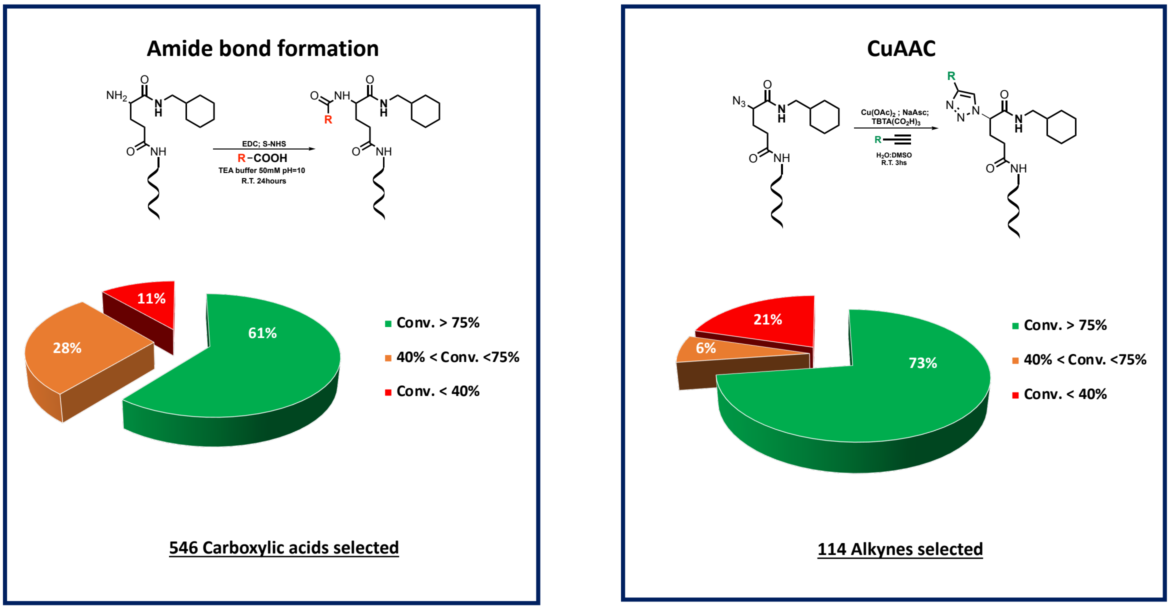

Supplementary figure 7: Left panel reports test reactions of the **std-1-scaffold** oligo conjugate with carboxylic acids. 546 Cas gave conversions greater than 75% and were suitable for library construction. Right panel shows the CuAAC model reaction of the **std-1-scaffold** oligo conjugate with Alkynes. 131 alkynes reacted with conversion rates above 75% and were thus fitting the cut-off imposed for library construction.

4.3.4. Encoded pool step 1 reactions with building blocks B (611 reactions)

The encoded oligonucleotides (**4.3.2**) were reacted as described in section **4.1.2** with 480 of the 546 carboxylic acids (well 1-480), wells 481-611 were reacted with 131 alkynes using general procedure **4.1.7.** A schematic representation of the process is depicted in **supplementary figure 8.**

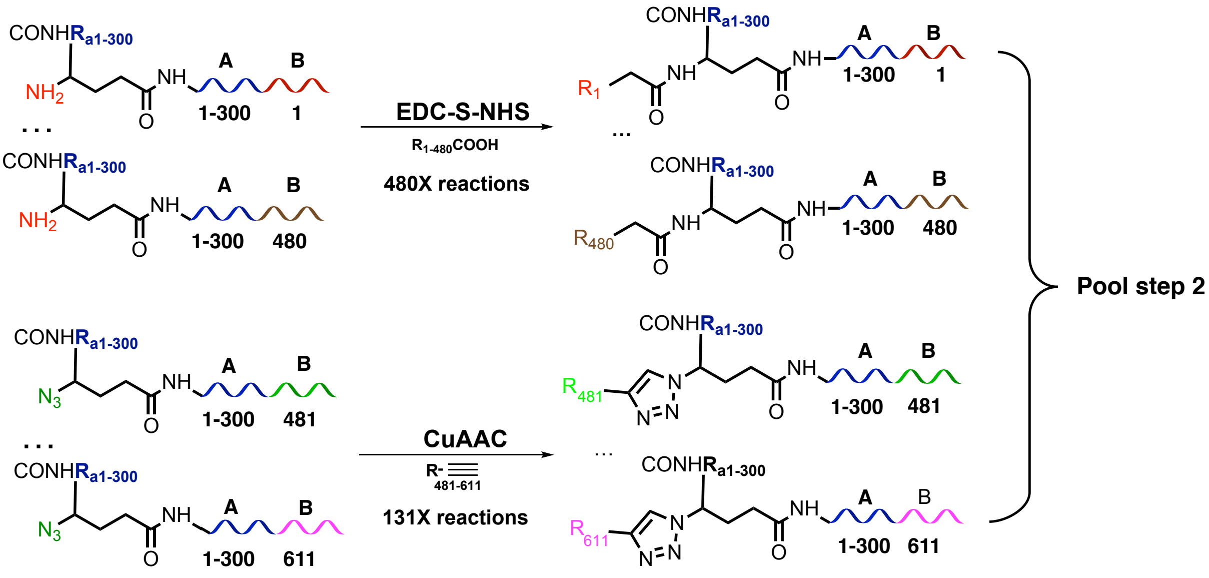

Supplementary figure 8: Reactions of 497 encoded **NH_2-Pool step 1** with carboxylic acids (top) and CuAAC click reaction of 114 encoded **N_3-Pool step 1** with the alkynes (bottom).

After reaction quenching, EtOH precipitation and centrifugation, the DNA pellets in the 611 wells were re-dissolved in mQ H_2O and pooled together prior to RP-HPLC purification. Details on the purification are described in section **4.3.5**. A detailed list of the Building blocks B used in the library construction can be found in **supplementary table 2.**

Supplementary table 2: List of Building blocks B used for library construction

CdId	Structure	Mol Weight	Formula	LogP
1	OC(=O)COC1=C2C=CC=CC2=CC=C1	202.21	C12H10O3	2.28
2	CC1=CC2=C(SCC(O)=O)N=CN=C2S1	240.30	C9H8N2O2S2	2.33
3	OC(=O)C1=CNC(=O)N1	128.09	C4H4N2O3	-0.74
4	CC(C)CC(O)=O	102.13	C5H10O2	1.21
5	CCC=CCC(O)=O	114.14	C6H10O2	1.45
6	CCC(C)CC(O)=O	116.16	C6H12O2	1.65
7	CC(C)CCC(O)=O	116.16	C6H12O2	1.65
8	OC(=O)CC1CCCC1	128.17	C7H12O2	1.63
9	OC(=O)CCC1CCCC1	142.20	C8H14O2	2.08

10	OC(=O)CC1CCCCC1	142.20	C8H14O2	2.08
11	CC(C)(C)CC(O)=O	116.16	C6H12O2	1.51
12	OC(=O)CC1=CC(F)=CC=C1F	172.13	C8H6F2O2	1.90
13	CC(CC(O)=O)C=C	114.14	C6H10O2	1.35
14	CC(=O)[C@@H]1C[C@@H](CC(O)=O)C1(C)C	184.24	C10H16O3	1.33
15	CC(=O)CCC(O)=O	116.12	C5H8O3	-0.07
16	CCCCC(O)=O	102.13	C5H10O2	1.37
17	OC(=O)CCC=C	100.12	C5H8O2	1.06
18	CN(C)CC(O)=O	103.12	C4H9NO2	-3.06
19	CCOCCC(O)=O	118.13	C5H10O3	0.20
20	CC(C)(C)OCCC(O)=O	146.19	C7H14O3	0.89
21	OC(=O)CNC(=O)C1=CC=CC=C1	179.18	C9H9NO3	0.53
22	OC(=O)C[C@@H]1C[C@H]2CC[C@@H]1C2	154.21	C9H14O2	1.74
23	CC1=CC=CC=C1CC(O)=O	150.18	C9H10O2	2.12
24	CC1=CC=C(CC(O)=O)C=C1	150.18	C9H10O2	2.12
25	CC1=CC(CC(O)=O)=CC=C1	150.18	C9H10O2	2.12
26	OC(=O)CCC1=CC=CC=C1	150.18	C9H10O2	2.06
27	OC(=O)CCCCC#C	126.16	C7H10O2	1.45
28	OC(=O)CCS(=O)(=O)C1=CC=CC=C1	214.24	C9H10O4S	0.68
29	CC1(C)[C@@H]2CC[C@@]1(C)[C@H](CC(O)=O)[C@H]2O	212.29	C12H20O3	1.40
30	CCC=CCC1C(CC(O)=O)CCC1=O	210.27	C12H18O3	2.41
31	OC(=O)CC=CC1=CC=CC=C1	162.19	C10H10O2	2.22
32	CC1=CC=C(SCC(O)=O)C=C1	182.24	C9H10O2S	2.28
33	O[C@H](CC(O)=O)C1=CC=CC=C1	166.18	C9H10O3	0.98
34	C[C@@H](NC(=O)CCC(O)=O)C1=CC=CC=C1	221.26	C12H15NO3	1.16
35	C[C@H](NC(=O)CCC(O)=O)C1=CC=CC=C1	221.26	C12H15NO3	1.16
36	COC1=CC=CC=C1CC(O)=O	166.18	C9H10O3	1.45
37	COC1=CC(CCC(O)=O)=C(OC)C=C1	210.23	C11H14O4	1.74
38	COC1=CC(OC)=C(CCC(O)=O)C=C1	210.23	C11H14O4	1.74
39	OC(=O)CCC1=CC=CO1	140.14	C7H8O3	0.96
40	COC1=CC=CC(CCC(O)=O)=C1OC	210.23	C11H14O4	1.74
41	CC1=C(OCC(O)=O)C=CC=C1	166.18	C9H10O3	1.81
42	CC1=CC=CC=C1C(=O)NCC(O)=O	193.20	C10H11NO3	1.04
43	CC1=CC(=CC=C1)C(=O)NCC(O)=O	193.20	C10H11NO3	1.04
44	CC1=CC=C(C=C1)C(=O)NCC(O)=O	193.20	C10H11NO3	1.04
45	CC(O)(CC(O)=O)C(F)(F)F	172.10	C5H7F3O3	0.49
46	OC(=O)CCNC(=O)C1=CC=CC=C1	193.20	C10H11NO3	0.76
47	COC1=CC=C(OC)C(CC(O)=O)=C1	196.20	C10H12O4	1.30
48	COC1=C(OC)C(CC(O)=O)=CC=C1	196.20	C10H12O4	1.30
49	OC(=O)CCC1=CC2=C(OCC2)C=C1	192.21	C11H12O3	1.94
50	COC1=CC=C(CC(O)=O)C(OC)=C1OC	226.23	C11H14O5	1.14
51	COC1=C(OCC(O)=O)C=CC=C1	182.18	C9H10O4	1.14
52	COC1=CC=C(OC)C(=C1)C(=O)CCC(O)=O	238.24	C12H14O5	1.04
53	OC(=O)CC1=CC=C(F)C=C1Cl	188.58	C8H6ClFO2	2.36
54	OC(=O)CCCC1=CC2=C(N1)C=CC=C2	203.24	C12H13NO2	2.36
55	OC(=O)CC1=C(F)C=C(F)C=C1F	190.12	C8H5F3O2	2.04
56	COC1=CC2=C(NC(CC(O)=O)=C2)C=C1	205.21	C11H11NO3	1.47
57	OC(=O)COC1=CC=CC=C1	152.15	C8H8O3	1.29

58	OC(=O)CC1=CC=C(F)C(F)=C1F	190.12	C8H5F3O2	2.04
59	OC(=O)CCNC(=O)C1=CC=C(C=C1)[N+]([O-])=O	238.20	C10H10N2O5	0.70
60	OC(=O)CNC(=O)OCC1=CC=CC=C1	209.20	C10H11NO4	1.02
61	COC1=CC(OC)=CC(CCC(O)=O)=C1	210.23	C11H14O4	1.74
62	OC(=O)CC1=CC=C(O)C(=C1)[N]([O-])=O	197.15	C8H7NO5	0.48
63	COC1=CC2=C(C=C1)C(=O)C(CC(O)=O)C2	220.22	C12H12O4	1.35
64	COC1=CC=C(Br)C=C1CC(O)=O	245.07	C9H9BrO3	2.22
65	OC(=O)CC1=CC=C(F)C(F)=C1	172.13	C8H6F2O2	1.90
66	OC(=O)CC1=CC=C(F)C(Cl)=C1	188.58	C8H6ClFO2	2.36
67	CC1=CC=C(F)C=C1CC(O)=O	168.17	C9H9FO2	2.27
68	OC(=O)CC1=C(F)C(F)=C(F)C(F)=C1F	226.10	C8H3F5O2	2.32
69	OC(=O)CC1NC(=O)C(CC2=CC=CC=C2)NC1=O	262.27	C13H14N2O4	-0.06
70	OC(=O)CC1=CC=C(Cl)N=C1	171.58	C7H6ClNO2	1.22
71	OC(=O)CC1=CC=CC=C1Cl	170.59	C8H7ClO2	2.22
72	OC(=O)CC1=CC=CC(=C1)C(F)(F)F	204.15	C9H7F3O2	2.49
73	OC(=O)CC1=CC=C(C=C1)C(F)(F)F	204.15	C9H7F3O2	2.49
74	OC(=O)CC1=CC=CC(F)=C1	154.14	C8H7FO2	1.75
75	OC(=O)CC1=CC(F)=CC(F)=C1	172.13	C8H6F2O2	1.90
76	OC(=O)CC1=CC=C(F)C=C1F	172.13	C8H6F2O2	1.90
77	COC1=C(OC)C=C(CC(O)=O)C(Br)=C1	275.10	C10H11BrO4	2.06
78	COC1=CC2=C(NC(CC(O)=O)=C2C)C=C1	219.24	C12H13NO3	1.99
79	OC(=O)CCNS(=O)(=O)C1=CC2=C(C=CC=C2)C=C1	279.31	C13H13NO4S	1.51
80	OC(=O)CC1=CC(=CC=C1F)[N+]([O-])=O	199.14	C8H6FNO4	1.69
81	C[C@@H](CC(O)=O)C1=CC=CC=C1	164.20	C10H12O2	2.34
82	OC(=O)CC1=CC=CC=C1F	154.14	C8H7FO2	1.75
83	OC(=O)CC1=CC=CC(F)=C1F	172.13	C8H6F2O2	1.90
84	CC(O)=O	60.05	C2H4O2	-0.22
85	OC(=O)CN1C(=O)C2=C(C=CC=C2)C1=O	205.17	C10H7NO4	0.40
86	OC(=O)CCCC1=CC=C2OCCOC2=C1	222.24	C12H14O4	2.01
87	COC1=CC=C2C(CC(O)=O)=COC2=C1	206.20	C11H10O4	1.61
88	OC(=O)CC1=CSC2=C1C=CC=C2	192.23	C10H8O2S	2.49
89	CC1=C(CC(O)=O)N=C(O1)C1=CC=CC=C1	217.22	C12H11NO3	2.16
90	OC(=O)CC1=COC2=C1C=CC=C2	176.17	C10H8O3	1.77
91	OC(=O)CCN1C(=O)C2=C(C=CC=C2)C1=O	219.20	C11H9NO4	0.63
92	COC1=CC=C(CC(O)=O)C=C1	166.18	C9H10O3	1.45
93	CCOC1=CC=C(CC(O)=O)C=C1	180.20	C10H12O3	1.81
94	COC1=CC(CC(O)=O)=CC=C1	166.18	C9H10O3	1.45
95	CCOC1=CC=CC=C1CC(O)=O	180.20	C10H12O3	1.81
96	OC(=O)COC1=C(C=CC=C1)[N+]([O-])=O	197.15	C8H7NO5	1.23
97	OC(=O)CCCC1=CC=C(C=C1)[N+]([O-])=O	209.20	C10H11NO4	2.44
98	CC1=C(CC(O)=O)NC2=C1C=CC=C2	189.21	C11H11NO2	2.14
99	CN1C=C(CC(O)=O)C2=C1C=CC=C2	189.21	C11H11NO2	1.93
100	COC1=CC=C(CC(O)=O)C=C1OC	196.20	C10H12O4	1.30
101	COC1=CC(CC(O)=O)=CC(OC)=C1OC	226.23	C11H14O5	1.14
102	CCOC1=CC=C(CCC(O)=O)C=C1	194.23	C11H14O3	2.25
103	OC(=O)CCC1=CC2=C(OCO2)C=C1	194.19	C10H10O4	1.68
104	OC(=O)CCC1=NC(=NO1)C1=C(F)C=CC=C1	236.20	C11H9FN2O3	2.30
105	COC1=C(CCC(O)=O)C=CC=C1	180.20	C10H12O3	1.90
106	COC1=CC=C(CCC(O)=O)C=C1	180.20	C10H12O3	1.90

107	COC1=CC=C(CC(O)=O)C=C1C	180.20	C10H12O3	1.97
108	CCOC1=CC=C(CC(O)=O)C=C1OC	210.23	C11H14O4	1.65
109	OC(=O)CCC1=CC(=O)C2=C(O1)C=CC(F)=C2	236.20	C12H9FO4	1.66
110	OC(=O)CCC1=CC(=O)C2=C(O1)C=CC(Cl)=C2	252.65	C12H9ClO4	2.12
111	OC(=O)CCC1=NC=C(O1)C1=CC=CC=C1	217.22	C12H11NO3	1.43
112	OC(=O)CCC1=NC=C(O1)C1=CC=C(Cl)C=C1	251.67	C12H10ClNO3	2.03
113	OC(=O)CCC1=NOC(=C1)C1=CC=C(Cl)C=C1	251.67	C12H10ClNO3	2.47
114	OC(=O)CNC(=O)C=CC1=CC=CO1	195.17	C9H9NO4	0.09
115	OC(=O)CC1=CC=CS1	142.17	C6H6O2S	1.52
116	OC(=O)CCC1=NC=C(O1)C1=CC=C(Br)C=C1	296.12	C12H10BrNO3	2.20
117	CC1=CC(OCC(O)=O)=C2C3=C(CCC3)C(=O)OC2=C1	274.27	C15H14O5	2.29
118	OC(=O)COC1=CC2=C(C=CC=C2)C=C1	202.21	C12H10O3	2.28
119	OC(=O)CCC1=CC=CS1	156.20	C7H8O2S	1.97
120	OC(=O)CC1=CSC=C1	142.17	C6H6O2S	1.39
121	OC(=O)CCCC1=CC=CS1	170.23	C8H10O2S	2.41
122	OC(=O)CCC(=O)C1=CC=CS1	184.21	C8H8O3S	1.27
123	COC1=CC=C(C=C1F)C(=O)CCC(O)=O	226.20	C11H11FO4	1.34
124	COC1=CC=C(C=C1)C(=O)CCC(O)=O	208.21	C11H12O4	1.20
125	COC1=CC(CC(O)=O)=CC=C1F	184.17	C9H9FO3	1.60
126	OCC1=CC=C(OCCC(O)=O)C=C1	196.20	C10H12O4	0.76
127	OC(=O)CCC1=CC=C(F)C=C1	168.17	C9H9FO2	2.20
128	OC(=O)COC1=CC=C(F)C=C1	170.14	C8H7FO3	1.44
129	OC(=O)CCC1=CC(F)=C(F)C=C1	186.16	C9H8F2O2	2.34
130	OC(=O)CCC(=O)C1=CC=C(F)C=C1	196.18	C10H9FO3	1.50
131	OC(=O)CCC(=O)C1=CC=C(Cl)C=C1	212.63	C10H9ClO3	1.96
132	CC1=C(OCC(O)=O)C=CC(Cl)=C1	200.62	C9H9ClO3	2.41
133	OC(=O)COC1=CC=C(Cl)C=C1	186.59	C8H7ClO3	1.90
134	OC(=O)CCN1C(=O)COC2=C1C=CC=C2	221.21	C11H11NO4	0.33
135	OC(=O)CCC#C	98.10	C5H6O2	0.56
136	OC(=O)CC1=CC=C(Cl)C=C1F	188.58	C8H6ClFO2	2.36
137	CCC1CCC(CC1)C(=O)NCC(O)=O	213.28	C11H19NO3	1.52
138	OC(=O)CC(C1=CC=CO1)C1=CC=CC=C1	216.24	C13H12O3	2.46
139	CC1=NC2=C(C=CC=C2)N1CCC(O)=O	204.23	C11H12N2O2	-0.09
140	CC1=NN=NN1C1=CC=C(CC(O)=O)C=C1	218.22	C10H10N4O2	0.77
141	CC1=NN=C(O1)SCC(O)=O	174.17	C5H6N2O3S	-0.44
142	CC1=NN(CCC(O)=O)C(C)=C1C	182.22	C9H14N2O2	0.52
143	CCC1=CC(=O)OC2=CC(C)=CC(OCC(O)=O)=C12	262.26	C14H14O5	2.36
144	CC1=CC(OCC(O)=O)=C2C(C)=C(C)C(=O)OC2=C1	262.26	C14H14O5	2.31
145	COC1=CC=CC=C1C1=NOC(CCCC(O)=O)=N1	262.27	C13H14N2O4	2.38
146	OC(=O)C1=CC2=CC=CC=C2N=C1	173.17	C10H7NO2	1.46
147	OC(=O)C1=NC2=C(O)C=CC=C2C=C1	189.17	C10H7NO3	-0.05
148	OC(=O)C1=NC2=CC=CC=C2C=C1	173.17	C10H7NO2	0.79
149	OC(=O)C1CC2CCC1C2	140.18	C8H12O2	1.56
150	OC(=O)C1=NC=CC2=CC=CC=C12	173.17	C10H7NO2	0.25
151	OC(=O)C1=CC=C2NC=CC2=C1	161.16	C9H7NO2	1.73
152	OC(=O)CC1=CNC2=CC=C(Br)C=C12	254.08	C10H8BrNO2	2.48
153	OC(=O)CN1C=C(C(=O)C2CC2)C2=CC=CC=C12	243.26	C14H13NO3	2.11
154	CN1N=C(C(O)=O)C2=CC=CC=C12	176.18	C9H8N2O2	1.46
155	OC(=O)C1=CC=C(CN2C=NC3=CC=CC=C23)C=C1	252.27	C15H12N2O2	1.82

156	CC(CC1=CC=C2OCOC2=C1)C(O)=O	208.21	C11H12O4	2.22
157	OC(=O)CCCC1=CC2=C(OCCO2)C=C1	222.24	C12H14O4	2.01
158	CC1=CN=C(C=N1)C(O)=O	138.13	C6H6N2O2	-0.29
159	OC(=O)C1=NC2=C(C=CC=C2)N=C1	174.16	C9H6N2O2	1.34
160	OC(=O)C1=CC2=C(C=C1)N=CC=N2	174.16	C9H6N2O2	0.96
161	CSC1=NC(C(O)=O)=C(Cl)C=N1	204.63	C6H5ClN2O2S	0.31
162	OC(=O)C1=CN=C(N=C1)C1=CC=CS1	206.22	C9H6N2O2S	1.61
163	COC1=CC(C(O)=O)=C(OC)N=N1	184.15	C7H8N2O4	0.31
164	OC(=O)CCNC(=O)NC12CC3CC(CC(C3)C1)C2	266.34	C14H22N2O3	0.89
165	OC(=O)C1=CC2=C(O1)C=CC=C2	162.14	C9H6O3	1.71
166	COC1=CC=CC2=C1OC(=C2)C(O)=O	192.17	C10H8O4	1.55
167	OC(=O)C1=CC2=C(OC=C2)C=C1	162.14	C9H6O3	1.79
168	OC(=O)C1=CN2C=CSC2=N1	168.17	C6H4N2O2S	0.47
169	CSC1=NC=C(N1CC1=CC=CC=C1)C(O)=O	248.30	C12H12N2O2S	2.13
170	OC(=O)C1=CC=C(C=C1)N1C=CN=C1	188.19	C10H8N2O2	0.68
171	C[C@H]1CC[C@@H](CC1)C(O)=O	142.20	C8H14O2	2.18
172	OC(=O)C1CCC(=O)CC1	142.15	C7H10O3	0.71
173	COC1=NC2=CC=CC=C2N=C1CCC(O)=O	232.24	C12H12N2O3	1.69
174	OC(=O)CC1CCN(CC2=CC=CC=C2)CC1	233.31	C14H19NO2	-0.55
175	OC(=O)C1CCN(CC1)C(=O)C1=CC=C(F)C=C1	251.26	C13H14FNO3	1.49
176	COC1=CC=CC(OC)=C1C(=O)N1CCC(CC1)C(O)=O	293.32	C15H19NO5	1.03
177	COC1=CC(=CC(OC)=C1)C(=O)N1CCC(CC1)C(O)=O	293.32	C15H19NO5	1.03
178	COC1=CC(Cl)=CC2=C1OC(=C2)C(O)=O	226.61	C10H7ClO4	2.16
179	CC1=CC=C2NC(=O)C(CC(O)=O)C2=C1	205.21	C11H11NO3	1.25
180	CC1=CC(=O)N(CC(O)=O)C2=CC=CC=C12	217.22	C12H11NO3	1.24
181	OC(=O)CCN1C(=O)CSC1=S	205.25	C6H7NO3S2	0.55
182	OC(=O)C1=CC=C(CN2C=NN=N2)C=C1	204.19	C9H8N4O2	0.73
183	OC(=O)C1=CN2CCC3=C2C(=CC=C3)C1=O	215.21	C12H9NO3	1.41
184	CC(C)C1=CC(=NO1)C(O)=O	155.15	C7H9NO3	1.47
185	CN1N=CC(=C1C)C1=CC(=NO1)C(O)=O	207.19	C9H9N3O3	0.48
186	CN(CCC(O)=O)C1CCS(=O)(=O)C1	221.27	C8H15NO4S	-4.00
187	OC(=O)CCN1CCCCCC1=O	185.22	C9H15NO3	0.25
188	CC1=NN(CC(O)=O)C(=O)C2=CC=CC=C12	218.21	C11H10N2O3	0.54
189	CC1=CC=C(C=C1)N(CC(O)=O)S(C)(=O)=O	243.28	C10H13NO4S	0.56
190	OC(=O)C1C2CC(C=C2)C1C(=O)NC1CC1	221.26	C12H15NO3	0.30
191	OC(=O)C1=CN=C(N=C1)C1=CC=CN=C1	201.19	C10H7N3O2	0.16
192	CC1=CC=C(C=C1N1CCNC1=O)C(O)=O	220.23	C11H12N2O3	0.96
193	[H][C@]12CC[C@]([H])(C1)C(C2C(O)=O)C(=O)NC(C)C	225.29	C12H19NO3	0.97
194	OC(=O)[C@@H]1C[C@H]1C1=CC=CC=C1	162.19	C10H10O2	1.98
195	OC(=O)C1COC2=CC=CC=C2O1	180.16	C9H8O4	1.18
196	OC(=O)C1=NN(C(=O)C1)C1=CC=CC=C1	204.19	C10H8N2O3	1.49
197	O[C@H]1CC[C@@H](CC1)C(O)=O	144.17	C7H12O3	0.50
198	OCC(C(O)=O)C1=CC=CC=C1	166.18	C9H10O3	0.87
199	CC1(C)C(C(O)=O)C1(C)C	142.20	C8H14O2	1.73
200	CC(=O)N1C[C@H](O)C[C@H]1C(O)=O	173.17	C7H11NO4	-1.63
201	CCN1C=C(C(O)=O)C(=O)C2=C1N=C(C)C=C2	232.24	C12H12N2O3	0.79
202	CS(=O)(=O)C1=CC(F)=CC2=C1NC(=C2)C(O)=O	257.24	C10H8FNO4S	0.63
203	COC1=CC=C2OC(=CC2=C1)C(O)=O	192.17	C10H8O4	1.55

204	OC(=O)C1=CC2=C(O1)C=CC(Cl)=C2	196.59	C9H5ClO3	2.31
205	OC(=O)C1=CC=C2N=CSC2=C1	179.19	C8H5NO2S	1.66
206	CC(C)CN1CC(CC1=O)C(O)=O	185.22	C9H15NO3	0.27
207	CC(C)(O)C#CC1=CC=C(O1)C(O)=O	194.19	C10H10O4	1.01
208	COC1=CC=CC(=C1)N1C=C(C=N1)C(O)=O	218.21	C11H10N2O3	1.56
209	OC(=O)C1CN(C2CCCC2)C(=O)C1	197.23	C10H15NO3	0.38
210	CCCN1CCC(CC(O)=O)CC1	185.27	C10H19NO2	-1.40
211	OC(=O)CCCN1CCCCC1=O	185.22	C9H15NO3	0.09
212	CC(C)CN1CC(CCC1=O)C(O)=O	199.25	C10H17NO3	0.71
213	CCC(N1C=CC=N1)C(O)=O	154.17	C7H10N2O2	0.78
214	CC(C)C1=CC(=NC2=NC=NN12)C(O)=O	206.21	C9H10N4O2	1.34
215	CC(C)CC(=O)N1CCC(CC1)C(O)=O	213.28	C11H19NO3	0.92
216	OC(=O)C1CCN(CC1)C(=O)C1CCCC1	225.29	C12H19NO3	1.16
217	CCC(=O)N1CCCC(C1)C(O)=O	185.22	C9H15NO3	0.35
218	OC(=O)C1=NOC(=C1)C1=CC=CC=C1	189.17	C10H7NO3	1.95
219	OC(=O)C1=CC=C(CN2CCCCC2)C=C1	219.28	C13H17NO2	-0.31
220	CC(N(C1=CC=CC=C1)S(C)(=O)=O)C(O)=O	243.28	C10H13NO4S	0.62
221	COC1=CC=C(C=C1)C1=CC(=NN1)C(O)=O	218.21	C11H10N2O3	1.73
222	OC(=O)C1CCN(CC1)C(=O)C1=CC=C(C=C1)[N+]([O-])=O	278.26	C13H14N2O5	1.28
223	CC(CC(O)=O)N1N=C(C)C(C(C)=O)=C1C	224.26	C11H16N2O3	0.06
224	CN(C)C(=O)N1CCC(CC1)C(O)=O	200.24	C9H16N2O3	-0.40
225	CC1=C(C)N2C(S1)=NC=C(C(O)=O)C2=O	224.23	C9H8N2O3S	0.96
226	OC(=O)C1CCCC1	114.14	C6H10O2	1.45
227	OC(=O)C1=CC=CO1	112.08	C5H4O3	0.69
228	OC(=O)C1=COC=C1	112.08	C5H4O3	0.77
229	CC1=CC=C(S1)C(O)=O	142.17	C6H6O2S	2.19
230	CC1=NC(C)=C(S1)C(O)=O	157.19	C6H7NO2S	0.54
231	CN1N=C(C=C1C(O)=O)C(C)(C)C	182.22	C9H14N2O2	1.76
232	OC(=O)C1=CN=CC(O)=C1	139.11	C6H5NO3	-0.59
233	CC1=NC=C(C=C1)C(O)=O	137.14	C7H7NO2	-1.00
234	CSC1=C(C=CC=N1)C(O)=O	169.20	C7H7NO2S	1.47
235	OC(=O)C1=CC=CN=C1SCCS(=O)(=O)C1=CC=CC=C1	323.38	C14H13NO4S2	2.02
236	CC(C)OC1=CC=C(C=C1)C(O)=O	180.20	C10H12O3	2.25
237	CC(=O)NC1=CC=C(C=C1NC(C)=O)C(O)=O	236.23	C11H12N2O4	0.11
238	COC1=CC(=CC(OC)=C1)C(O)=O	182.18	C9H10O4	1.32
239	COC1=CC(=CC(OC)=C1C)C(O)=O	196.20	C10H12O4	1.83
240	CN(C)C1=CC=CC(=C1)C(O)=O	165.19	C9H11NO2	1.35
241	COC1=CC(=CC(OC)=C1OC)C(O)=O	212.20	C10H12O5	1.16
242	OC(=O)C1=CC(F)=C(F)C=C1F	176.09	C7H3F3O2	2.06
243	OC(=O)C1=C(F)C=C(Cl)C=C1	174.56	C7H4ClFO2	2.38
244	OC(=O)C1=CC(Cl)=C(F)C=C1	174.56	C7H4ClFO2	2.38
245	OC(=O)C1=C(F)C(F)=CC=C1	158.10	C7H4F2O2	1.92
246	OC(=O)C1=CC=C(F)C(F)=C1	158.10	C7H4F2O2	1.92
247	OC(=O)C1=CC=C(Cl)C=C1	156.57	C7H5ClO2	2.23
248	OC(=O)C1=CC=CC(Cl)=C1	156.57	C7H5ClO2	2.23
249	OC(=O)C1=C(Cl)C=CC=C1	156.57	C7H5ClO2	2.23
250	OC(=O)C1=C(Cl)C=CC(=C1)[N+]([O-])=O	201.56	C7H4ClNO4	2.17
251	OC(=O)C1=CC(Cl)=CC=C1[N+]([O-])=O	201.56	C7H4ClNO4	2.17

252	COC(=O)C1=CC(=CC=C1)C(O)=O	180.16	C9H8O4	1.63
253	CCOC(=O)C1=CC(=CC(=C1)C(O)=O)C(=O)OCC	266.25	C13H14O6	2.35
254	NC(=O)COC1=CC=CC(=C1)C(O)=O	195.17	C9H9NO4	0.14
255	OC(=O)C1=NC=CN=C1	124.10	C5H4N2O2	-0.42
256	OC(=O)C1=NC=CN1	112.09	C4H4N2O2	-1.41
257	CC1=C(CC(O)=O)N=C(O1)C1=CC=CC=C1	217.22	C12H11NO3	2.16
258	CCC1=CC(=O)OC2=CC(OCC(O)=O)=C(Cl)C=C12	282.68	C13H11ClO5	2.45
259	OC(=O)C1=CC(CN2C=CC=N2)=CC=C1	202.21	C11H10N2O2	1.70
260	OC(=O)C1=CC(=CC=C1)N1C=CC=N1	188.19	C10H8N2O2	1.72
261	CN(CCC(O)=O)C1CCN(C)C1	186.26	C9H18N2O2	-2.86
262	CC1=NNC(=C1)C(O)=O	126.12	C5H6N2O2	-0.26
263	OC(=O)C1=CC(=C(C=C1)N1CCCC1)[N+]([O-])=O	236.23	C11H12N2O4	2.08
264	OC(=O)C1=CC=CS1	128.15	C5H4O2S	1.54
265	CCN1N=C(C)C=C1C(O)=O	154.17	C7H10N2O2	0.23
266	OC(=O)C1=CC=NN1	112.09	C4H4N2O2	-0.14
267	OC(=O)C1=CC=CC(O)=N1	139.11	C6H5NO3	-0.58
268	COC1=C(OC)C(OC)=C(C=C1)C(O)=O	212.20	C10H12O5	1.16
269	CN1C=NC2=C(C(C)=C(S2)C(O)=O)C1=O	224.23	C9H8N2O3S	1.20
270	OC(=O)C1C2OC3(CN(CC4=NC=CC=C4)C(=O)C13)C=C2	286.29	C15H14N2O4	-1.04
271	CC(=O)NC1=CC(=CC(NC(C)=O)=C1)C(O)=O	236.23	C11H12N2O4	0.11
272	CC(=O)OC12CC3CC(C1)CC(C3)(C2)C(O)=O	238.28	C13H18O4	1.51
273	OC(=O)CC1=NN=C(N1)C1=CN=CC=C1	204.19	C9H8N4O2	-0.98
274	OC(=O)CN1N=C2C=CC=CC2=N1	177.16	C8H7N3O2	0.96
275	CCN1CC2=C(C1=O)C(=CC=C2)C(O)=O	205.21	C11H11NO3	1.04
276	CN1C(=O)C(=NC2=C1C=CC=C2)C(O)=O	204.19	C10H8N2O3	1.05
277	OC(=O)C1CCC=CC1	126.16	C7H10O2	1.53
278	CN(CC(O)=O)C1=NC=NC2=C1NC=N2	207.19	C8H9N5O2	-0.73
279	CC1=NC2=NC=NN2C(C)=C1CCC(O)=O	220.23	C10H12N4O2	0.63
280	OC(=O)C1=C(OC=N1)C1=CC=CO1	179.13	C8H5NO4	0.59
281	OC(=O)C1=CC=NO1	113.07	C4H3NO3	-0.08
282	CN1N=C(C)C(CCC(O)=O)=C1C	182.22	C9H14N2O2	0.48
283	CN1C=C(C=N1)C1=NOC(=C1)C(O)=O	193.16	C8H7N3O3	0.20
284	CS(=O)(=O)C1=CC=CC(=C1)C(O)=O	200.21	C8H8O4S	0.47
285	OC(=O)C1=CC(=CC=C1)C#C	146.15	C9H6O2	1.78
286	CS(=O)(=O)C1=CC=C(C=C1)C(O)=O	200.21	C8H8O4S	0.47
287	OC(=O)C1=CC=CC=C1	122.12	C7H6O2	1.63
288	CC1(CC1)C(O)=O	100.12	C5H8O2	1.11
289	OC(=O)C1CN(CC2=CC=CC=C2)C1	191.23	C11H13NO2	-1.31
290	CSC1=CC=C(CC(O)=O)C=C1	182.24	C9H10O2S	2.24
291	O[C@@H](C(O)=O)C1=CC=CC=C1	152.15	C8H8O3	0.90
292	NC(=O)C1=CC=C(C=C1)C(O)=O	165.15	C8H7NO3	0.48
293	CC1=CC=C(C=C1)C(O)=O	136.15	C8H8O2	2.14
294	CC1=CC=CC(=C1)C(O)=O	136.15	C8H8O2	2.14
295	OC(=O)C1=CC=CN=C1	123.11	C6H5NO2	-0.17
296	COC1=CC=CC(=C1)C(O)=O	152.15	C8H8O3	1.47
297	OC(=O)C1CCCCC1	128.17	C7H12O2	1.89
298	CC(C)C(O)C(O)=O	118.13	C5H10O3	0.42
299	O=C1CC2(CCCC2)CC(=O)O1	168.19	C9H12O3	1.19

300	O=C1OC(=O)C2CCCCC12	154.17	C8H10O3	1.25
301	CC1(C)CC(=O)OC(=O)C1	142.15	C7H10O3	0.77
302	OC(=O)CN1C=NC2=C(C=CC=C2)C1=O	204.19	C10H8N2O3	-0.49
303	OC(=O)C1=CC(Br)=CC=C1	201.02	C7H5BrO2	2.40
304	OC(=O)CN1C=CN=C1C1=CC=CC=C1	202.21	C11H10N2O2	0.34
305	CC(C)(O)C(O)=O	104.11	C4H8O3	-0.04
306	OC(=O)C1=CC=C(Br)O1	190.98	C5H3BrO3	1.16
307	OC(=O)CC1=CC(=CC=C1)[N+]([O-])=O	181.15	C8H7NO4	1.55
308	OC(=O)CSC1=CC=NC=C1	169.20	C7H7NO2S	-0.55
309	CC(=O)N[C@H](CC1=CNC2=C1C=CC=C2)C(O)=O	246.27	C13H14N2O3	1.00
310	CC(=O)N[C@@H](CC1=CNC2=CC=CC=C12)C(O)=O	246.27	C13H14N2O3	1.00
311	CC(=O)N[C@H](CC1=CC=CC=C1)C(O)=O	207.23	C11H13NO3	0.90
312	CC(=O)N[C@@H](CC1=CC=CC=C1)C(O)=O	207.23	C11H13NO3	0.90
313	CC(=O)N[C@H](CC1=CC=C(O)C=C1)C(O)=O	223.23	C11H13NO4	0.59
314	CC(=O)NC(CC1=CC=C(O)C=C1)C(O)=O	223.23	C11H13NO4	0.59
315	CC(=O)N[C@@H](CC(N)=O)C(O)=O	174.16	C6H10N2O4	-2.21
316	CC(=O)N[C@@H](CCC(N)=O)C(O)=O	188.18	C7H12N2O4	-1.92
317	CC(=O)N[C@@H](CC1=CNC=N1)C(O)=O	197.19	C8H11N3O3	-2.47
318	OC(=O)C1=CC=C(C=C1)C#N	147.13	C8H5NO2	1.49
319	O=C1OC(=O)C=C1	98.06	C4H2O3	0.56
320	OC(=O)C1CCC1	100.12	C5H8O2	1.00
321	CN1CCC(CC1)C(O)=O	143.19	C7H13NO2	-2.47
322	CC(O)CC(O)=O	104.11	C4H8O3	-0.39
323	CC1=NC(=CS1)C(O)=O	143.16	C5H5NO2S	0.80
324	OC(=O)C1=CSC(=N1)C(F)(F)F	197.13	C5H2F3NO2S	1.93
325	OC(=O)C1CC2=C(C1)C=CC=C2	162.19	C10H10O2	2.20
326	OC(=O)C1=CC(=C(Cl)C=C1)S(=O)(=O)N1CCOCC1	305.73	C11H12ClNO5S	1.07
327	CC1CN(CC(C)O1)S(=O)(=O)C1=CC=C(C=C1)C(O)=O	299.34	C13H17NO5S	1.30
328	OC(=O)C1=C(Cl)C=CC(=C1)S(=O)(=O)N1CCCCC1	303.76	C12H14ClNO4S	2.14
329	COC1=C(C=CC=C1)N(C)S(=O)(=O)C1=CC=C(C=C1)C(O)=O	321.35	C15H15NO5S	2.18
330	CC1(C)CC2=C(O1)C(OCC(O)=O)=CC=C2	222.24	C12H14O4	1.87
331	CC(C)CC(NC(N)=O)C(O)=O	174.20	C7H14N2O3	0.16
332	CCC(C)C(NC(N)=O)C(O)=O	174.20	C7H14N2O3	0.24
333	NC(=O)N1CCCC1C(O)=O	158.16	C6H10N2O3	-0.82
334	CC1=CC=C(CN2CC(CC2=O)C(O)=O)C=C1	233.27	C13H15NO3	1.26
335	CC1=C(SC2=C1C(=O)NC=N2)C(O)=O	210.21	C8H6N2O3S	0.97
336	OC(=O)CN1C=NC=N1	127.10	C4H5N3O2	-1.26
337	CC1=CC=C(C=C1)S(=O)(=O)N(CC(O)=O)CC(O)=O	287.29	C11H13NO6S	0.50
338	OC(=O)C1CN(CCC2=CC=CC=C2)C(=O)C1	233.27	C13H15NO3	1.04
339	OC1CC(N(C1)S(=O)(=O)C1=CC(Cl)=C(Cl)C=C1)C(O)=O	340.17	C11H11Cl2NO5S	1.18
340	CC(C)NS(=O)(=O)C1=CC(=CC=C1)C(O)=O	243.28	C10H13NO4S	1.23
341	CC(=O)NC1=NC(C(O)=O)=C(Br)C=C1	259.06	C8H7BrN2O3	-0.63
342	OC(=O)C1=C(ON=C1)C1=CC=CC=C1	189.17	C10H7NO3	1.56
343	CC1(CC(=O)OC1=O)C1=CSC=C1	196.22	C9H8O3S	1.75
344	CC1=NC(C(O)=O)=C(C)O1	141.13	C6H7NO3	0.28
345	OC(=O)C1=CC(=CN=C1)C1=CC=C(Cl)C=C1	233.65	C12H8ClNO2	2.27
346	CC1=CC(=CC=C1)C1=CC(=CN=C1)C(O)=O	213.24	C13H11NO2	2.18
347	NC1=C(Br)C=C(C=N1)C(O)=O	217.02	C6H5BrN2O2	-0.29

348	OC(=O)C1=CC=C(C=C1)S(=O)(=O)NCC=C	241.26	C10H11NO4S	1.19
349	OC(=O)C1=C(ON=C1)C1CC1	153.14	C7H7NO3	0.62
350	CN1C=C(C(O)=O)C(=N1)C(F)F	176.12	C6H6F2N2O2	0.60
351	O=C1CC2(CCCC2)C(=O)O1	154.17	C8H10O3	1.26
352	OC(=O)C1CN(C2CC2)C(=O)C1	169.18	C8H11NO3	-0.51
353	OC(=O)C1=C2N=CC(Br)=CN2N=C1	242.03	C7H4BrN3O2	1.08
354	CN1N=NC2=C1N=CC(=C2)C(O)=O	178.15	C7H6N4O2	0.23
355	OC(=O)C1=CC=C(C=C1)S(=O)(=O)NC1CC1	241.26	C10H11NO4S	0.93
356	OC(=O)C1CCN(CC1)C(=O)C1=CC=CS1	239.29	C11H13NO3S	1.26
357	OC(=O)C1=C(Br)C=CC(=C1)S(=O)(=O)NC1CC1	320.16	C10H10BrNO4S	1.69
358	COC1=C(C=C(C=C1)C(O)=O)S(=O)(=O)NC1CC1	271.29	C11H13NO5S	0.77
359	NC(=O)NC1=CC(=CC=C1)C(O)=O	180.16	C8H8N2O3	0.53
360	OC(=O)C1=CC2=C(N=CC=N2)N=C1	175.15	C8H5N3O2	0.06
361	CC1=C(Br)C=C(C(O)=O)C(O)=N1	232.03	C7H6BrNO3	2.25
362	CC1=CC(=CC=C1)C1=NN=C(S)N1CCC(O)=O	263.32	C12H13N3O2S	2.15
363	OC(=O)C1=C(N=CC=N1)C1=NC2=C(S1)C=CC=C2	257.27	C12H7N3O2S	2.13
364	CN1N=C2C=CC=CC2=C1C(O)=O	176.18	C9H8N2O2	1.35
365	OC(=O)C1CCN(CC1)S(=O)(=O)C1=CC2=C(CCC2)C=C1	309.38	C15H19NO4S	2.11
366	OC(=O)C1=CC2=C(N1)C=CC=C2F	179.15	C9H6FNO2	1.79
367	OC(=O)C1=NN(C(=O)NC1=O)C1=CC=CC=C1	233.18	C10H7N3O4	0.98
368	OC(=O)CC(CC(O)=O)C1=C(Br)C=CC=C1	287.11	C11H11BrO4	2.24
369	OC(=O)C1=CC=C(CN2C(=O)CNC2=O)C=C1	234.21	C11H10N2O4	0.16
370	CC1=C(C=C(C=C1)S(=O)(=O)NC1CC1)C(O)=O	255.29	C11H13NO4S	1.44
371	NC1=C(N=CC=N1)C(O)=O	139.11	C5H5N3O2	0.00
372	OC(=O)C1=C(Cl)C=CC(O)=C1	172.56	C7H5ClO3	1.93
373	CC1=NC2=C(C=C(O)C=C2)C(=O)N1CC(O)=O	234.21	C11H10N2O4	-0.74
374	CN1C=C(C)C2=C1CC(=O)OC2=O	179.18	C9H9NO3	1.14
375	CC1(CC(=O)OC1=O)C1=CC=C(F)C=C1	208.19	C11H9FO3	2.11
376	NC1=C2C=CN(CC(O)=O)C2=CC=C1	190.20	C10H10N2O2	0.66
377	NC1=C(NC(=O)NC1=O)C(O)=O	171.11	C5H5N3O4	-2.11
378	O=C1CC2=C(C=CO2)C(=O)O1	152.11	C7H4O4	0.46
379	CN1C2=C(N(CC(O)=O)C=N2)C(=O)N(C)C1=O	238.20	C9H10N4O4	-1.07
380	OC(=O)[C@@H]1CC(=O)NC(=O)N1	158.11	C5H6N2O4	-1.52
381	CC1=C(C(=O)C(O)=O)C2=C(N1)C=CC=C2	203.20	C11H9NO3	1.79
382	CC1=NOC2=C1C(=CC(=N2)C1CC1)C(O)=O	218.21	C11H10N2O3	1.21
383	CCC1(CC)CC(=O)OC1=O	156.18	C8H12O3	1.72
384	OC(=O)C1(CCCC1)NC(=O)NCC1=CC=CC=C1	262.31	C14H18N2O3	1.87
385	CCC=CCC(O)=O	114.14	C6H10O2	1.45
386	OCC(O)=O	76.05	C2H4O3	-1.04
387	CC1=CC=C(C=C1)S(=O)(=O)NCC(O)=O	229.25	C9H11NO4S	0.79
388	CN(CC(O)=O)C(=O)C1=CC=CC=C1	193.20	C10H11NO3	0.75
389	CN(C)C1=CC=C(CC(O)=O)C=C1	179.22	C10H13NO2	1.02
390	COC1=C(OC)C=C(CCC(O)=O)C=C1	210.23	C11H14O4	1.74
391	COC1=CC(CC(O)=O)=CC(Br)=C1O	261.07	C9H9BrO4	1.92
392	OC(=O)CC1=CC=C2OCOC2=C1	180.16	C9H8O4	1.23
393	COC1=CC(CCC(O)=O)=CC(OC)=C1OC	240.26	C12H16O5	1.58
394	OC(=O)CCC1=CNC2=C1C=CC=C2	189.21	C11H11NO2	2.15
395	COC1=CC(OC)=NC(CCC(O)=O)=N1	212.21	C9H12N2O4	0.59

396	OC(=O)CCC1=CC=CN=C1	151.17	C8H9NO2	-0.07
397	OC(=O)CCC1CNC2=C1C=CC=C2	191.23	C11H13NO2	0.83
398	OC(=O)CC1NC(=O)NC1=O	158.11	C5H6N2O4	-1.52
399	OC(=O)CCN1C(=O)OC2=C1C=CC=C2	207.19	C10H9NO4	0.91
400	CC1=CN(CC(O)=O)C(=O)NC1=O	184.15	C7H8N2O4	-0.76
401	CN1C2N=CN(CC(O)=O)C2C(=O)N(C)C1=O	240.22	C9H12N4O4	-2.94
402	CN(CC(O)=O)S(=O)(=O)C1=CC=CC=C1	229.25	C9H11NO4S	0.50
403	OC(=O)COC1=CC2=C(C=CC(=O)O2)C=C1	220.18	C11H8O5	1.10
404	CC1=C(C)C2=C(OC1=O)C=C(OCC(O)=O)C=C2	248.23	C13H12O5	1.80
405	OC(=O)CC1OC2=C(NC1=O)C=CC=C2	207.19	C10H9NO4	0.68
406	OC(=O)CC1=CC2=C(N1)C=CC=C2	175.19	C10H9NO2	1.63
407	COC1=C(CO)C=CC(OCC(O)=O)=C1	212.20	C10H12O5	0.37
408	CC1=CC=C(C=C1)C(=O)CCC(O)=O	192.21	C11H12O3	1.87
409	OC(=O)CCC1=CC(=O)C2=C(O1)C=CC(Br)=C2	297.10	C12H9BrO4	2.29
410	OC(=O)CCC1=NN=C(O1)C1=CC=CC=C1	218.21	C11H10N2O3	0.93
411	OC(=O)CNC(=O)C1=CC=CO1	169.14	C7H7NO4	-0.41
412	OC(=O)CCN1C(=O)COC2=C1C=C(Cl)C=C2	255.65	C11H10ClNO4	0.94
413	OC(=O)CCN1C=NC2=C(C=CC=C2)C1=O	218.21	C11H10N2O3	-0.27
414	OC(=O)CCC1=NC(=NO1)C1=CN=CC=C1	219.20	C10H9N3O3	0.41
415	OC(=O)CCN1C=CC(=O)NC1=O	184.15	C7H8N2O4	-0.92
416	CC(=O)C1=C(C)N(CCC(O)=O)N=C1C	210.23	C10H14N2O3	-0.41
417	CC1=CC2=C(C=CC=C2)N1CCC(O)=O	203.24	C12H13NO2	2.21
418	CC1=CC2=C(C=C1)C(CC(O)=O)C(=O)N2	205.21	C11H11NO3	1.25
419	CC1=CC(=O)OC2=C1C=C(OCC(O)=O)C=C2	234.21	C12H10O5	1.40
420	OC(=O)CCC1=NC(=NO1)C1=CC=CO1	208.17	C9H8N2O4	1.21
421	OC(=O)C1=C2C=CN=CC2=CC=C1	173.17	C10H7NO2	0.35
422	OC(=O)C1=CN=C2C=CC=CN2C1=O	190.16	C9H6N2O3	0.13
423	OC(=O)C1=CC=C2C=CNC2=C1	161.16	C9H7NO2	1.73
424	OC(=O)C1=CC2=CC(O)=CC=C2N1	177.16	C9H7NO3	1.35
425	COC1=CC=C2NC(=CC2=C1)C(O)=O	191.19	C10H9NO3	1.49
426	OC(=O)C1=NNC2=C1C=CC=C2	162.15	C8H6N2O2	1.34
427	OC(=O)C1=CC=C2NC=NC2=C1	162.15	C8H6N2O2	-0.24
428	CC1=NC(C)=C(CC(O)=O)C(O)=N1	182.18	C8H10N2O3	0.49
429	OC(=O)C1=CN=C(N=C1)N1CCOCC1	209.21	C9H11N3O3	0.13
430	OC(=O)C1=NNC(=C1)C1CC1	152.15	C7H8N2O2	0.94
431	COC1=CC2=C(C=C1)C(CC(O)=O)=CO2	206.20	C11H10O4	1.61
432	CC1=C(C=C(O1)S(=O)(=O)N1CCOCC1)C(O)=O	275.28	C10H13NO6S	-0.11
433	CC1=NC2=CC=C(C=C2N=C1C)C(O)=O	202.21	C11H10N2O2	1.22
434	CCCC(=O)C1=CN(CC(O)=O)C2=CC=CC=C12	245.28	C14H15NO3	2.48
435	OCCN1C=NC2=CC(=CC=C12)C(O)=O	206.20	C10H10N2O3	-0.68
436	CCN1N=C(C=C1C(O)=O)C(C)C	182.22	C9H14N2O2	1.54
437	OCCC1=CN2N=C(C=C2N=C1)C(O)=O	207.19	C9H9N3O3	0.22
438	OC(=O)C1=CC=C(CN2C=CC=N2)O1	192.17	C9H8N2O3	0.48
439	CC1(C)NC(=O)N(CC(O)=O)C1=O	186.17	C7H10N2O4	-0.75
440	OC(=O)C1CCN(CC2=CC=CO2)CC1	209.25	C11H15NO3	-1.67
441	NC(=O)CN1CCCC(C1)C(O)=O	186.21	C8H14N2O3	-3.62
442	C[C@H]1CCC[C@@H](C)N1CCC(O)=O	185.27	C10H19NO2	-1.14
443	CC1=CC=CN2C(CC(O)=O)=CN=C12	190.20	C10H10N2O2	-0.43
444	C[C@@H]1C[C@H](NC(=S)N1)C(O)=O	174.22	C6H10N2O2S	-0.03

445	OC(=O)[C@@H]1CCC(=O)N1	129.12	C5H7NO3	-0.89
446	OC(=O)C1CN(CC2=CN=CC=C2)C(=O)C1	220.23	C11H12N2O3	-1.38
447	COC1=C2OCC(CC2=CC=C1)C(O)=O	208.21	C11H12O4	1.52
448	CCCN1N=CC(C(O)=O)=C1C	168.20	C8H12N2O2	0.96
449	CC(=O)C1=C(C)N(CC(O)=O)N=C1C	196.21	C9H12N2O3	-0.75
450	OC(=O)C1=CC=CC=C1N1CCC(=O)NC1=O	234.21	C11H10N2O4	0.33
451	COC1=C(Cl)C=C(C=C1)N1CC(CC1=O)C(O)=O	269.68	C12H12ClNO4	1.13
452	CC1CCN(CC1)C1=C(C=C(C=C1)C(O)=O)[N+]([O-])=O	264.28	C13H16N2O4	2.82
453	OC(=O)CCCC1=NC(=NO1)C1=CC=NC=C1	233.23	C11H11N3O3	1.15
454	CN(C)S(=O)(=O)C1=CC(C(O)=O)=C(C)O1	233.24	C8H11NO5S	0.11
455	CN1C=CC=C1C(O)=O	125.13	C6H7NO2	0.85
456	CN1C(=CC2=C1C=CO2)C(O)=O	165.15	C8H7NO3	1.04
457	OC(=O)C1=CC=C(N=C1)C(F)(F)F	191.11	C7H4F3NO2	1.68
458	CC1=CC=CN=C1C(O)=O	137.14	C7H7NO2	-0.27
459	CCOC1=C(C=CC=N1)C(O)=O	167.16	C8H9NO3	1.21
460	OC(=O)C1=CN=C(O)C=C1	139.11	C6H5NO3	0.70
461	OC(=O)C1=CC=C(Br)C=N1	202.01	C6H4BrNO2	1.57
462	OC(=O)C1=CC=C(OC2=CC=C3OCOC3=C2)N=C1	259.22	C13H9NO5	2.13
463	OC(=O)C1=NNC(=O)C=C1	140.10	C5H4N2O3	-0.34
464	CC1=NC2=CC=CC=C2N1CC(O)=O	190.20	C10H10N2O2	-0.44
465	OC(=O)C1=CN=CN=C1	124.10	C5H4N2O2	-0.29
466	OC(=O)C1=CNN=C1	112.09	C4H4N2O2	-0.06
467	CC1=CC=C(C(O)=O)C(O)=N1	153.14	C7H7NO3	1.08
468	CC1=CC=C(O1)C1=NNC(=C1)C(O)=O	192.17	C9H8N2O3	1.15
469	OC(=O)C1=C(N=CC=N1)C(=O)N1CCCCC1	235.24	C11H13N3O3	0.12
470	CC1=CC=CC2=NC(=O)CC(C)(N12)C(O)=O	220.23	C11H12N2O3	-0.07
471	OC(=O)C1=CN=C2SC=CN2C1=O	196.18	C7H4N2O3S	0.47
472	OC(=O)C1=CC2=C(NC(=O)C(=O)N2)C=C1	206.16	C9H6N2O4	0.29
473	NC(=O)C1(CC1)C(O)=O	129.12	C5H7NO3	-0.50
474	CC1=NN2C(=C1)N=CC(C(O)=O)=C2C	191.19	C9H9N3O2	0.43
475	CC1=C(C(O)=O)C(C)=NO1	141.13	C6H7NO3	0.33
476	OC(=O)C1=CC=C2N=CNC(=O)C2=C1	190.16	C9H6N2O3	-0.16
477	OC(=O)C1=CC=C(OC2=CC=CN=C2)O1	205.17	C10H7NO4	-0.04
478	O[C@H](C(O)=O)C1=CC=CC=C1	152.15	C8H8O3	0.90
479	CNC(=O)C1=CC=C(C=C1)C(O)=O	179.18	C9H9NO3	0.71
480	CC(=O)N1CCC(CC1)C(O)=O	171.20	C8H13NO3	-0.51
481	C#CCN1C=CC2=C1C=CC=C2	155.20	C11H9N	2.52
482	Cl.NCCC(O)CCC#C	163.65	C7H14ClNO	-0.23
483	CCOC(=O)C1=C(NCC#C)N=C(Cl)C=C1	238.67	C11H11ClN2O2	2.89
484	Cl.NC1=C(C=CC=C1)C(=O)NCC#C	210.66	C10H11ClN2O	1.10
485	O=C(NCC#C)NC1=CC=CC=C1	174.20	C10H10N2O	1.33
486	CC1=CC(NC(=O)NCC#C)=NO1	179.18	C8H9N3O2	0.49
487	CN(CC#C)C1=NC=C(N)N=C1	162.20	C8H10N4	0.23
488	OC(=O)C1=C(NCC#C)N=C(Cl)C=C1	210.62	C9H7ClN2O2	2.18
489	Cl.C#CCNCC1=CC=CO1	171.62	C8H10ClNO	0.82
490	BrC1=C(OCC#C)C=CC=N1	212.05	C8H6BrNO	1.80
491	CC(O)CC#C	84.12	C5H8O	0.41
492	OC(=O)C1=CC(=CN=C1)C#C	147.13	C8H5NO2	0.36
493	COC1=C(Br)C=C(C=C1)C#C	211.06	C9H7BrO	2.74

494	BrC1=CC2=C(OCC(=O)N2CC#C)C=C1	266.09	C11H8BrNO2	1.62
495	OC1=CC(OCC#C)=CC=C1	148.16	C9H8O2	1.74
496	Cl.NC(CC#C)CC(F)(F)F	187.59	C6H9ClF3N	0.96
497	OC(=O)[C@@H]1CCCN1CC#C	153.18	C8H11NO2	-1.52
498	O=C1NCCN1CC#C	124.14	C6H8N2O	-0.64
499	NC(=O)C1=CC(=CN=C1)C#C	146.15	C8H6N2O	-0.24
500	C#CCN1C2=C(C=CC=C2)C2=C1C=CC=C2	205.26	C15H11N	3.54
501	COC1=C(N)C=C(C=C1)C#C	147.18	C9H9NO	1.14
502	BrC1=NC=C(OCC#C)C=C1	212.05	C8H6BrNO	1.80
503	COC1=C(C=C(Cl)C=C1)C#C	166.60	C9H7ClO	2.57
504	NC(=O)NC1=CC(=CC=C1)C#C	160.18	C9H8N2O	1.03
505	C#CCN1C=CN=C1	106.13	C6H6N2	0.31
506	COC1=C(F)C=C(C=C1)C#C	150.15	C9H7FO	2.11
507	FC(F)OC1=C(C=CC=C1)C#C	168.14	C9H6F2O	2.89
508	CCN1C=C(C=N1)C#C	120.16	C7H8N2	0.91
509	C#CCN1C=CN=C1C1=CC=CS1	188.25	C10H8N2S	2.11
510	C#CC1=NC2=C(C=CC=C2)N=C1	154.17	C10H6N2	1.84
511	O=C(NCC#C)NC1CC1	138.17	C7H10N2O	-0.22
512	OC(=O)C1=CC=C(C=C1)S(=O)(=O)NCC#C	239.25	C10H9NO4S	0.69
513	O=C(NCC#C)C1=CNC(=O)C=C1	176.18	C9H8N2O2	-0.90
514	COC1=CC(C#C)=C(Cl)C=C1	166.60	C9H7ClO	2.57
515	FC1=CC(=CC(F)=C1)C#C	138.12	C8H4F2	2.41
516	OC(=O)C1(CC#C)CCC1	138.17	C8H10O2	1.64
517	CC1=CC(NC(=O)CNCC#C)=CC=C1	202.26	C12H14N2O	1.46
518	OC(=O)CC(=O)NCC#C	141.13	C6H7NO3	-0.69
519	CC1=NC(=CS1)C#C	123.17	C6H5NS	1.29
520	Cl.NC(C#C)C1CCOCC1	175.66	C8H14ClNO	0.08
521	Cl.NC1(CCC1)C#C	131.60	C6H10ClN	0.44
522	CCC(C)(O)C#C	98.15	C6H10O	0.93
523	FC(F)(F)C1=NC(OCC#C)=CC=C1	201.15	C9H6F3NO	2.68
524	CC1=NC(=CC=C1)C#C	117.15	C8H7N	1.42
525	Cl.NC(=N)NCC#C	133.58	C4H8ClN3	-0.73
526	C#CCNC1CCCC1	123.20	C8H13N	1.39
527	C#CC1=CN=CN=C1	104.11	C6H4N2	0.20
528	C#CCN1C=NC=N1	107.12	C5H5N3	-0.06
529	ClC1=C(C=CC=C1)C#C	136.58	C8H5Cl	2.73
530	O=C1CCCCN1CC#C	137.18	C8H11NO	0.32
531	C#CC1=CC=NN1	92.10	C5H4N2	0.35
532	C#CC1=CC(=CC=C1)C1=CC=CC=C1	178.23	C14H10	3.77
533	O=C1NC(CC#C)C(=O)N1	138.13	C6H6N2O2	-0.72
534	O=S(=O)(NCC#C)C1=CC=CC=C1	195.24	C9H9NO2S	1.03
535	Cl.C#CCN1C=NC2=C1C=CC=C2	192.65	C10H9ClN2	1.71
536	COC1=NC(=CC=C1)C#C	133.15	C8H7NO	1.73
537	CC1=C(OCC#C)C=CC=C1	146.19	C10H10O	2.56
538	BrC1=CC2=C(OCCC2NCC#C)C=C1	266.14	C12H12BrNO	2.32
539	CC(NCC#C)C1=CC2=C(OCC(=O)N2)C=C1	230.27	C13H14N2O2	0.96
540	NC(C#C)C1CCCCC1	137.23	C9H15N	1.78
541	CC(C)(NS(C)(=O)=O)C#C	161.22	C6H11NO2S	-0.39
542	C#CC1=NC=CS1	109.15	C5H3NS	1.16

543	Cl.NC(CC#C)C(N)=O	148.59	C5H9ClN2O	-1.23
544	COC1=CC(=NC=C1)C#C	133.15	C8H7NO	1.13
545	CC1=NC2=C(C=C1)C=C(C=C2)C#C	167.21	C12H9N	2.41
546	CNC(=O)C#C	83.09	C4H5NO	-0.40
547	CC(O)(C#C)C1=C(Br)C=CC=C1	225.09	C10H9BrO	2.54
548	FC1=C(C=CC(Br)=C1)C(=O)C#C	227.03	C9H4BrFO	2.85
549	Cl.C#CCNCC1CC1	145.63	C7H12ClN	0.82
550	C#CC1=CN=CS1	109.15	C5H3NS	0.92
551	CN1CCN(CCC#C)CC1	152.24	C9H16N2	0.55
552	OC(CC#C)C1CC1	110.16	C7H10O	0.84
553	Cl.C#CC1CNC1	117.58	C5H8ClN	-0.19
554	OC(CC1=CC=CC=C1)C#C	146.19	C10H10O	1.78
555	COCCN(C)CC#C	127.19	C7H13NO	0.37
556	C#CCN1CCCC1	109.17	C7H11N	0.82
557	ClC1=CC(=CC(Cl)=C1)C#C	171.02	C8H4Cl2	3.33
558	CN1C=C(C=N1)C#C	106.13	C6H6N2	0.55
559	COCC(N)C#C	99.13	C5H9NO	-0.38
560	CN(C)CCC#C	97.16	C6H11N	0.71
561	ClC1=C(Cl)C(=CC=C1)C#C	171.02	C8H4Cl2	3.33
562	C#CCCNC1COC1	125.17	C7H11NO	0.17
563	FC1=C(C=CN=C1)C#C	121.11	C7H4FN	1.05
564	CS(=O)(=O)NCCC#C	147.19	C5H9NO2S	-0.79
565	NC(CO)CC#C	99.13	C5H9NO	-0.74
566	NC1=C(F)C(F)=C(C#C)C(F)=C1F	189.11	C8H3F4N	1.87
567	C#CC1=NC2=C(S1)C=CC=C2	159.21	C9H5NS	2.64
568	OC(=O)C1=CC(=CC=C1)S(=O)(=O)NCC#C	239.25	C10H9NO4S	0.69
569	OC(CC#C)C(O)=O	114.10	C5H6O3	-0.31
570	C#CC1=NC=NC=C1	104.11	C6H4N2	0.59
571	O=CC1=CC=C(OCC#C)C=C1	160.17	C10H8O2	1.76
572	OC(=O)C1=CC=C(C=C1)C#C	146.15	C9H6O2	1.78
573	O=S1(=O)CCN(CC#C)CC1	173.23	C7H11NO2S	-0.92
574	NC1=CC=CC(=C1)C#C	117.15	C8H7N	1.30
575	NS(=O)(=O)C1=CC=C(C=C1)C#C	181.21	C8H7NO2S	0.73
576	C#CCNC1CCC2=C1C=CC=C2	171.24	C12H13N	2.30
577	CC(C)C(NC(=O)NCC1=CC=CC=C1)C(O)=O	250.30	C13H18N2O3	1.74
578	CCCN1C(=O)NC(=O)C2=C1N=C(C=C2C(O)=O)C1CC1	289.29	C14H15N3O4	1.44
579	CC1=C(N=NN1C1=CC=C(F)C=C1)C(O)=O	221.19	C10H8FN3O2	2.06
580	OC(=O)CCC1=NNC(=O)NC1=O	185.14	C6H7N3O4	-1.04
581	OC(=O)C1=CC=C(NC(=O)NC2CC2)C=C1	220.23	C11H12N2O3	1.22
582	OC(=O)C1=CC=C(NC1=O)C1=CC=CC=C1	215.21	C12H9NO3	0.90
583	OC(=O)C1=NNC2=C1CCCCC2	180.21	C9H12N2O2	1.98
584	OC(=O)CCC(NC(=O)NC1=CC=C(F)C=C1)C(O)=O	284.24	C12H13FN2O5	0.94
585	O=C1CC2=C(NC3=C2C=CC=C3)C(=O)O1	201.18	C11H7NO3	1.42
586	O=C1OC(=O)C2CC12	112.08	C5H4O3	-0.09
587	OC(=O)C1=CC(NC(=O)NC2CC2)=CC=C1	220.23	C11H12N2O3	1.22
588	CN1N=C(C)C(Br)=C1C(O)=O	219.04	C6H7BrN2O2	0.88
589	OC(=O)C1CCCN1C(=O)C1=CC=C(Br)C=C1	298.14	C12H12BrNO3	2.14
590	OC(=O)C1=CC=C(C=C1)C(F)F	172.13	C8H6F2O2	2.02
591	OC(=O)[C@@H]1CCCN1CC#C	153.18	C8H11NO2	-1.52

592	OC(=O)C1=NNC2=C1C=C(I)C=C2	288.04	C8H5IN2O2	2.27
593	OC(=O)CCC(NC(=O)NC1=CC=C(C=C1)C#N)C(O)=O	291.26	C13H13N3O5	0.65
594	OC(=O)CC1(O)CCCCC1	158.20	C8H14O3	0.92
595	O=C1OC(=O)C2CCCC12	140.14	C7H8O3	0.80
596	CC1=NC(C(O)=O)=C(Cl)C=N1	172.57	C6H5ClN2O2	0.08
597	CC1=C(Br)C=NN1CC(O)=O	219.04	C6H7BrN2O2	0.27
598	O=C1OC(=O)[C@@H]2CC=CC[C@H]12	152.15	C8H8O3	0.89
599	NS(=O)(=O)C1=C(Cl)C=CC(=C1)C(O)=O	235.64	C7H6ClNO4S	0.84
600	OC(=O)CCCC1=CC=C(I)C=C1	290.10	C10H11IO2	3.43
601	OC(=O)C1=COC(=N1)C1CCCCC1	195.22	C10H13NO3	2.20
602	OC(=O)CN1CCC2=C(C=CC=C2)C1=O	205.21	C11H11NO3	0.79
603	OC(=O)CCN1C=CC2=C1C=CC=C2	189.21	C11H11NO2	2.01
604	CC1=CC(=NN1C1=CC(F)=CC=C1)C(O)=O	220.20	C11H9FN2O2	2.44
605	CC(=O)NC1=CC(C(O)=O)=C(F)C=C1	197.17	C9H8FNO3	1.01
606	OC(=O)CCCC1=NC2=C(C=CC=C2)C(=O)N1	232.24	C12H12N2O3	0.12
607	CC1=C2C=C(C=NC2=NN1)C(O)=O	177.16	C8H7N3O2	0.62
608	OC(=O)C1=C(C=CC=N1)C(F)(F)F	191.11	C7H4F3NO2	0.02
609	OC1=C(O)C=C(C=C1)C(=O)CN1C=NC2=C1C=C(C=C2)C(=O)NCC#C	349.35	C19H15N3O4	1.41
610	O=C(CN1C=NC2=CC(C(NCC#C)=O)=CC=C21)C3=CC(O)=C(C=C3)O	349.35	C19H15N3O4	1.41
611	OC1=C(O)C=C(C=C1)C(=O)CN1C=NC2=C1C=CC=C2C(=O)NCC#C	349.35	C19H15N3O4	1.41

4.3.5. HPLC purification of step 2 (pool step 2)

The **pool step 2** was purified using the following conditions:

Buffers: Buffer A (0.1 M TEAA), Buffer B (MeCN:H_2O=8:1, 0.1 M TEAA)

Gradient (% of buffer B): **5%** for 1 min., **5% → 18%** in 14 mins., **18% → 80%** in 5 mins., **80% → 100%** in 2 mins., **100%** for 7 mins. Flow = 4.00 mL/min.

Column temperature: 60°C

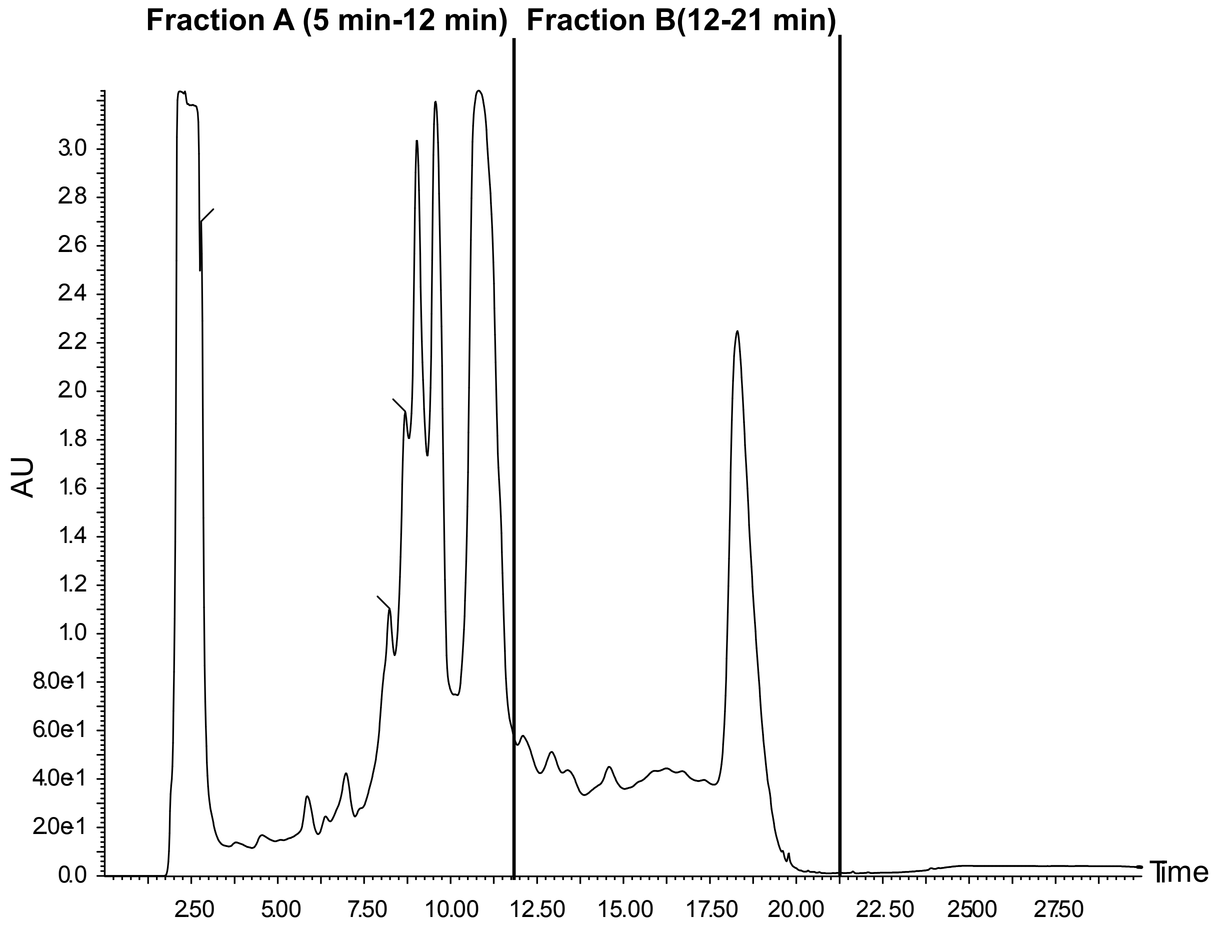

Supplementary figure 9: HPLC chromatogram registered at λ=260 nm. **Fraction 1** (5-12 minutes) contains: Splint oligonucleotide (**Ad1**) and unreacted **Codes B**. Fraction 2 (12-21 minutes) includes the final library (**pool step 2**).

After HPLC purification the **pool step 2** was isolated with a total yield of 49% (30 nmol).

4.3.6. 2 % Agarose gel of pool step 2 after HPLC purification

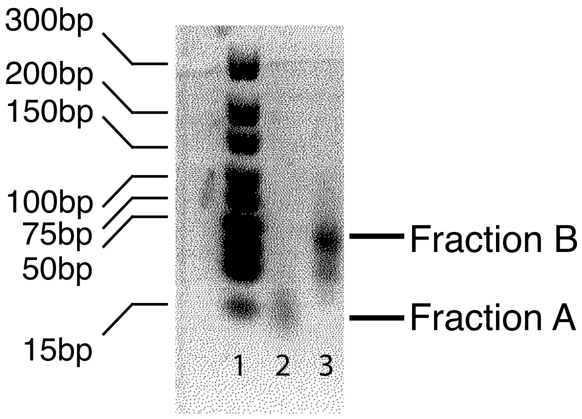

Supplementary figure 10: 2% agarose gel where **1** = ladder, **2** = **Fraction A** of the HPLC chromatogram containing **CodeB** (16 bp) unreacted and **Ad1** (11 bp); 3 = Fraction B of the HPLC chromatogram corresponding to **pool step 2** (38 bp).

4.4. Library design

Code A: 5′ GGAGCTTCTGAATTCTGTGTGCTGXXXXXXCGAGCGTCAGGCAGC **3′**

Code B: 3′ CTGTGTGCTGXXXXXXCGAGTCCCATGGCGC **5′**

Ad1 : 5′ GCAGrU-rC-rC-rG-TCGGArC-rA-rC-rA-CGAC **3′**

Glu DEL:

5′ GGAGCTTCTGAATTCTGTGTGCTGXXXXXXCGAGCGTCAGGCAGCCTGTGTGCTGXXXXXXCGAGTCCCATGGCGC **3′**

Supplementary figure 11: Sequence of **CodeA, CodeB, Ad1** and the final library (**Glu-DEL**).

5. Dual pharmacophore 2+1 affinity maturation libraries

5.1. Synthesis and characterization of known ligands (HIT 1-5) to protein targets

Five ligands (**HITs**) were selected for dual pharmacophore 2+1 affinity maturation library formation. The chemical structure of **HITs** and their protein targets are summarized in **Supplementary Figure 11.**

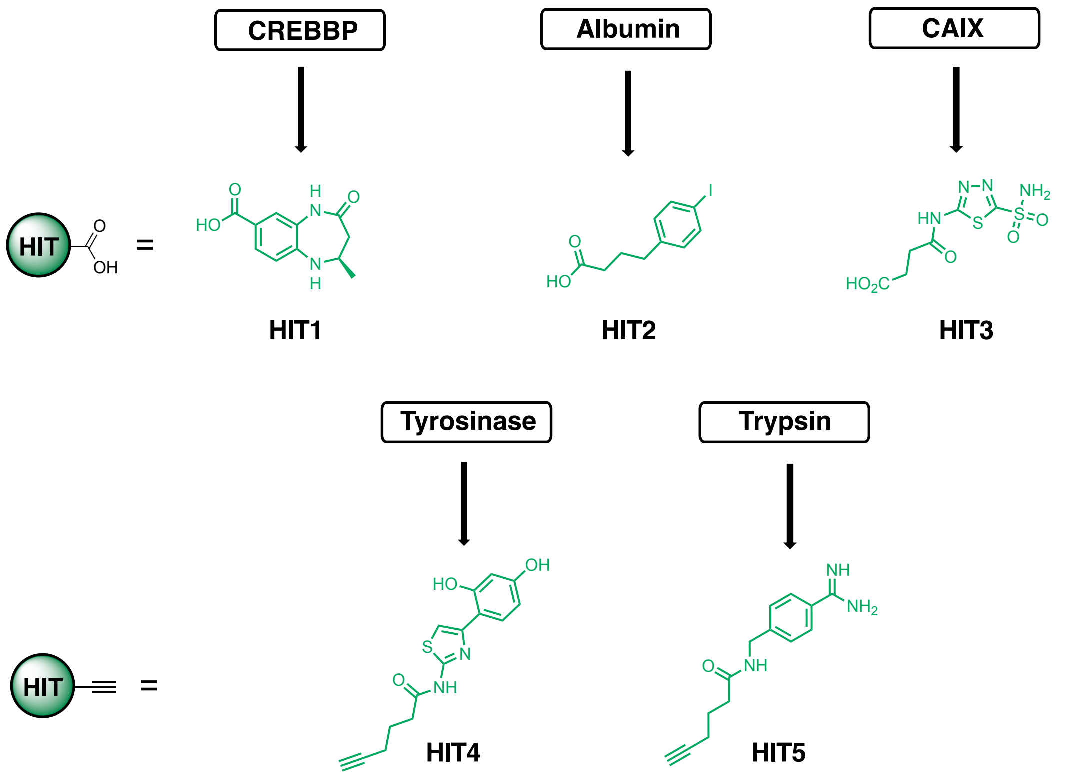

Supplementary figure 11: Ligands selected for 2+1 affinity maturation. **HIT1-3** are carboxylic acid derivatives of reported ligands: **HIT1** binds to CREB binding protein with a Kd = 32 uM;[2] **HIT2** binds to human serum albumin (HSA)[3] with a Kd = 2 uM; **HIT3** binds to Carbonic anhydrase IX with a Kd = 20 nM;[4] **HIT4-5** are alkyne derivatives of published inhibitors: **HIT4** is a tyrosinase Inhibitor (IC_{50} = 3 uM)[5] and **HIT5** is a benzamidine derivative described as trypsin inhibitor(IC_{50} = 2 uM).[6]

5.1.1. CREBBP ligand HIT1

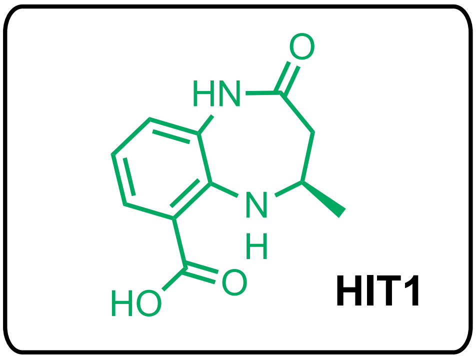

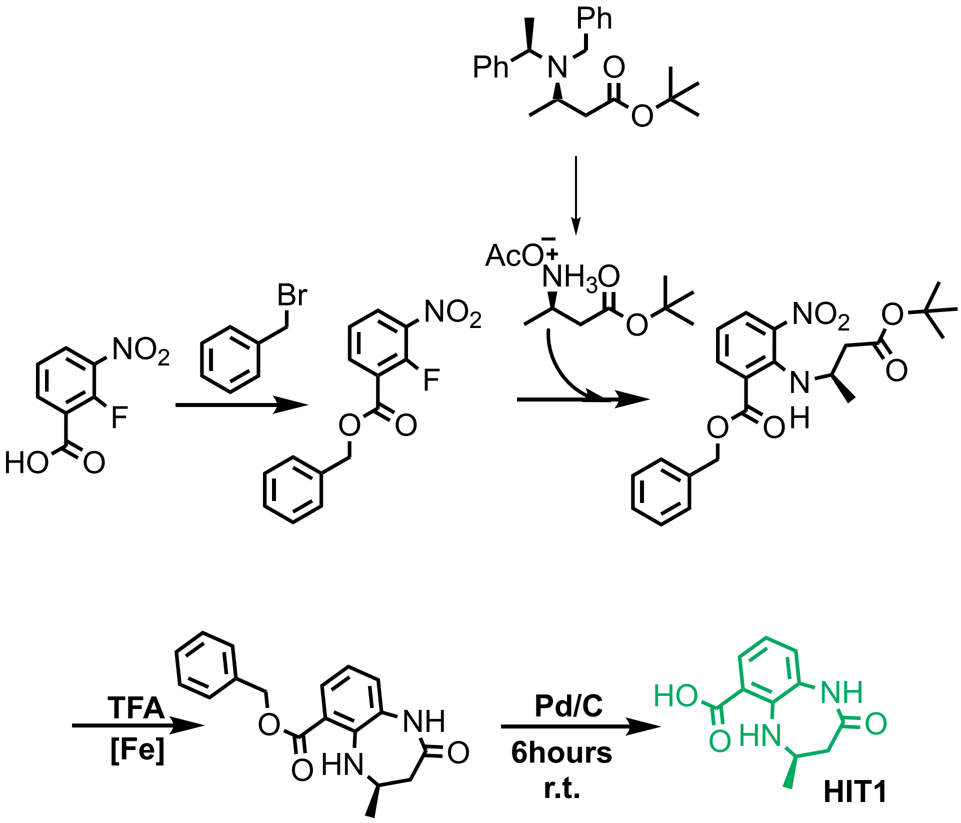

Supplementary figure 12: Synthetic strategy for **HIT1** formation

***tert*-Butyl (*R*)-3-(benzyl((*R*)-1'-phenylethyl)amino)butanoate**

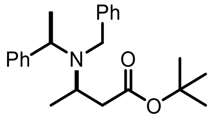

To a stirred colourless solution of (*R*)-*N*-benzyl-1-phenylethan-1-amine (4.04 g, 19.1 mmol, 1.5 eq.) in anhydrous THF (22 mL) at −78 °C was added *n*-BuLi in *n*-hexane (2.5 M, 8.16 mL,

20.1 mmol, 1.6 eq.), dropwise over a period of 15 min, and the pink reaction mixture was stirred at −78 °C for 30 min. *tert* Butyl crotonate (2.04 mL, 1.81 g, 12.8 mmol, 1.0 eq.) in THF (16 mL) was added dropwise over a period of 1 h and the reaction mixture was stirred at −78 °C for a further 4 h. The reaction mixture was quenched with sat. aq. NH_4Cl (20 mL) and was warmed to rt. The reaction mixture was evaporated *in vacuo*. 0.5 M aq. Citric acid (75 mL) was added to the residue and the resulting mixture was extracted with CH_2Cl_2 (3 × 100 ml). The combined organic components were washed with sat. aq. $NaHCO_3$ (150 mL), brine (150 mL) dried (Na_2SO_4), filtered, and evaporated *in vacuo* to afford a colourless oil. The crude oil was purified by silica gel chromatography (elution with 3% Et_2O in petroleum ether) to yield *tert*-butyl (*R*)-3-(benzyl((*R*)-1'-phenylethyl)amino)butanoate as a colourless oil (3.75 g, 83%); R_f 0.52 (Et_2O/*n*-hexane 1:9); $[\alpha]_D^{25}$= −3.9 (*c* 1.0, $CHCl_3$); [Lit:[2] $[\alpha]_D^{25}$= −5.2 (c 1.1, $CHCl_3$)]; ^1H NMR (400 MHz; $CDCl_3$) δ_H 7.46–7.10 (10H, m), 3.89 (1H, q, *J* 6.9), 3.76 (1H, d, *J* 15.0), 3.61 (1H, d, *J* 15.0), 3.42 (1H, dqd, *J* 9.1, 6.7, 4.7), 2.25 (1H, dd, *J* 14.1, 4.7), 2.01 (1H, dd, *J* 14.1, 9.1), 1.39 (9H, s), 1.33 (3H, d, *J* 6.9), 1.11 (3H, d, *J* 6.7); LRMS *m/z* (ESI^+) 354 ($[M+H]^+$, 100%). These data are in accordance with the literature.[7]

***tert*-Butyl (*R*)-3-aminobutanoate**

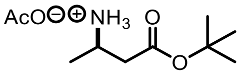

20 % $Pd(OH)_2$/C (1.80 g, 2.57 mmol, 0.2 eq.) was added to a stirred solution of *tert*-butyl (*R*)-3-(benzyl((*R*)-1'-phenylethyl)amino)butanoate (4.54 g, 12.8 mmol, 1.0 eq.) in H_2O (7.3 mL), glacial acetic acid (4.6 mL) and MeOH (182 mL), which was purged under a nitrogen atmosphere. The atmosphere was replaced with hydrogen gas and the suspension was stirred at rt for 20 h. The black suspension was filtered through Celite® (eluent MeOH) and evaporated *in vacuo* to yield a yellow oil. The oil was azeotroped with toluene (3 × 50 mL) to yield *tert*-butyl (*R*)-3-aminobutanoate containing 1.24 eq. acetic acid as colourless solid (2.85 g, 95%); R_f 0.00 (EtOAc); $[\alpha]_D^{25}$= −17.3 (*c* 0.5, $CHCl_3$), [Lit:[2] $[\alpha]_D^{25}$= −22.2 (*c* 0.5, $CHCl_3$)]; ^1H NMR (400 MHz; $CDCl_3$) δ_H 3.57–3.47 (1H, m), 2.62–2.42 (2H, m), 2.09 (9H, s), 1.29 (3H, d, *J* 6.6); LRMS *m/z* (ESI^+) 160 ($[M+H]^+$, 81%). These data are in accordance with the literature.[7]

Benzyl 2-fluoro-3-nitrobenzoate

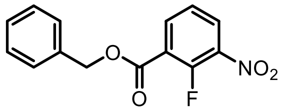

To a solution of 2-fluoro-3-nitrobenzoic acid (1.00 g, 5.40 mmol, 1.0 eq.) in DMF (12.5 mL) was added K_2CO_3 (1.12 g, 8.10 mmol, 1.5 eq.) and the reaction mixture was stirred at rt for 5 min. Benzyl bromide (964 µL, 8.10 mmol, 1.5 eq.) was added and the reaction mixture was stirred at 60 °C for 17 h. The cooled reaction mixture was diluted with EtOAc (150 mL) and was washed with 1 M aq. K_2CO_3 (3 × 100 mL). The organic component was washed with 0.5 M aq. LiCl (3 × 100 mL), brine (100 mL), dried ($MgSO_4$), filtered, and evaporated *in vacuo* to afford an orange solid. The crude solid was purified by silica gel chromatography (elution with 10% to 20% EtOAc in petroleum ether) to yield benzyl 2-fluoro-3-nitrobenzoate as a pale yellow oil (1.33 g, 89%); R_f 0.19 (EtOAc/petroleum ether 1:9); ^{1}H NMR (400 MHz; $CDCl_3$) δ_H 8.28–8.15 (2H, m), 7.49–7.33 (6H, m), 5.42 (2H, s); ^{19}F NMR (376 MHz; $CDCl_3$) δ_F −116.1 (dd, *J* 6.4, 6.4); LRMS *m/z* (ESI$^+$) 298 ([M+Na]$^+$, 100%). These data are in accordance with the literature.[7]

Benzyl (*R*)-2-((4-(*tert*-butoxy)-4-oxobutan-2-yl)amino)-3-nitrobenzoate

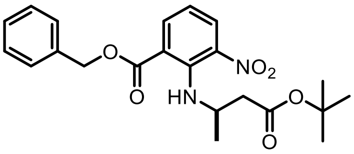

To a stirred suspension of benzyl 2-fluoro-3-nitrobenzoate (939 mg, 3.41 mmol, 1.0 eq.) and Cs_2CO_3 (3.34 g, 10.2 mmol, 4.0 eq.) in toluene (54 mL) at rt was added *tert*-butyl (*R*)-3-aminobutanoate containing 1.63 eq. acetic acid (1.23 g, 4.78 mmol, 1.2 eq.) and the suspension was stirred at 85 °C for 18 h. To the cooled suspension was added 1 M aq. K_2CO_3 (150 mL) and the resulting mixture was extracted with EtOAc (3 × 150 mL). The combined organic components were washed with brine (100 mL), dried ($MgSO_4$), filtered, and evaporated *in vacuo* to afford an orange oil. The crude oil was purified by silica gel

chromatography (elution with 10% to 20% EtOAc in petroleum ether) to yield benzyl (*R*)-2-((4-(*tert*-butoxy)-4-oxobutan-2-yl)amino)-3-nitrobenzoate as an orange oil (1.40 g, 99%); R_f 0.33 (EtOAc/petroleum ether 1:4); $[\alpha]_D^{25}$= +69.6 (*c* 1.0, $CHCl_3$); ^{1}H NMR (400 MHz; $CDCl_3$) δ_H 8.20 (1H, d, *J* 9.8), 8.04 (1H, dd, *J* 8.0, 1.7), 7.94 (1H, dd, *J* 8.0, 1.7), 7.47–7.33 (5H, m), 6.70 (1H, dd, *J* 7.8, 7.8), 5.35 (2H, s), 3.63 (1H, m), 2.39 (2H, dd, *J* 6.4, 1.8), 1.33 (9H, s), 1.25 (3H, d, *J* 6.4); LRMS *m/z* (ESI^+) 437 ($[M+Na]^+$, 100%). These data are in accordance with the literature.[7]

Benzyl (*R*)-4-methyl-2-oxo-2,3,4,5-tetrahydro-1*H*-benzo[*b*][1,4]diazepine-6-carboxylate

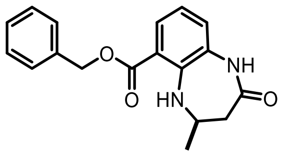

To a solution of benzyl (*R*)-2-((4-(*tert*-butoxy)-4-oxobutan-2-yl)amino)-3-nitrobenzoate (1.29 g, 3.11 mmol 1.0 eq.) in CH_2Cl_2 (10 mL) was added trifluoroacetic acid (10 mL) and the orange solution was stirred at rt for 2 h. The reaction mixture was evaporated *in vacuo* and the resulting residue was dissolved in glacial acetic acid (30 mL). Iron powder (868 mg, 15.6 mmol, 5.0 eq.) was added and the brown suspension was stirred under reflux for 4 h. 1 M aq. K_2CO_3 (300 mL) was added to the cooled suspension and the resulting mixture was extracted with EtOAc (3 × 200 mL). The combined organic components were washed with 0.5 M aq. LiCl (3 × 100 mL), brine (150 mL), dried ($MgSO_4$), filtered, and evaporated *in vacuo* to afford a brown oil. The crude oil was purified by silica gel chromatography (elution with 0% to 50% EtOAc in petroleum ether) to afford benzyl (*R*)-4-methyl-2-oxo-2,3,4,5-tetrahydro-1*H*-benzo[*b*][1,4]diazepine-6-carboxylate as a pale yellow solid (632 mg, 65%); R_f 0.14 (EtOAc/petroleum ether 2:3); $[\alpha]_D^{25}$= −49.1 (*c* 1.0, $CHCl_3$); mp 105–107 °C (EtOAc); ^{1}H NMR (400 MHz; $CDCl_3$) δ_H 8.00 (1H, br s), 7.87 (1H, br s), 7.84 (1H, dd, *J* 7.9, 1.6), 7.47–7.31 (5H, m), 6.99 (1H, dd, *J* 7.9, 1.6), 6.67 (1H, dd, *J* 7.9, 7.9), 5.32 (2H, s), 4.12–4.02 (1H, m), 2.68 (1H, d, *J* 14.2), 2.58 (1H, dd, *J* 14.2, 8.3), 1.39 (3H, d, *J* 6.5); LRMS *m/z* (ESI^+) 311 ($[M+H]^+$, 100%). These data are in accordance with the literature.[7]

(*R*)-4-Methyl-2-oxo-2,3,4,5-tetrahydro-1*H*-benzo[*b*][1,4]diazepine-6-carboxylic acid

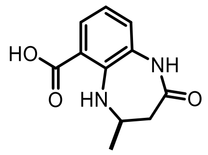

10% Pd/C (33 mg, 0.030 mmol, 0.1 eq.) was added to a stirred solution of benzyl (*R*)-4-methyl-2-oxo-2,3,4,5-tetrahydro-1*H*-benzo[*b*][1,4]diazepine-6-carboxylate (94 mg, 0.30 mmol, 1.0 eq.) in EtOAc (2 mL) and MeOH (2 mL), which was purged under a nitrogen atmosphere. The atmosphere was replaced with hydrogen gas and the suspension was stirred at rt for 6 h. The black suspension was filtered through Celite® and washed with MeOH (50 mL). The filtrate was evaporated *in vacuo* to yield (*R*)-4-methyl-2-oxo-2,3,4,5-tetrahydro-1*H*-benzo[*b*][1,4]diazepine-6-carboxylic acid as a yellow solid (24 mg, 37%); R_f 0.11 (AcOH/EtOAc 1:99); $[\alpha]_D^{25}$= −34.8 (*c* 0.5, MeOH); mp 254–257 °C (EtOAc); $\bar{\nu}_{max}$ (thin film)/cm^{-1} 3030 (C-H, w), 1655 (C=O, s); ^{1}H NMR (500 MHz; DMSO-d_6) δ_H 9.55 (1H, br s), 7.63 (1H, dd, *J* 7.8, 1.6), 7.07 (1H, dd, *J* 7.8, 1.6), 6.68 (1H, dd, *J* 7.8, 7.8), 3.95 (1H, m), 2.50 (1H, m), 2.37 (1H, dd, *J* 13.8, 8.2), 1.24 (3H, d, *J* 6.3); ^{13}C NMR (126 MHz; DMSO-d_6) δ_C 171.3, 170.3, 142.6, 127.9, 127.6, 126.4, 116.8, 51.9, 41.8, 23.8; HRMS *m/z* (ESI$^+$) [Found: 221.0921, $C_7H_{13}O_3N_2$ requires M$^+$ 221.0921]; LRMS *m/z* (ESI$^-$) 219 ([M-H]$^-$, 100%); HPLC Method 2: Retention time 5.2 min, purity 99.1%.

5.1.2. Human serum albumin (HSA) ligand HIT2

4-(4-iodophenyl)butanoic acid (**HIT2**) is commercially available and was purchased from ABCR GmbH (Germany).

5.1.3. Carbonic anhydrase IX ligand HIT3

4-oxo-4-((5-sulfamoyl-1,3,4-thiadiazol-2-yl)amino)butanoic acid (**HIT3**) is commercially available and was purchased from ABCR GmbH (Germany).

5.1.4. Tyrosinase ligand HIT4

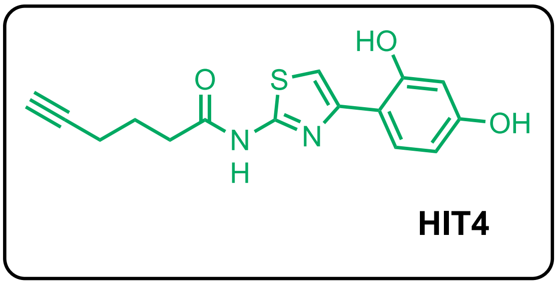

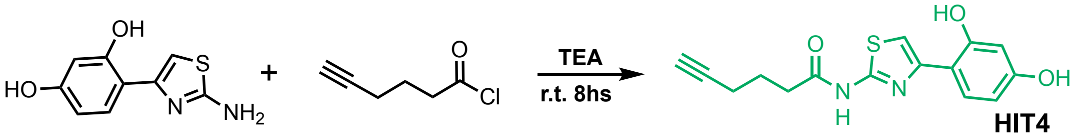

Supplementary figure 13: Synthetic strategy for **HIT4** formation

In a round bottom flask 50 mg (0.24 mmol, 1 eq) of 4-(2-aminothiazol-4-yl)benzene-1,3-diol and 31 mg (0.24 mmol, 1.2 eq) of hex-5-ynoyl chloride were dissolved in 10 mL of DMF. Triethylamine (0.17 mL, 1.2 mmol, 5 eq.) was added dropwise to the stirring solution and the obtained solution was allowed to react eight hours at room temperature. The solvent was evaporated under reduced pressure and the obtained product was purified by HPLC. The pure compound N-(4-(2,4-dihydroxyphenyl)thiazol-2-yl)hex-5-ynamide (**HIT4**) was obtained with 52% yield**.** ^{1}**H NMR** (400 MHz, DMSO-d6): δ 12.19 (s, 1H), 10.79 (s, 1H), 9.37 (s, 1H), 7.59 (d, J = 8.3 Hz, 1H), 7.34 (s, 1H), 6.31 – 6.14 (m, 2H), 2.76 (t, J = 2.6 Hz, 1H), 2.50 (t, J = 7.4 Hz, 2H), 2.17 (td, J = 7.1, 2.7 Hz, 2H), 1.72 (p, J = 7.2 Hz, 2H). ^{13}**C NMR** (101 MHz, DMSO): δ 171.23, 158.84, 157.32, 156.89, 147.00, 128.73, 111.16, 107.64, 105.77, 103.46, 84.23, 72.26, 34.24, 23.90, 17.76. MS (m/z) $[M^+H^+]$ $C_{15}H_{14}N_2O_3S$ calcd: 302.07, found: 302.0596.

5.1.5. Synthesis of trypsin ligand HIT5

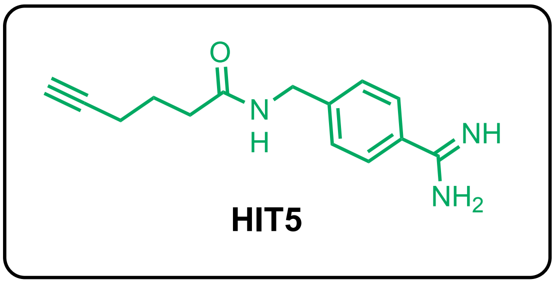

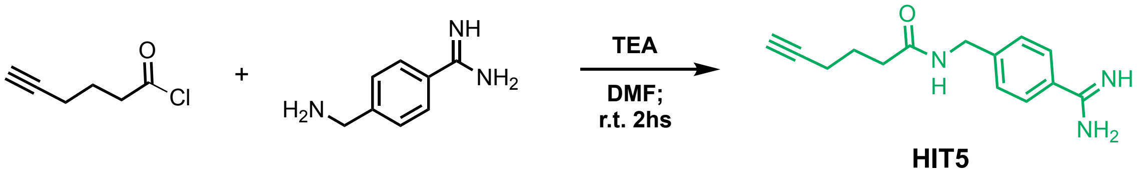

Supplementary figure 14 : Schematic representation of **HIT5** synthesis

In a round bottom flask 150 mg (0.8 mmol, 1 eq) of 4-(aminomethyl)benzamidine dihydrochloride and 125 mg (0.96 mmol, 1.2 eq) of hex-5-ynoyl chloride were dissolved in 20 mL of DMF. Triethylamine (0.33 mL, 2.4 mmol, 3 eq) was added dropwise to the stirring solution and the obtained solution was allowed to react overnight at room temperature. The solvent was evaporated under reduced pressure and the obtained product was purified by HPLC. The pure compound *N*-(4-carbamimidoylbenzyl) hex-5-ynamide (**HIT5**) was obtained with **52%** yield. **^{1}H NMR** (400 MHz, DMSO-d_6): δ 8.20 (d, *J* = 8.1 Hz, 1H), 7.33 (dd, *J* = 7.7, 1.1 Hz, 1H), 7.24 (d, *J* = 8.1 Hz, 1H), 7.13 (d, *J* = 8.2 Hz, 2H), 7.00 – 6.93 (m, 3H), 6.89 (t, *J* = 7.4 Hz, 1H), 6.10 (s, 1H), 4.35 (td, *J* = 8.5, 5.1 Hz, 1H), 4.13 (t, *J* = 7.1 Hz, 2H), 2.89 (dd, *J* = 13.7, 5.1 Hz, 1H), 2.71 (dd, *J* = 13.7, 8.9 Hz, 1H), 2.48 – 2.39 (m, 5H), 2.35 – 2.25 (m, 5H), 1.47 (h, *J* = 7.2 Hz, 2H), 0.92 (t, *J* = 7.4 Hz, 3H) **^{13}C NMR** (101 MHz, DMSO) δ 171.07, 165.96, 159.21, 158.90, 158.59, 146.39, 128.53, 127.85, 126.97, 84.22, 71.91, 42.20, 34.63, 14.76. MS (ESI) (m/z) [M^+H^+] $C_{14}H_{17}N_3O$ calcd: 243.14.

5.2. Conjugation of HITs to 24mer DNA (DB24)

A universal 24mer oligonucleotide (**DB24**), complementary to the **Glu-DEL** 5′ end with the sequence: 5′-CAGCACACAGAATTCAGAAGCTCC-3'-C6-NH_2 was used for the synthesis of oligo (**DB24**)-ligand (**HIT**) conjugates.

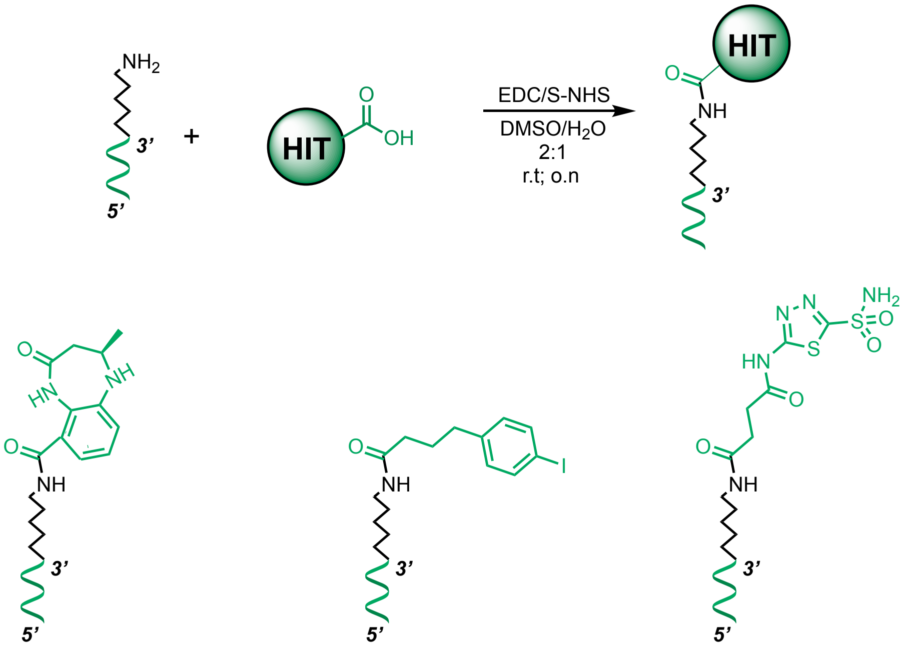

DB24-HIT1 **DB24-HIT2** **DB24-HIT3**

Supplementary figure 15: Schematic representation of the **DB24-HIT(1/2/3)** oligo conjugates complementary to **Glu-DEL.**

DB24-HIT1, DB24-HIT2 and **DB24-HIT3** were synthetized following the procedure described in section **4.1.1.** LC-MS characterization of the products is shown in **supplementary table 3.**

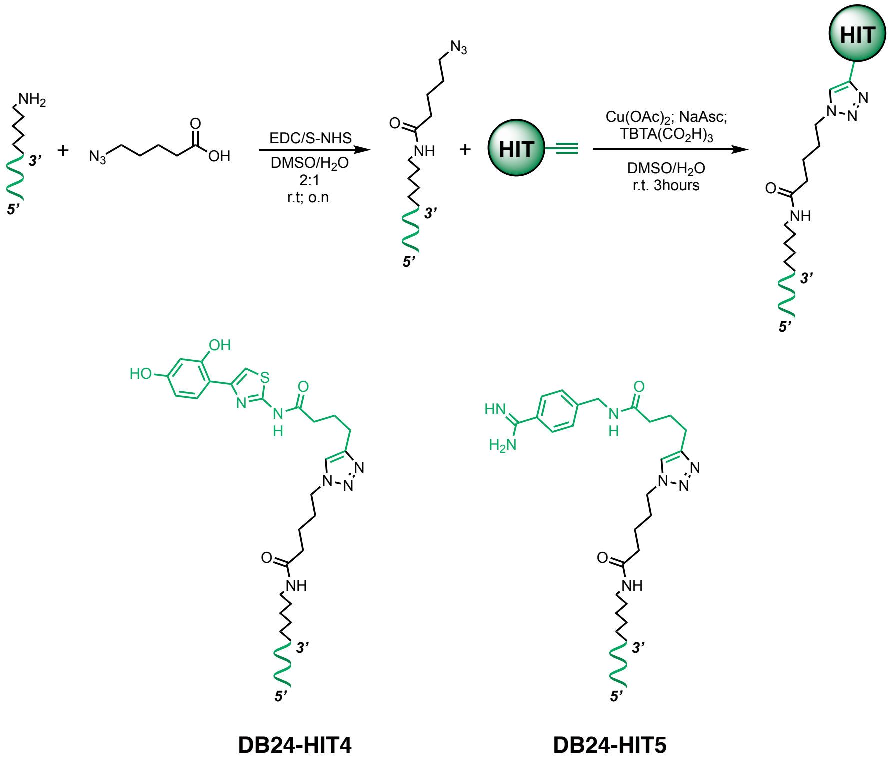

Supplementary figure 16: Schematic representation of the **DB24-HIT (4/5)** oligo conjugates complementary to **Glu-DEL.**

DB24-HIT4 and **DB24-HIT5** were synthetized by first coupling of 5-azido pentanoic acid to DB24 (**4.1.1**) followed by CuAAC click reaction (**4.1.7**). LC-MS characterization of the products is shown in **supplementary table 3.**

oligo(**DB24**)-ligand(**HIT**)	**MS$_{Calculated}$**	**MS$_{found}$**
**DB24-HIT1**	7680	7681
**DB24-HIT2**	7750	7750
**DB24-HIT3**	7740	7741
**DB24-HIT4**	7905	7907
**DB24-HIT5**	7846	7849

supplementary table 3: MS characterization of **DB24-HITs** conjugates used for the construction of **Glu-DEL** derived affinity maturation 2+1 libraries.

5.3. Formation of dual pharmacophore 2+1 affinity maturation libraries

a)

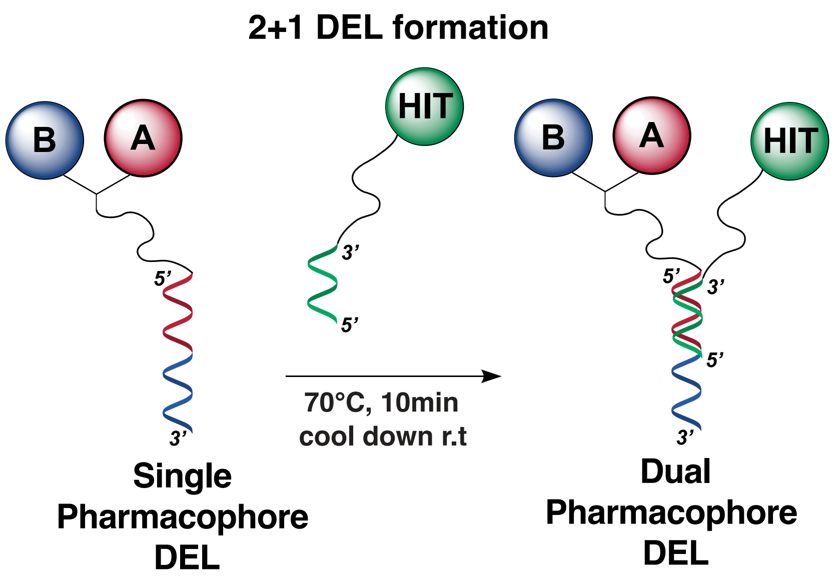

b)

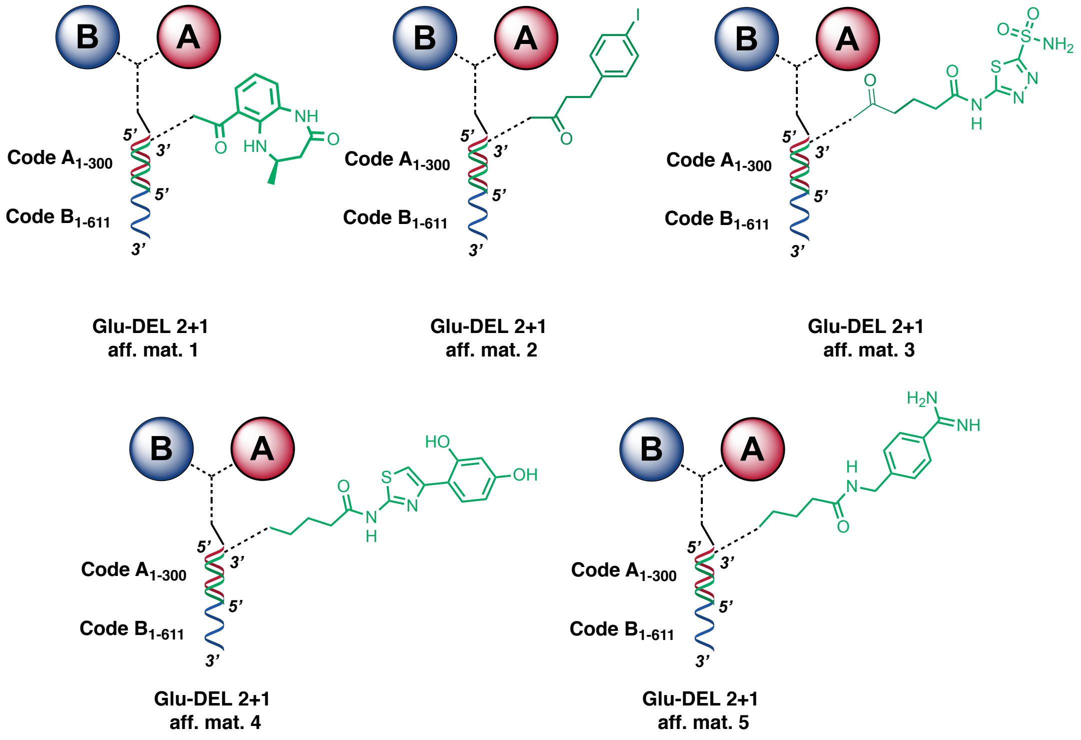

Supplementary figure 17: a) Schematic representation of the **Glu-DEL** annealing to the **DB24-HITs** conjugates. In panel **(b)** from top left (target): **Glu-DEL 2+1 aff. mat. 1** (CREBBP)**, Glu-DEL 2+1 aff. mat. 2** (HSA)**, Glu-DEL 2+1 aff. mat. 3** (CAIX), **Glu-DEL 2+1 aff. mat. 4** (Tyrosinase), **Glu-DEL 2+1 aff. mat. 5** (Trypsin).

DB24-HIT conjugates (1.2 nmol each) were individually incubated with **Glu-DEL** at 1.2:1 ratio (**supplementary figure 17-a**). The resulting mixtures were first heated at 70°C for 10 minutes and allowed to cool down to room temperature. The resulting dual pharmacophore 2+1 affinity maturation **Glu-DEL aff. mat.** libraries (**supplementary figure 17-b**) were analysed by SDS-PAGE to evaluate annealing outcome (**supplementary figure 18**).

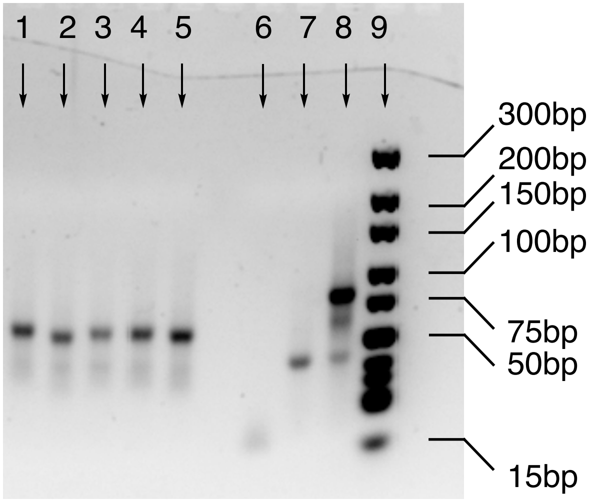

Supplementary figure 18: 2% agarose SDS of **1** = **Glu-DEL 2+1 aff. mat. 1** (50bp), **2 = Glu-DEL 2+1 aff. mat. 2** (50bp)**, 3 = Glu-DEL 2+1 aff. mat. 3** (50bp), **4 = Glu-DEL 2+1 aff. mat. 4** (50bp), **5 = Glu-DEL 2+1 aff. mat. 5** (50bp), **6** = **DB24** (12bp), **7 =** single stranded **Glu-DEL**, **8 =** double stranded **Glu-DEL** (76bp), **9 =** ladder.

6. Affinity selections

6.1. Selection procedure

Affinity selections were performed with both single (ss) and double stranded (ds) libraries with 10^7 copies of individual library members for each selection.[8] Each selection was performed in duplicate or in triplicate (**supplementary figure 24-30**). The ss-library and ds-library were diluted to 110 nM in protein-specific buffer, containing also 0.05% tween-20 and 20 µg/mL herring sperm DNA (100 µL). The selections against immobilized protein targets were performed with the automated system King Fisher (Thermo Fisher) as reported by Decurtins et al.[9]

6.2. Proteins for affinity selections

Supplementary table 4: List of screened protein targets.

Target	buffer	MW, Da	ε (280 nM), $M^{-1}cm^{-1}$	tag	beads	[protein], µM
CAIX	PBS, pH=7.4	32’732	34850	Biotin		
CREBBP	**HEPES 1**: 50 mM Hepes, 500 mM NaCl, pH=7.6	26’930	16’673	Biotin		
wt-PI3K	**HEPES 2**: 100 mM NaCl, 20 mM Hepes, 2 mM DTT, pH=7.5.	195K	277’950	Enzymatic Biotinylation		
Streptavidin	PBS, pH=7.4	-	-	-		
AASSc-214	500mM NaCl, **HEPES 3**: 5%Glycerol, 50mM HEPES(pH7.5), 0.5mM TCEP	52’021	46’550	Biotin	Dynabeads™ MyOne™ Streptavidin C1	2.0
Trypsin	PBS, pH=7.4	23’293	37’525	Biotin		
FAN-1wt	**TRIS**: 20 mM Tris, 150 mM NaCl, 5 mM β-mercaptoethanol, pH 8.0.	118’220	158’710	His-tag		
FAN-1D960A		118’220	158’710	His-tag		
Albumin	PBS, pH=7.4	66’561	34’445	Biotin		
Tyrosinase	PBS, pH=7.4	60’393	112’270	Biotin		

6.2.1. CAIX. Recombinant His6-tagged human CAIX was expressed and purified as previously described.[10] The protein was chemically biotinylated following the procedure described in section **6.2.11.**

6.2.2. CREBBP bromodomain. Protein was gently provided by Professor Stuart Conway laboratory. The CREBBP bromodomain (Addgene plasmid # 38977) construct was transformed into E. coli BL21 (DE3) cells for expression, as described previously.[11] The proteins were purified using Immobilized Metal Affinity Chromatography (IMAC) with an HisTrapTM column (GE Healthcare) followed by gel filtration chromatography with Superdex 75 resin (GE Healthcare). The protein purity was assessed by SDS-PAGE (**supplementary figure 19**). The protein was freshly biotinylated in house following the procedure described in **6.2.11**. Sodium dodecyl sulfate polyacrylamide gel was cast using Mini-PROTEAN® 3 Cell (Bio-Rad). SDS-PAGE was performed using Mini-PROTEAN® 3 Electrophoresis Module (Bio-Rad). The Tris-Glycine discontinuous buffer system was used in the SDS-PAGE experiments. Protein samples for SDS-PAGE was first prepared in 1× Laemmli sample buffer (diluted from 4× Laemmli sample buffer, Bio-Rad) containing 1.42 M β-mercaptoethanol (aliquoted from 14.2 M β-mercaptoethanol, Bio-Rad), and then denatured at 100 °C for 10 min. Chemicals for SDS-PAGE, Trizma® base, 30% acrylamide/bis-acrylamide solution, sodium dodecyl sulfate dust-free pellets, N,N,N′,N′-t etramethylethane-1,2-diamine (TEMED) and ammonium persulfate (APS), were purchased from Sigma Aldrich. Glycine was purchased from Fisher Scientific. After SDS-PAGE, the gels were stained with Staining Solution (0.5% Brilliant Blue G (Sigma Aldrich), 50% (v/v) MeOH (Sigma Aldrich), 10% (v/v) glacial acetic acid (Fisher Scientific), 40% (v/v) Milli-Q water) for 15 min. Destaining was performed with 40% (v/v) MeOH, 10% (v/v) glacial acetic acid, 50% (v/v) Milli-Q water until protein bands became visible.

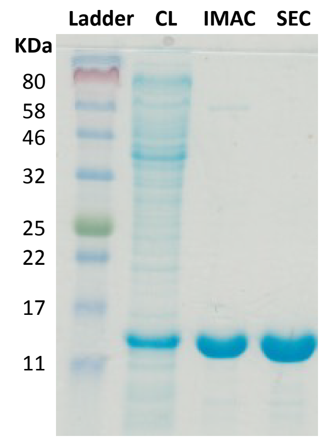

Supplementary figure 19: SDS PAGE of CREBBP (From left to right): **Ladder,** Cell lysate(**CL**), CREBBP sample after Ni IMAC purification (**IMAC**) and CREBBP sample after gel filtration (**SEC**)

6.2.3. PI3K. Proteins were kindly provided by Prof. Vogt's group. His-tagged p110α protein and Avi-tagged p85α protein were co-expressed in High Five insect cells (bti-tn-5b1-4, Thermo Fisher). Subsequently, p110α/p85α dimer proteins were purified by Ni-NTA chromatography, Heparin column chromatography and followed by gel filtration chromatography. Finally, SDS-PAGE (**supplementary figure 20**) was used to examine protein size and purity. The protein was enzymatically biotinylated following a protocol in BirA500 kit (AVIDITY).

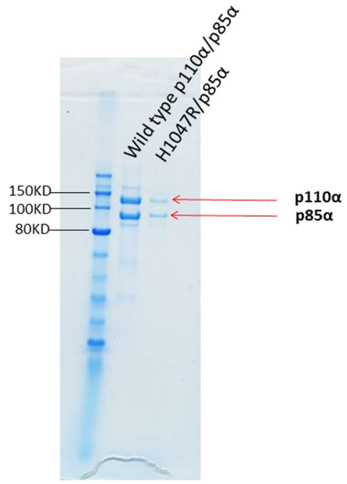

Supplementary figure 20: SDS PAGE of wt PI3K (**wild type p110α/p85α**)

6.2.4. Streptavidin. Dynabeads™ MyOne™ Streptavidin C1 were purchased from Thermo Fisher Scientific.

6.2.5. AASS. The protein was kindly provided by Structural Genomic Consortium Oxford (UK). The AASS454-926 DNA fragment encoding the saccharopine dehydrogenase domain was subcloned into the pFB-Bio5 vector incorporating an N-terminal His6-tag and a C-terminal biotin attachment site.

AASS454-926 was expressed in Sf9 cells with supplementation of biotin, and purified from cell pellets by Ni-NTA and size exclusion chromatography. The N-terminal His6-tag was removed by overnight treatment with TEV protease and protein was further purified from reverse Ni-NTA. The purified protein was verified by SDS-PAGE (not shown) and intact MS (**supplementary figure 21**).

Sequence of the purified protein (after His6-tag removal) is as follows:

```
SMTLPDKYKYIQTLRESRERAQSLSMGTRRKVLVLGSGYISEPVLEYLSRDGNIEITVGSDM
KNQIEQLGKKYNINPVSMDICKQEEKLGFLVAKQDLVISLLPYVLHPLVAKACITNKVNMVT
ASYITPALKELEKSVEDAGIIGELGLDPGLDHMLAMESIDKAKEVGATIESYISYCGGLPAP
EHSNNPLRYKFSWSPVGVLMNVMQSATYLLDGKVVNVAGGISFLDAVTSMDFFPGLNLEGYP
NRDSTKYAEIYGISSAHTLLRGTLRYKGYMKALNGFVKLGLINREALPAFRPEANPLTWKQL
LCDLVGISPSSEHDVLKEAVLKKLGGDNTQLEAAEWLGLLGDEQVPQAESILDALSKHLVMK
LSYGPEEKDMIVMRDSFGIRHPSGHLEHKTIDLVAYGDINGFSAMAKTVGLPTAMAAKMLLD
GEIGAKGLMGPFSKEIYGPILERIKAEGIIYTTQSTIKPSSKGGYGLNDIFEAQKIEWHE
```

where vector incorporated sequence is underlined and the C-terminal biotin attachment site is in red text. The protein was chemically biotinylated following the procedure described in section **6.2.11.**

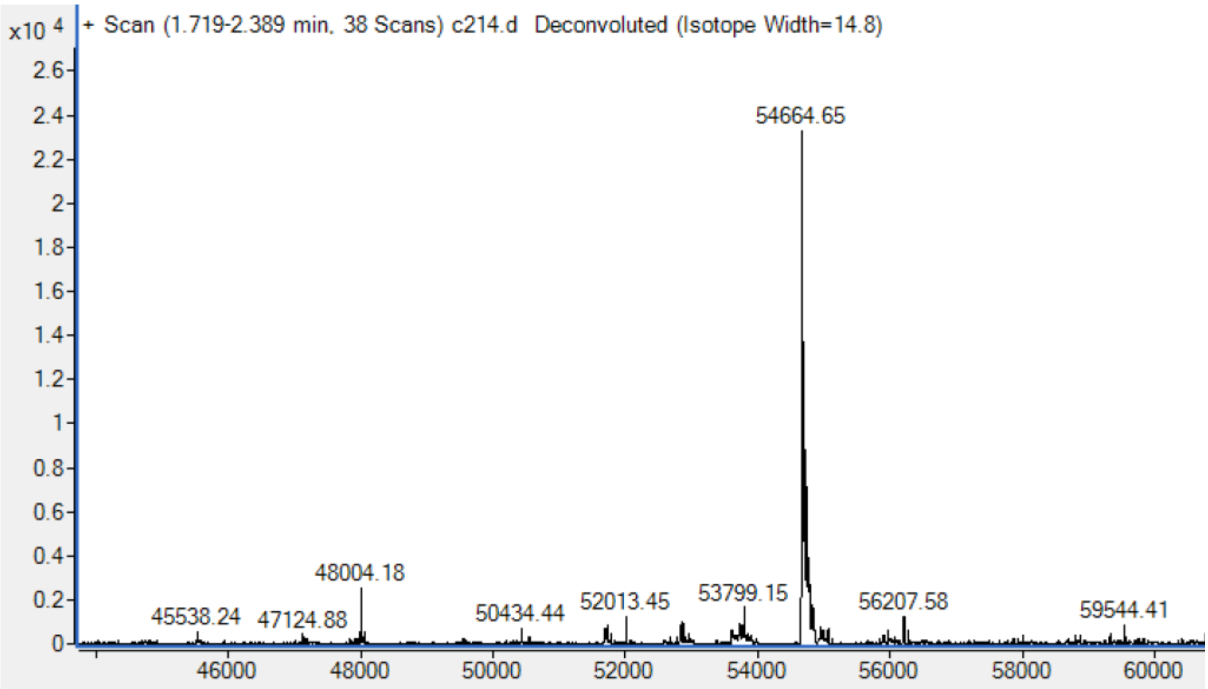

Supplementary figure 21 : Intact MS profile of purified $AASS_{454-926}$ construct. Mass detected corresponds to the expected protein that was biotinylated.

6.2.6. Trypsin. Trypsin from porcine pancreas was purchased from sigma Aldrich **CAS number 9002-07-7** and biotinylated in house following the procedure described in section **6.2.11.** After biotinylation, the protein was purified with a PD-10 column GE-Healtcare. LC-MS analysis of the protein samples were measured before and after biotinylation **supplementary figure 22.**

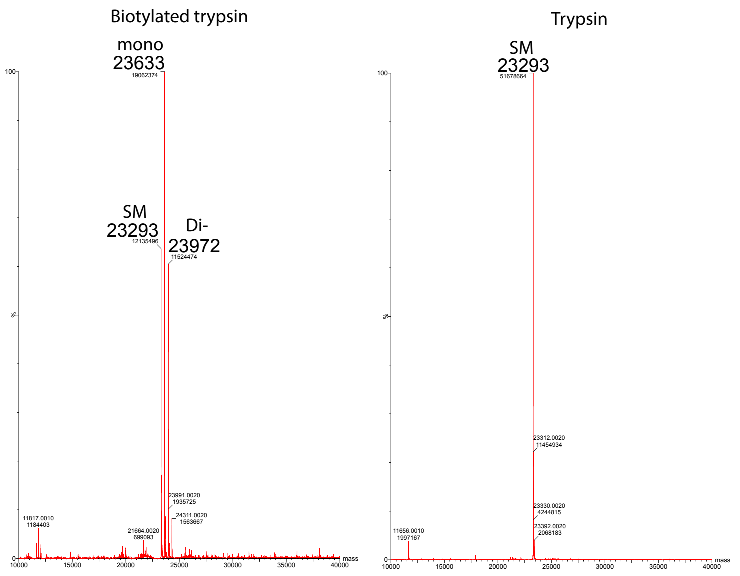

Supplementary figure 22: MS characterization of biotinylated trypsin (left) and unmodified trypsin (SM). The Molecular weight increase of 343 and 686 Da respectively, corresponds to mono and di- biotinylation of trypsin lysine residues as indicated in the ESI profile.

6.2.7. D960A FAN-1. FAN1 (aa 373-1017) wt and D960A were amplified by PCR from eGFP-FAN1 (pAIO vector)[12] and cloned into the pET28-MBP TEV vector using the In-Fusion cloning kit (Takara) (primer sequences in Table 5-1). FAN1 (aa 373-1017) was expressed in BL21 for 20 h at 18 °C in the presence of 0.5 mM IPTG. Soluble extracts were produced as for CtIP-NTD, loaded onto Ni-NTA affinity columns (5 ml HisTrap HP, GE Healthcare) and eluted with 20 mM Tris pH 8.0, 2 mM β-mercaptoethanol, 300 mM NaCl, 200 mM imidazole. Next, FAN1 was further purified by amylose chromatography (5 ml MBPTrap HP, GE Healthcare) and elution with 20 mM Tris pH 8.0, 2 mM β-mercaptoethanol, 300 mM NaCl, 20 mM maltose. Remaining contaminants were removed by preparative size-exclusion chromatography (Superdex 200 10/300 GL, GE Healthcare) in 20 mM Tris pH 8.0, 150 mM NaCl, 5 mM β-mercaptoethanol. Results are shown in **supplementary figure 23**. The protein was anchored to Ni beads used for selections via the His-tag residue. List of DNA oligonucleotides:

FAN1373-1017_F	ATACTTCCAAGGATCCCCTTACTACCTTCGGAGTTTCC
FAN1373-1017_R	GGTGGTGGTGCTCGAGGCTAAGGCTTTGGCTCTTAG

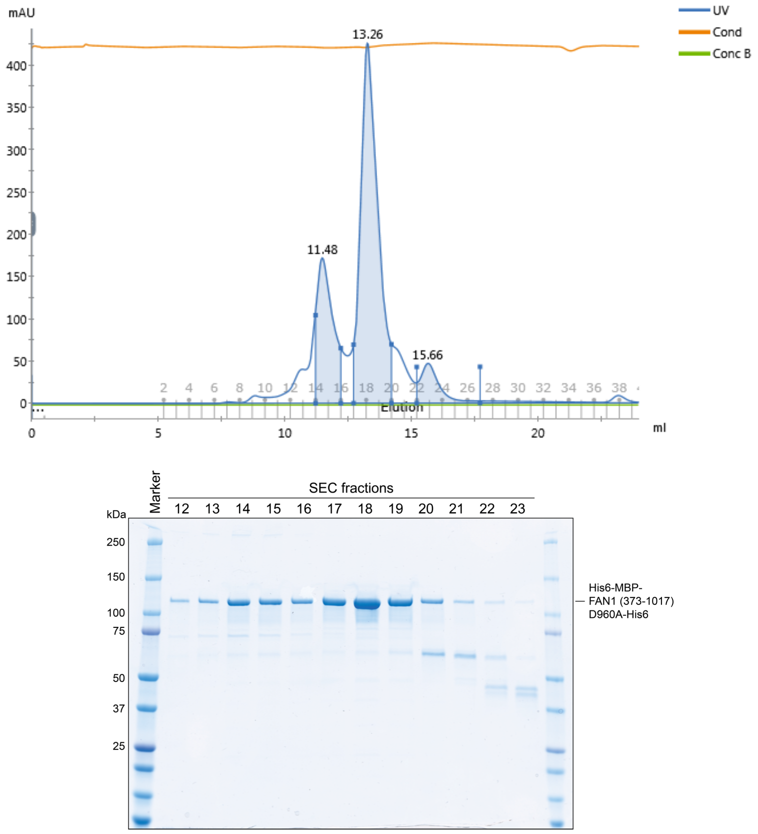

Supplementary figure 23: SEC chromatogram registered at λ= 280nm. Fractions 12-19 contains the target protein.

6.2.8. wt FAN-1 Details on expression and purification are described in section **6.2.7.**

6.2.9. Human serum albumin. HSA was purchased from Sigma Aldrich, CAS: 70024-90-7 and biotinylated in house following the protocol **5.2.13.** Results have been reported by Dumelin et al.[3]

6.2.10. Tyrosinase was kindly provided by Dr. Professor Montserrat Soler-Lopez from ESFR (The European Synchrotron Radiation Facility).

6.2.11. Protein biotinylation. All the proteins were freshly biotinylated using 3 equivalents of NHS-LC-Biotin (Thermofisher, Cat #: 21336). The reaction was kept at room temperature for 1 hour, quenched by addition of TRIS buffer and the product was purified by PD-10 column GE Healthcare. An LC-MS spectra was taken after purification to assess biotinylation outcome.

6.3. PCR amplification and sequencing

The selection eluates were amplified by two rounds of PCR as previously reported[9] using the following primers:

PCR1-a: 5'-TACACGACGCTCTTCCGATCTXXXXXXGGAGCTTCTGAATTCTGTGTG-3' where X represent a variable region which codify the selection.

PCR1-b_a: 5'-CAGACGTGTGCTCTTCCGATC TATCGA GCGCCATGGGACTCG

PCR1-b_b: 5'- CAGACGTGTGCTCTTCCGATC GATACT GCGCCATGGGACTCG

PCR2-a: 5'- AATGATACGGCGACCACCGAGATCTACACTCTTTCCCTACACGACGCTCTTCCGATCT-3'

PCR2-b: 5'-CAAGCAGAAGACGGCATACGAGATATTGGCGTGACTGGAGTTCAGACGTGTGCTCTTCCGATC-3'

The PCR products were sequenced by Illumina high-throughput sequencing and the data obtained were processed and analysed as previously reported.[9]

6.4. 2BB single pharmacophore selections replicates

6.4.1. Naïve library

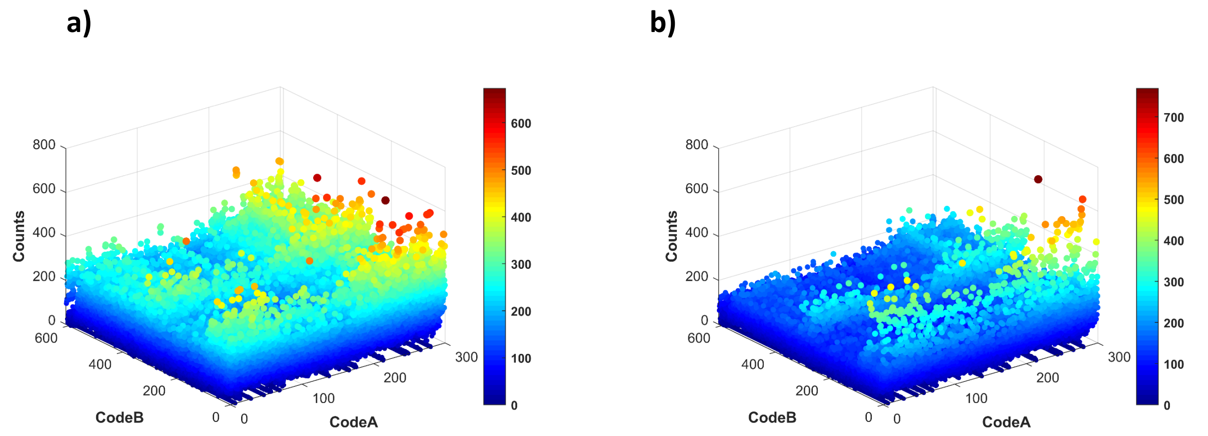

Supplementary figure 24: a), b) Fingerprints of unselected library. The combinations of code A and B are reported in the *xy* plane while the number of counts is visualized on the *z* axis. The total counts for the unselected library duplicates are 17’046’900 and 12’001’356, respectively.

Replicate selection fingerprints are given for model selections or where at least one hit compound from the selection was chosen for re-synthesis and validated. Results are reported in **supplementary figure 25-30**.

6.4.2. Streptavidin fingerprints

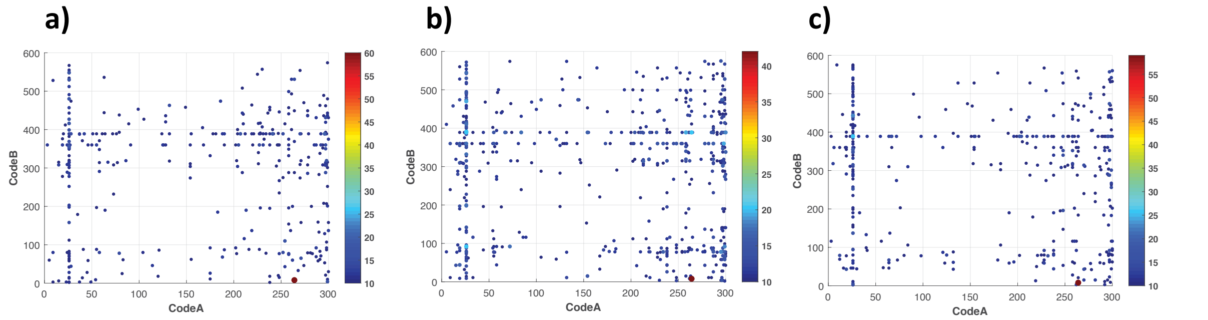

Supplementary figure 25: Fingerprint of selections performed against streptavidin beads (no protein). The total counts (**TCs**) for each replicate: **a)** TCs = 232’874, **b)** TCs = 260’182, **c)** TCs = 225’678 .

6.4.3. CAIX fingerprints

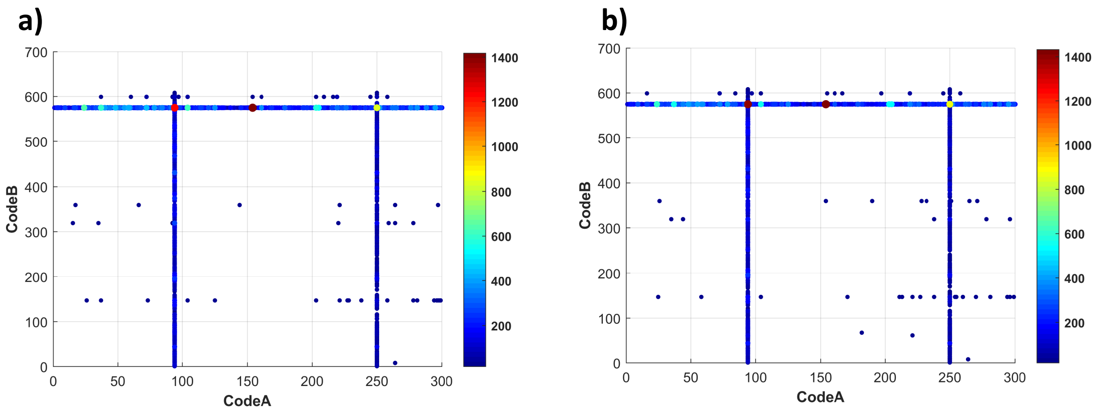

Supplementary figure 26: Fingerprint of selections performed against CAIX. The total counts (**TCs**) for each replicate are: **a)** TCs = 290’800, **b)** TCs = 240’300.

6.4.4. D960A FAN1 fingerprints

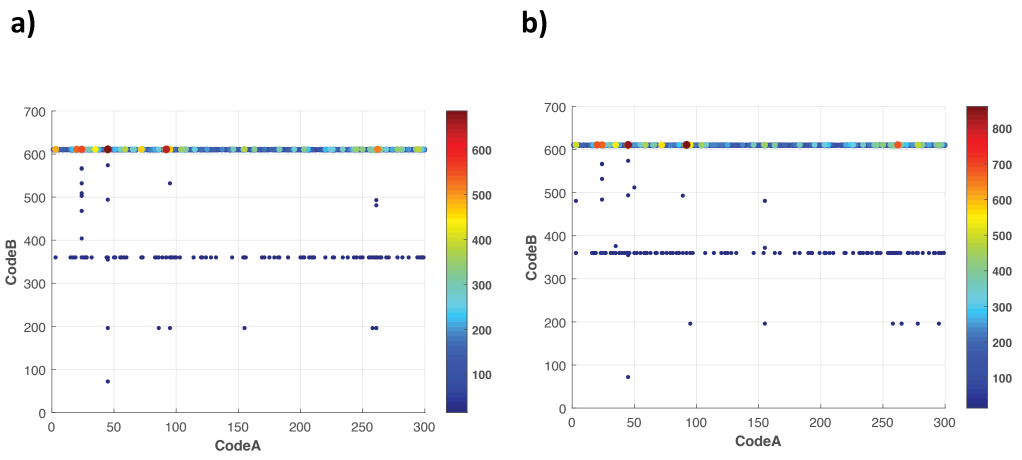

Supplementary figure 27: Fingerprint of selections performed against FAN1 D960A. The total counts (**TCs**) for each replicate are: **a)** TCs = 172’079, **b)** TCs = 198’004.

6.4.5. AASSA fingerprints

a) **b)** **c)**

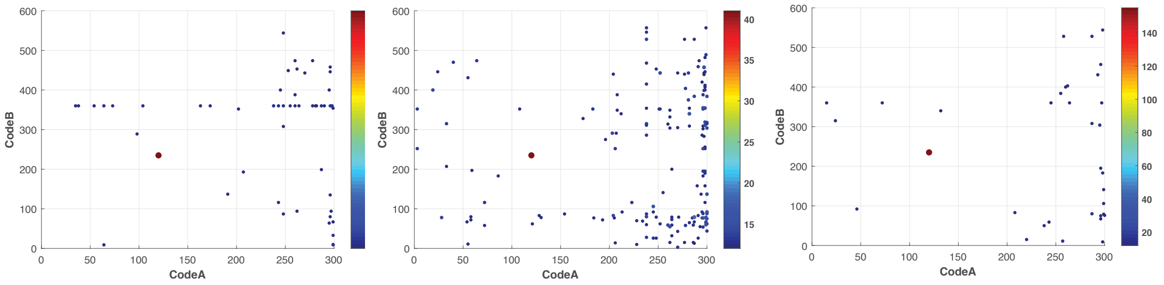

Supplementary figure 28: Fingerprint of selections performed against AASSA. The total counts (**TCs**) for each replicate are: **a)** TCs = 359’959, **b)** TCs = 291’442 **c)** TCs = 288’756.

6.4.6. PI3K fingerprints

a) **b)** **c)**

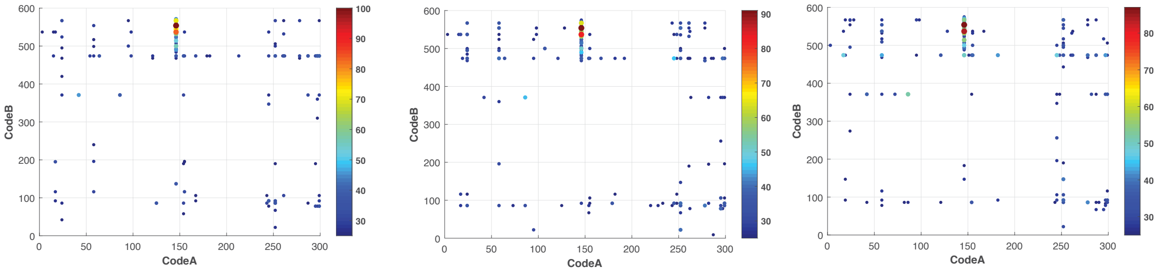

Supplementary figure 29: Fingerprint of selections performed against PI3K. The total counts (**TCs**) for each replicate are respectively: **a)** TCs = 361’245, **b)** TCs = 350’839 **c)** TCs = 342’638.

6.4.7. Albumin fingerprints

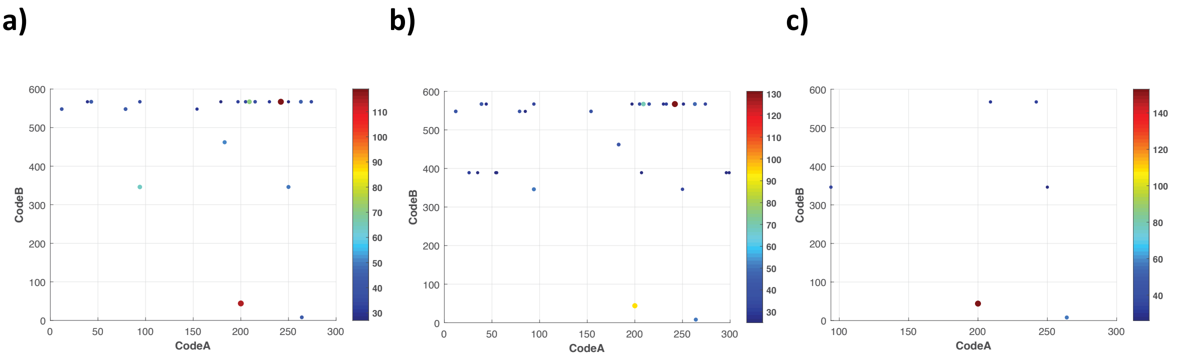

Supplementary figure 30: Fingerprint of selections performed against human serum albumin (HSA). The total counts (**TCs**) for each replicate are: **a)** TCs = 190’200, **b)** TCs = 245’600 **c)** TCs = 178’700.

6.5. Enrichment factors calculation

Definitions

(1) $$TCs = \sum_{i=1}^{300} \sum_{j=1}^{611} SCs(codeAi, codeBj)$$

(2) $$ACs = \frac{TCs}{300\ X\ 611}$$

(3) $$EFi, j = \frac{SCs(CodeAi, CodeBj)}{ACs}$$

Equation 1: Definition of the total counts (**TC**) for a given selection **s**, where i and j define the number of diversity elements A and B and SC corresponds to sequence counts. **Equation 2:** definition of average counts (AC) in a given selection **s. Equation 3:** Definition of the enrichment factor (EF) for the *i*-th, *j*-th combination of building blocks A and B.

Supplementary table 5: Single pharmacophore selections hits enrichment factor calculation. SCs, sequence counts and EF, enrichment factor.

Selection	Selected HIT	Sel. 1 SCs.	Sel. 1 EF	Sel. 2 SCs.	Sel. 2 EF	Sel.3 SCs.	Sel.3 EF	σ	EF (av.)
Streptavidin	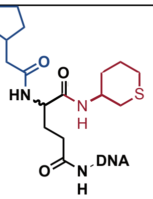 4;5	60	47	42	30	59	48	10	42±10
CAIX	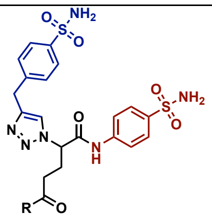 A94/B575	1319	834	1290	985	n/a	n/a	n/a	946
	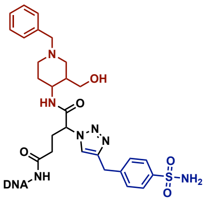 A156/B575	1397	879	1386	1058	n/a	n/a	n/a	969
FAN-1(D960A)	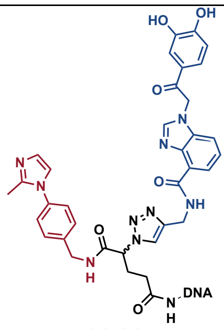 6;7	536	571	687	636	n/a	n/a	n/a	604
	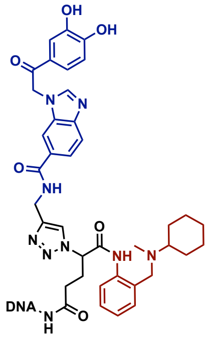 A46/B611	437	465	380	352	n/a	n/a	n/a	409

FAN-1(D960A	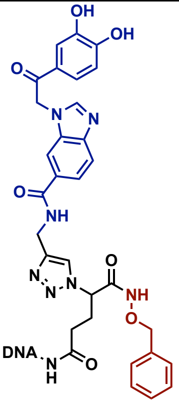 **A91/B611**	359	382	387	359	n/a	n/a	n/a	371
	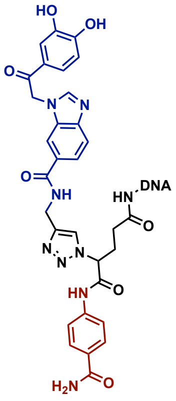 **A262/B611**	523	556	485	449	n/a	n/a	n/a	503
AASSA	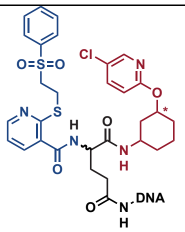 **8;9**	41	21	197	124	155	98	54	81±54
PI3K	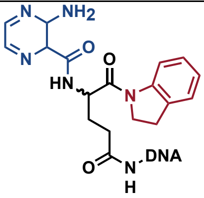 **10;11**	39	20	45	24	52	28	4	24±4
	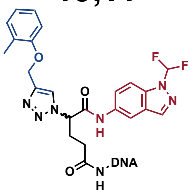 **12;13**	83	42	82	43	84	45	1	43±1
	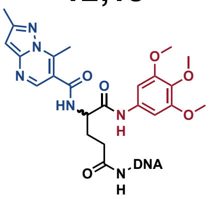 **14;15**	30	15	46	24	48	26	6	22±6

**HSA**	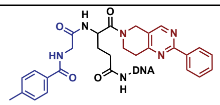 **16;17**	114	110	94	70	153	157	43	112±43

7. Nuclease Activity assay

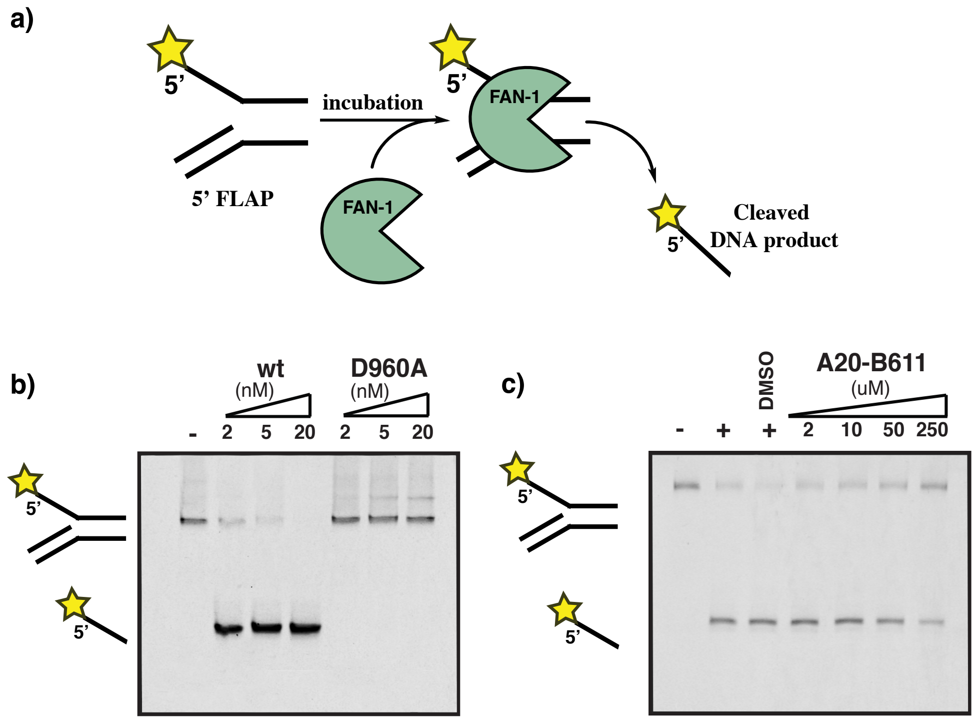

Supplementary figure 31: a) Schematic representation of FAN-1 nuclease activity assay. The fluorescently labelled **5'FLAP** substrate is cleaved by FAN-1 to yield a **cleaved DNA fragment**. **b)** SDS-PAGE of **wt** and **D960A FAN-1** incubated with **5'FLAP**. The nuclease activity is abrogated in the **D960A FAN-1** wells at three different protein concentration (**2, 5, 20 nM**) as shown by the presence of reaction starting material (**5'FLAP**). Incubation of **5'FLAP** with **wt FAN-1** at three different protein concentrations (**2, 5, 20 nM**) yields the **cleaved DNA fragment**; **c)** SDS-PAGE of nuclease activity competition assay. Compound **6** was incubated at 2, 10, 50, 250 uM concentrations (**A20-B611** wells) with **wt FAN-1**; **+ DMSO** = DMSO control, **+** = no ligand control and **-** = no protein control.

8. Selection experiments with affinity maturation dual pharmacophore 2+1 DELs

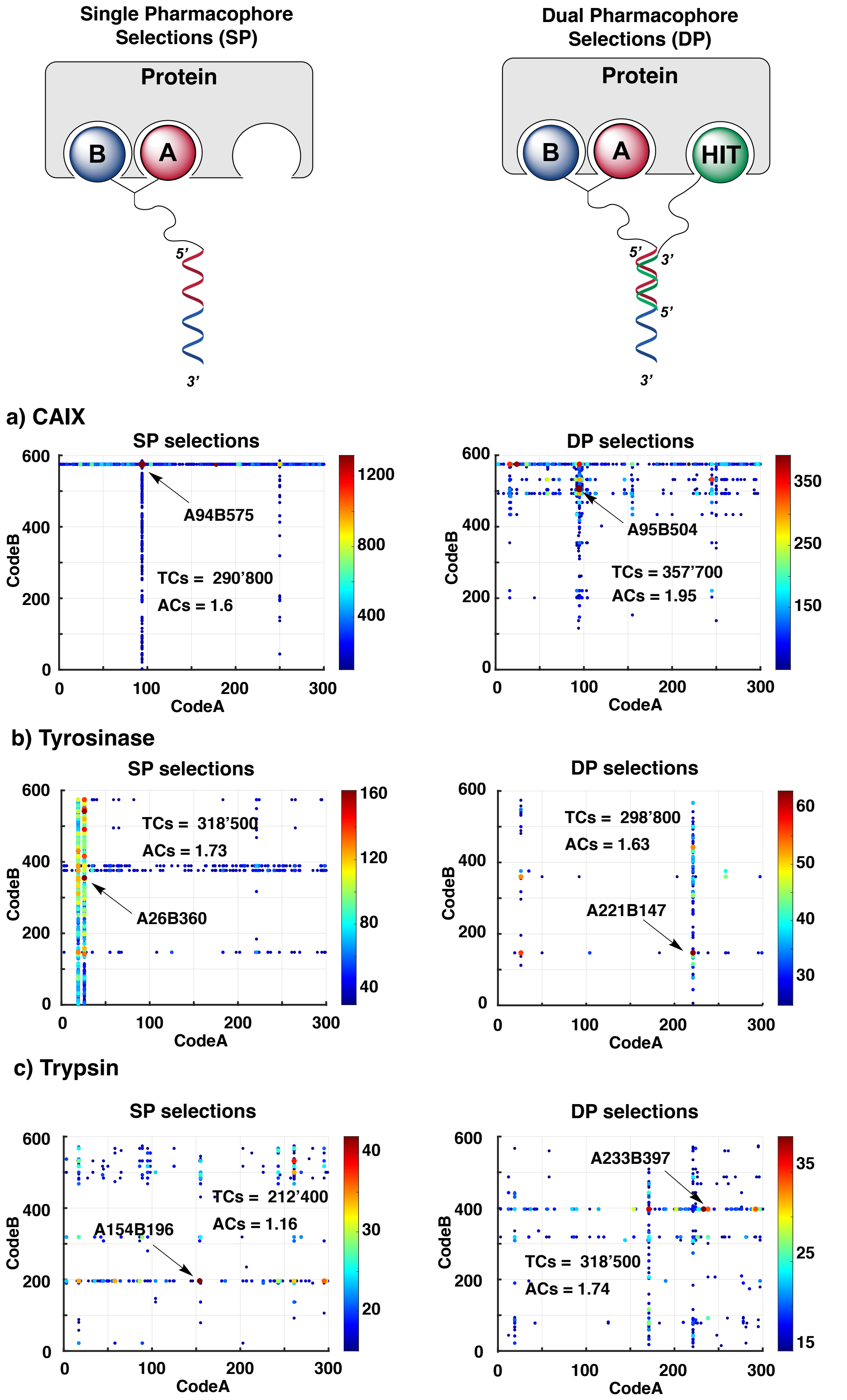

Supplementary figure 32: Left panels show selection fingerprints in single pharmacophore format of Glu-DEL against CAIX, tyrosinase and trypsin, respectively. Right panels show CAIX, tyrosinase and trypsin selection in dual pharmacophore 2+1 affinity maturation format. Indicated by the arrows are the most enriched **CodeA/CodeB** combinations in each selection experiment. **TCs** and **ACs** values used for enrichment factor calculations of selected dual pharmacophore hits (**supplementary table** 6) are given inside the HTDS plots.

Supplementary table 6: Enrichment factor comparison of single and dual pharmacophore hit compounds.

Selection		Selected compound	Counts (SP)	Counts (DP)	EF (SP)	EF (DP)
**CREBBP**						
	**SP**	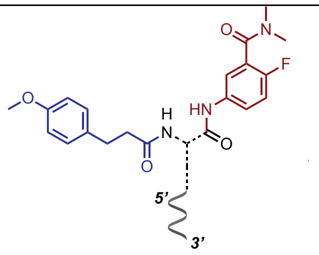 **A219/B106**	4	-	3	-
	**DP**	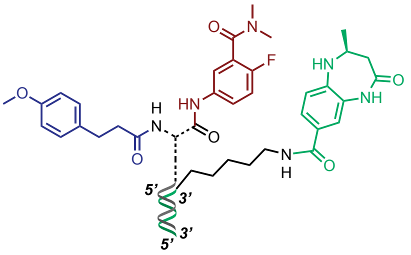 **A219/B106-HIT-1**	-	39	-	22
**HSA**						
	**SP**	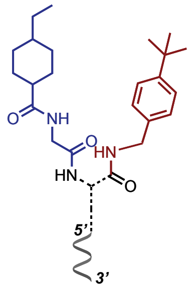 **A24/B137**	3	-	3	-
	**DP**	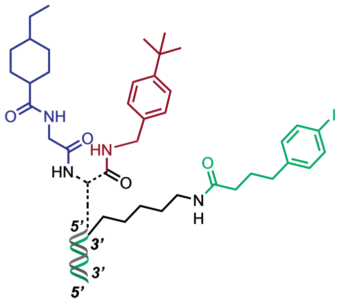 **A24/B137-HIT-2**	-	1476	-	1119

**CAIX**						
	**SP**	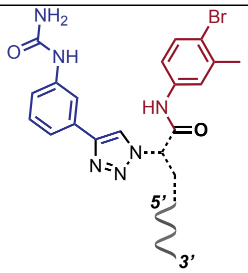<br>A95/B504	1	-	0.5	-
	**DP**	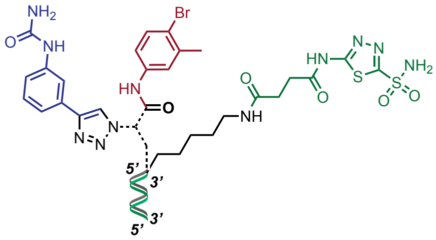<br>A95/B504-HIT3	-	395	-	202
**Tyrosinase**						
	**SP**	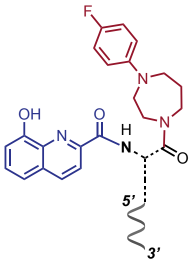<br>A221/B147	64	-	37	-
	**DP**	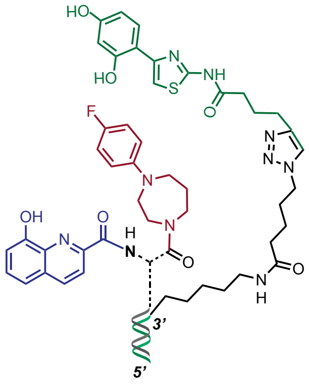<br>A221/B147-HIT4	-	63	-	38

**Trypsin**						
	**SP**	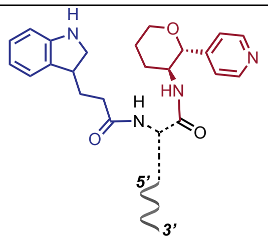 A233/B397	1	-	0.9	-
	**DP**	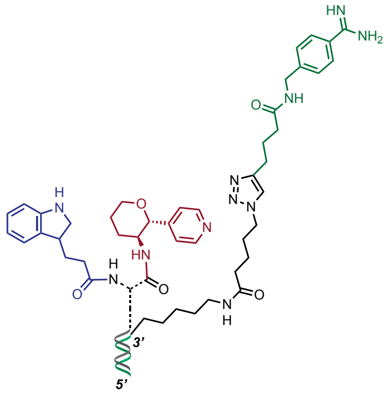 A233/B397-HIT5	-	39	-	23

9. Single pharmacophore library: re-synthesis of selection hits

9.1. Solid phase synthesis

9.1.1. General procedure of Fmoc Deprotection

The Fmoc protecting group was removed by incubating the resin with piperidine:DMF = 1:4 solution (1x30 minutes, 2x10 minutes). The resin was washed several times with DMF.

9.1.2. General procedure of amino-acid loading

The "free-amino" resin (1 eq.) was incubated for 4 hours with a solution of protected amino acid (4 eq.), O-(7-Azabenzotriazol-1-yl)-N,N,N',N'-tetramethyluronium hexafluorophosphate (HATU, 4 eq.), DIPEA (8 eq.) in dry DMF. The reaction was quenched by washing the resin several times with DMF.

9.1.3. TNBS test

The TNBS (2,4,6-trinitrobenzenesulfonic acid) test was used for detecting primary amino groups. The beads turned orange-red in presence of free primary amino group. An aliquot of the resin was poured in a of 2,4,6-trinitrobenzenesulfonic acid solution (DMF:DIPEA=9:1) and incubated for 5 minutes.

9.1.4. General procedure of amide coupling

The "free-amino" resin (1 eq.) was incubated overnight with a solution of activated carboxylic acid (2 eq.). The carboxylic acids were activated by HATU (2 eq.) - DIPEA (4 eq.) procedure. The coupling reaction was quenched by washing the resin several times with DMF.

9.1.5. General procedure of diazo-transfer.

The “free-amino” resin was swollen in DMSO and subsequently incubated for 1 hour with 1H-imidazole-1-sulfonyl azide hydrochloride (3 eq.), DIPEA (9 eq.) in dry DMSO. The resin was then washed with DMF.

9.1.6. General procedure of CuAAC.

The “free-amino” resin was incubated overnight with a solution of alkyne (1.5 eq.), copper iodide (0.2 eq.) and Tris(benzyltriazolylmethyl)amine (TBTA, 0.25 eq.) in degassed DMF:TEA = 9:1. The resin was washed with 0.5M EDTA solution and several times with DMF.

9.1.7. General procedure of Alloc protective group deprotection

The resin (1 eq.) was swollen in DCM for 30 minutes. $Pd(Ph_3P)_4$ was added and $PhSiH_3$ (10 eq.) was used as scavenger. The resin was incubated for two hours at room temperature until reaction completion. In order to remove all the reagents, five washing steps in DCM were needed.

9.1.8. General procedure of resin cleavage.

The resin was incubated for 1 hour with a solution of trifluoroacetic acid : water : triisopropylsilane = 95:2.5:2.5 (20 mL/g). The cleavage solution was poured in cold diethyl ether (5 volumes) and the cleaved product was precipitated for 1 hour at -20°C. The pellet was centrifuged for 20 minutes and the supernatant was discarded. The crude product was dissolved in a water : acetonitrile mixture and purified by RP chromatography.

9.1.9. Reverse amide bond formation (COOH on resin).

The “free-acid” resin (1 eq.) was incubated for 4 hours with a solution of Fmoc protected amino-acid (4 eq.), O-(7-Azabenzotriazol-1-yl)-N,N,N',N'-tetramethyluronium hexafluorophosphate (HATU, 4 eq.), DIPEA (8 eq.) in dry DMF. The reaction was quenched by washing the resin several times with DMF.

9.2. Linker synthesis

9.2.1. Synthesis of Resin-(S)-FmocNH-glu(OtBu)-COOAllyl R2

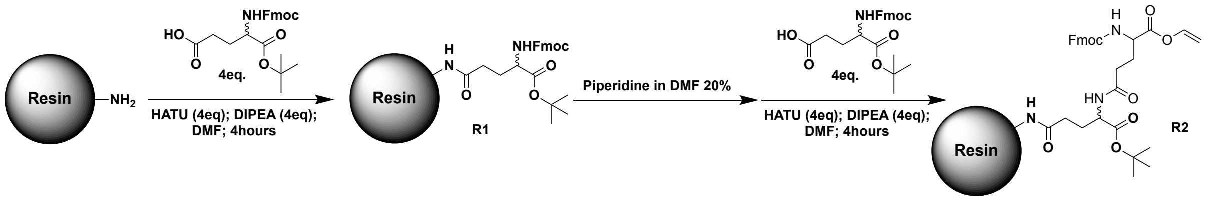

Supplementary figure 33: Solid phase peptide synthesis (SPPS) of R2.

The linker **R2** was assembled on H-Rink amide ChemMatrix® resin (Sigma-Aldrich, 35-100 mesh, wet sieved) using the general procedures (**9.1.1** and **9.1.2**) with the following aminoacid order of addition: Fmoc-L-glutamic acid alpha-tert-butyl ester, Fmoc-R/S-glutamic acid alpha-allyl ester.

9.2.2. Synthesis of FITC-labelled negative control (Fluo-NH_2)

Synthesis and characterization of the fluorescent linker used for the small molecule labelling was previously described by Favalli et al.[13] A schematic representation of the process is indicated in **supplementary figure 34.**

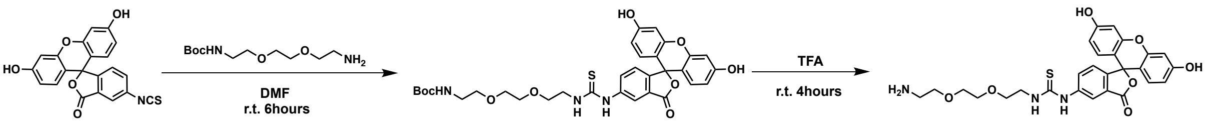

Supplementary figure 34: Schematic representation of the negative control fluorescent linker used for small molecule conjugation

9.3. Synthesis of streptavidin binder: (S)-COOH-Glu/(R,S)-Glu-A8/B264

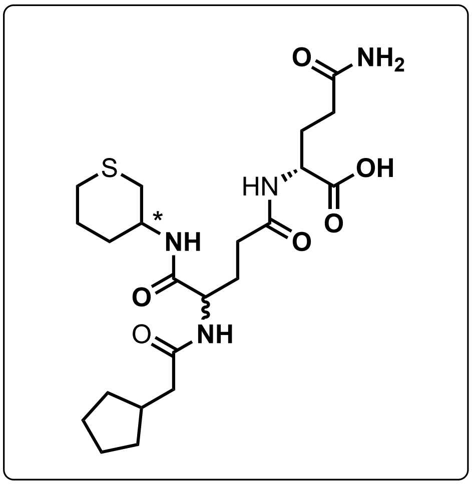

(*R*)-Glu-A8/B264 Compound **4**
(S)-Glu-A8/B264 Compound **5**

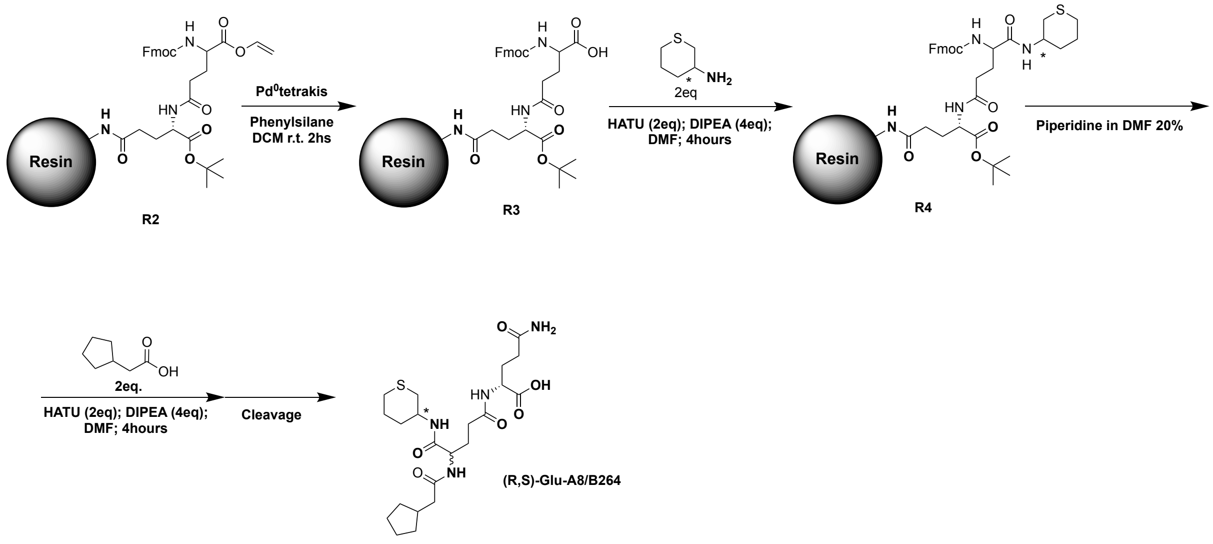

Supplementary figure 35: Synthetic strategy used for the synthesis of Streptavidin ligands (compounds **4, 5**).

The allyl ester of **R2** was removed using the protocol described in section **9.1.7.** Amine A8 was added to **R3** (**9.1.9**) obtaining the intermediate **R4**. After Fmoc deprotection (**9.1.1),** coupling of building block B264 (**9.1.4)** and resin cleavage (**9.1.8**), compounds **4, 5** were isolated with 12% and 15% yield respectively. Compound **4**: white solid, **^{1}H NMR** (600 MHz, DMSO-d6) δ 12.51 (s, 1H), 8.08 (t, J = 8.5 Hz, 1H), 7.94 – 7.83 (m, 2H), 7.29 – 7.25 (m, 1H), 6.77 – 6.72 (m, 1H), 4.19 (tdd, J = 8.2, 6.1, 4.5 Hz, 1H), 4.11 (dddd, J = 9.4, 7.5, 5.1, 2.2 Hz, 1H), 3.72 (tdd, J = 10.5, 7.3, 3.6 Hz, 1H), 3.50 (s, 1H), 2.64 – 2.51 (m, 1H), 2.48 – 2.39 (m, 2H), 2.37 (ddd, J = 12.9, 9.8, 5.5 Hz, 1H), 2.18 – 2.06 (m, 6H), 2.10 – 1.97 (m, 1H), 1.97 – 1.86 (m, 1H), 1.85 – 1.51 (m, 9H), 1.55 – 1.40 (m, 2H), 1.33 (dtt, J = 17.1, 11.3, 3.5 Hz, 1H), 1.16 – 1.05 (m, 2H). **^{13}C NMR** (151 MHz, DMSO-d6) δ 174.20, 174.11, 172.46, 172.43, 172.28, 52.88, 52.75, 52.27,

47.74, 47.70, 41.91, 37.36, 32.92, 32.08, 29.64, 28.94, 27.74, 27.64, 27.52, 25.16. **MS (ESI)** m/z calcd. for [C22H36N4O6S]1+: 485.24[M+H]1+, found: 485.2484, calcd. 2*[C22H36N4O6S]1+: 969.48 [2M+H]1+, found: 969.5043 (100%). Compound **5**: white solid, **^{1}H NMR** (600 MHz, DMSO-d6): δ 12.52 (s, 1H), 8.07 (d, J = 7.9 Hz, 1H), 7.93 – 7.83 (m, 2H), 7.28 (s, 1H), 6.75 (s, 1H), 4.22 – 4.09 (m, 2H), 3.72 (dtt, J = 10.5, 7.4, 3.7 Hz, 1H), 2.65 – 2.55 (m, 1H), 2.45 (dd, J = 5.7, 3.5 Hz, 2H), 2.37 (dd, J = 12.9, 9.8 Hz, 1H), 2.15 – 2.06 (m, 7H), 2.10 – 1.98 (m, 1H), 1.95 – 1.88 (m, 1H), 1.85 – 1.72 (m, 1H), 1.75 – 1.68 (m, 1H), 1.71 – 1.65 (m, 1H), 1.68 – 1.60 (m, 1H), 1.59 – 1.52 (m, 1H), 1.47 (td, J = 5.1, 2.0 Hz, 1H), 1.38 – 1.28 (m, 1H), 1.15 – 1.07 (m, 2H). **^{13}C NMR** (151 MHz, DMSO-d6): δ 174.22, 174.11, 172.52, 172.49, 172.26, 52.77, 52.65, 52.26, 52.21, 47.74, 41.92, 32.92, 32.51, 32.07, 32.05, 29.64, 28.84, 27.62, 27.52, 25.16. **MS (ESI)** m/z calcd. for [C22H36N4O6S]1+: 485.24[M+H]1+, found: 485.2490, calcd. 2*[C22H36N4O6S]1+: 969.48 [2M+H]1+, found: 969.5005 (100%).

9.4. Synthesis of FAN-1 binders: (*S*)-COOH-glu/(*R,S*)-A20/B611

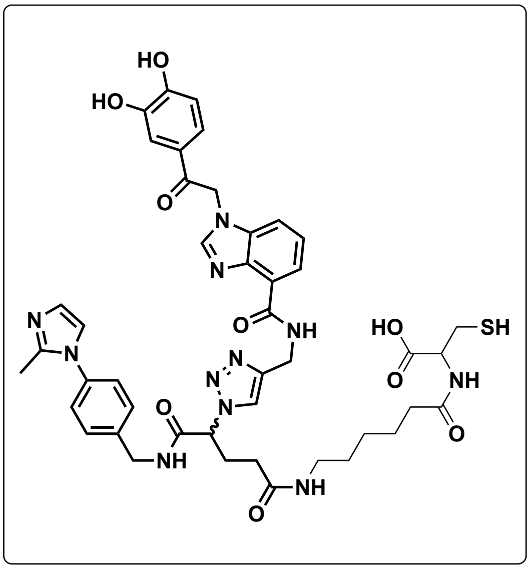

(*R*)-Glu-A20/B611 Compound **6**
(*S*)-Glu-A20/B611 Compound **7**

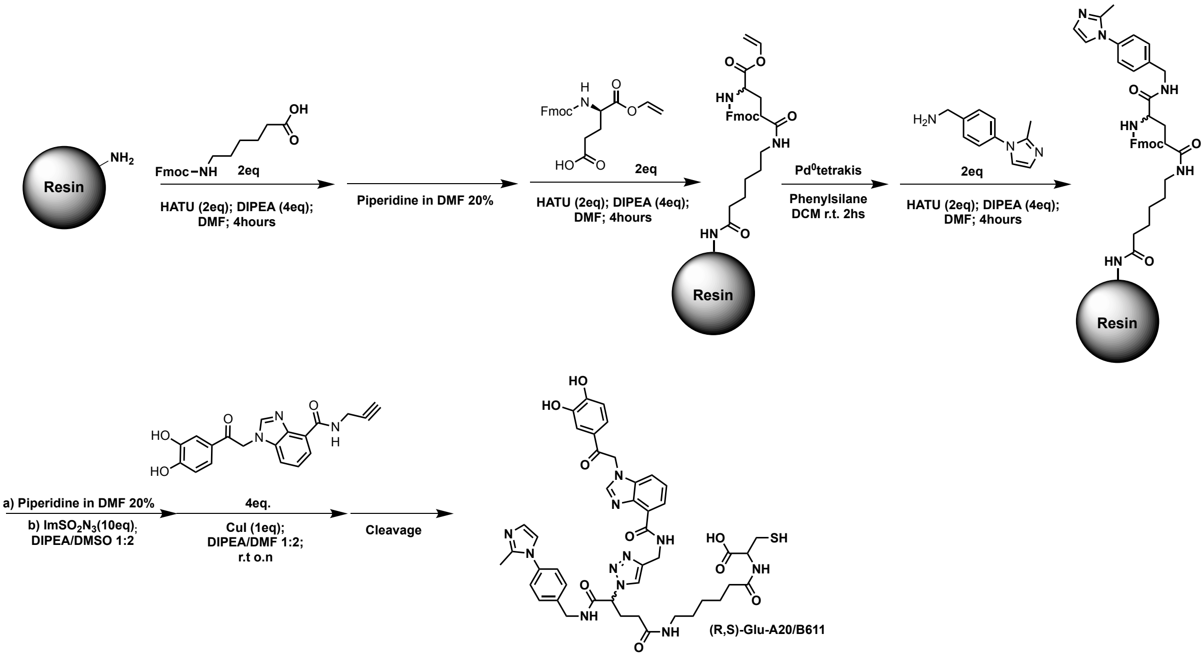

Supplementary figure 36: Synthetic strategy used for the synthesis of FAN1 ligands (compounds **6, 7**).

Wang-Cys-trt resin was swollen in 20% piperidine in DMF for 1 hour to remove Fmoc protective group. After that, 6-((((9H-fluoren-9-yl)methoxy)carbonyl)amino)hexanoic acid was added following the general procedure **9.1.4.** The obtained compound, was treated with Fmoc-R/S-glutamic acid alpha-allyl ester following general the procedure **9.1.4**. Allyl ester was removed using the protocol described in section **9.1.7.** Amine A20 was added to the mixture (**9.1.9**). After Fmoc deprotection (**9.1.1**)**,** diazo transfer (**9.1.5**), coupling of building block B611 (**9.1.4**) and resin cleavage (**9.1.8**), compounds **6, 7** were isolated with 22% and

19% yield respectively. Compound **6**: **^{1}H NMR** (500 MHz, DMSO-d6) δ 10.21 (t, J = 5.7 Hz, 1H), 9.05 (p, J = 7.3, 5.4 Hz, 1H), 8.41 (s, 1H), 8.15 (s, 1H), 8.12 – 8.02 (m, 1H), 7.93 (d, J = 7.4 Hz, 1H), 7.83 – 7.71 (m, 2H), 7.55 (dd, J = 8.3, 2.1 Hz, 1H), 7.44 (d, J = 2.1 Hz, 1H), 7.41 – 7.31 (m, 4H), 7.24 (s, 1H), 6.92 (dd, J = 4.8, 3.5 Hz, 2H), 5.98 (s, 2H), 5.36 (dd, J = 9.2, 6.1 Hz, 1H), 4.76 (h, J = 6.9, 6.3 Hz, 2H), 4.45 (td, J = 8.3, 4.4 Hz, 1H), 4.36 (d, J = 5.8 Hz, 2H), 3.20 (dd, J = 13.6, 4.5 Hz, 1H), 3.14 – 2.68 (m, 5H), 2.65 – 2.52 (m, 1H), 2.47 – 2.21 (m, 5H), 2.19 – 1.87 (m, 5H), 1.54 – 1.13 (m, 7H). **^{13}C NMR** (126 MHz, DMSO) δ 191.37, 172.72, 172.69, 172.29, 170.69, 168.17, 164.91, 163.58, 152.11, 146.28, 146.00, 145.19, 144.07, 140.72, 139.04, 136.75, 135.45, 130.42, 127.28, 127.23, 126.58, 125.61, 123.18, 122.75, 122.73, 122.53, 122.15, 121.40, 115.76, 115.58, 115.39, 115.10, 62.94, 54.80, 51.92, 51.04, 42.35, 35.53, 35.03, 31.56, 29.28, 28.31, 26.48, 26.09, 25.38, 13.90. **MS (ESI)** m/z calcd. for [C44H39N1O9S]2+: 453.6718 [M+H]2+, found: 454.6870 (100%). Compound **7: ^{1}H NMR** (500 MHz, DMSO-d6) δ 10.22 (dd, J = 10.8, 5.9 Hz, 1H), 9.04 (d, J = 16.8 Hz, 1H), 8.56 – 8.33 (m, 1H), 8.16 (s, 1H), 8.05 (s, 1H), 7.94 (d, J = 7.5 Hz, 1H), 7.85 – 7.72 (m, 2H), 7.58 (dd, J = 22.2, 8.2 Hz, 1H), 7.46 – 7.33 (m, 4H), 7.31 – 7.18 (m, 2H), 7.01 – 6.70 (m, 2H), 6.01 (d, J = 11.8 Hz, 2H), 5.37 (dd, J = 9.2, 5.9 Hz, 1H), 4.76 (s, 2H), 4.42 (d, J = 44.9 Hz, 3H), 3.15 – 2.58 (m, 7H), 2.44 – 1.67 (m, 10H), 1.51 – 1.18 (m, 7H). **^{13}C NMR** (126 MHz, DMSO) δ 190.89, 172.28, 172.19, 171.38, 170.21, 167.70, 164.48, 164.42, 157.39, 151.64, 151.47, 145.90, 145.82, 145.54, 144.71, 140.26, 138.54, 135.03, 128.25, 126.96, 126.11, 125.34, 125.13, 122.70, 122.27, 122.06, 121.67, 115.29, 114.93, 114.61, 107.73, 106.74, 62.48, 50.56, 41.89, 38.45, 35.08, 34.58, 31.10, 28.85, 27.84, 26.09, 24.93, 13.50. **MS (ESI)** m/z calcd. for [C44H39N1O9S]2+: 453.6718 [M+H]2+, found: 454.6867 (100%).

9.5. Synthesis of AASSA binders: (*S*)-COOH-glu/(*R,S*)-A120/B235

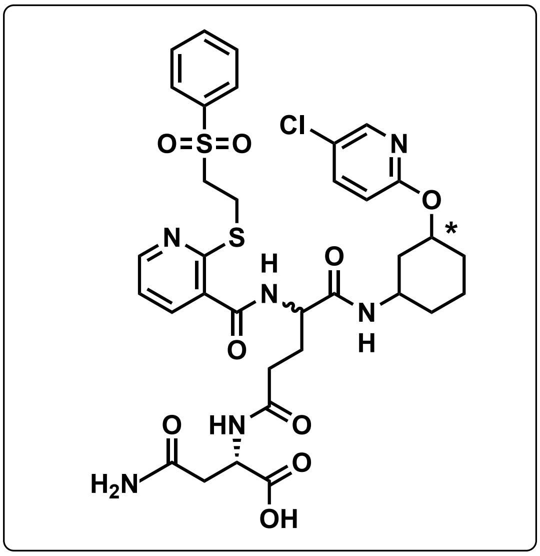

(R)-Glu-A120/B236 Compound **8**
(S)-Glu-A120/B236 Compound **9**

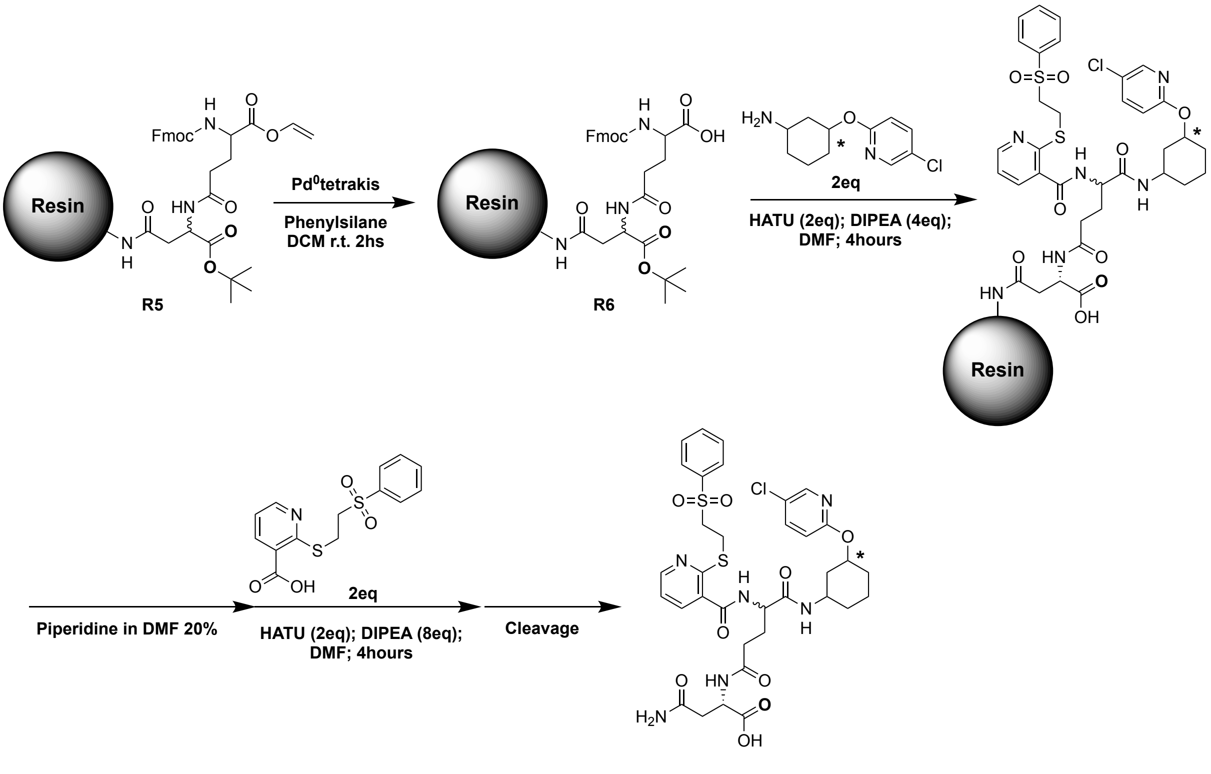

(R,S)-Glu-A120/B235

Supplementary figure 37: Synthetic strategy used for the synthesis of AASS ligands (compounds **8, 9**).

R5 was prepared by swelling H-Rink amide ChemMatrix® resin (Sigma-Aldrich, 35-100 mesh, wet sieved) using the general procedures (**9.1.1** and **9.1.2**) with the following aminoacids order of addition: Fmoc-L-aspartic acid alpha-tert-butyl ester, Fmoc-R/S-glutamic acid alpha-allyl ester.

The allyl ester of **R5** was removed using the protocol described in section **9.1.7.** Amine A120 was added to **R6** (**9.1.9**). After Fmoc deprotection (**9.1.1**), coupling of building block B235 (**9.1.4**) and resin cleavage (**9.1.8**), compounds **8, 9** were isolated with 8% and 11% yield, respectively. Compound **8:** ^{1}H NMR (600 MHz, DMSO-d6) δ 12.62 (s, 1H), 8.79 – 8.63 (m, 1H), 8.28 (dddd, J = 11.4, 6.6, 4.8, 1.8 Hz, 1H), 8.16 – 8.02 (m, 2H), 7.96 – 7.93 (m, 2H), 7.89 (ddd, J = 7.7, 3.8, 1.8 Hz, 1H), 7.79 (dt, J = 7.6, 2.0 Hz, 1H), 7.75 – 7.68 (m, 3H), 7.25 (d, J = 9.1 Hz, 1H), 7.22 – 7.15 (m, 1H), 6.84 – 6.78 (m, 1H), 6.76 (s, 1H), 4.97 (dddq, J = 17.9, 11.0, 7.4, 3.5 Hz, 1H), 4.90 – 4.76 (m, 1H), 4.18 – 4.09 (m, 1H), 3.70 – 3.55 (m, 4H), 3.50 (d, J = 3.1 Hz, 2H), 3.16 – 3.08 (m, 1H), 2.33 – 1.91 (m, 8H), 1.88 – 1.22 (m, 7H). ^{13}C NMR (151 MHz, DMSO) δ 171.50, 169.50, 165.33, 165.14, 160.95, 160.76, 156.62, 156.47, 150.13, 144.68, 139.13, 138.50, 135.97, 133.82, 129.32, 127.76, 123.18, 118.86, 112.65, 69.66, 54.06, 51.48, 48.77, 44.77, 41.80, 39.69, 39.00, 31.25, 28.90, 28.49, 27.40, 27.05, 26.80, 22.34, 21.44. **MS (ESI)** m/z calcd. for [C34H39ClN6O9S2]1+: 775.1908 [M+H]1+, found: 775.1851 (100%). Compound **9**: ^{1}H NMR (600 MHz, DMSO-d6) δ 12.60 (s, 1H), 8.72 (ddd, J = 35.9, 14.9, 8.2 Hz, 2H), 8.32 – 8.24 (m, 2H), 8.18 (dd, J = 4.7, 2.6 Hz, 1H), 8.04 (d, J = 2.8 Hz, 1H), 7.95 (ddt, J = 8.3, 2.6, 1.1 Hz, 3H), 7.91 (ddd, J = 7.7, 3.3, 1.8 Hz, 1H), 7.81 – 7.76 (m, 2H), 7.75 – 7.69 (m, 4H), 7.25 – 7.15 (m, 3H), 6.83 – 6.70 (m, 3H), 4.97 (dddd, J = 25.9, 11.0, 7.1, 3.5 Hz, 2H), 4.85 (ddd, J = 12.8, 8.8, 4.7 Hz, 1H), 4.77 (td, J = 8.5, 4.7 Hz, 1H), 4.12 (ddd, J = 33.5, 13.4, 8.0 Hz, 2H), 3.94 – 3.85 (m, 1H), 3.65 – 3.53 (m, 5H), 3.25 – 3.05 (m, 4H), 2.31 (dt, J = 15.5, 7.8 Hz, 1H), 2.25 – 2.18 (m, 2H), 2.16 (t, J = 7.7 Hz, 1H), 2.08 (dqd, J = 12.8, 8.1, 5.5, 4.6 Hz, 3H), 2.02 – 1.86 (m, 4H), 1.79 (dt, J = 15.1, 7.3 Hz, 3H), 1.73 – 1.62 (m, 3H), 1.59 – 1.49 (m, 1H), 1.49 – 1.32 (m, 3H). ^{13}C NMR (151 MHz, DMSO) δ 173.36, 173.33, 171.37, 171.32, 169.69, 169.56, 169.49, 169.37, 165.47, 165.22, 165.18, 160.92, 160.74, 156.61, 156.43, 150.13, 144.67, 144.60, 139.12, 138.50, 136.00, 133.86, 129.34, 128.27, 127.75, 123.18, 118.86, 118.76, 112.85, 112.66, 112.60, 69.66, 69.32, 69.07, 68.80, 54.04, 51.49, 41.76, 31.29, 31.17, 31.12, 30.87, 30.80, 28.93, 28.78, 28.46, 28.24, 27.50, 27.23, 26.98, 26.88, 26.84, 26.78, 22.55, 22.40, 22.26, 21.40, 21.30. **MS (ESI)** m/z calcd. for [C34H39ClN6O9S2]1+: 775.1908 [M+H]1+, found: 775.1722 (100%).

9.6. Synthesis of wt-PI3K binders

9.6.1. Synthesis of (S)-COOH-glu/(R,S)-A86/B371

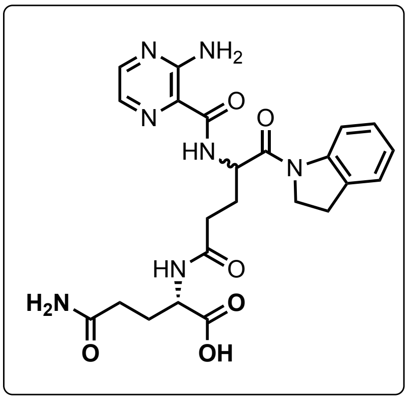

(**R**)-**Glu-A86/B371** Compound **10**
(**S**)-**Glu-A86/B371** Compound **11**

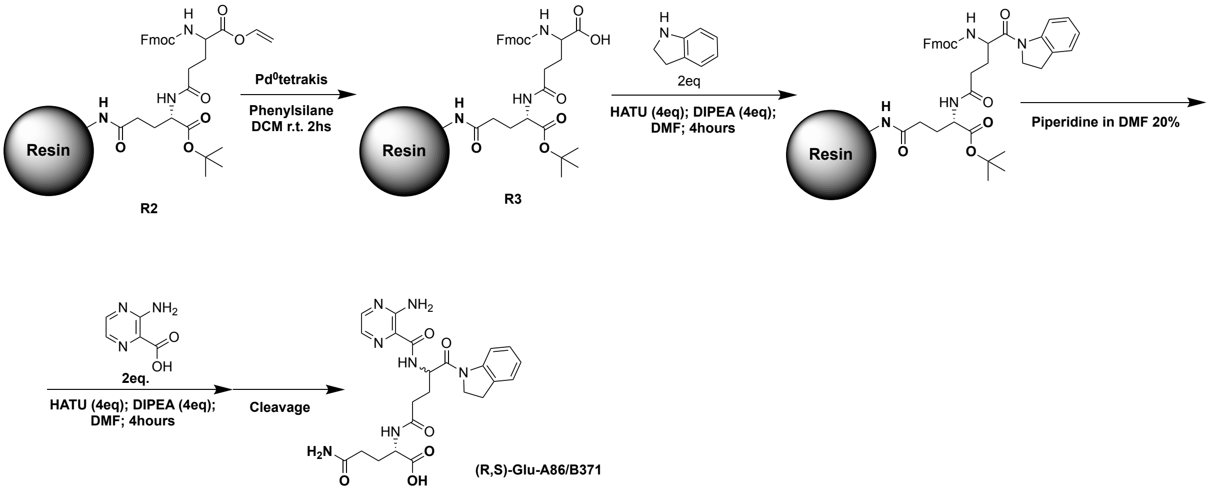

Supplementary figure 38: Synthetic strategy used for the synthesis of PI3K ligands (compounds **10, 11**).

The allyl ester of **R2** was removed using the protocol described in section **9.1.7.** Amine A86 was added to **R3** (**9.1.9**). After Fmoc deprotection (**9.1.1**), coupling of building block B371 (supplementary info **9.1.4**) and resin cleavage (supplementary info **9.1.8**), compounds **10, 11** were isolated with 7% and 12% respectively. Compound **10:** ^{1}**H NMR** (600 MHz, DMSO-d6) δ 8.75 (d, J = 7.7 Hz, 1H), 8.25 (d, J = 2.4 Hz, 1H), 8.15 (d, J = 7.7 Hz, 1H), 8.08 (d, J = 8.0 Hz, 1H), 7.88 (d, J = 2.3 Hz, 1H), 7.50 (s, 1H), 7.30 – 7.22 (m, 2H), 7.19 – 7.13 (m, 1H), 7.03 (td, J = 7.4, 1.1 Hz, 1H), 6.74 (s, 1H), 4.83 (td, J = 8.2, 4.4 Hz, 1H), 4.28 (qt, J = 10.4, 5.2 Hz, 2H), 4.13 (ddd, J = 8.9, 7.6, 5.1 Hz, 1H), 3.51 (s, 1H), 3.20 (t, J = 8.5 Hz, 3H), 2.33 – 2.24 (m, 2H), 2.16 – 1.96 (m, 4H), 1.91 (tdd, J = 8.9, 7.7, 5.1 Hz, 1H), 1.71 (dtd, J = 13.5, 8.7, 6.5 Hz, 1H). ^{13}**C NMR** (151

MHz, DMSO) δ 173.35, 173.27, 171.41, 169.49, 165.66, 155.02, 147.16, 142.52, 132.03, 130.98, 126.84, 124.80, 124.77, 123.71, 116.29, 69.67, 51.52, 50.99, 47.32, 31.25, 30.87, 27.45, 26.81. **MS (ESI)** m/z calcd. for [C23H29N7O6]1+: 498.2179 [M+H]1+, found: 498.2062, calcd. 2*[C23H29N7O6]1+: 995.2178. [2M+H]1+, found: 995.3952 (100%). Compound **11** white solid, **^{1}H NMR** (600 MHz, DMSO-d6) δ 8.74 (d, J = 7.9 Hz, 1H), 8.24 (d, J = 2.4 Hz, 1H), 8.14 (d, J = 7.7 Hz, 1H), 8.08 (d, J = 8.0 Hz, 1H), 7.87 (d, J = 2.3 Hz, 1H), 7.49 (s, 2H), 7.29 – 7.22 (m, 2H), 7.19 – 7.13 (m, 1H), 7.03 (td, J = 7.5, 1.1 Hz, 1H), 6.75 (s, 1H), 4.81 (td, J = 8.5, 4.5 Hz, 1H), 4.32 (td, J = 9.7, 7.3 Hz, 1H), 4.25 (q, J = 9.6 Hz, 1H), 4.15 (ddd, J = 9.1, 7.7, 5.0 Hz, 1H), 3.50 (s, 1H), 3.20 (t, J = 8.7 Hz, 2H), 2.36 – 2.20 (m, 2H), 2.18 – 2.05 (m, 3H), 2.03 – 1.88 (m, 2H), 1.71 (dtd, J = 13.6, 9.0, 6.0 Hz, 1H). **^{13}C NMR** (151 MHz, DMSO-d6) δ 174.32, 174.24, 172.27, 170.46, 166.58, 155.95, 148.08, 143.43, 133.00, 131.90, 127.76, 125.73, 125.70, 124.65, 117.21, 52.37, 51.66, 48.22, 32.14, 31.64, 28.35, 27.81, 27.75. **MS (ESI)** m/z calcd. for [C23H29N7O6]1+: 498.2179 [M+H]1+, found: 498.2065, calcd. 2*[C23H29N7O6]1+: 995.2178. [2M+H]1+, found: 995.3952 (100%).

9.6.2. Synthesis of (*S*)-COOH-glu/(*R,S*)-A146/B537

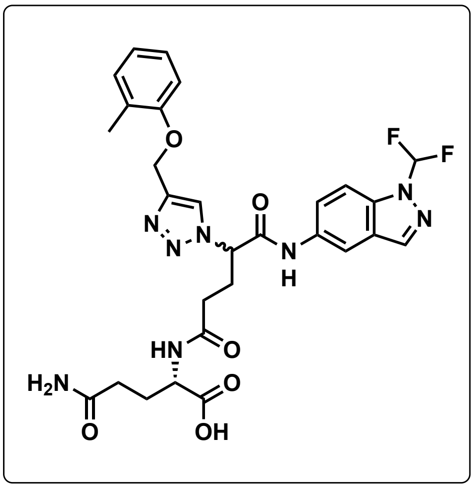

(*R*)-Glu-A146/B537 Compound **12**
(*S*)-Glu-A146/B537 Compound **13**

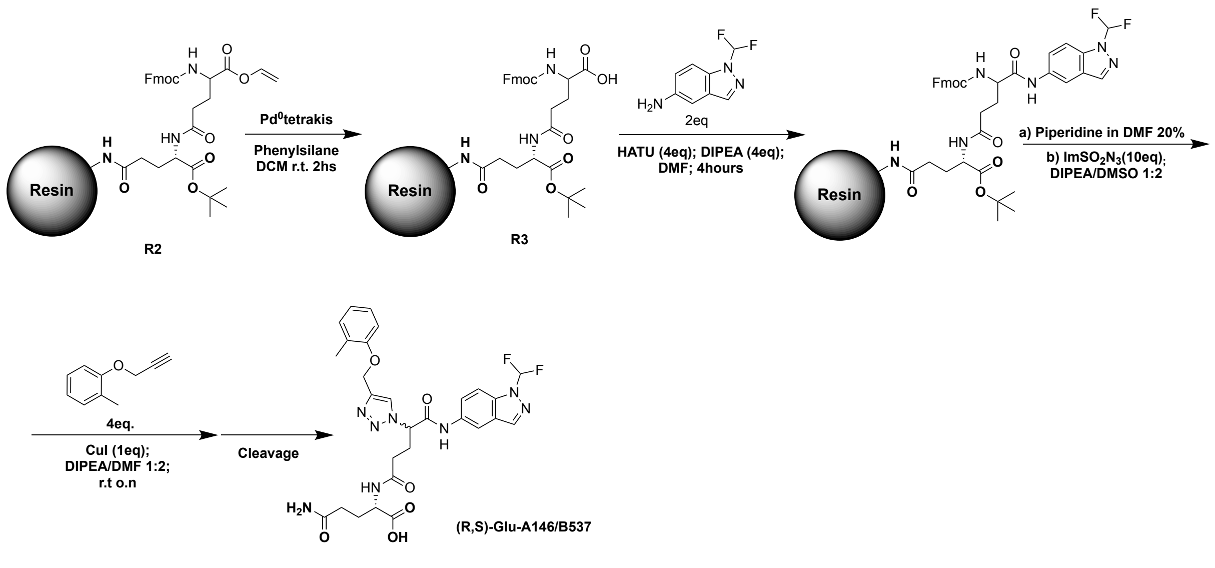

Supplementary figure 39: Synthetic strategy used for the synthesis of PI3K ligands (compounds **12, 13**).

The allyl ester of **R2** was removed using the protocol described in section **9.1.7.** Amine A146 was added to **R3** (**9.1.9**). After Fmoc deprotection (**9.1.1**), coupling of building block B537 (**9.1.4**) and resin cleavage (**9.1.8**), compounds **12, 13** were isolated with 10% and 11% yield, respectively. Compound **12**: ^{1}H NMR (600 MHz, DMSO-d6) δ 10.87 (s, 1H), 8.71 (s, 1H), 8.38 (d, J = 0.9 Hz, 1H), 8.27 (d, J = 1.8 Hz, 1H), 8.14 (d, J = 7.5 Hz, 1H), 7.88 – 7.79 (m, 2H), 7.75 – 7.60 (m, 2H), 7.53 – 7.21 (m, 2H), 6.91 – 6.70 (m, 2H), 5.62 (dd, J = 9.0, 6.4 Hz, 1H), 4.12 (ddd, J = 8.8, 7.6, 5.1 Hz, 1H), 3.95 (s, 4H), 3.51 (s, 1H), 2.54 (d, J = 5.2 Hz, 2H), 2.19 (qdd, J = 15.4, 9.0, 6.3 Hz, 2H), 2.13 – 2.03 (m, 2H), 1.96 – 1.88 (m, 1H), 1.71 (dtd, J = 13.5, 8.8, 6.2 Hz, 1H), 1.20 (d, J = 36.0 Hz, 1H). ^{13}C NMR (151 MHz, DMSO) δ 173.30, 170.45, 166.19, 163.27, 147.26, 147.03, 139.89, 138.06, 134.65, 125.11, 122.49, 122.37, 122.15, 112.20, 111.32, 110.61, 109.64, 69.66, 63.09, 62.93, 52.95, 31.46, 31.27, 30.90, 27.88, 27.66, 26.92, 26.77. **MS (ESI)** m/z calcd. for [C26H29F2N9O6]1+: 600.20 [M+H]1+, found: 600.2103, calcd. 2*[C28H30F2N9O6]1+: 1199.3912 [2M+H]1+, found: 1199.4025 (95%). Compound **13:** ^{1}H NMR (600 MHz, DMSO-d6) δ 12.62 (s, 1H), 10.85 (s, 1H), 8.71 (s, 1H), 8.38 (t, J = 0.8 Hz, 1H), 8.31 – 8.23 (m, 1H), 8.14 (d, J = 8.7 Hz, 1H), 7.84 – 7.76 (m, 2H), 7.73 – 7.62 (m, 2H), 7.48 – 7.24 (m, 2H), 6.81 – 6.69 (m, 2H), 5.61 (ddd, J = 11.9, 8.8, 6.5 Hz, 1H), 4.16 – 4.08 (m, 1H), 4.03 – 3.74 (m, 4H), 3.51 (s, 1H), 2.54 (s, 2H), 2.28 – 2.15 (m, 2H), 2.13 – 2.05 (m, 2H), 1.92 (qd, J = 11.7, 9.9, 6.9 Hz, 1H), 1.75 – 1.66 (m, 1H), 1.20 (d, J = 35.8 Hz, 1H). ^{13}C NMR (151 MHz, DMSO) δ 173.30, 170.45, 166.15, 163.27, 147.26, 147.03, 139.89, 138.06, 134.65, 125.11, 122.49, 122.37, 122.12, 112.20, 111.39, 110.69, 109.64, 69.66, 63.09, 62.93, 52.95, 31.46, 31.23,

30.86, 27.88, 27.66, 26.92, 26.77. **MS (ESI)** m/z calcd. for [C26H29F2N9O6]1+: 600.1956 [M+H]1+, found: 600.1857, calcd. 2*[C28H30F2N8O6]1+: 1199.3912 [2M+H]1+, found: 1199.3752 (85%).

9.6.3. Synthesis of (*S*)-COOH-glu/(*R,S*)-A245/B474

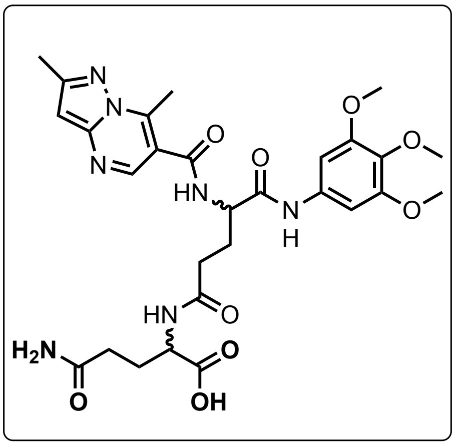

(*R*)-Glu-A245/B474 Compound **14**
(*S*)-Glu-A245/B474 Compound **15**

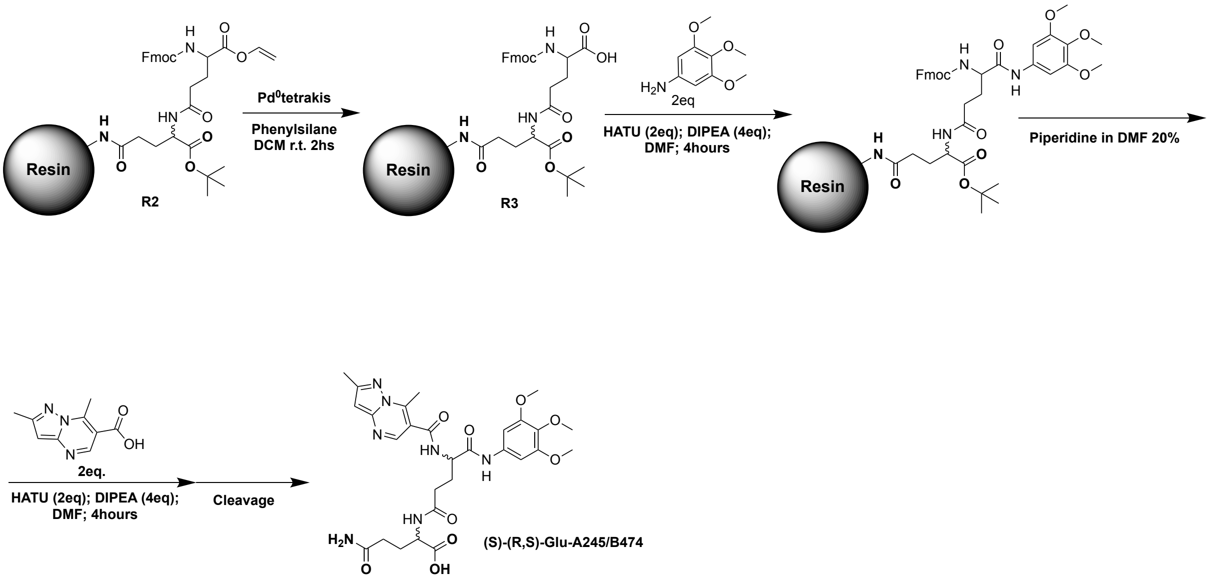

Supplementary figure 40: Synthetic strategy used for the synthesis of PI3K ligands (compounds **14, 15**).

The allyl ester of **R2** was removed using the protocol described in section **9.1.7.** Amine A245 was added to **R3** (**9.1.9**). After Fmoc deprotection (**9.1.1**), coupling of building block B737 (**9.1.4**) and resin cleavage (**9.1.8**), compounds **14, 15** were isolated with 19% and 8% of yield respectively. Compound **14:** ^{1}**H NMR** (600 MHz, DMSO-d6) δ 10.13 (s, 1H), 8.91 (d, J = 7.1 Hz, 1H), 8.55 (s, 1H), 8.17 (d, J = 7.7 Hz, 1H), 7.27 (s, 1H), 7.05 (s, 2H), 6.76 (s, 1H), 6.61 (d, J = 0.7 Hz, 1H), 4.54 (ddd, J = 8.6, 7.1, 5.6 Hz, 1H), 4.15 (ddd, J = 9.0, 7.7, 5.2 Hz, 1H), 3.76 (s, 6H), 3.62

(s, 3H), 3.51 (s, 1H), 2.87 (s, 3H), 2.47 (s, 3H), 2.41 – 2.26 (m, 2H), 2.09 (dddd, J = 19.1, 16.3, 9.0, 4.3 Hz, 3H), 2.02 – 1.89 (m, 2H), 1.74 (dtd, J = 13.6, 8.9, 6.4 Hz, 1H). **^{13}C NMR** (151 MHz, DMSO) δ 173.40, 173.30, 171.32, 169.82, 164.77, 159.29, 155.19, 152.61, 148.21, 147.70, 145.49, 134.98, 133.38, 128.91, 115.67, 96.87, 96.15, 69.67, 60.00, 55.60, 54.19, 51.55, 31.59, 31.30, 27.49, 26.80, 14.57, 14.41. **MS (ESI)** m/z calcd. for [C28H35N7O9]1+: 614.2496 [M+H]1+, found: 614.2440 calcd. 2*[C28H35N7O9]1+: 1227.4992 [2M+H]1+, found: 1227.4839 (95%).Compound **15: ^{1}H NMR** (600 MHz, DMSO-d6) δ 10.14 (s, 1H), 8.90 (d, J = 7.2 Hz, 1H), 8.54 (s, 1H), 8.12 (d, J = 7.8 Hz, 1H), 7.27 (s, 1H), 7.05 (s, 2H), 6.74 (s, 1H), 6.60 (s, 1H), 4.52 – 4.48 (m, 1H), 4.15 (s, 1H), 3.74 (s, 6H), 3.61 (s, 3H), 3.50 (s, 1H), 2.85 (s, 3H), 2.46 (s, 3H), 2.35 (s, 2H), 2.10 (ddd, J = 10.4, 5.5, 2.3 Hz, 5H), 1.72 (dt, J = 6.5, 1.9 Hz, 1H). **^{13}C NMR** (151 MHz, DMSO) δ 173.48, 173.39, 171.25, 169.83, 164.80, 155.24, 155.19, 152.60, 148.21, 147.70, 145.49, 135.00, 133.37, 115.66, 96.90, 96.16, 72.22, 69.66, 60.00, 55.60, 54.10, 51.68, 31.58, 31.35, 27.46, 26.97, 14.57, 14.41. **MS (ESI)** m/z calcd. for [C28H35N7O9]1+: 614.2496 [M+H]1+, found: 614.2521 calcd. 2*[C28H35N7O9]1+: 1227.4992 [2M+H]1+, found: 1227.4816 (95%).

9.7. Synthesis of HSA binder

9.7.1. (S)-COOH-glu/(R,S)-A200/B44

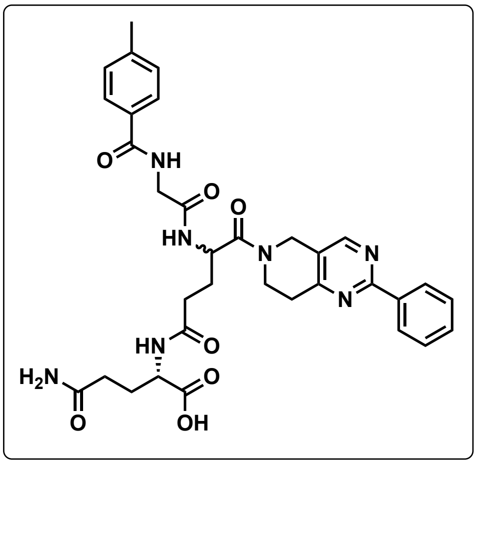

(***R***)**-Glu-A200/B44** Compound **16**
(***S***)**-Glu-A200/B44** Compound **17**

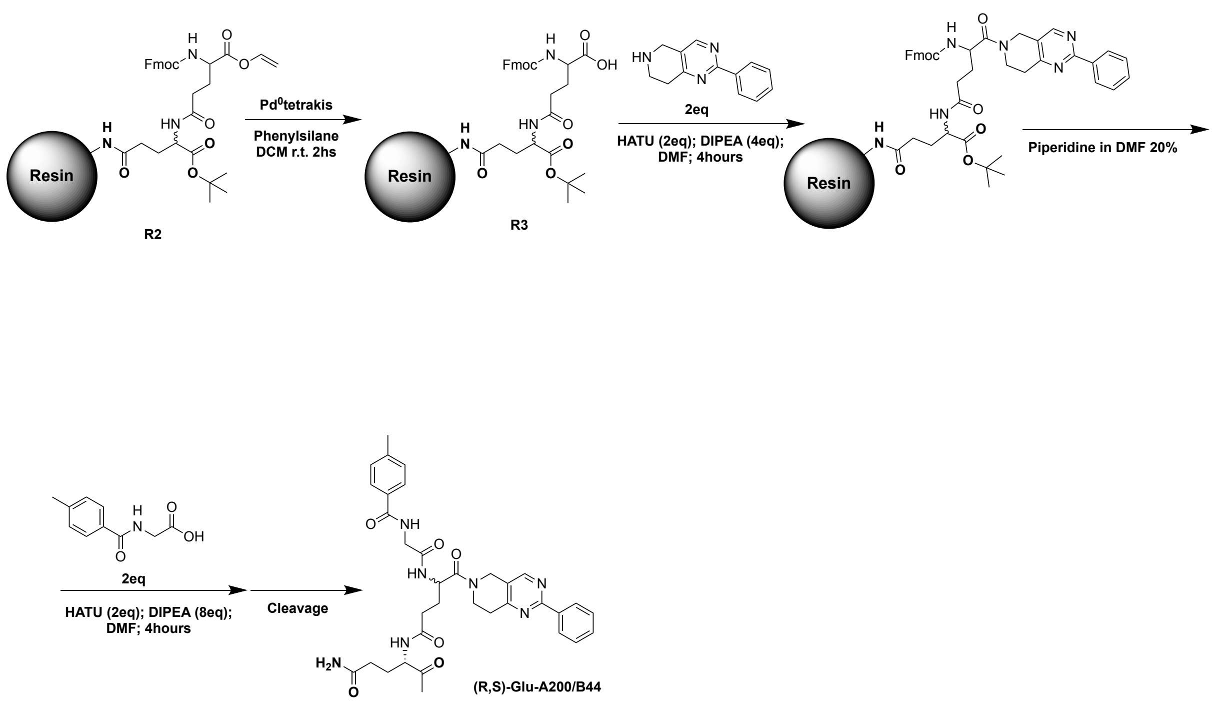

Supplementary figure 41: Synthetic strategy used for the synthesis of PI3K ligands (compounds **16, 17**).

The allyl ester of **R2** was removed using the protocol described in section **9.1.7.** Amine A200 was added to **R3** (**9.1.9**). After Fmoc deprotection (**9.1.1**), coupling of building block B44 (**9.1.4**) and resin cleavage (**9.1.8**), compounds **16** and **17** were isolated with 25% and 17% yields, respectively. Compound **16:** **^{1}H NMR** (600 MHz, DMSO-d6) δ 8.74 (s, 1H), 8.70 – 8.54 (m, 2H), 8.38 – 8.28 (m, 3H), 8.13 – 8.09 (m, 1H), 7.74 (t, J = 7.8 Hz, 2H), 7.51 – 7.48 (m, 3H), 7.24 (d, J = 8.0 Hz, 2H), 6.75 (s, 1H), 4.93 – 4.82 (m, 2H), 4.58 (d, J = 17.2 Hz, 1H), 4.13 (pd, J = 9.3, 7.9, 4.7 Hz, 1H), 3.92 – 3.79 (m, 3H), 3.49 (s, 1H), 3.18 – 2.94 (m, 2H), 2.33 (s, 3H), 2.28 – 2.07 (m, 4H), 2.01 – 1.83 (m, 2H), 1.81 – 1.64 (m, 2H). **^{13}C NMR** (151 MHz, DMSO) δ 173.38, 173.30, 171.64, 170.25, 168.85, 166.24, 163.23, 161.17, 155.42, 141.04, 136.95, 131.12, 130.47, 128.67, 128.53, 127.44, 127.20, 125.25, 125.04, 69.66, 51.51, 48.49, 48.02, 43.31, 42.12, 41.63, 40.84, 31.79, 31.26, 30.86, 27.61, 26.73, 20.84. **MS (ESI)** m/z calcd. for [C34H39N7O6]1+: 644.2962 [M+H]1+, found: 644.2863, calcd. 2*[C34H39N7O6]1+: 1287.5845 [2M+H]1+, found: 1287.5785 (100%). Compound **17:** ^{1}H NMR (600 MHz, DMSO-d6) δ 8.75 (s, 1H), 8.62 (t, J = 5.9 Hz, 1H), 8.39 – 8.32 (m, 3H), 8.06 (d, J = 7.7 Hz, 1H), 7.75 (t, J = 7.9 Hz, 2H), 7.54 – 7.48 (m, 3H), 7.30 – 7.24 (m, 3H), 6.76 (s, 1H), 4.92 – 4.86 (m, 2H), 4.59 (d, J = 17.4 Hz, 1H), 4.16 (tt, J = 8.8, 4.3 Hz, 1H), 3.87 (dddd, J = 22.9, 17.1, 7.9, 3.8 Hz, 3H), 3.51 (s, 1H), 3.03 (ddt, J = 22.8, 17.4, 5.2 Hz, 2H), 2.34 (s, 3H), 2.27 – 2.07 (m, 4H), 1.99 – 1.90

(m, 2H), 1.73 (ddtd, J = 22.9, 13.6, 9.2, 6.1 Hz, 2H). ^{13}C NMR (151 MHz, DMSO) δ 173.40, 171.50, 170.31, 168.92, 166.28, 163.19, 161.17, 155.43, 155.03, 141.05, 136.95, 131.09, 130.47, 128.67, 128.53, 127.44, 127.21, 125.23, 69.67, 51.46, 47.84, 43.29, 42.22, 41.59, 40.83, 31.78, 31.25, 30.84, 30.71, 27.42, 27.29, 26.88, 20.84. **MS (ESI)** m/z calcd. for [C34H39N7O6]1+: 644.2962 [M+H]1+, found: 644.2937, calcd. 2*[C34H39N7O6]1+: 1287.5845 [2M+H]1+, found: 1287.5941 (100%).

10. Dual pharmacophore libraries: re-synthesis of affinity maturation hits

10.1. On-DNA Dual Pharmacophore hits re-synthesis

Hits compounds selected from affinity maturation 2+1 selections were synthetized and validated on DNA-LNA hybrid as shown in **supplementary figure 42.** Two complementary oligonucleotides were used, named: **B12-DNA** (5'-GGATGGCTACTA-3'AmMC6) and **A12-LNA** (3'-CCTACCGATGA(dTfluorescein)-5'AmMC6), respectively.

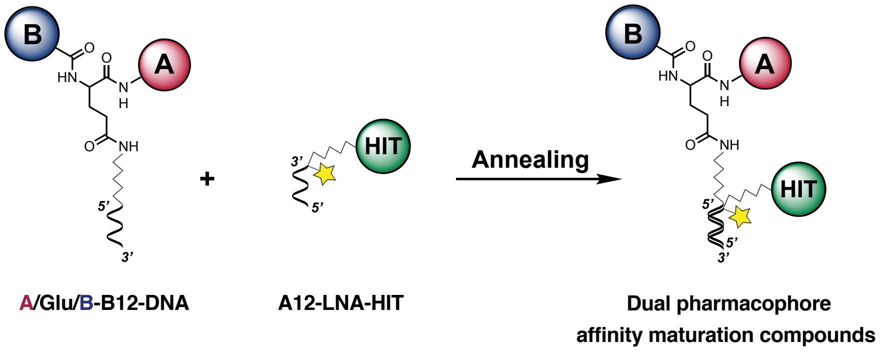

Supplementary figure 42: On-DNA dual pharmacophore affinity maturation hits formation: Hybridization of **A/glu/B-B12DNA** and **A12LNA**HIT conjugates.

10.1.1. CREBBP 2+1 affinity maturation binder

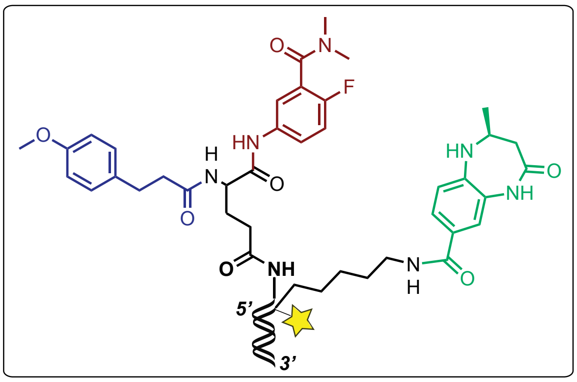

B12DNAA219/B106-A12LNAHIT1

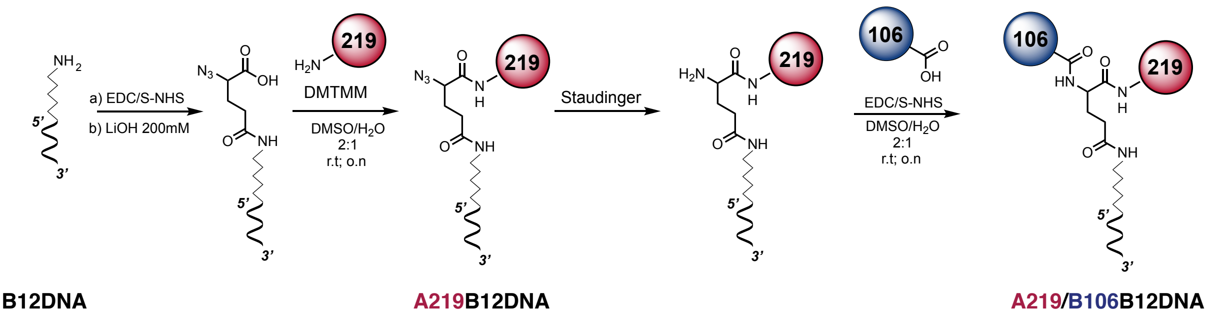

Supplementary figure 43: Synthetic approach for the formation of B12DNAA219/B106 oligo conjugate.

Supplementary figure 43: Synthetic approach for the formation of **B12DNAA219/B106** oligo conjugate.

Library scaffold (Compound **3**) was coupled to **A12DNA** via amide bond following the general procedure, **4.1.1.** The obtained conjugate was treated for methyl ester removal using the procedure described in **4.1.3.** Amine 219 (**A219**) was added using reverse amide bond formation (**4.1.5**). The resulting N_3 derivative was reacted with TCEP (**4.1.6**) followed by coupling with carboxylic acid 106 (**B106**) to afford **B12DNAA219/B106**. A graphical representation of the process is schematized in **supplementary figure 43**. LC-MS was measured after RP HPLC. **MS (ESI)** MW calcd.: 4319.7, found: 4320.

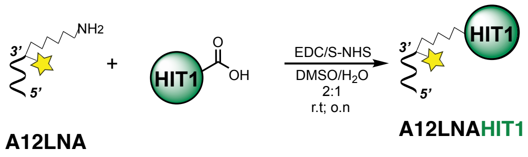

Supplementary figure 44: Synthetic approach for the formation of **A12LNAHIT1** oligo conjugate.

HIT1 was paired to A12LNA via amide bond following, **4.1.1** to obtain **A12LNAHIT1.** A schematic representation of the process is represented in **supplementary figure 44**. LC-MS was measured after RP HPLC. **MS (ESI)** MW calcd.: 4’863.1, found: 4’863. The two oligo conjugates, **B12DNAA219/B106** and **A12LNAHIT1** were annealed by heating at 65°C for 10 min and cooling down at room temperature for 15 minutes to obtain the final product (**B12DNAA219/B106-A12LNAHIT1**).

10.1.2. HSA 2+1 affinity maturation binder

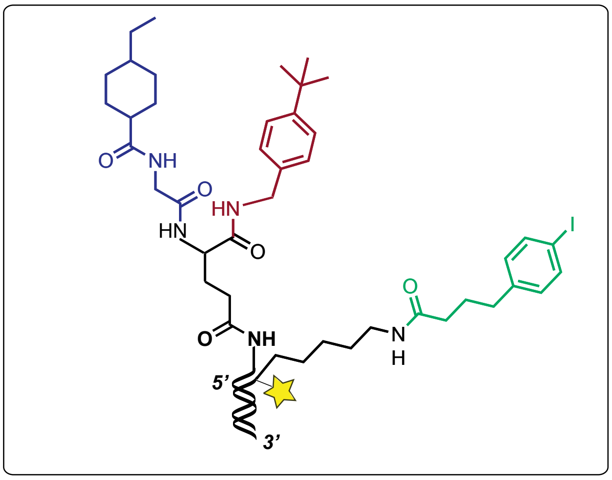

B12DNAA24/B137-A12LNAHIT2

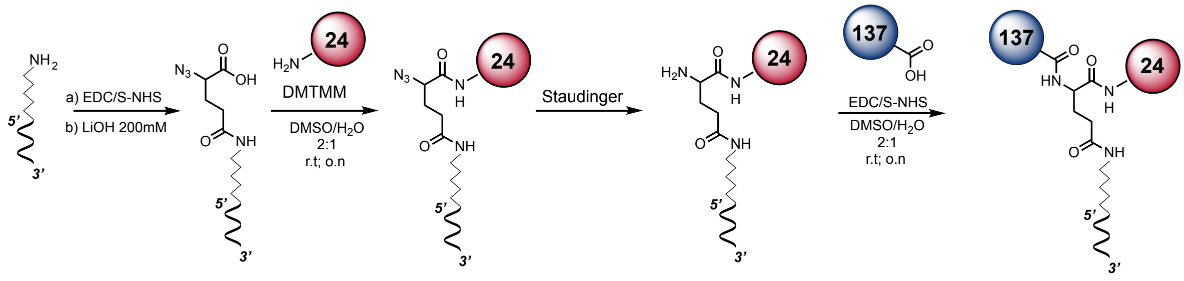

Supplementary figure 45: Synthetic approach for the formation of **B12DNAA24/B137** oligo conjugate.

Library scaffold (Compound **3**) was coupled to **A12DNA** via amide bond following the general procedure **4.1.1.** The obtained conjugate was treated for methyl ester removal using the procedure described in **4.1.3.** Amine 24 (**A24**) was added using reverse amide bond formation (**4.1.5**). The resulting N_3 derivative was reacted with TCEP (**4.1.6**), followed by coupling with carboxylic acid 137 (**B137**) to afford **B12DNAA24/B137**. A schematic representation of the process is represented in **supplementary figure 45**. LC-MS was measured after RP HPLC. **MS (ESI)** MW calcd.: 4333.8, found: 4334.

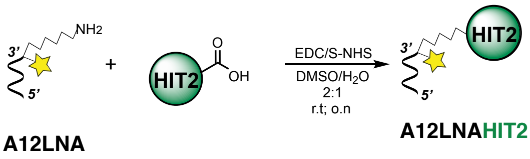

Supplementary figure 46: Synthetic approach for the formation of **A12LNAHIT2** oligo conjugate.

HIT2 was paired to A12LNA via amide bond following, **4.1.1** to obtain **A12LNAHIT2.** A schematic representation of the process is depicted in **supplementary figure 46**. LC-MS was measured after RP HPLC. **MS (ESI)** MW calcd.: 4931.9, found: 4933. The two oligo conjugates previously synthetized, **B12DNAA24/B137** and **A12LNAHIT2**, were annealed by heating at 65°C for 10 min and cooling down at room temperature for 15 minutes to obtain the final product (**B12DNAA24/B137-A12LNAHIT2**).

10.2. Off-DNA dual pharmacophore hits re-synthesis

10.2.1. Synthesis of compound 18 allyl ester

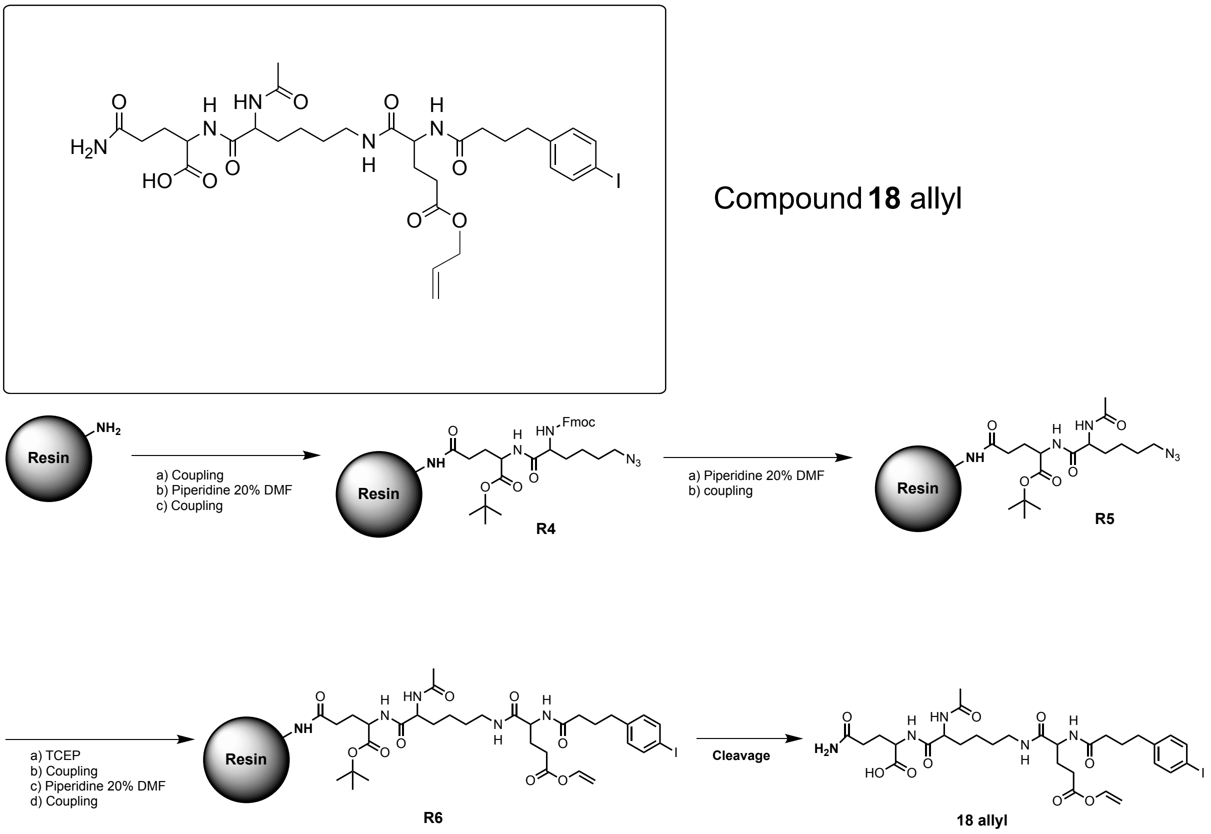

Supplementary figure 47: Synthetic strategy used for the synthesis of HSA ligand control (compound **18**).

Commercially available rink amide resin was coupled to (*S*) Fmoc-Glu(OtBu)-OH following the general procedure, **9.1.2.** The peptide was extend on solid phase by adding (*S*)-Fmoc-Lys(N_3)-OH using procedures **9.1.1** and **9.1.2.** After Fmoc deprotection (**9.1.1**), acetic acid was added according to procedure **9.1.2.** Subsequently, Staudinger reduction was performed (**9.1.5**). After N_3 removal the resin was further elongated with (*S*)-Fmoc-Glu(Alloc)-OH and 4-(4-iodo-phenyl)butanoic acid according to general procedures **9.1.2** and **9.1.1**. The final compound was cleaved from the solid support following the procedure reported in section **9.1.8** and purified by RP HPLC. The product was obtained with 20% yield. Compound **18**: ^{1}H NMR (500 MHz, DMSO-d6) δ 12.54 (s, 1H), 8.15 (d, J = 7.6 Hz, 1H), 7.92 (t, J = 7.6 Hz, 2H), 7.85 (t, J = 5.6 Hz, 1H), 7.67 – 7.52 (m, 2H), 7.29 – 7.20 (m, 1H), 7.07 – 6.94 (m, 2H), 6.75 (s, 1H), 5.88 (ddt, J = 17.3, 10.7, 5.4 Hz, 1H), 5.26 (dq, J = 17.2, 1.7 Hz, 1H), 5.18 (dq, J = 10.5, 1.5 Hz, 1H), 4.51 (dt, J = 5.5, 1.6 Hz, 2H), 4.22 (dtd, J = 16.5, 8.5, 5.3 Hz, 2H), 4.12 (ddd, J = 9.1, 7.6, 5.0 Hz, 1H), 3.50 (s, 1H), 3.07 – 2.86 (m, 2H), 2.31 (dd, J = 8.8, 6.9 Hz, 2H), 2.18 – 2.05 (m, 4H), 1.99 – 1.85 (m, 2H), 1.82 (s, 3H), 1.74 (tdd, J = 12.7, 7.5, 3.4 Hz, 4H), 1.59 (ddt, J = 13.8, 9.3, 5.8 Hz, 1H), 1.51 – 1.18 (m, 6H). ^{13}C NMR (126 MHz, DMSO) δ 173.94, 173.81, 172.44, 172.40, 171.38, 171.34, 169.64, 142.08, 137.42, 133.16, 131.35, 118.15, 91.73, 70.25, 64.86, 52.61, 52.17, 51.97, 40.15, 38.89, 34.89, 34.48, 32.20, 31.76, 30.56, 29.22, 28.08, 27.80, 27.18, 23.06, 22.97. **MS (ESI)** m/z calcd. for [C31H44IN5O9]1+: 758.2263 [M+H]1+, found: 758.2056.

10.2.2. Synthesis of compound 19 allyl ester

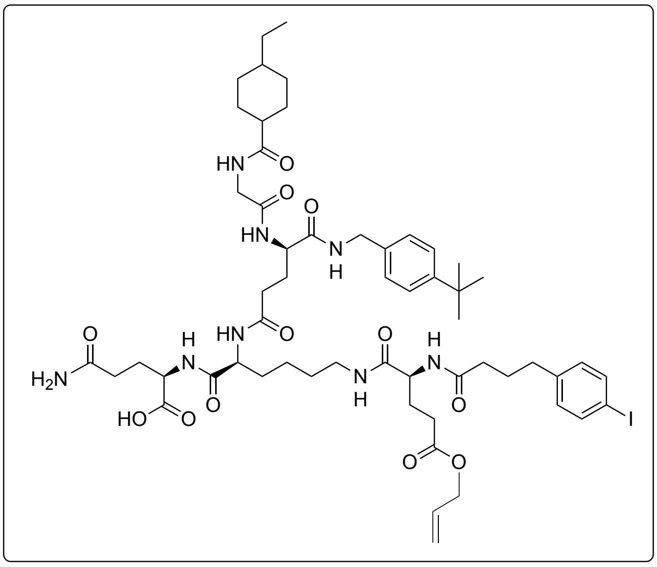

Compound **19** allyl

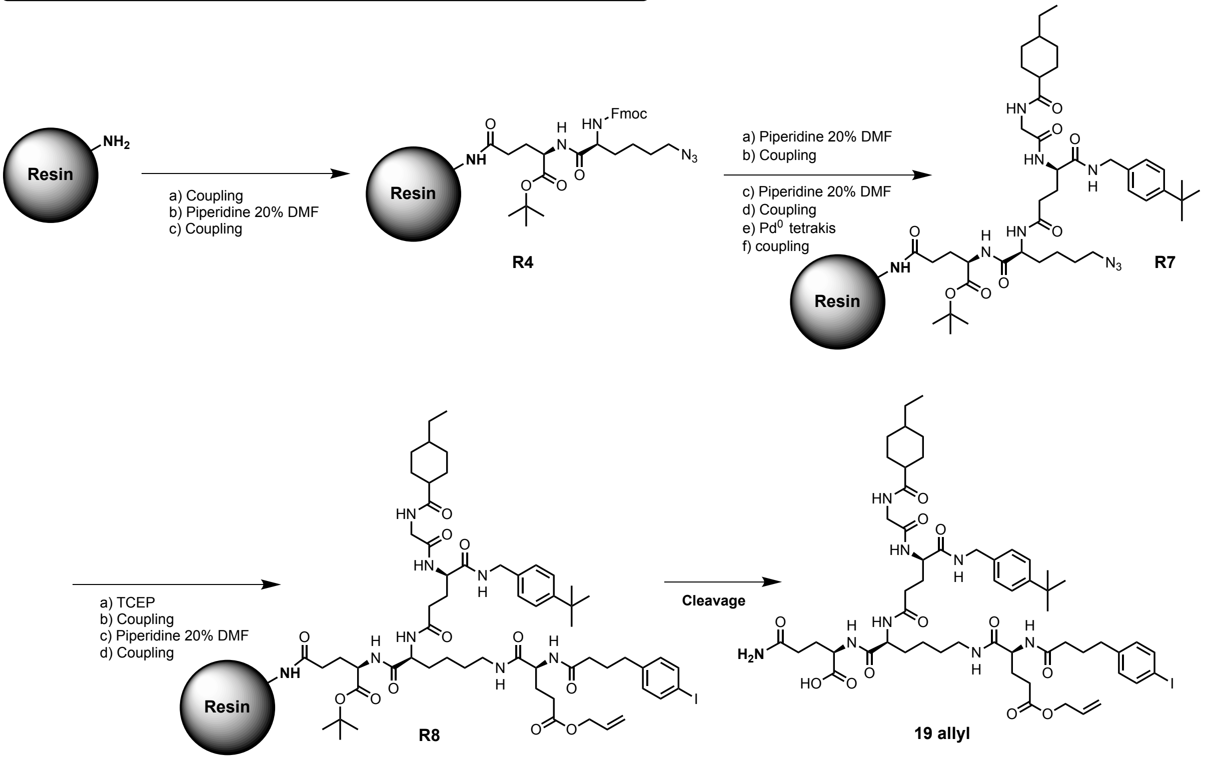

Supplementary figure 48: Synthetic strategy used for the synthesis of HSA affinity matured ligand (compound **19**).

Commercially available rink amide resin was coupled to (*S*) Fmoc-Glu(OtBu)-OH following the general procedure **9.1.2.** The peptide was extend, on solid phase, by adding (*S*)-Fmoc-Lys(N_3)-OH, Fmoc-6-Ahx-OH and (*S*)-Fmoc-Glu(OH)-Alloc using procedures **9.1.1** and **9.1.2.** After that, Alloc protective group was removed according to procedure **9.1.7.** Subsequently, after the addition of A24 and B137 to the solid support according to **9.1.1** and **9.1.2**, Staudinger

reduction was performed (**9.1.5**). After N_3 removal, the resin was further elongated with (*S*)-Fmoc-Glu(Alloc)-OH and 4-(4-iodo-phenyl)butanoic acid according to general procedures **9.1.2** and **9.1.1**. The final compound was cleaved from the solid support following the procedure reported in section **9.1.8** and purified by RP HPLC. The product was obtained with 7% yield. Compound **19 allyl**: ^{1}H NMR (500 MHz, DMSO-d6) δ 12.53 (s, 1H), 8.34 (t, J = 5.9 Hz, 1H), 8.20 – 8.15 (m, 1H), 8.03 – 7.97 (m, 2H), 7.96 – 7.84 (m, 4H), 7.63 – 7.60 (m, 2H), 7.33 – 7.29 (m, 2H), 7.27 – 7.23 (m, 1H), 7.15 (d, J = 8.2 Hz, 2H), 7.02 – 6.99 (m, 2H), 6.79 – 6.74 (m, 1H), 5.89 (ddt, J = 17.2, 10.7, 5.5 Hz, 1H), 5.27 (dq, J = 17.2, 1.6 Hz, 1H), 5.19 (dq, J = 10.5, 1.5 Hz, 1H), 4.52 (dt, J = 5.5, 1.5 Hz, 2H), 4.23 (dtd, J = 19.5, 8.9, 8.0, 5.4 Hz, 7H), 3.72 – 3.65 (m, 2H), 3.11 – 2.94 (m, 4H), 2.31 (dd, J = 8.9, 6.9 Hz, 2H), 2.22 – 2.05 (m, 10H), 1.92 (tdd, J = 21.6, 18.6, 10.1, 4.4 Hz, 5H), 1.81 – 1.68 (m, 12H), 1.25 (s, 9H), 0.87 – 0.79 (m, 6H). ^{13}C NMR (126 MHz, DMSO) δ 176.36, 173.95, 173.83, 173.74, 172.44, 172.40, 171.98, 171.59, 171.55, 171.38, 171.32, 169.62, 149.54, 142.07, 137.42, 136.60, 133.16, 131.34, 129.11, 127.34, 125.42, 118.14, 91.73, 80.88, 70.25, 64.86, 61.79, 52.89, 52.73, 52.63, 52.15, 52.00, 44.38, 42.65, 42.23, 38.96, 38.74, 34.89, 34.59, 34.48, 32.10, 31.77, 31.63, 30.54, 29.90, 29.51, 29.47, 29.26, 28.55, 28.07, 27.81, 27.19, 27.03, 23.12, 14.34, 11.76. **MS (ESI)** m/z calcd. for [C56H81IN8O12]1+: 1185.5098 [M+H]1+, found: 1185.4440.

11. Hit Validation

11.1. Synthesis of fluorescently labelled ligands

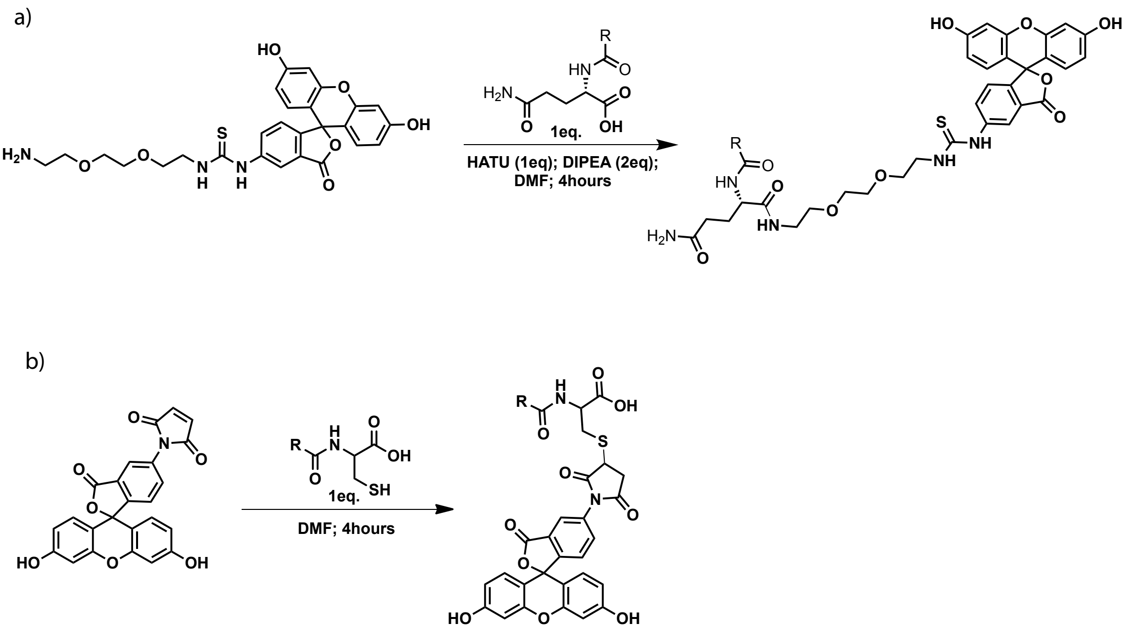

Supplementary figure 49: Schematic representation of the synthetic ¨strategies involved in the formation of fluorescently labelled compounds.

1mg of compounds **4, 5, 8-17** was reacted as represented in **supplementary figure 49,a**. After 4 hours, the reaction were RP HPLC purified and LC-MS was measured. Compounds **6, 7** bearing a terminal cysteine were reacted In a 1 to 1 molar ratio with commercially available 5-maleimido fluorescein by mixing for 4 hours in dry DMF. The resulting mixtures were RP-HPLC purified and LC-MS was measured. Fluorescently labelled compounds **18** and **19** were synthetized according to the scheme in **supplementary figure 49,a** and subsequent Alloc deprotection performed in 1 M NaOH in H_2O/THF 8:2. The resulting mixtures were RP-HPLC purified and LC-MS was measured. Results of the measurements are listed as follows: compound **4 fluo**: **MS (ESI)** m/z calcd. for [C54H69N9O14S2]1+: 1004.3864 [M+H]1+, found:1004.3328. Compound **5 fluo**: **MS (ESI)** m/z calcd. for [C54H69N9O14S2]1+: 1004.3864 [M+H]1+, found:1004.3922. Compound **6 fluo: MS (ESI)** m/z calcd. for [C54H69N9O14S2]1+: , 445.1402 [M+H]3+, found: 445.8141. Compound **7 fluo: MS (ESI)** m/z calcd. for [C54H69N9O14S2]1+: , 445.1402 [M+H]3+, found: 445.4315. Compound **8 fluo: MS (ESI)** m/z calcd. for [C61H64ClN9O15S3]2+: 647.1726 [M+H]2+, found: 647.6506. Compound **9 fluo: MS**

(ESI) m/z calcd. for [C61H64ClN9O15S3]2+: 647.1726 [M+H]2+, found: 647.6506. Compound **10 fluo: MS (ESI)** m/z calcd. for [C50H54N10O12S]1+: 1017.3563 [M+H]1+, found: 1017.3483. Compound **11 fluo: MS (ESI)** m/z calcd. for [C50H54N10O12S]1+: 1017.3563 [M+H]1+, found: 1017.3137. Compound **12 Fluo**: **MS (ESI)** m/z calcd. for [C53H54F2N12O11S]1+: 1119.3420 [M+H]1+, found: 1119.3037. Compound **13 Fluo**: MS (ESI) m/z calcd. for [C53H54F2N12O11S]1+: 1119.3420 [M+H]1+, found: 1119.3106 [C53H54F2N12O11S]2+: 560.15 [M+2H]2+: 560.18 (90%). Compound **14 fluo**: **MS (ESI)** m/z calcd. for [C55H60N10O15S]1+: 1133.3960 [M+H]1+, found: 1133.3748 (90%). Compound **15 fluo**: **MS (ESI)** m/z calcd. for [C55H60N10O15S]1+: 1133.3960 [M+H]1+, found: 1133.3748 (90%). Compound **16 fluo**: **MS (ESI)** m/z calcd. for [C61H64N10O12S]1+: 1163.44 [M+H]1+, found: 1163.4572, m/z calcd. for [C61H64N10O12S]2+: 582.22 [M+2H]2+, found: 582.2443 (98%). Compound **17 fluo**: **MS (ESI)** m/z calcd. for [C61H64N10O12S]1+: 1163.44 [M+H]1+, found: 1163.4540, m/z calcd. for [C61H64N10O12S]2+: 582.22 [M+2H]2+, found: 582.2426 (90%). Compound **18 fluo: MS (ESI)** m/z calcd. for [C55H65IN8O15S]1+: 1237.3414 [M+H]1+, found:1237.2513 (90%). Compound **19 fluo: MS (ESI)** m/z calcd. for [C80H102IN11O18S]1+: 1664.6249 [M+H]1+, found: 1664.5303, m/z calcd. for [C80H102IN11O18S]2+: 832.3124 [M+2H]2+, found: 832.7975 (90%).

12. References

[1] K. S. Midelfort, H. H. Hernandez, S. M. Lippow, B. Tidor, C. L. Drennan, K. D. Wittrup, *J Mol Biol* **2004**, *343*, 685-701.

[2] A. M. Taylor, A. Cote, M. C. Hewitt, R. Pastor, Y. Leblanc, C. G. Nasveschuk, F. A. Romero, T. D. Crawford, N. Cantone, H. Jayaram, J. Setser, J. Murray, M. H. Beresini, G. de Leon Boenig, Z. Chen, A. R. Conery, R. T. Cummings, L. A. Dakin, E. M. Flynn, O. W. Huang, S. Kaufman, P. J. Keller, J. R. Kiefer, T. Lai, Y. Li, J. Liao, W. Liu, H. Lu, E. Pardo, V. Tsui, J. Wang, Y. Wang, Z. Xu, F. Yan, D. Yu, L. Zawadzke, X. Zhu, X. Zhu, R. J. Sims, 3rd, A. G. Cochran, S. Bellon, J. E. Audia, S. Magnuson, B. K. Albrecht, *ACS Med Chem Lett* **2016**, *7*, 531-536.

[3] C. E. Dumelin, S. Trussel, F. Buller, E. Trachsel, F. Bootz, Y. Zhang, L. Mannocci, S. C. Beck, M. Drumea-Mirancea, M. W. Seeliger, C. Baltes, T. Muggler, F. Kranz, M. Rudin, S. Melkko, J. Scheuermann, D. Neri, *Angew Chem Int Ed Engl* **2008**, *47*, 3196-3201.

[4] N. Krall, F. Pretto, W. Decurtins, G. J. Bernardes, C. T. Supuran, D. Neri, *Angew Chem Int Ed Engl* **2014**, *53*, 4231-4235.

[5] T. Mann, W. Gerwat, J. Batzer, K. Eggers, C. Scherner, H. Wenck, F. Stab, V. J. Hearing, K. H. Rohm, L. Kolbe, *J Invest Dermatol* **2018**, *138*, 1601-1608.
[6] L. Mannocci, Y. Zhang, J. Scheuermann, M. Leimbacher, G. De Bellis, E. Rizzi, C. Dumelin, S. Melkko, D. Neri, *Proceedings of the National Academy of Sciences* **2008**, *105*, 17670-17675.
[7] M. Brand, J. Clayton, M. Moroglu, M. Schiedel, S. Picaud, J. P. Bluck, A. Skwarska, A. K. N. Chan, C. M. C. Laurin, A. R. Scorah, K. F. L. See, T. P. C. Rooney, O. Fedorov, G. Perell, P. Kalra, W. A. Cortopassi, K. E. Christensen, R. I. Cooper, R. S. Paton, W. C. K. Pomerantz, P. C. Biggin, E. M. Hammond, P. Filippakopoulos, S. J. Conway, *ChemRxiv.* **2020**, *in press*.
[8] G. Bassi, N. Favalli, S. Oehler, A. Martinelli, M. Catalano, J. Scheuermann, D. Neri, *BBRC* **2020**, *in press*.
[9] W. Decurtins, M. Wichert, R. M. Franzini, F. Buller, M. A. Stravs, Y. Zhang, D. Neri, J. Scheuermann, *Nat Protoc* **2016**, *11*, 764-780.
[10] M. Wichert, N. Krall, W. Decurtins, R. M. Franzini, F. Pretto, P. Schneider, D. Neri, J. Scheuermann, *Nat Chem* **2015**, *7*, 241-249.
[11] T. P. Rooney, P. Filippakopoulos, O. Fedorov, S. Picaud, W. A. Cortopassi, D. A. Hay, S. Martin, A. Tumber, C. M. Rogers, M. Philpott, M. Wang, A. L. Thompson, T. D. Heightman, D. C. Pryde, A. Cook, R. S. Paton, S. Muller, S. Knapp, P. E. Brennan, S. J. Conway, *Angew Chem Int Ed Engl* **2014**, *53*, 6126-6130.
[12] A. Porro, M. Berti, J. Pizzolato, S. Bologna, S. Kaden, A. Saxer, Y. Ma, K. Nagasawa, A. A. Sartori, J. Jiricny, *Nat Commun* **2017**, *8*, 1073.
[13] N. Favalli, S. Biendl, M. Hartmann, J. Piazzi, F. Sladojevich, S. Gräslund, P. J. Brown, K. Näreoja, H. Schüler, J. Scheuermann, R. Franzini, D. Neri, *ChemMedChem* **2018**, *13*, 5.

13. Appendix I: NMR data

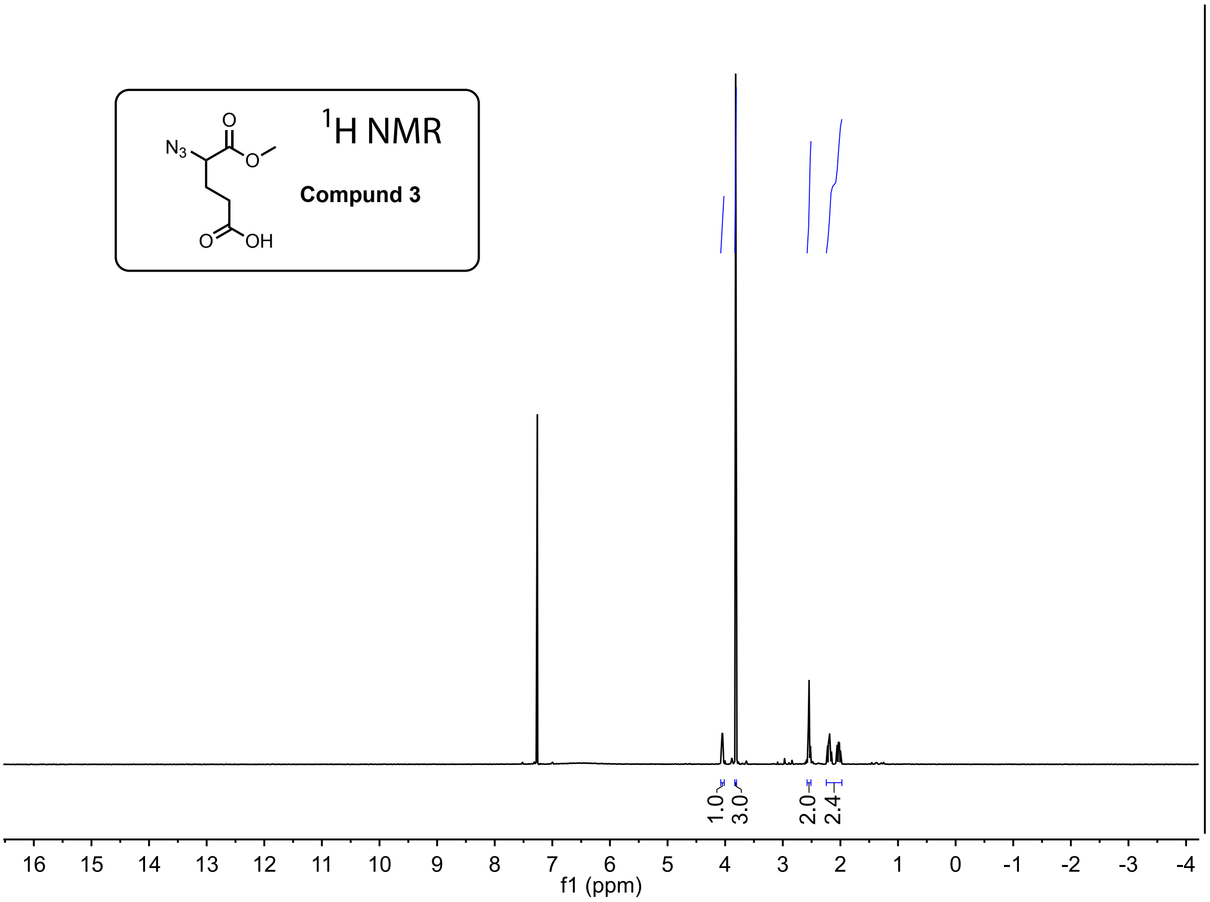

1H NMR
Compund 3
N3
O
OH
1.0
3.0
2.0
2.4
16 15 14 13 12 11 10 9 8 7 6 5 4 3 2 1 0 -1 -2 -3 -4
f1 (ppm)

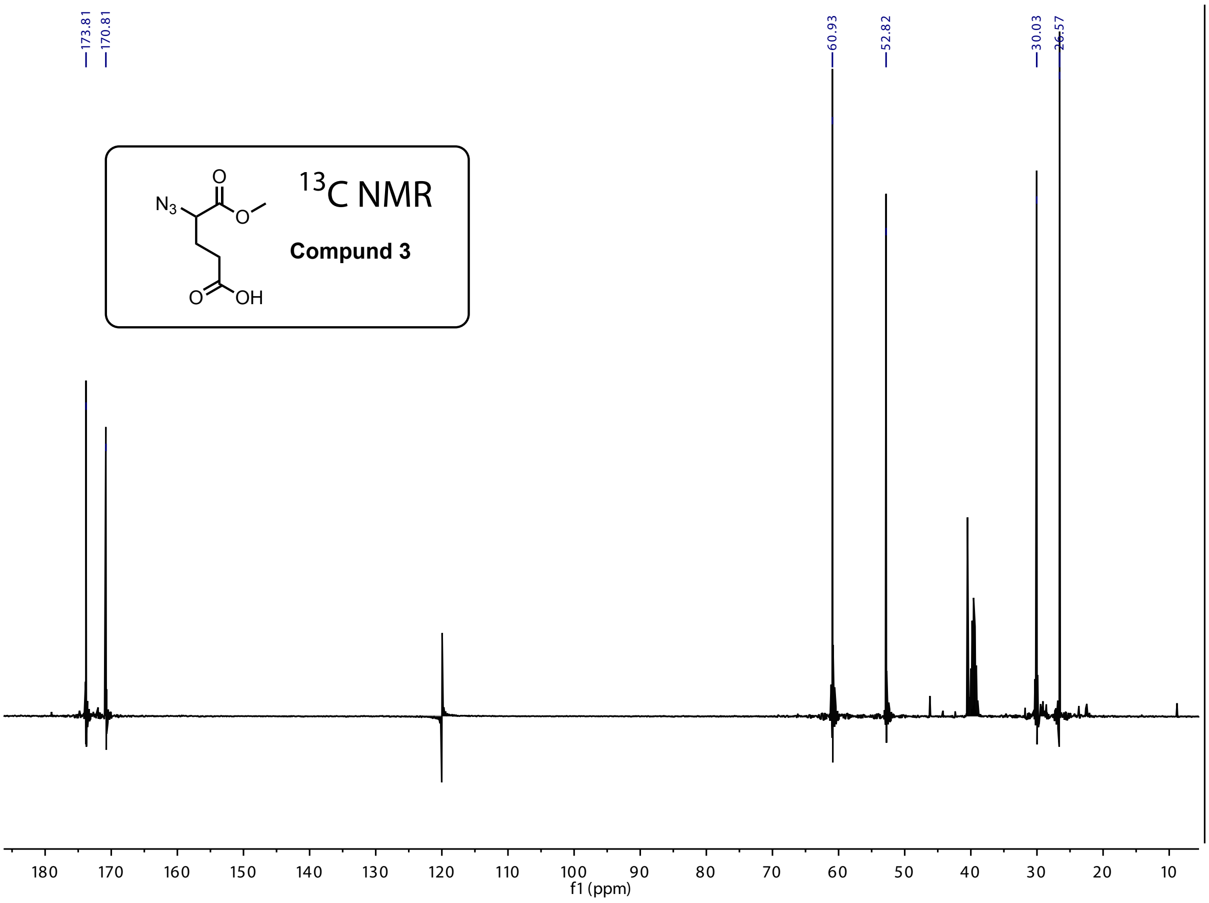

13C NMR
Compund 3
N3
O
OH
173.81
170.81
60.93
52.82
30.03
26.57
180 170 160 150 140 130 120 110 100 90 80 70 60 50 40 30 20 10
f1 (ppm)

^{1}H NMR

***(R)*-Glu-A8/B264** Compound **4**

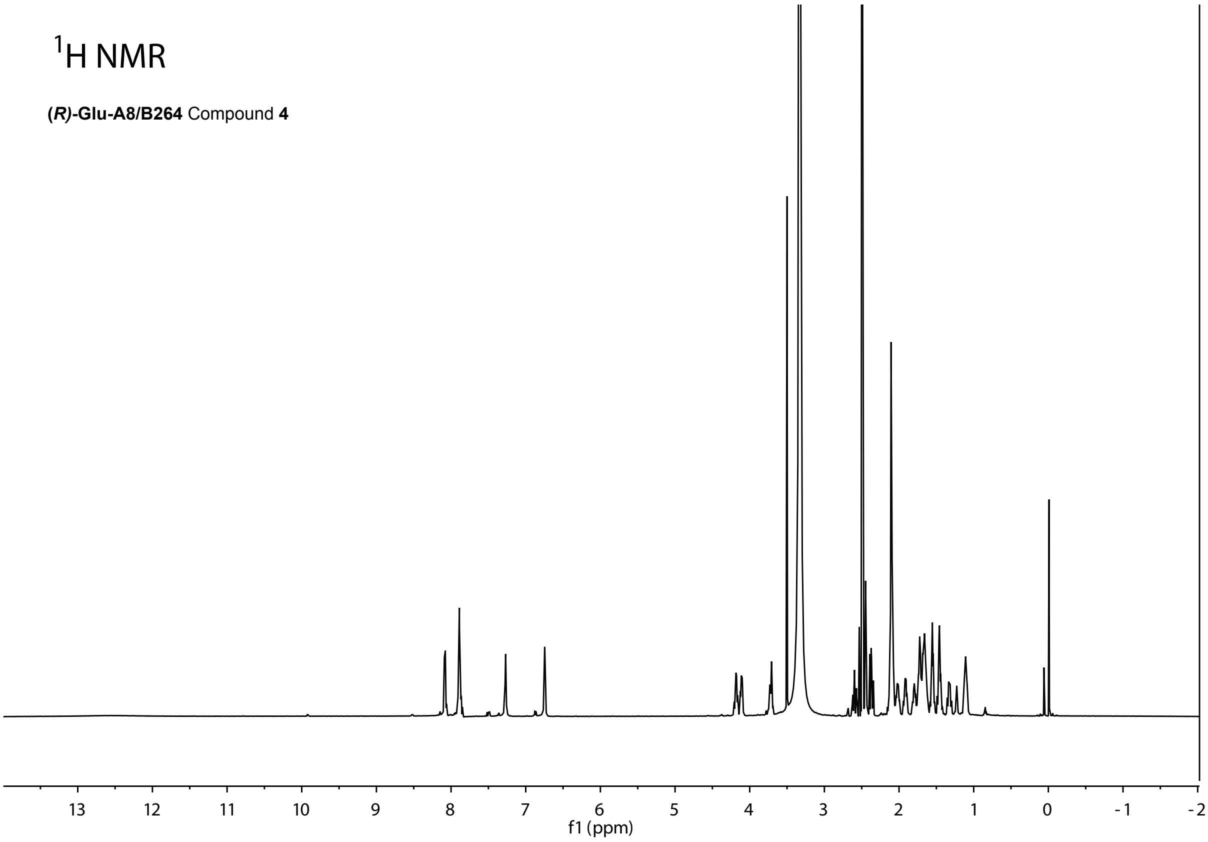

^{13}C NMR

***(R)*-Glu-A8/B264** Compound **4**

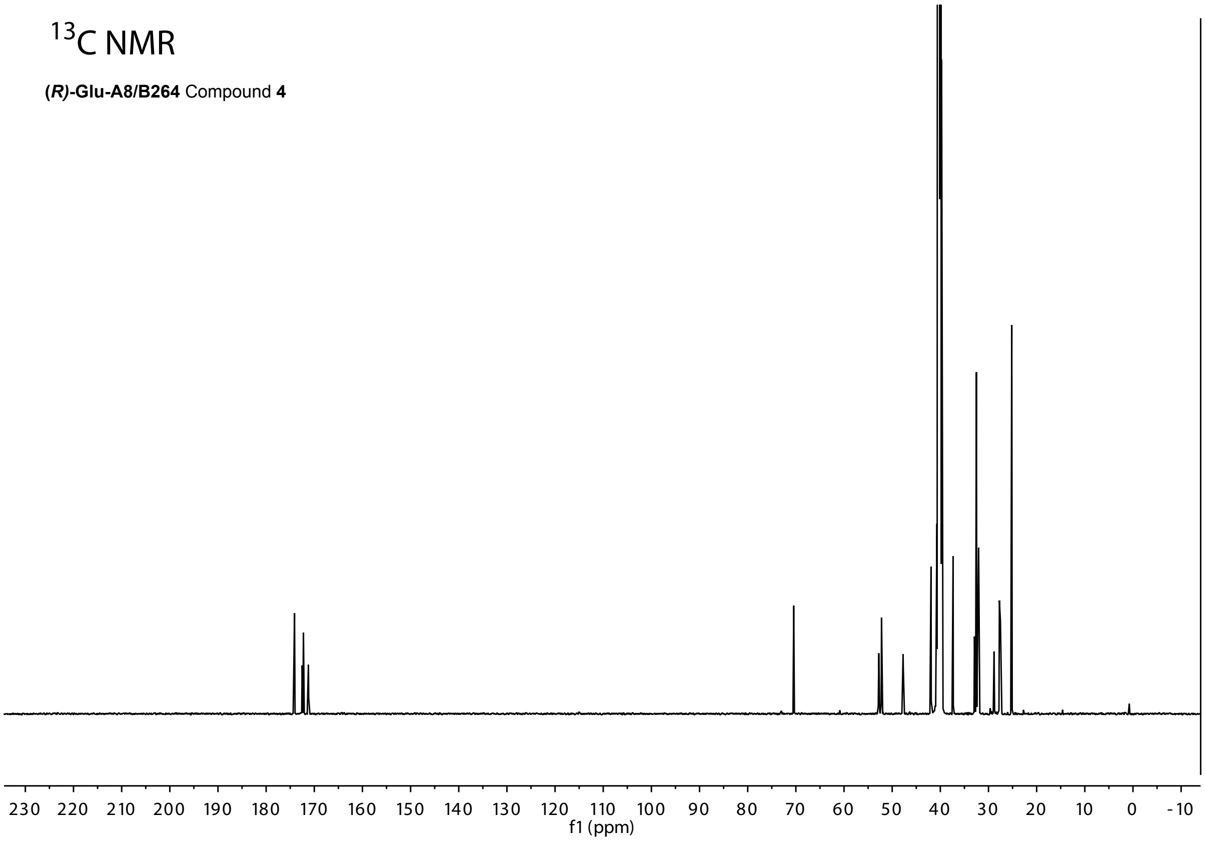

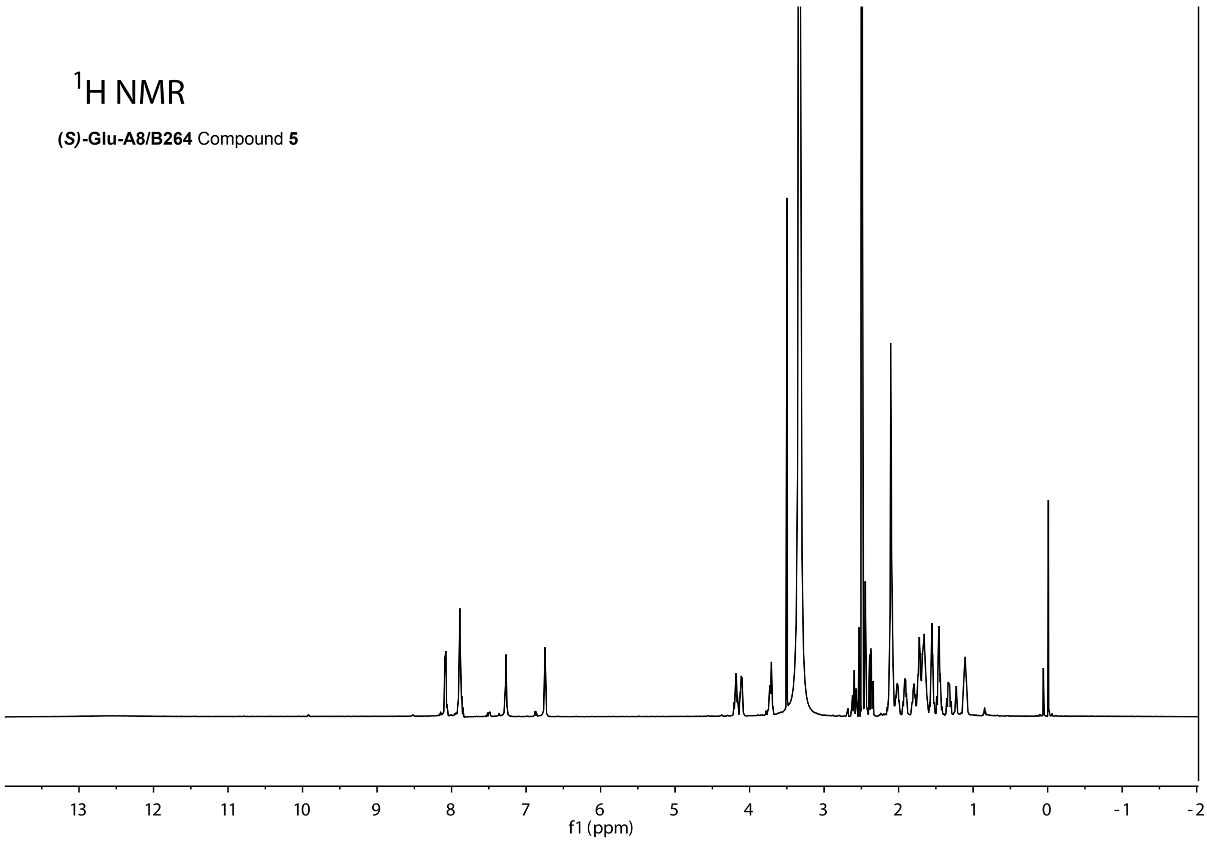
^{1}H NMR
(*S*)-Glu-A8/B264 Compound 5
f1 (ppm)

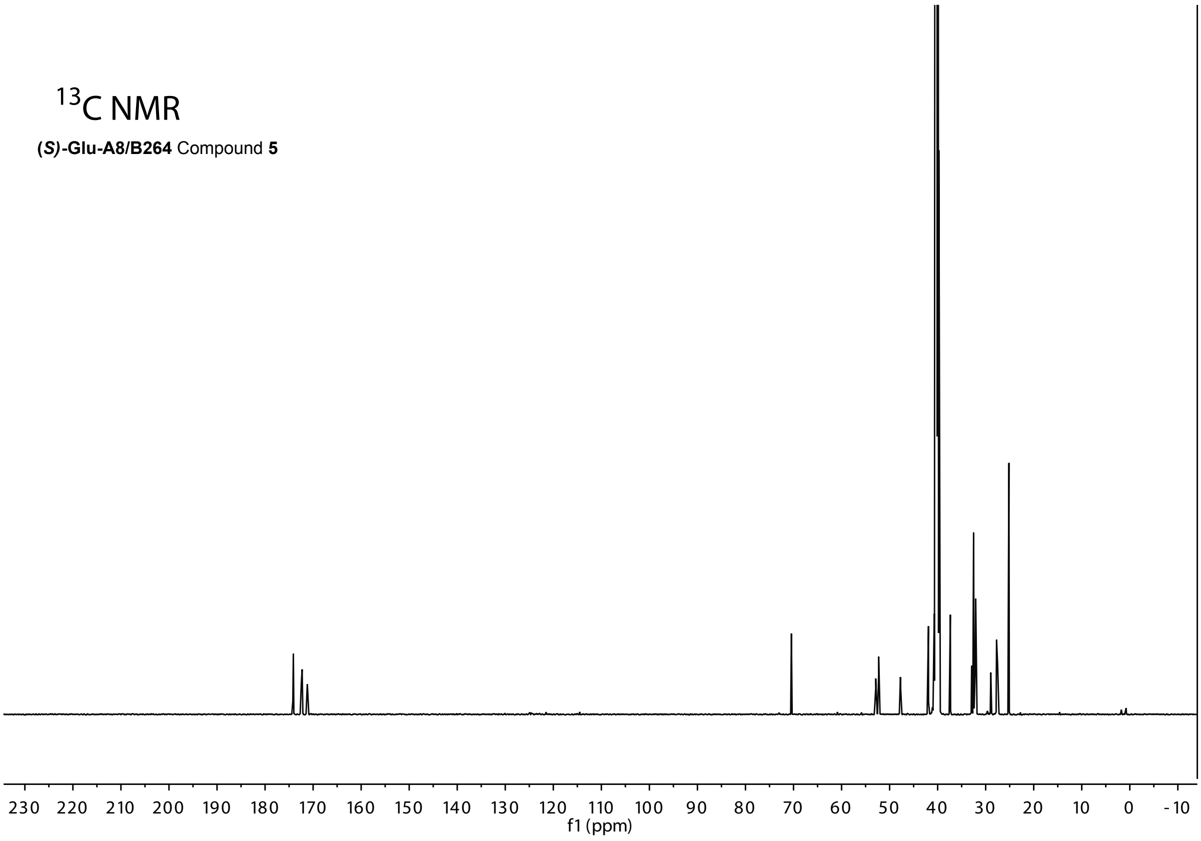
^{13}C NMR
(*S*)-Glu-A8/B264 Compound 5
f1 (ppm)

^{1}H NMR

(*R*)-Glu-A20/B611 Compound **6**

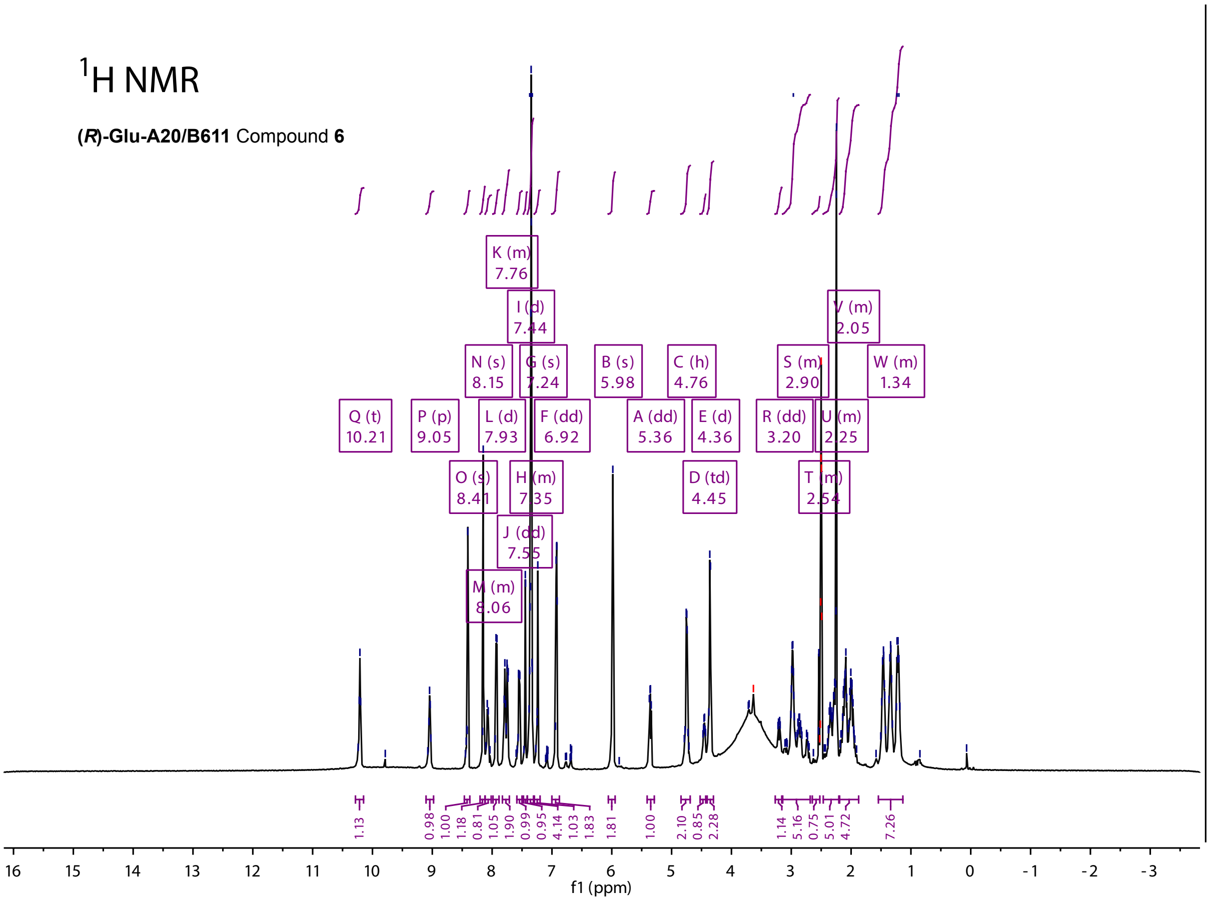

^{13}C NMR

(*R*)-Glu-A20/B611 Compound **6**

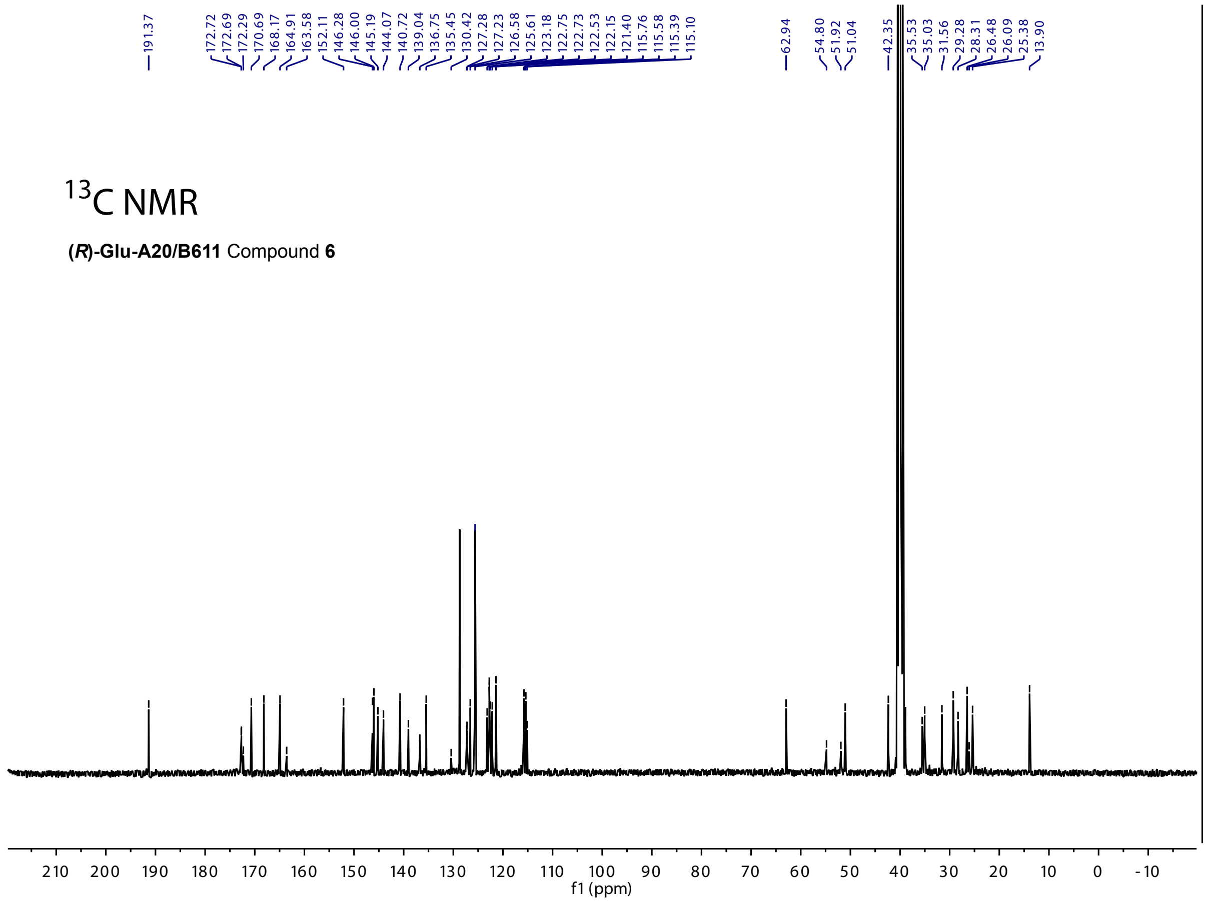

^{1}H NMR

***S*)-Glu-A20/B611** Compound **7**

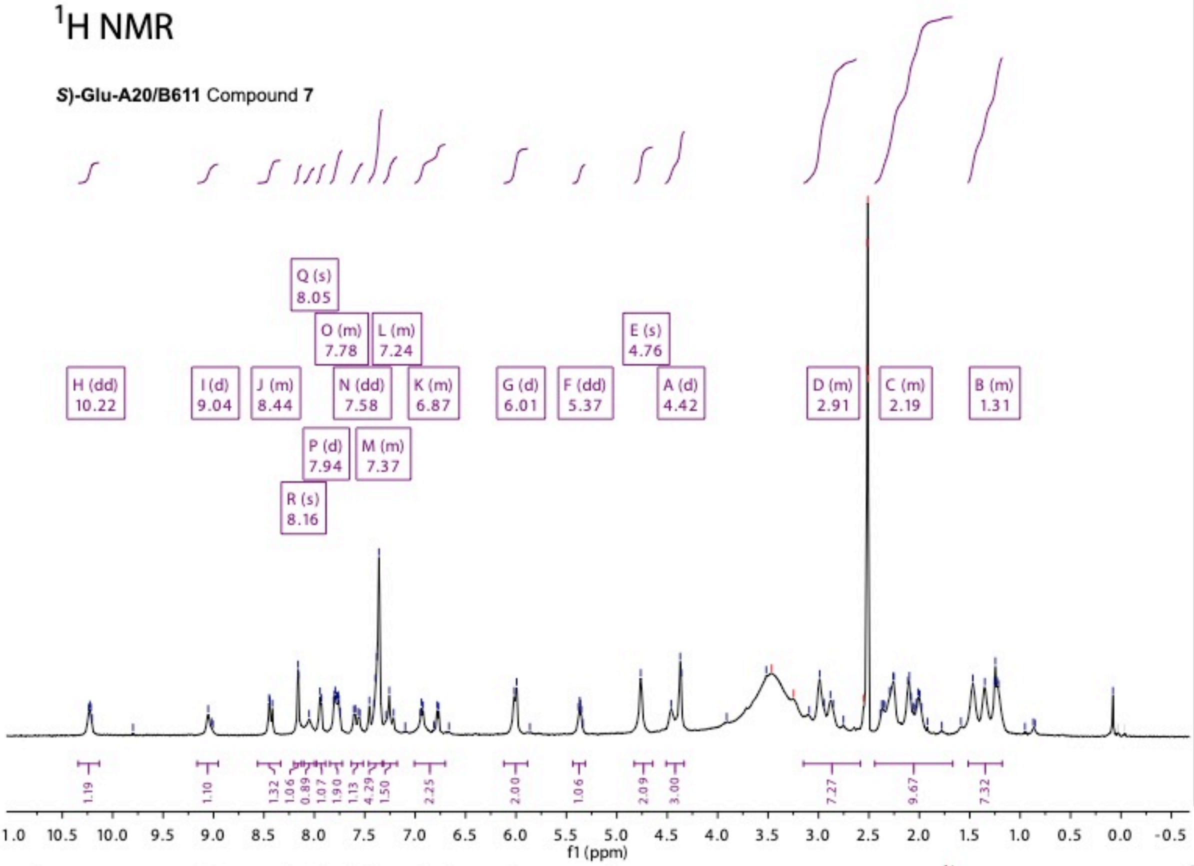

^{13}C NMR

***S*)-Glu-A20/B611** Compound **7**

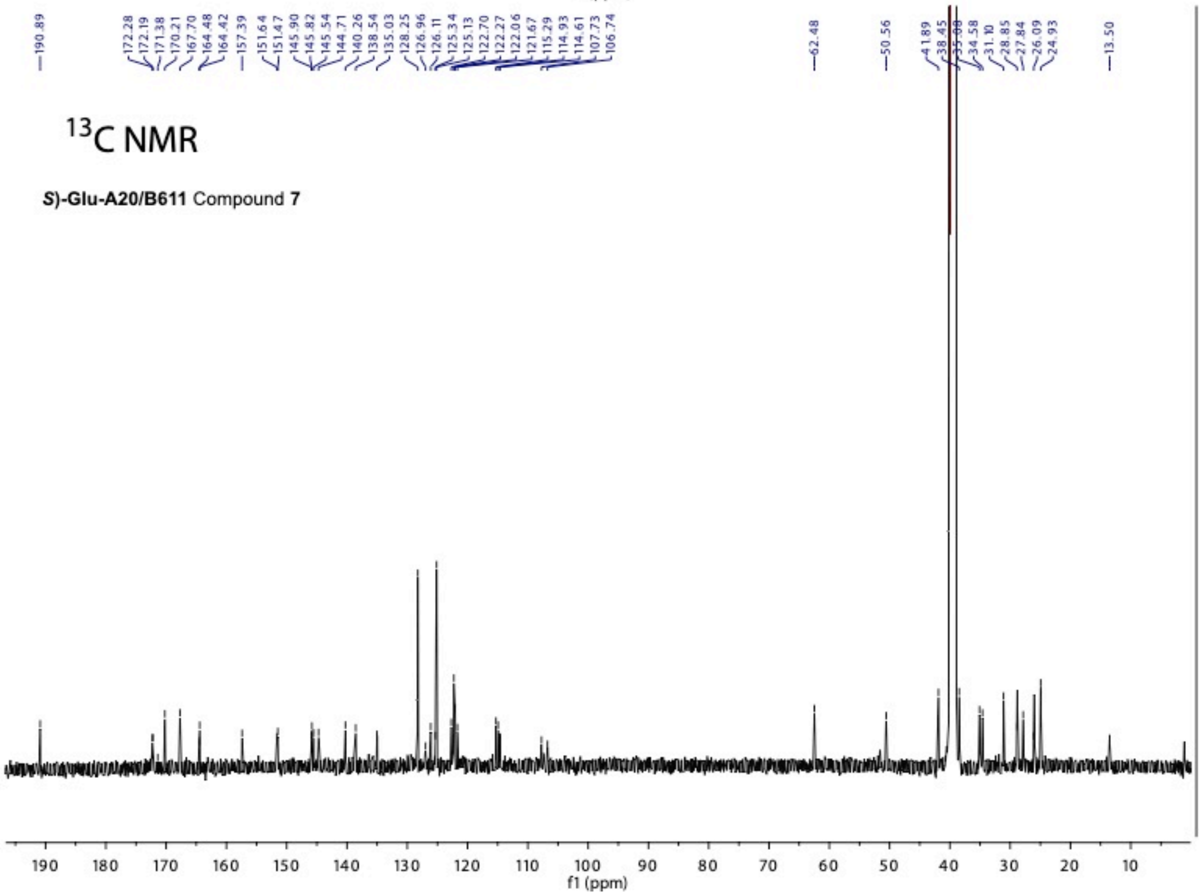

^{1}H NMR

(R)-Glu-A120/B235 Compound **8**

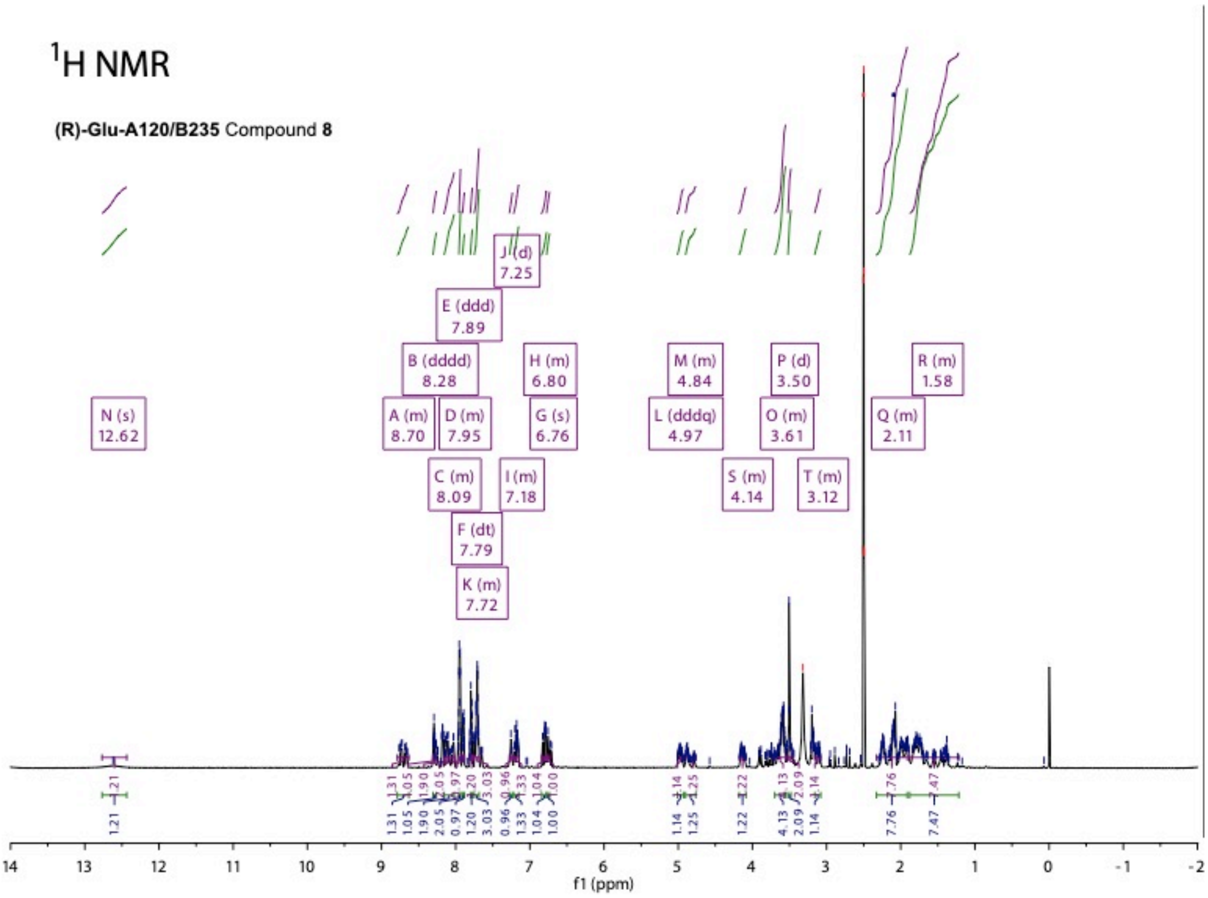

^{13}C NMR

(R)-Glu-A120/B235 Compound **8**

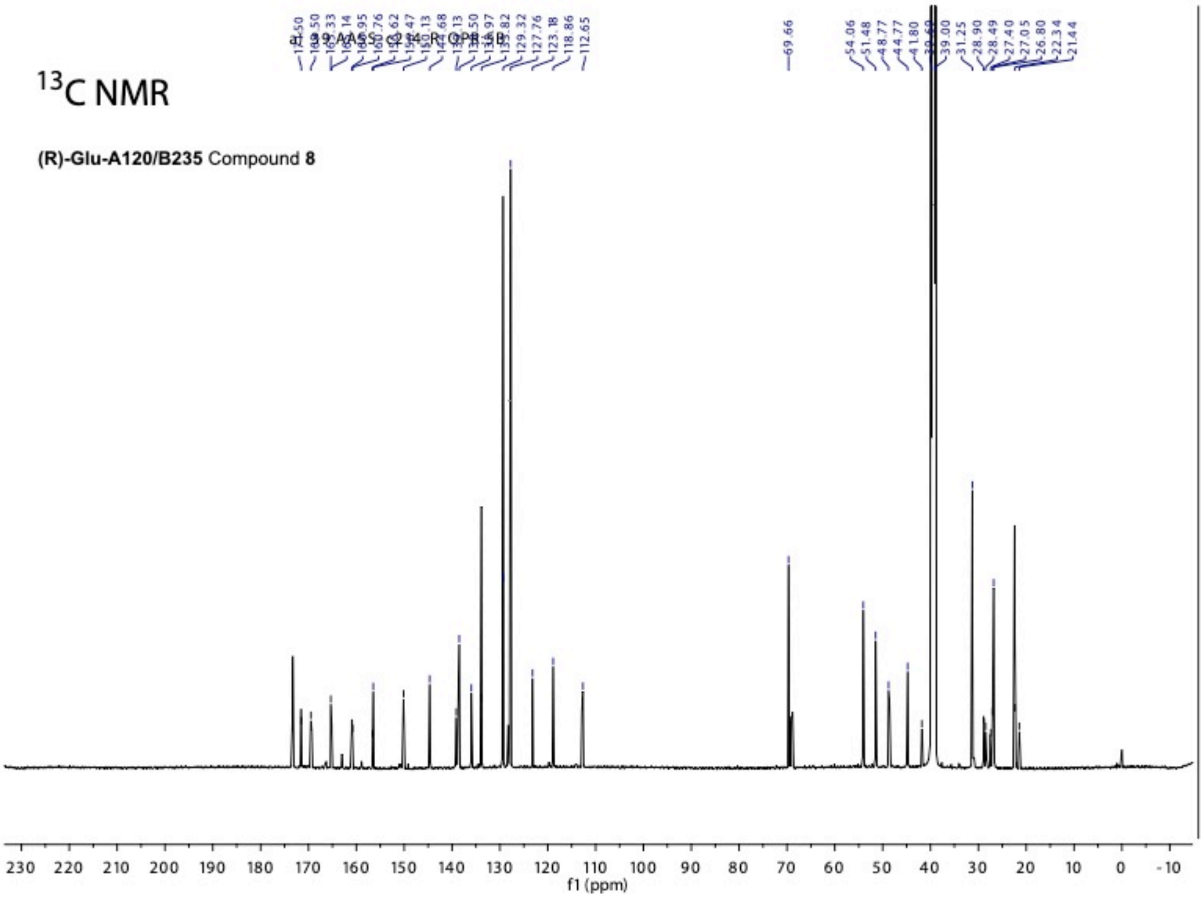

^{1}H NMR

(S)-Glu-A120/B235 Compound **9**

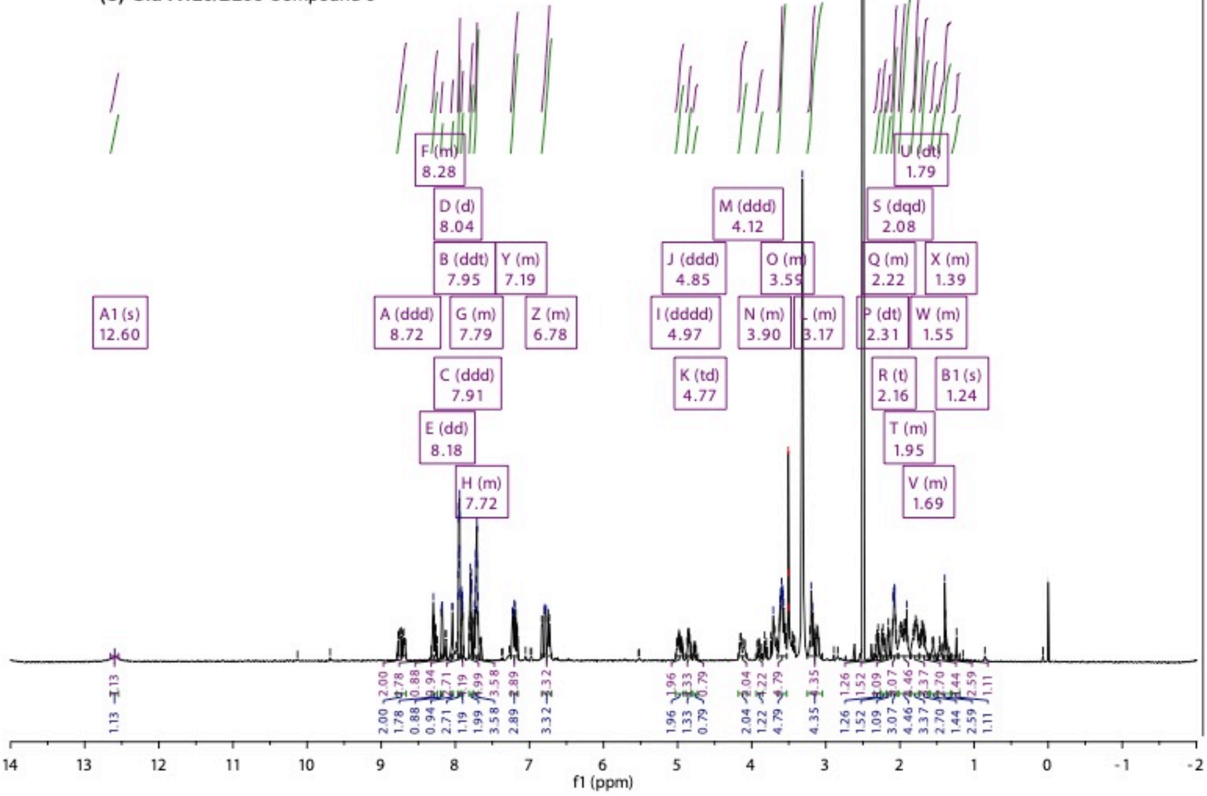

^{13}C NMR

(S)-Glu-A120/B235 Compound **9**

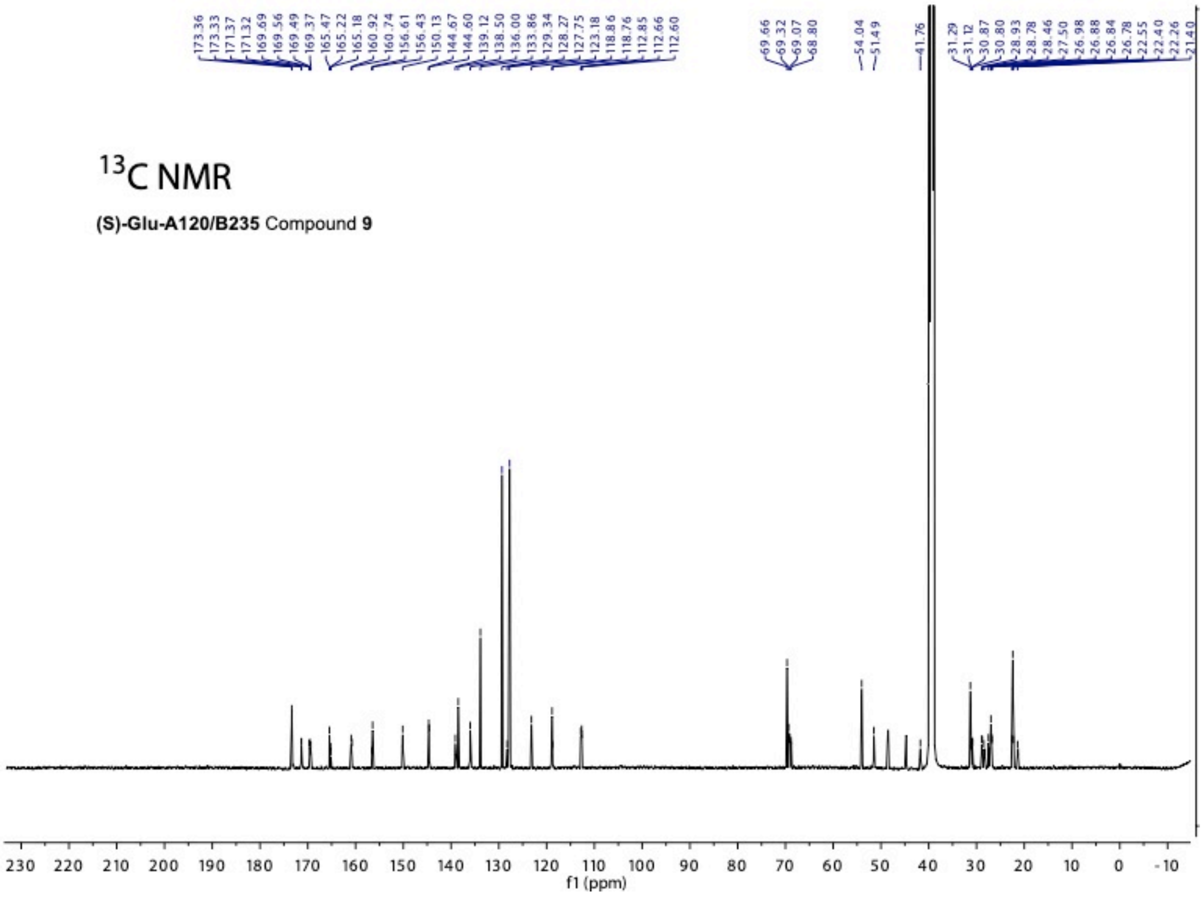

^{1}H NMR

(*R*)-Glu-A86/B371 Compound **10**

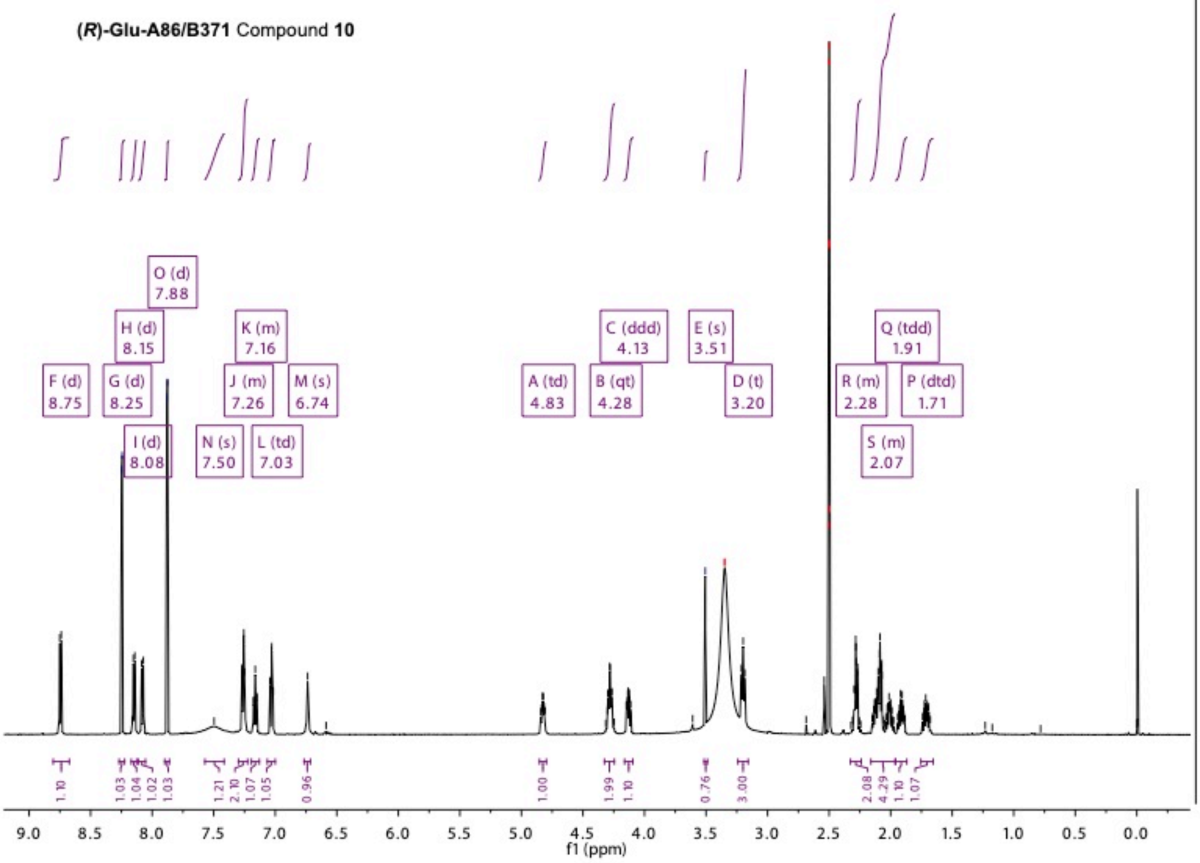

^{13}C NMR

(*R*)-Glu-A86/B371 Compound **10**

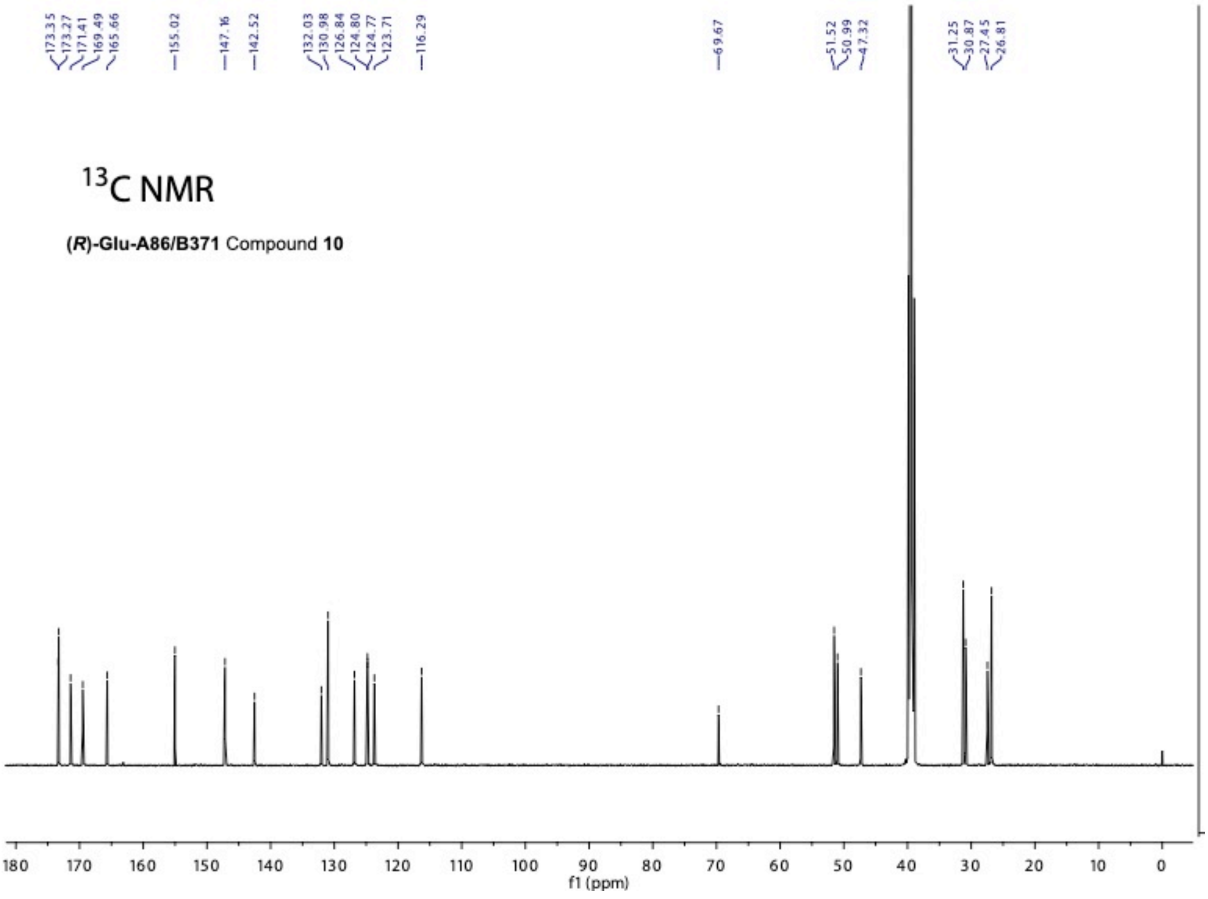

^{1}H NMR

(*S*)-Glu-A86/B371 Compound **11**

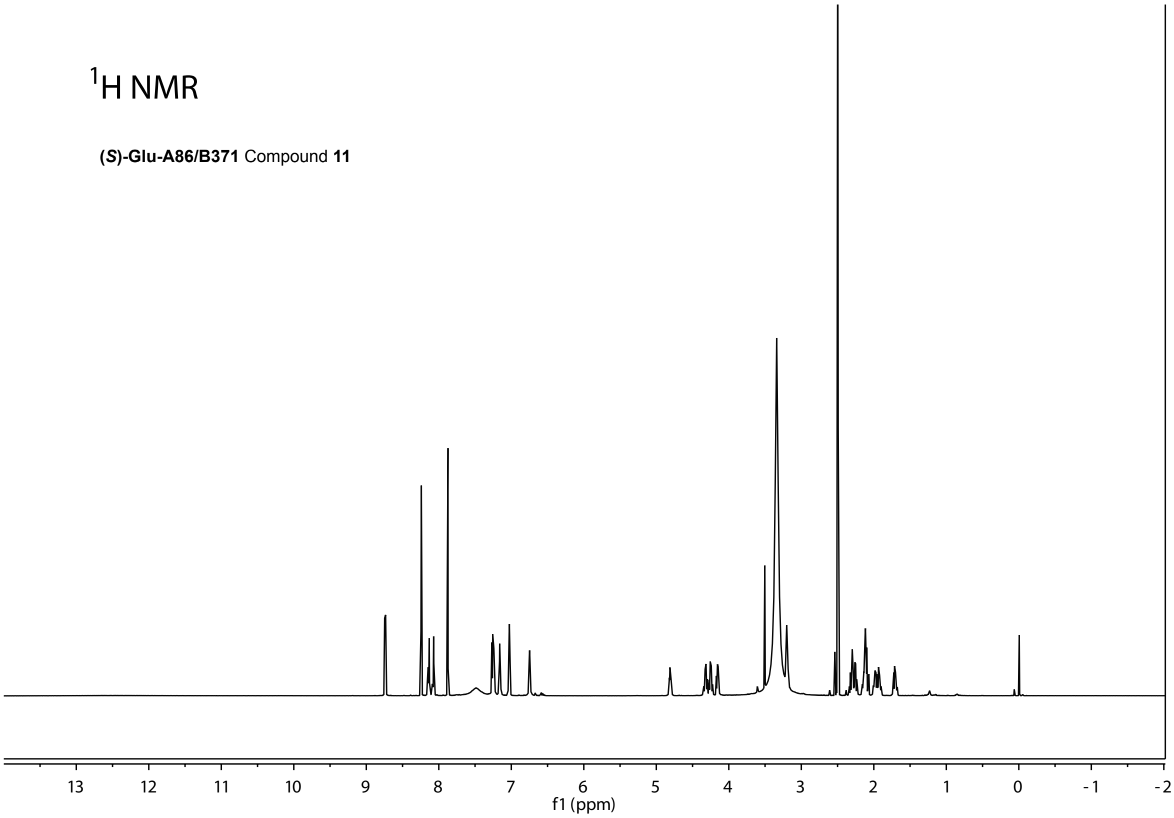

^{13}C NMR

(*S*)-Glu-A86/B371 Compound **11**

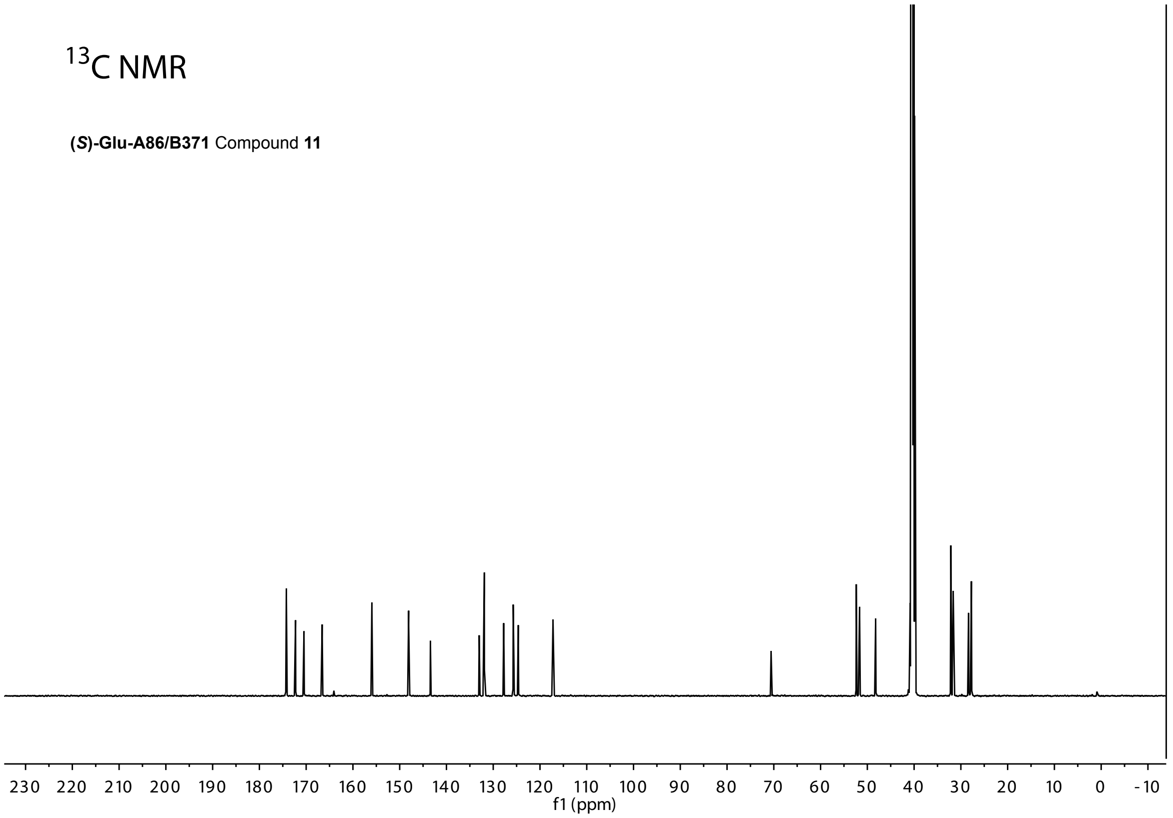

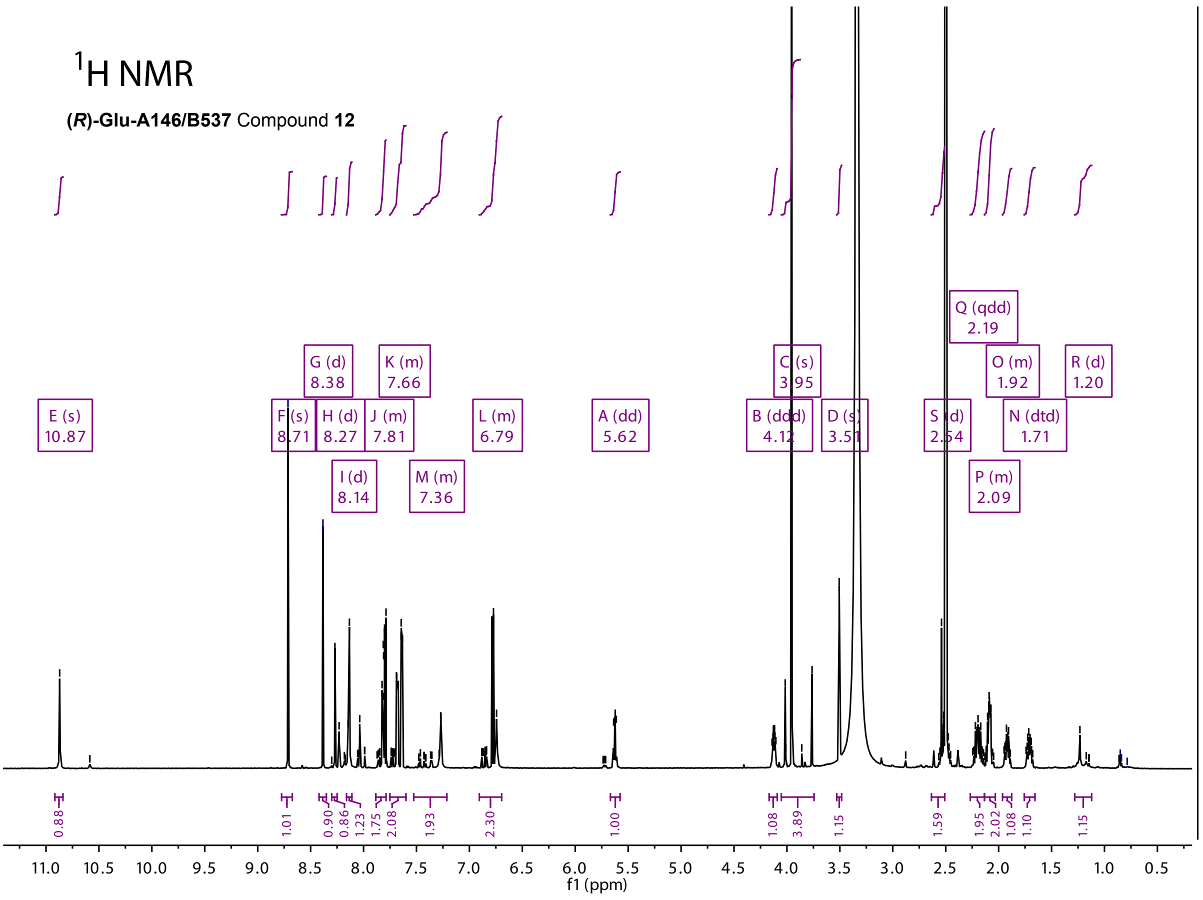

1H NMR
(R)-Glu-A146/B537 Compound 12
E (s) 10.87
F (s) 8.71
G (d) 8.38
H (d) 8.27
I (d) 8.14
J (m) 7.81
K (m) 7.66
M (m) 7.36
L (m) 6.79
A (dd) 5.62
B (ddd) 4.12
C (s) 3.95
D (s) 3.51
S (d) 2.54
Q (qdd) 2.19
P (m) 2.09
O (m) 1.92
N (dtd) 1.71
R (d) 1.20
0.88
1.01
0.90
0.86
1.23
1.75
2.08
1.93
2.30
1.00
1.08
3.89
1.15
1.59
1.95
2.02
1.08
1.10
1.15
f1 (ppm)

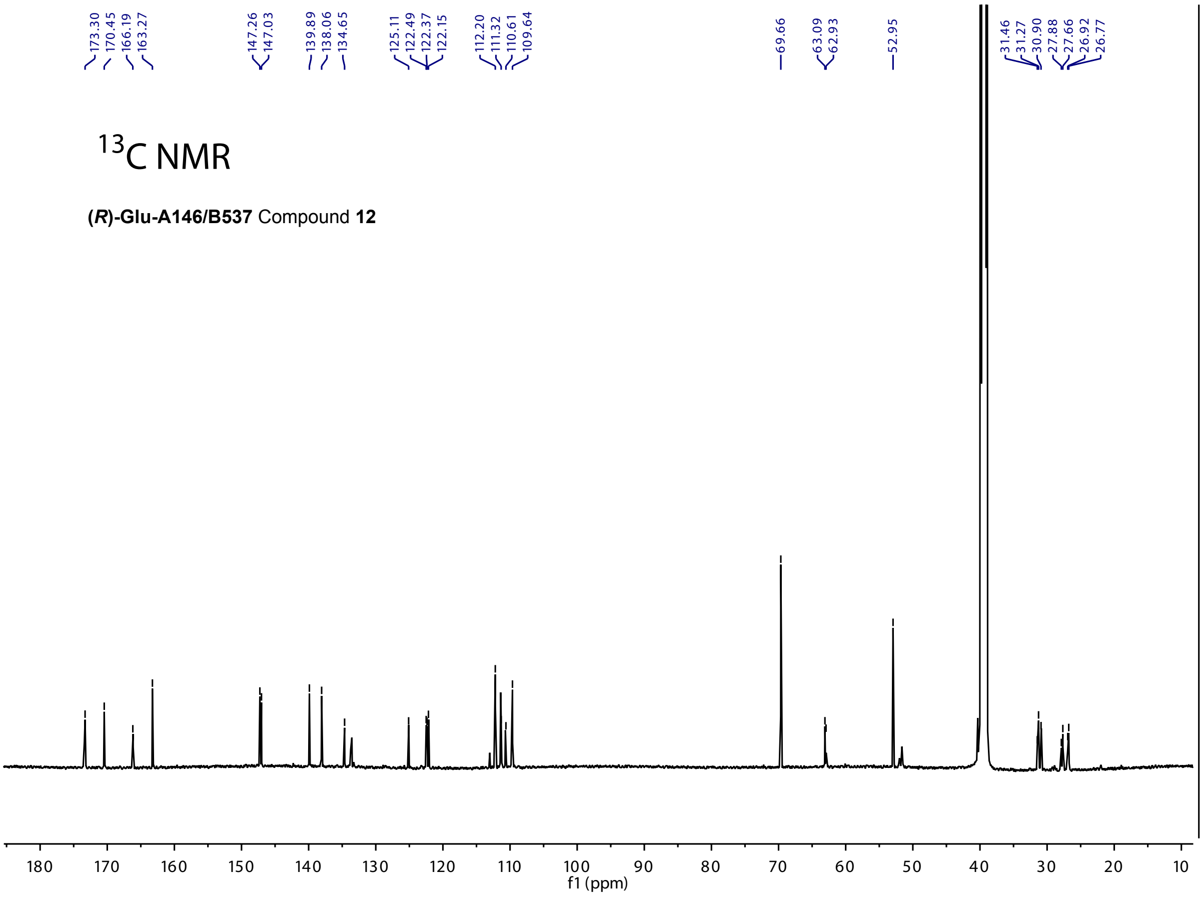

13C NMR
(R)-Glu-A146/B537 Compound 12
173.30
170.45
166.19
163.27
147.26
147.03
139.89
138.06
134.65
125.11
122.49
122.37
122.15
112.20
111.32
110.61
109.64
69.66
63.09
62.93
52.95
31.46
31.27
30.90
27.88
27.66
26.92
26.77
f1 (ppm)

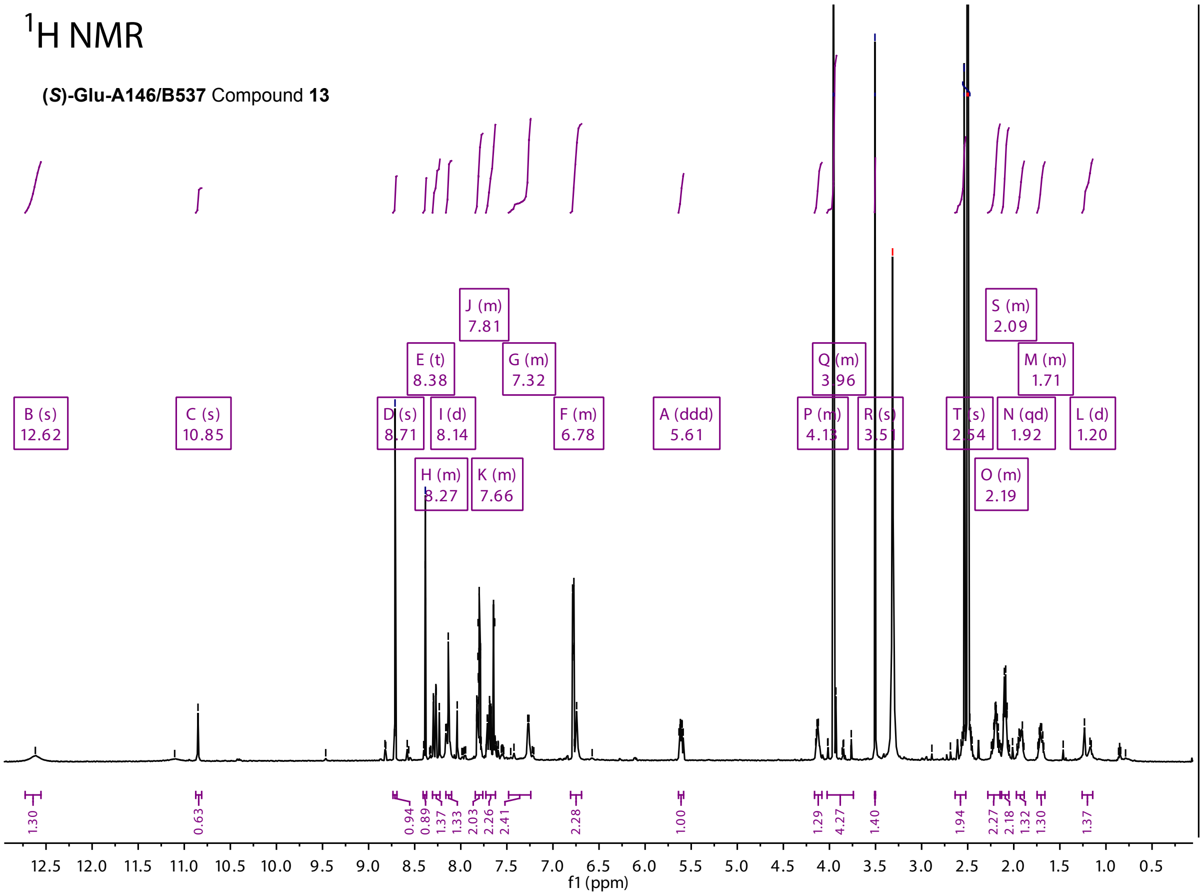

1H NMR
(S)-Glu-A146/B537 Compound 13
B (s) 12.62
C (s) 10.85
D (s) 8.71
E (t) 8.38
H (m) 8.27
I (d) 8.14
J (m) 7.81
K (m) 7.66
G (m) 7.32
F (m) 6.78
A (ddd) 5.61
P (m) 4.13
Q (m) 3.96
R (s) 3.51
T (s) 2.54
S (m) 2.09
O (m) 2.19
N (qd) 1.92
M (m) 1.71
L (d) 1.20
f1 (ppm)

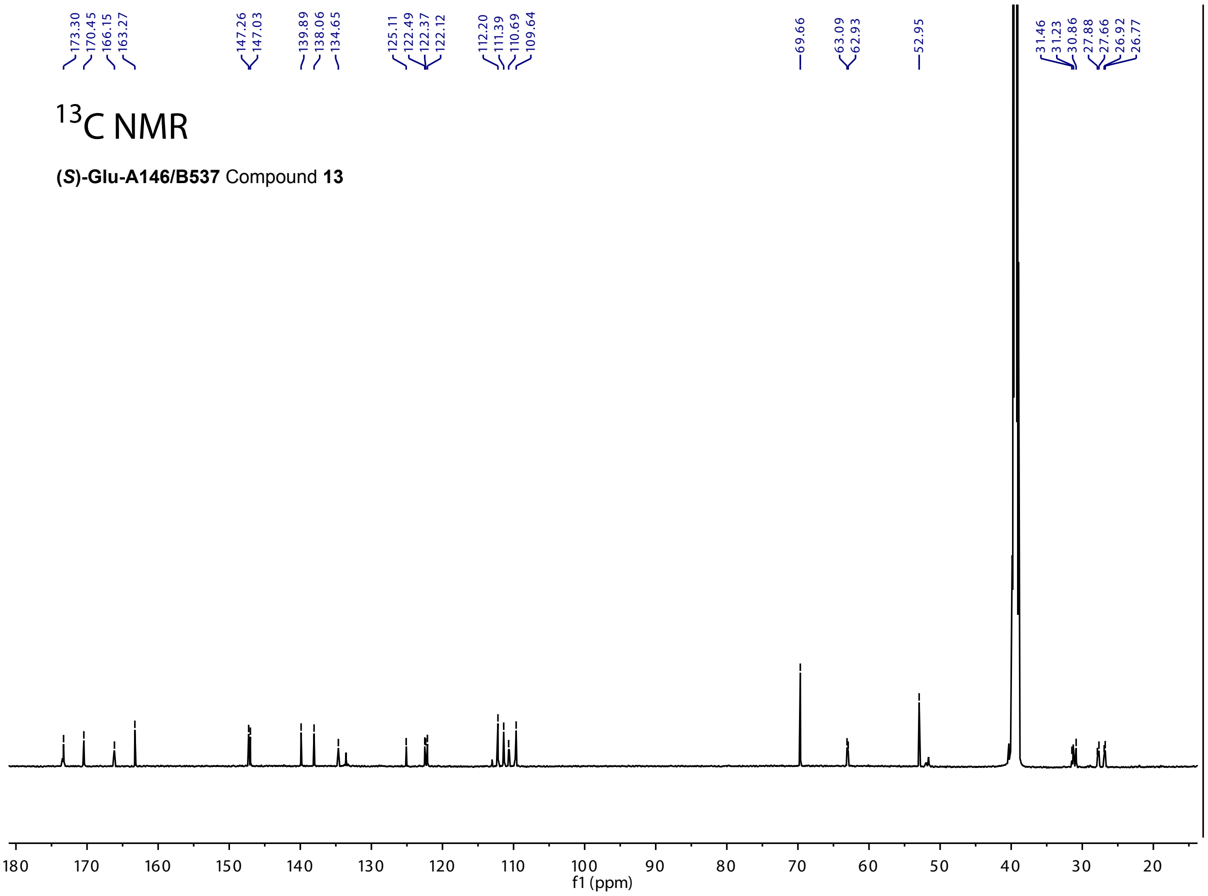

13C NMR
(S)-Glu-A146/B537 Compound 13
173.30
170.45
166.15
163.27
147.26
147.03
139.89
138.06
134.65
125.11
122.49
122.37
122.12
112.20
111.39
110.69
109.64
69.66
63.09
62.93
52.95
31.46
31.23
30.86
27.88
27.66
26.92
26.77
f1 (ppm)

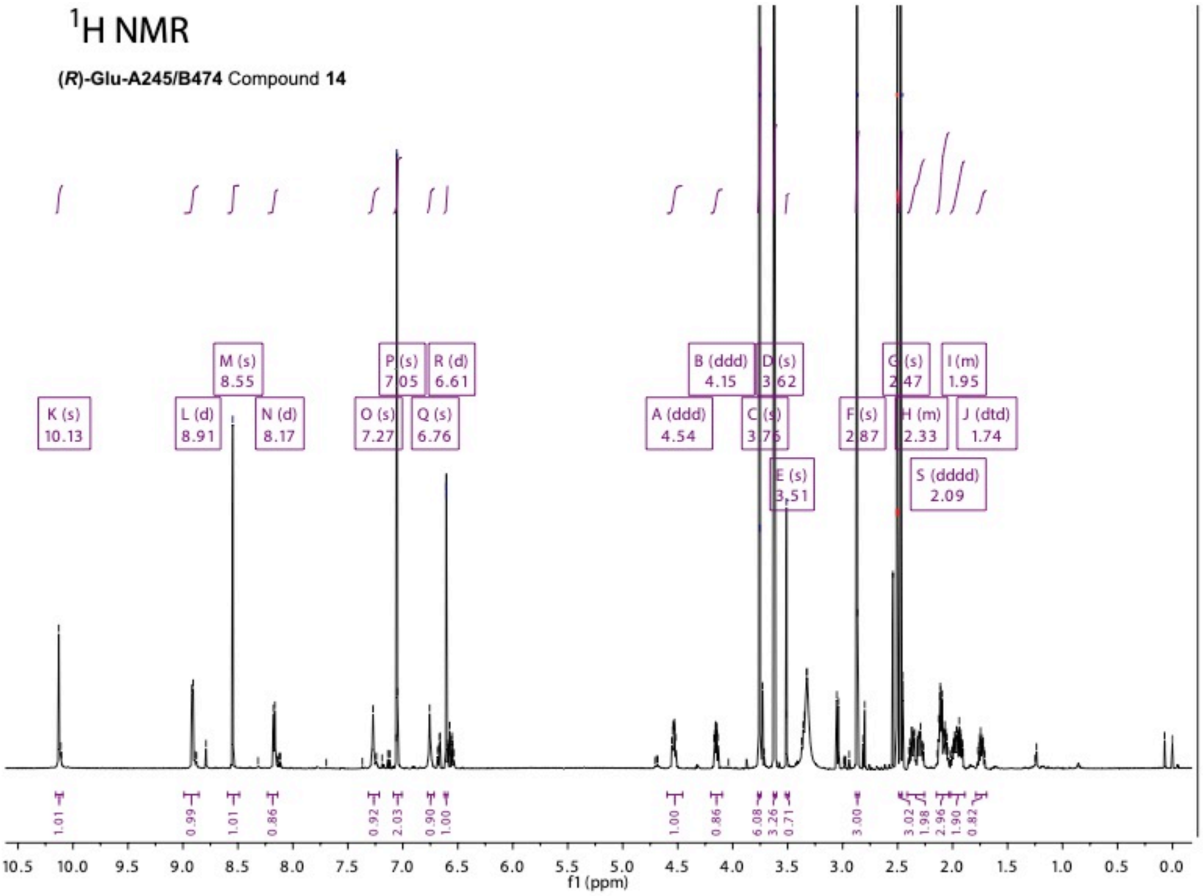

^{1}H NMR
(*R*)-Glu-A245/B474 Compound 14
K (s) 10.13
L (d) 8.91
M (s) 8.55
N (d) 8.17
O (s) 7.27
P (s) 7.05
Q (s) 6.76
R (d) 6.61
A (ddd) 4.54
B (ddd) 4.15
C (s) 3.76
D (s) 3.62
E (s) 3.51
F (s) 2.87
G (s) 2.47
H (m) 2.33
S (dddd) 2.09
I (m) 1.95
J (dtd) 1.74
1.01 0.99 1.01 0.86 0.92 2.03 0.90 1.00 1.00 0.86 6.08 3.26 0.71 3.00 3.02 1.98 2.96 1.90 0.82
f1 (ppm)

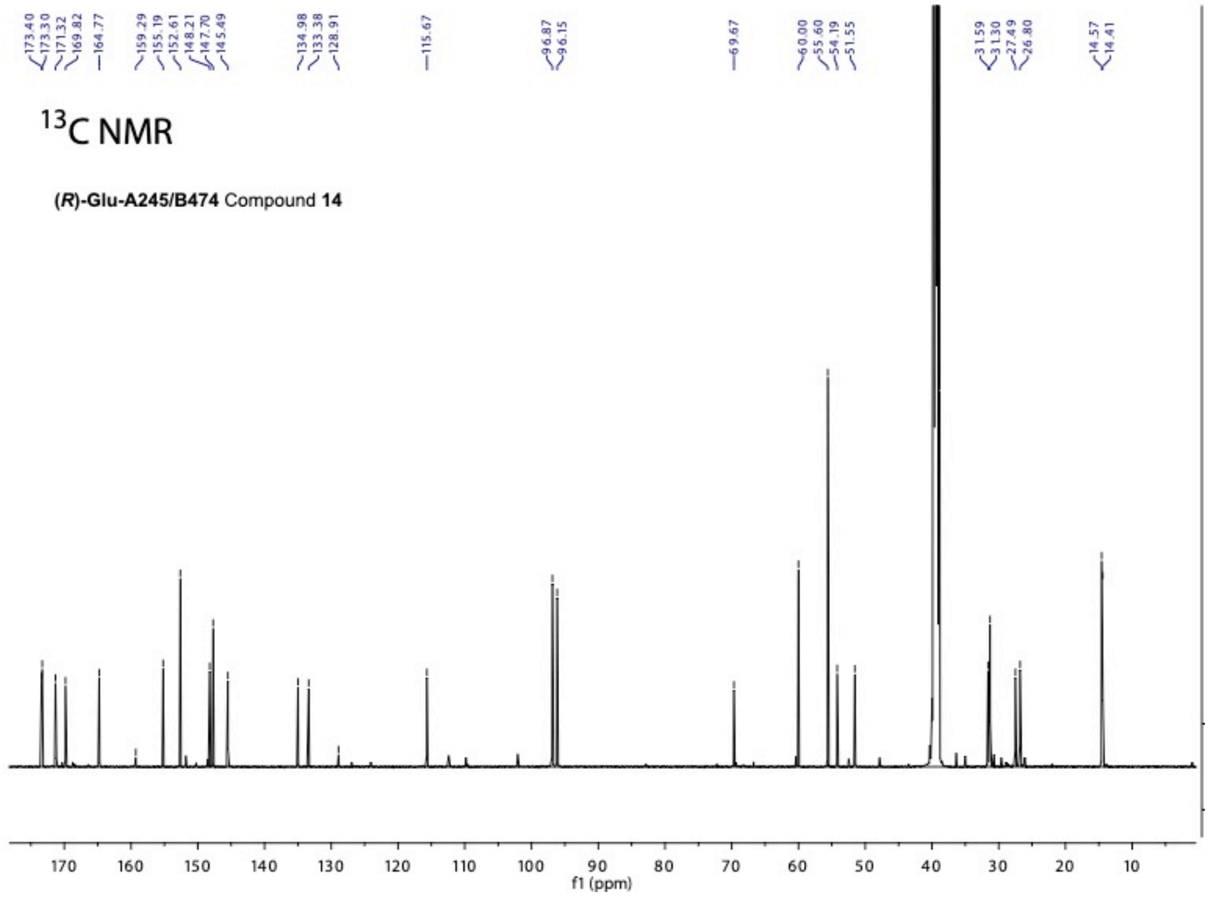

173.40 173.30 171.32 169.82 164.77 159.29 155.19 152.61 148.21 147.70 145.49 134.98 133.38 128.91 115.67 96.87 96.15 69.67 60.00 55.60 54.19 51.55 31.59 31.30 27.49 26.80 14.57 14.41
^{13}C NMR
(*R*)-Glu-A245/B474 Compound 14
f1 (ppm)

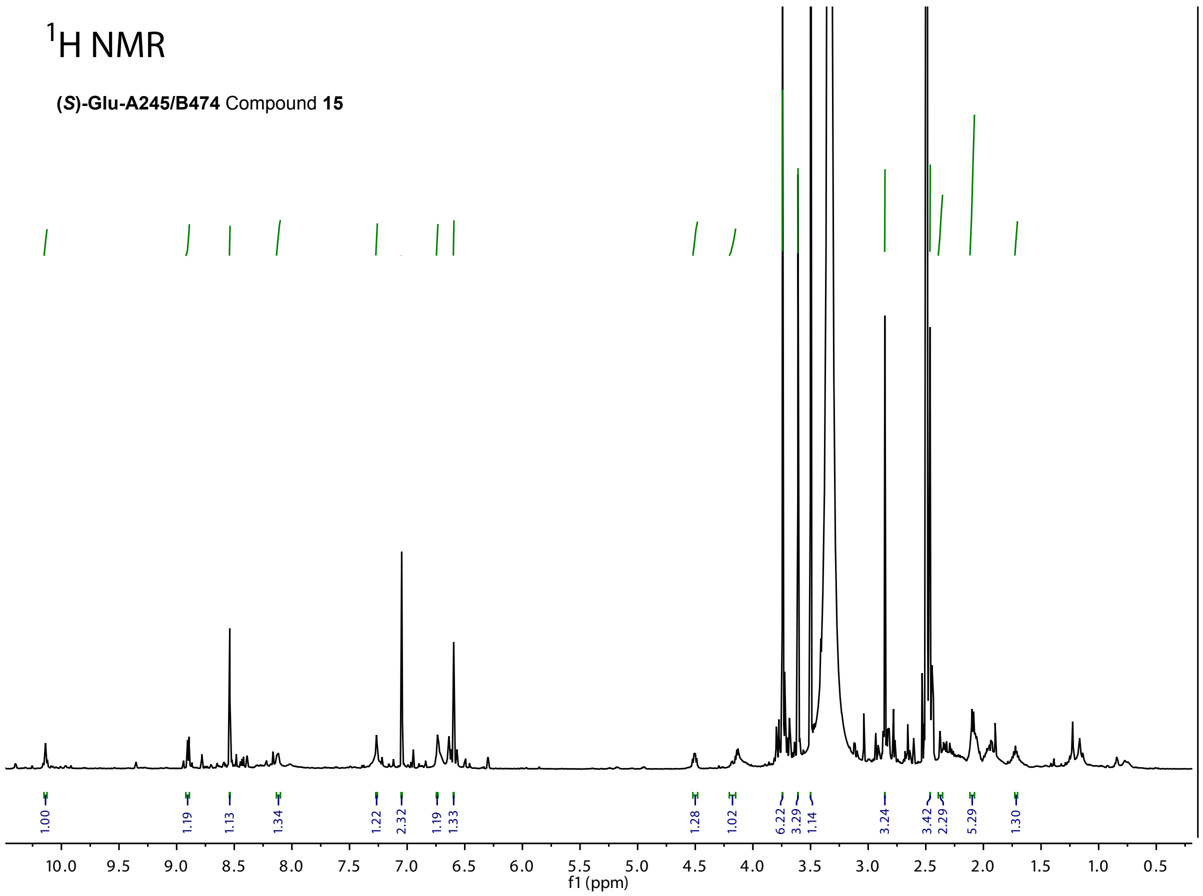

1H NMR
(S)-Glu-A245/B474 Compound 15
f1 (ppm)

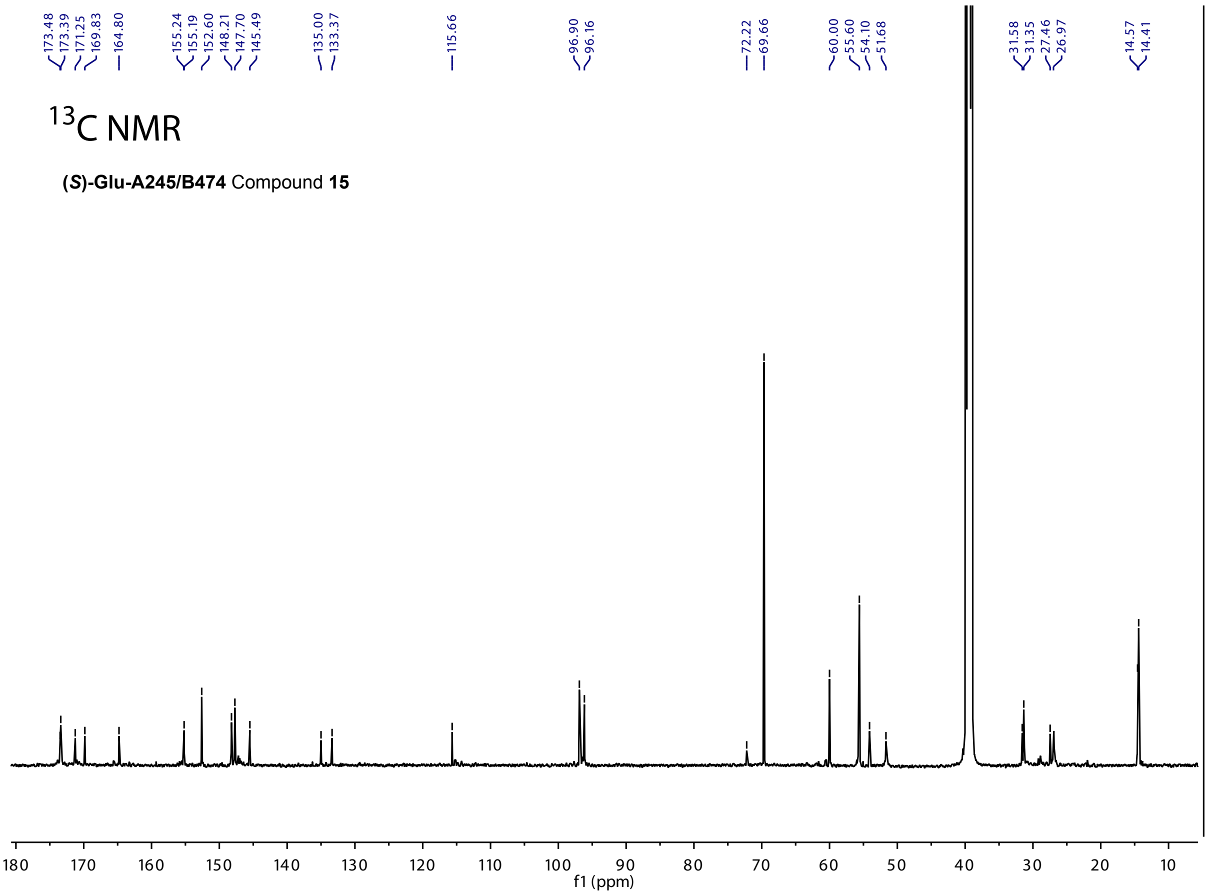

13C NMR
(S)-Glu-A245/B474 Compound 15
f1 (ppm)

^{1}H NMR

(*R*)-Glu-A200/B44 Compound **16**

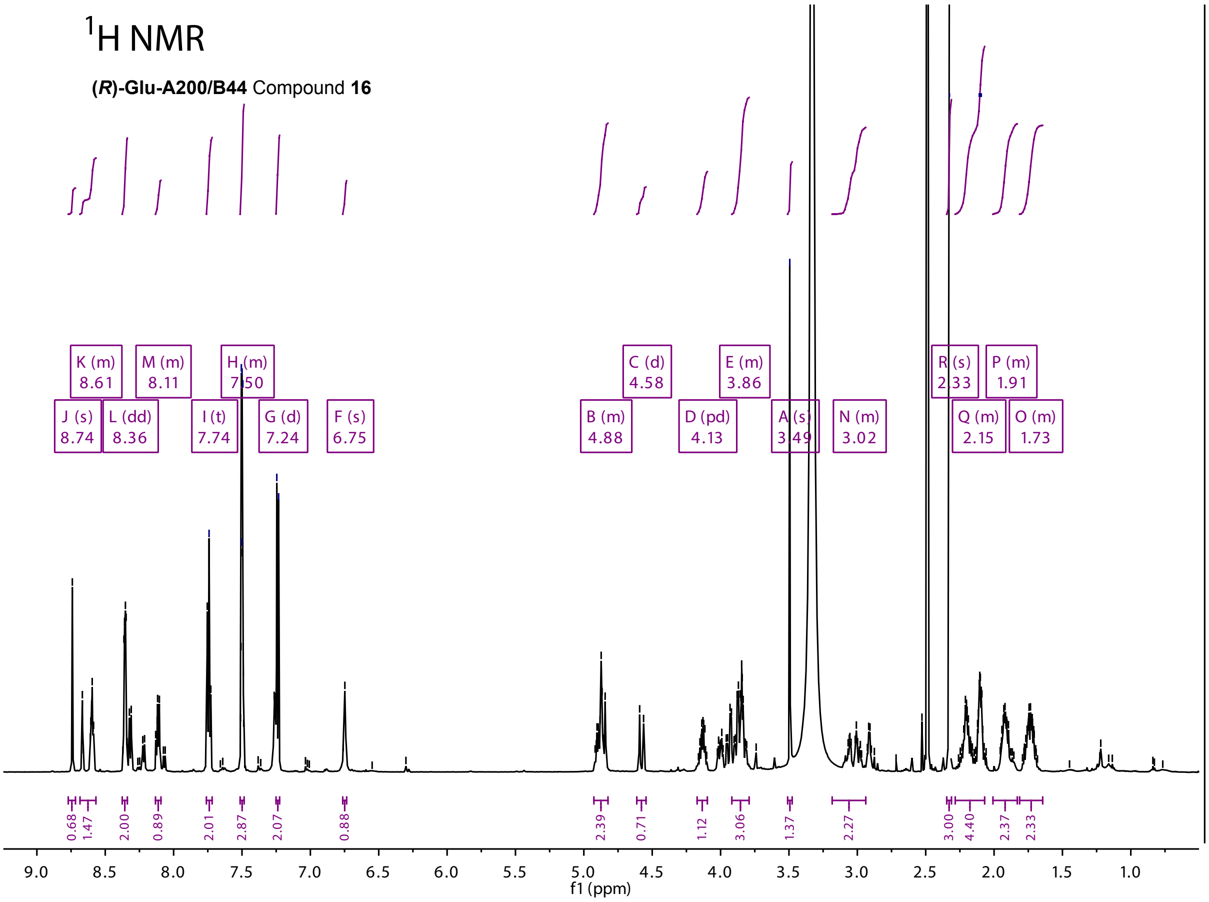

^{13}C NMR

(*R*)-Glu-A200/B44 Compound **16**

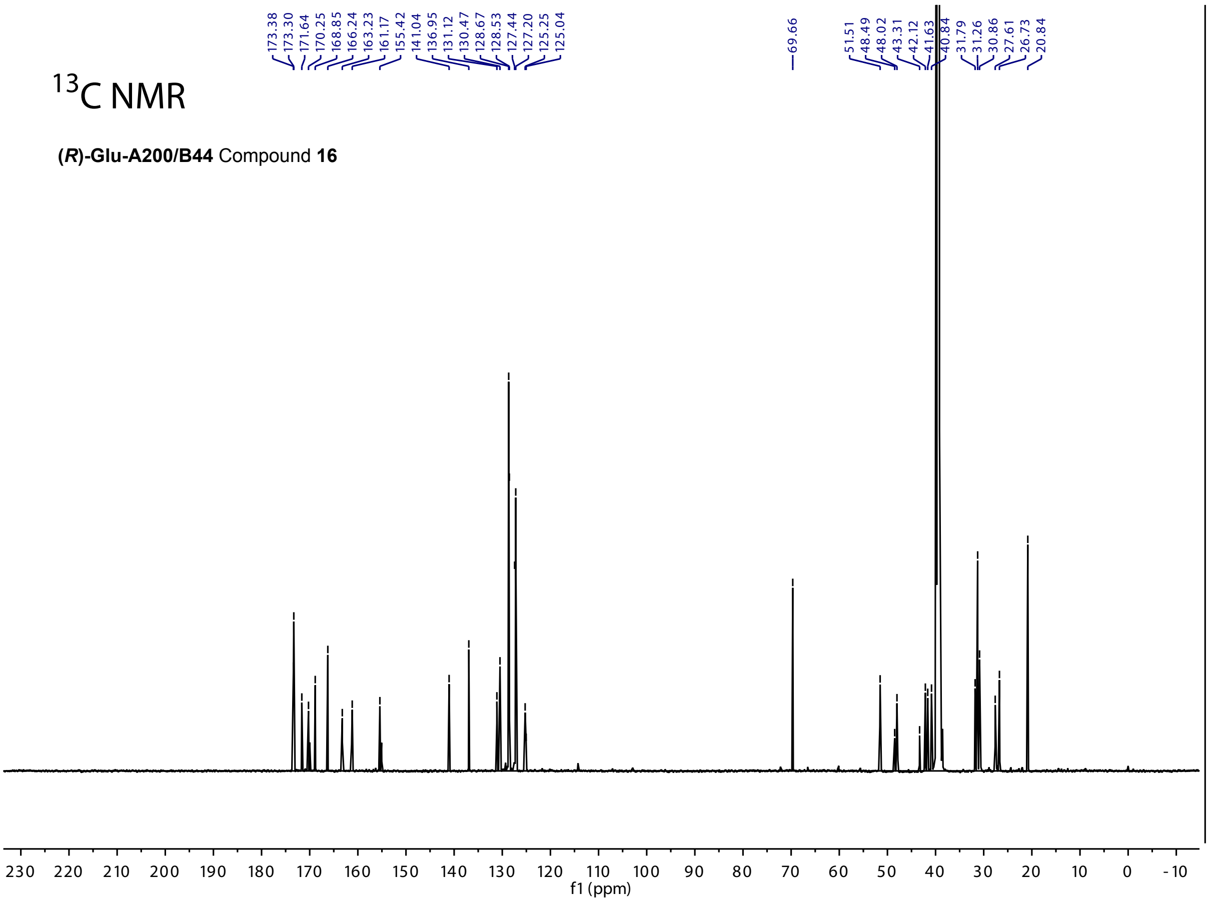

^{1}H NMR

(*S*)-Glu-A200/B44 Compound **17**

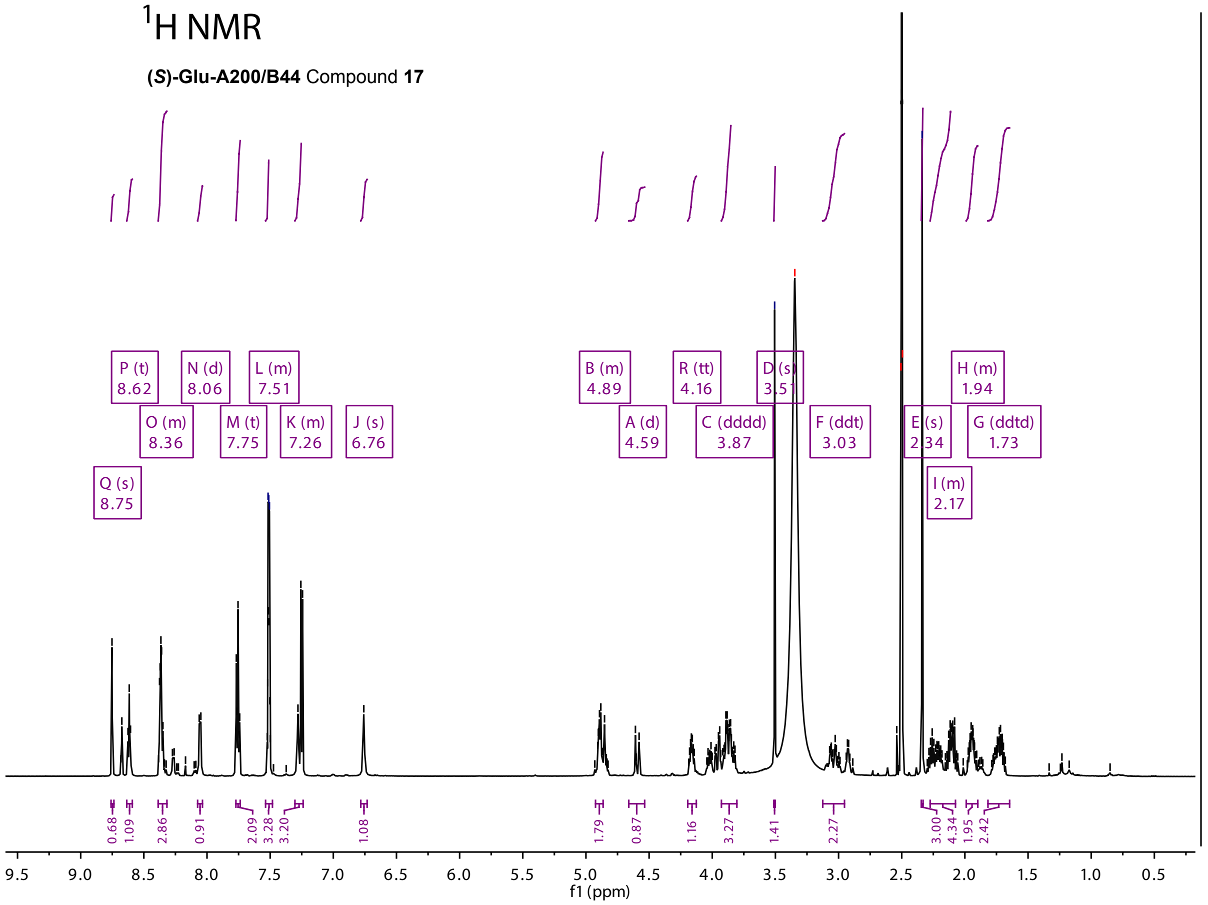

^{13}C NMR

(*S*)-Glu-A200/B44 Compound **17**

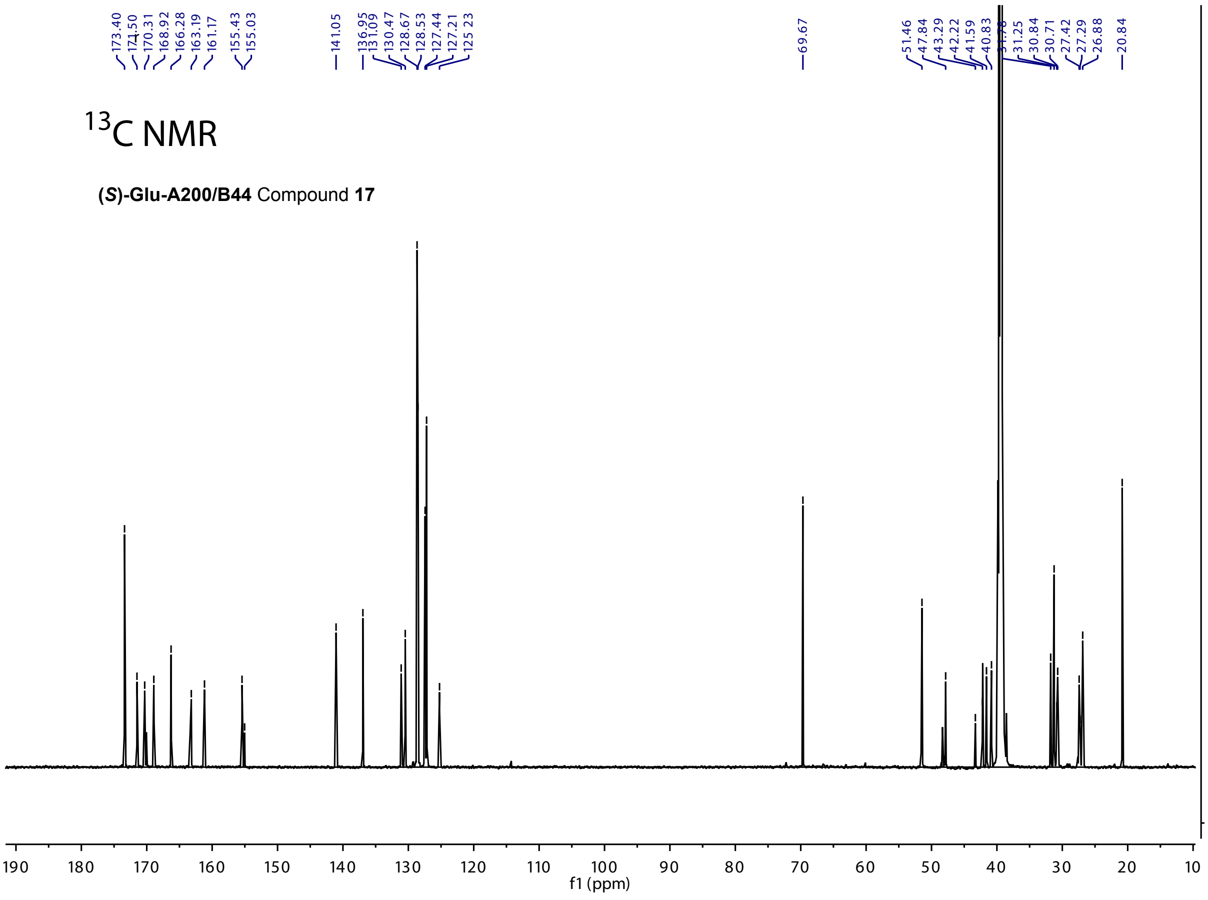

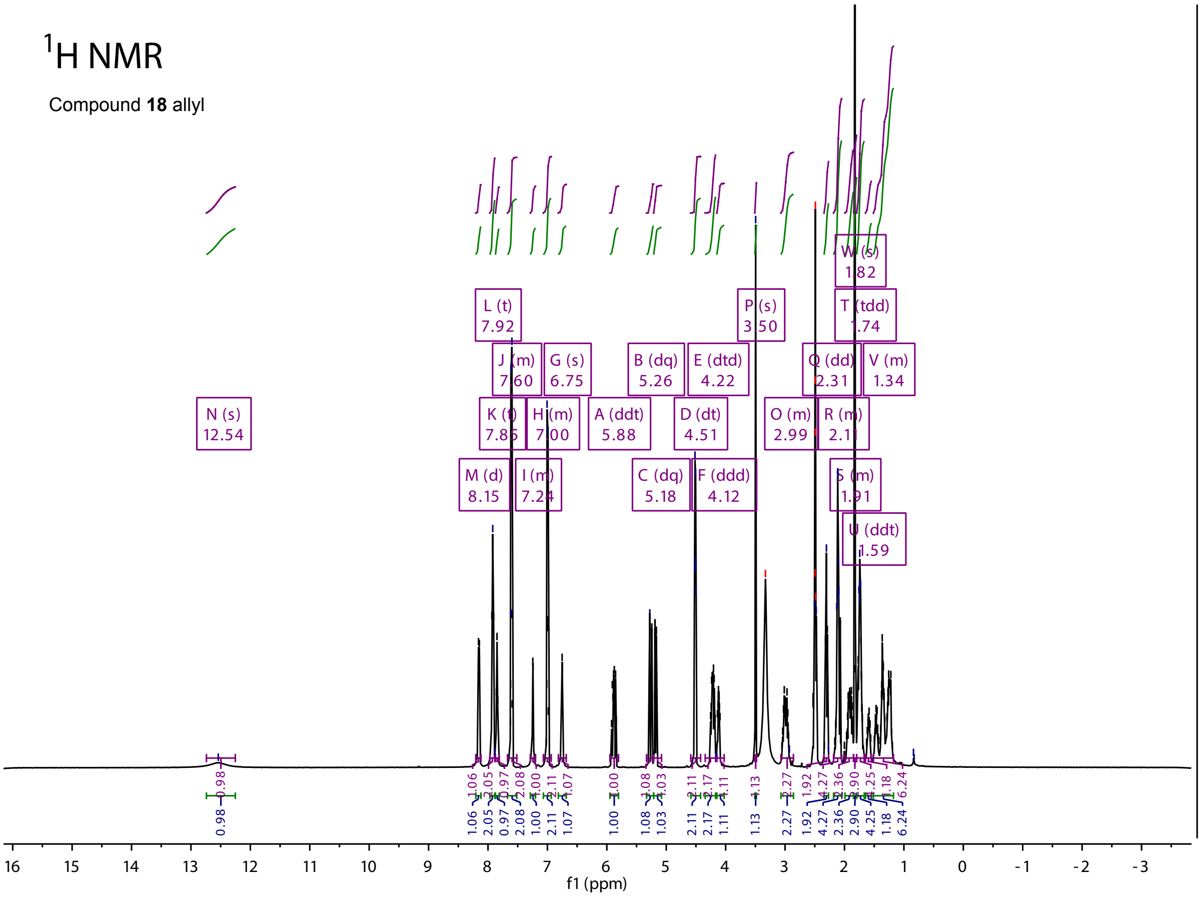

1H NMR
Compound 18 allyl
N (s) 12.54
M (d) 8.15
L (t) 7.92
K (t) 7.85
J (m) 7.60
I (m) 7.24
H (m) 7.00
G (s) 6.75
A (ddt) 5.88
B (dq) 5.26
C (dq) 5.18
D (dt) 4.51
E (dtd) 4.22
F (ddd) 4.12
P (s) 3.50
O (m) 2.99
Q (dd) 2.31
R (m) 2.11
S (m) 1.91
W (s) 1.82
T (tdd) 1.74
U (ddt) 1.59
V (m) 1.34
f1 (ppm)

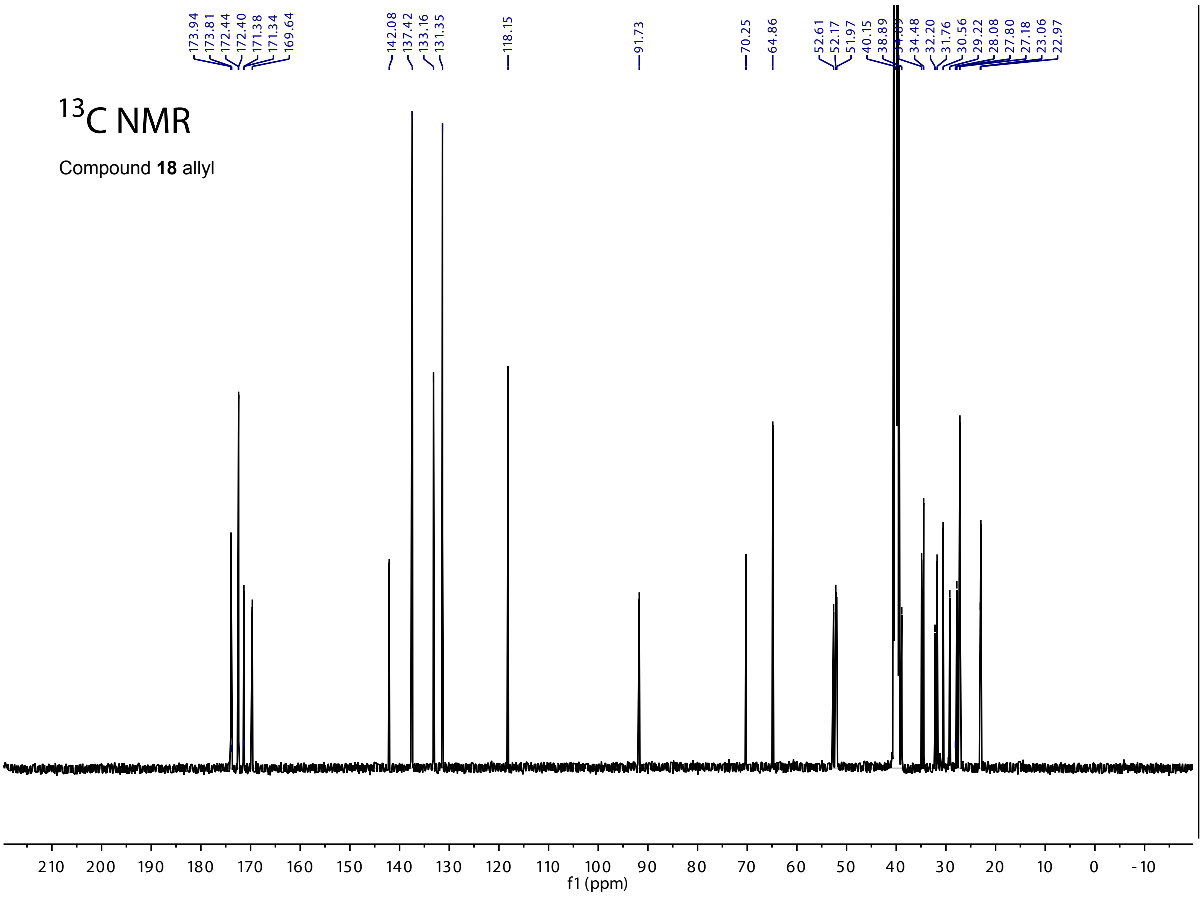

13C NMR
Compound 18 allyl
173.94
173.81
172.44
172.40
171.38
171.34
169.64
142.08
137.42
133.16
131.35
118.15
91.73
70.25
64.86
52.61
52.17
51.97
40.15
38.89
34.48
32.20
31.76
30.56
29.22
28.08
27.80
27.18
23.06
22.97
f1 (ppm)

^{1}H NMR

Compound **19** Allyl

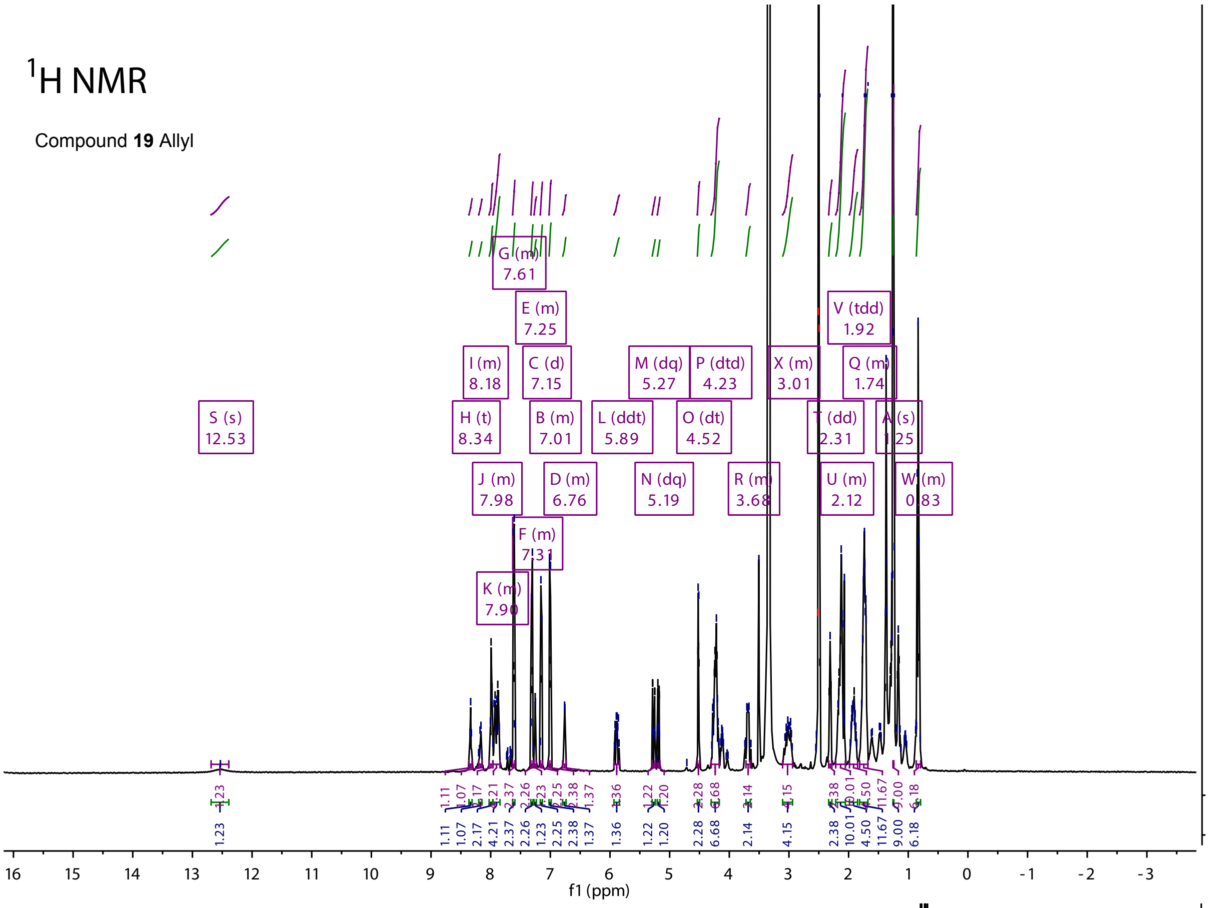

^{13}C NMR

Compound **19** Allyl

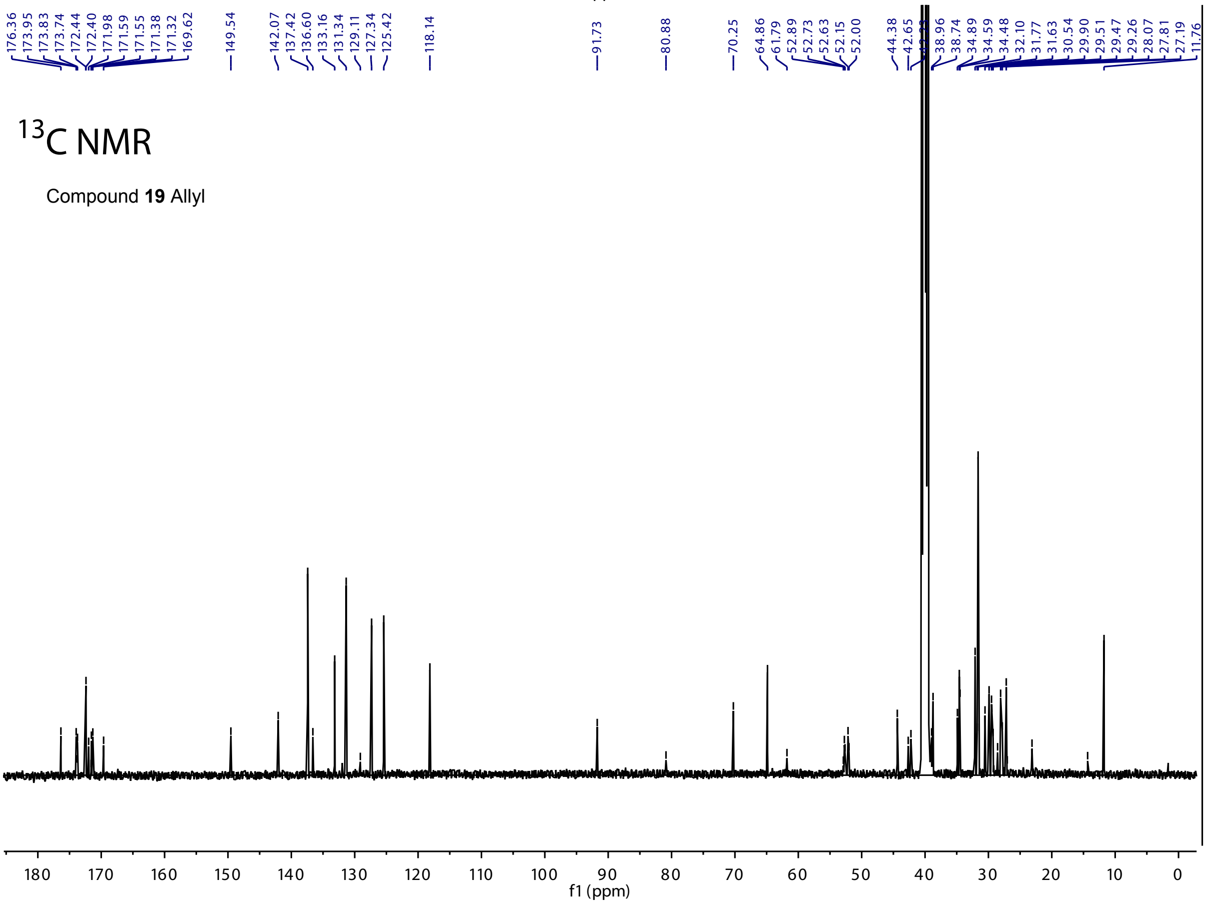

14. Appendix II: LC-MS data

DB24-HIT1

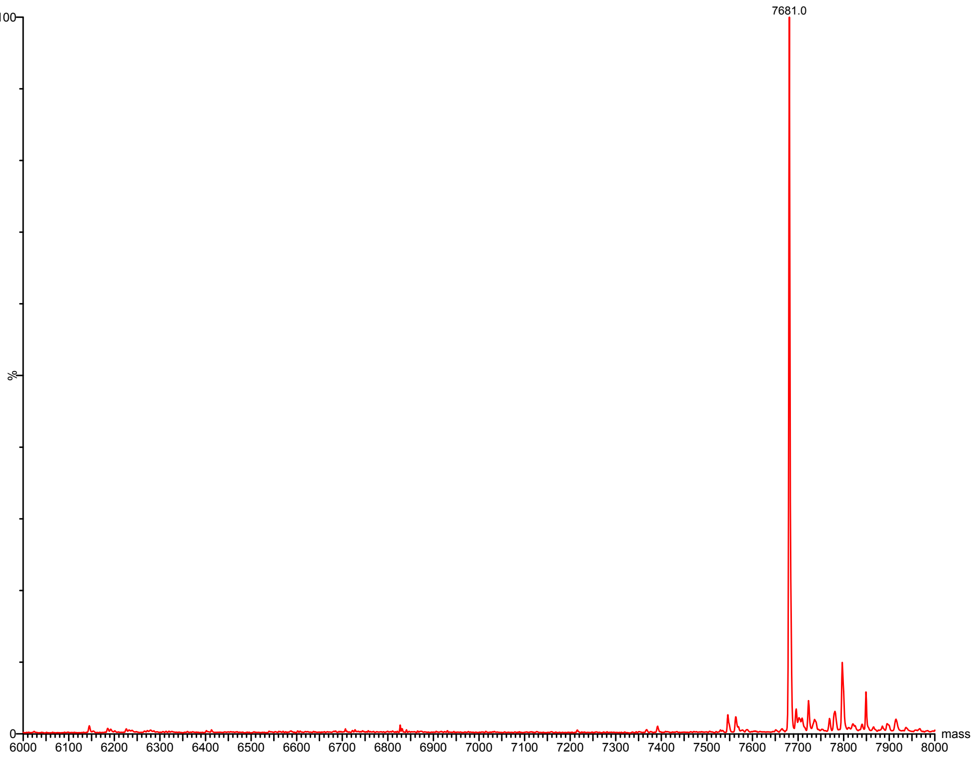

DB24-HIT2

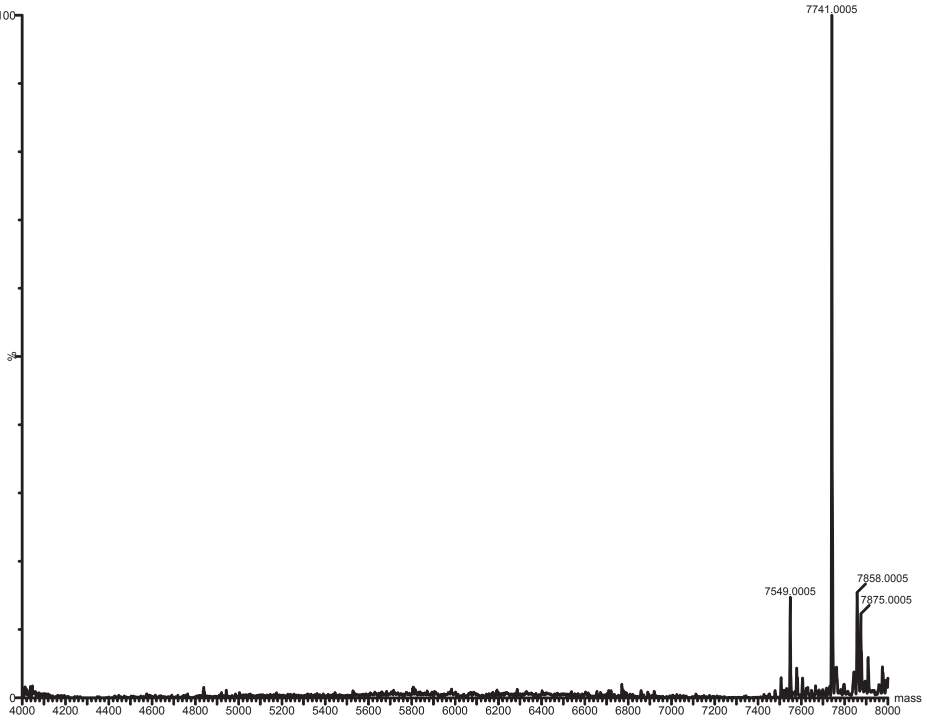

DB24-HIT3

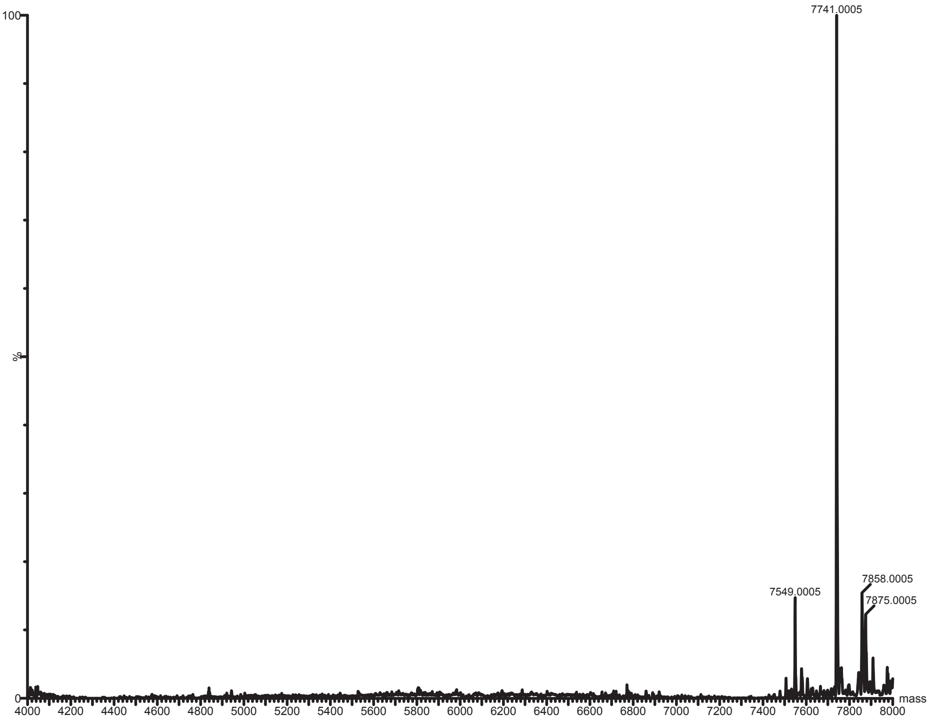

DB24-HIT4

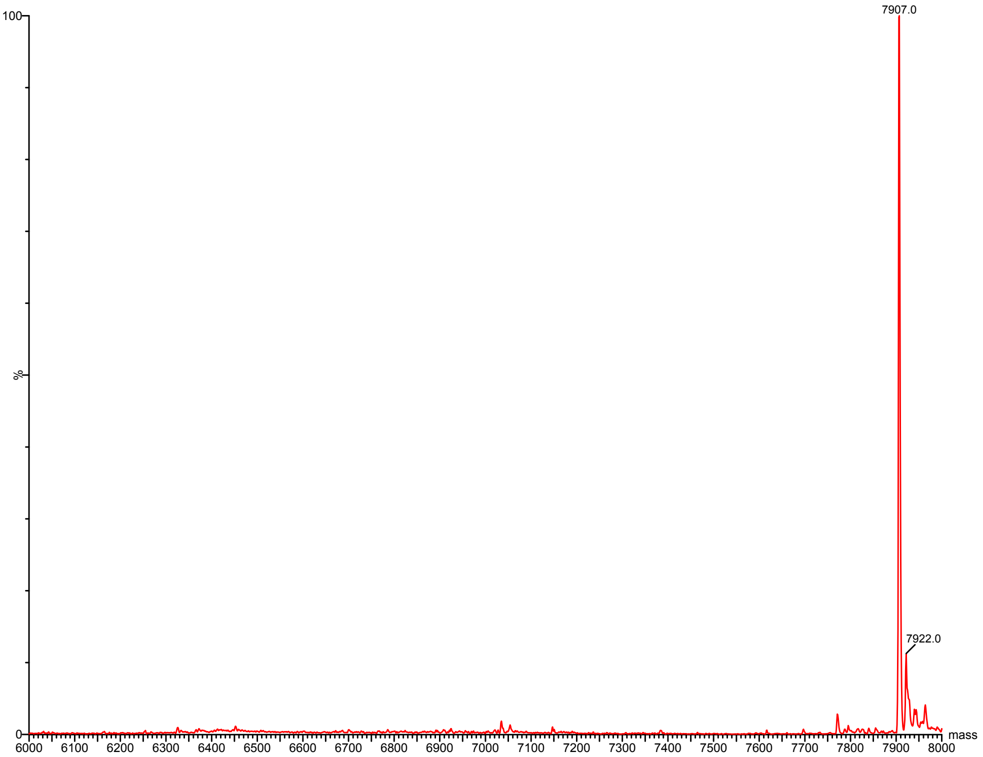

DB24-HIT5

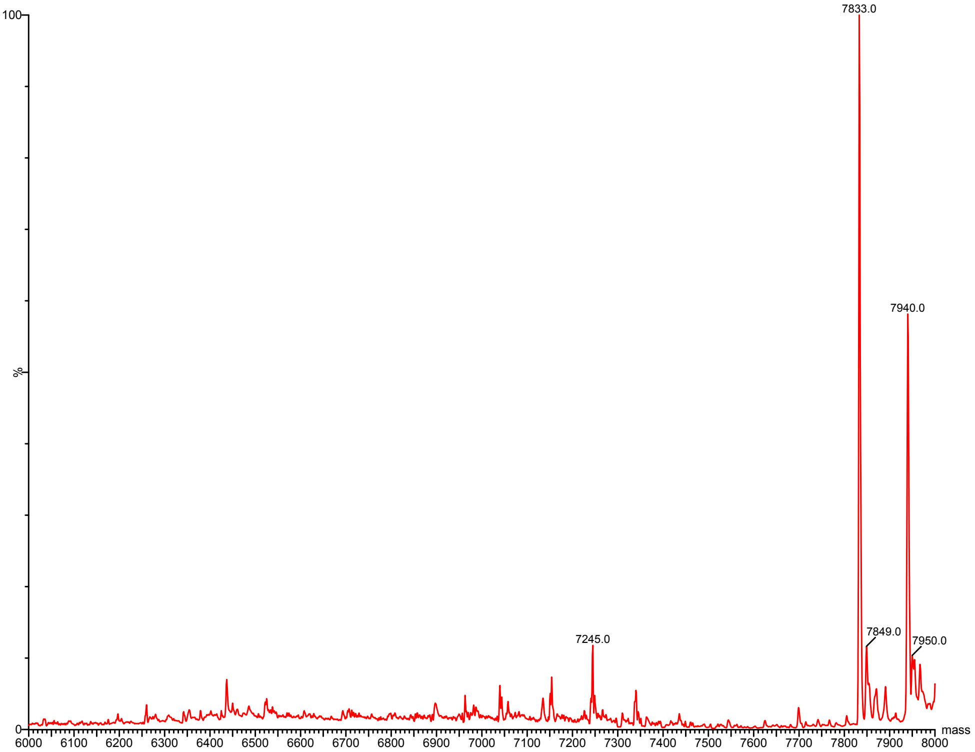

100
%
0
7833.0
7940.0
7245.0
7849.0
7950.0
6000
6100
6200
6300
6400
6500
6600
6700
6800
6900
7000
7100
7200
7300
7400
7500
7600
7700
7800
7900
8000
mass

(*R)*-Glu-A8/B264 Compound **4**

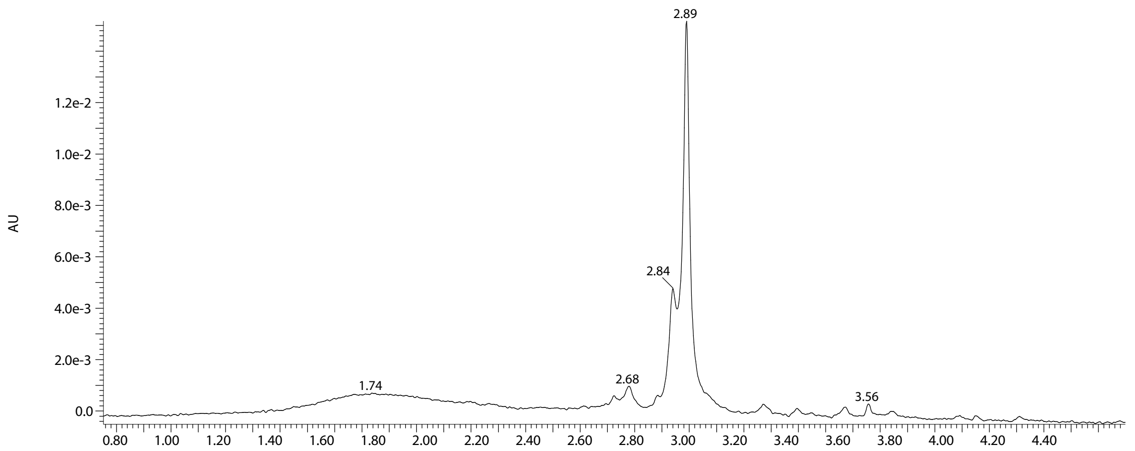

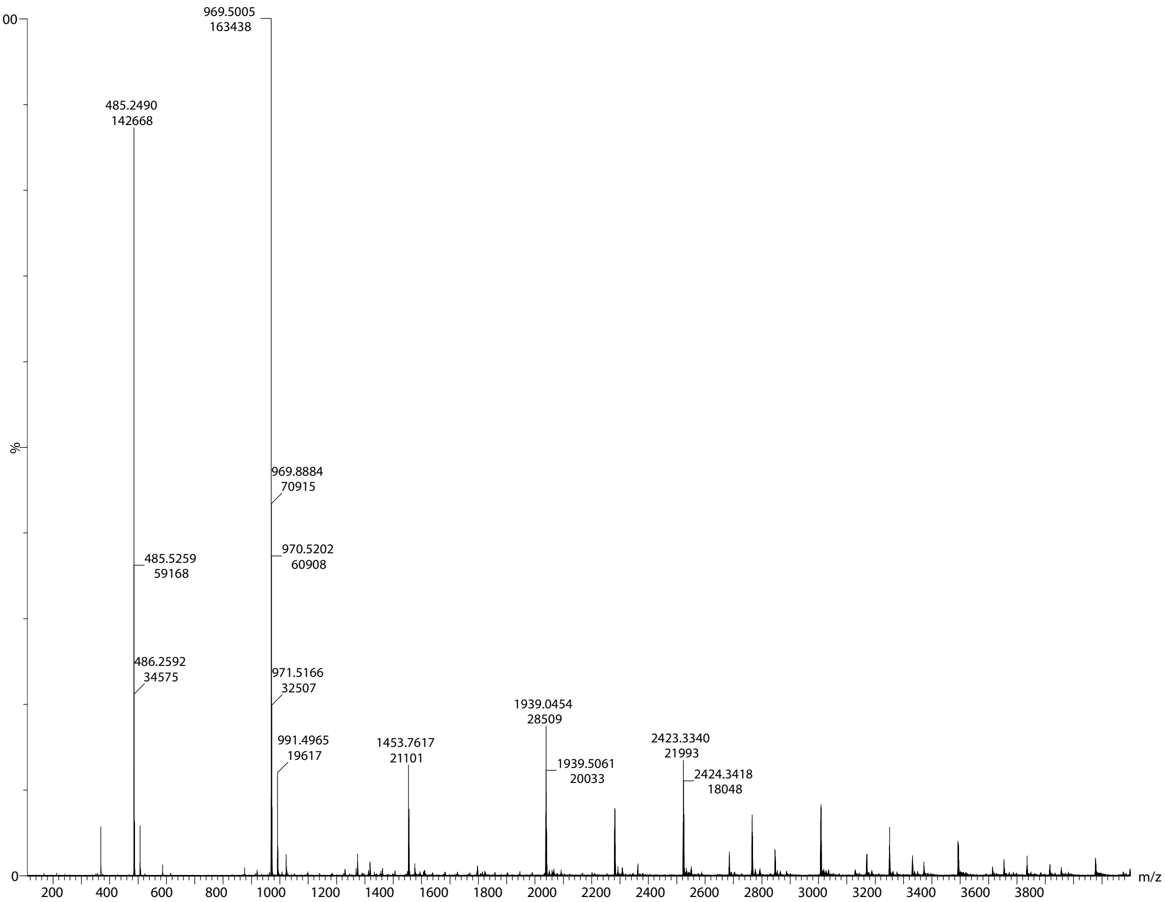

(*R)*-Glu-A8/B264 Compound **4 fluo**

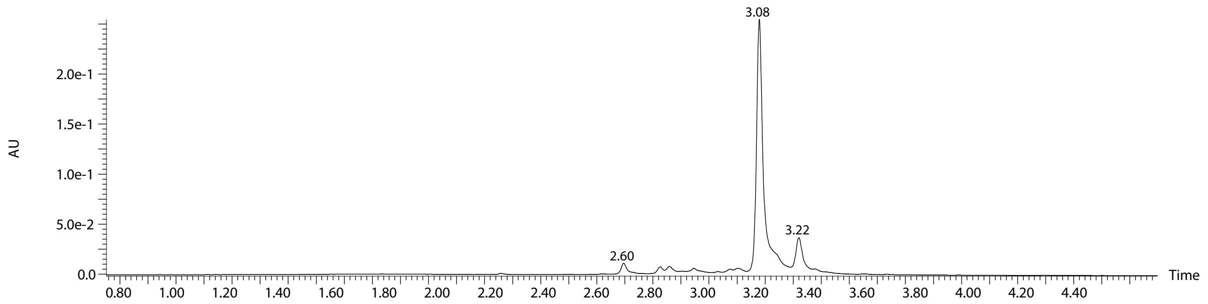

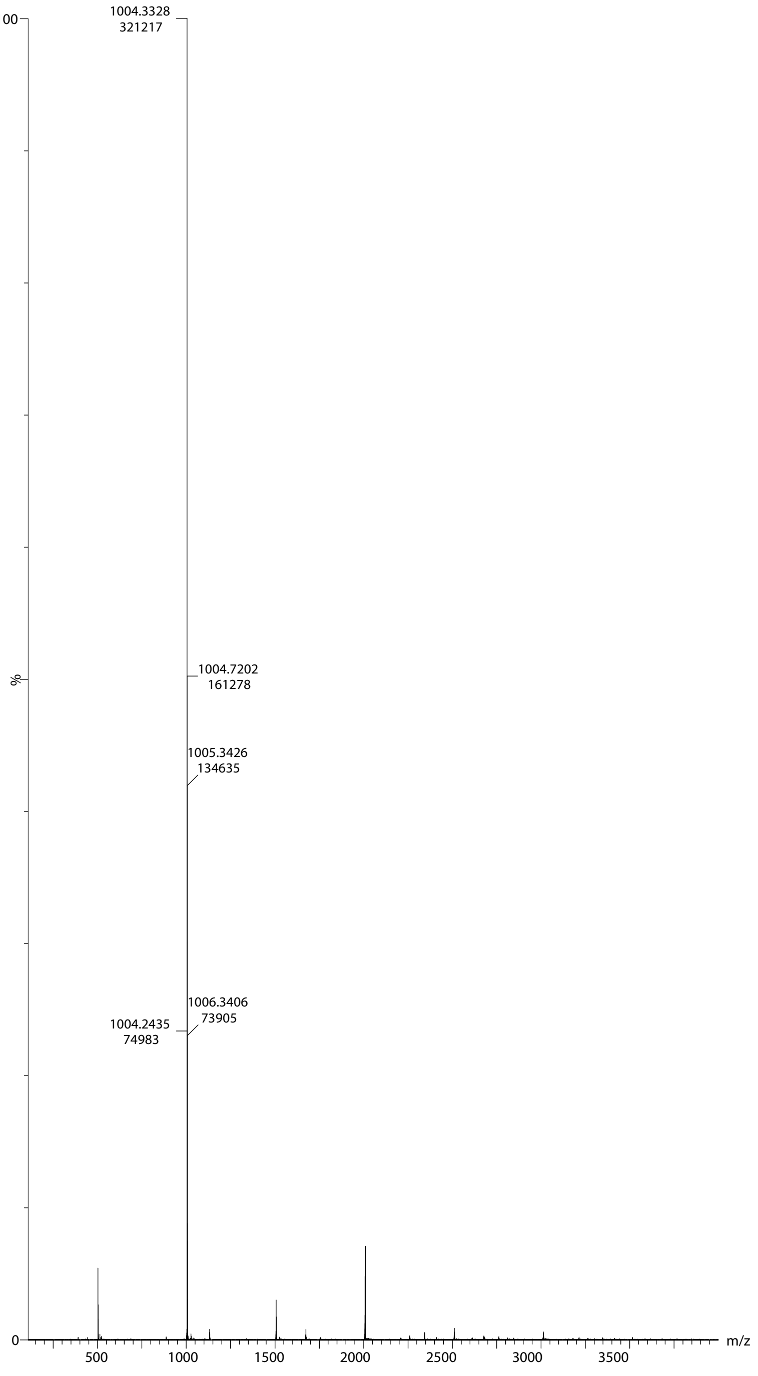

(*R*)-Glu-A8/B264 Compound **5**

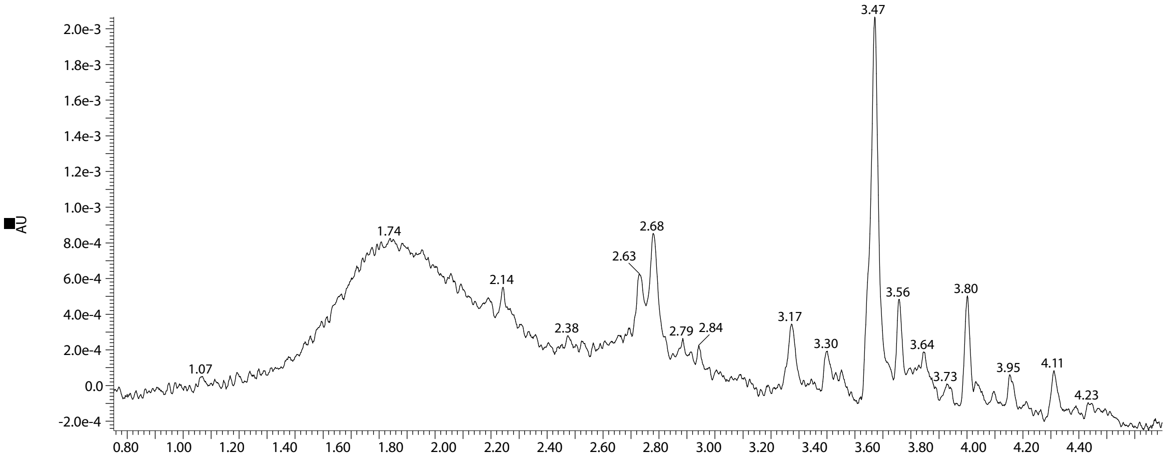

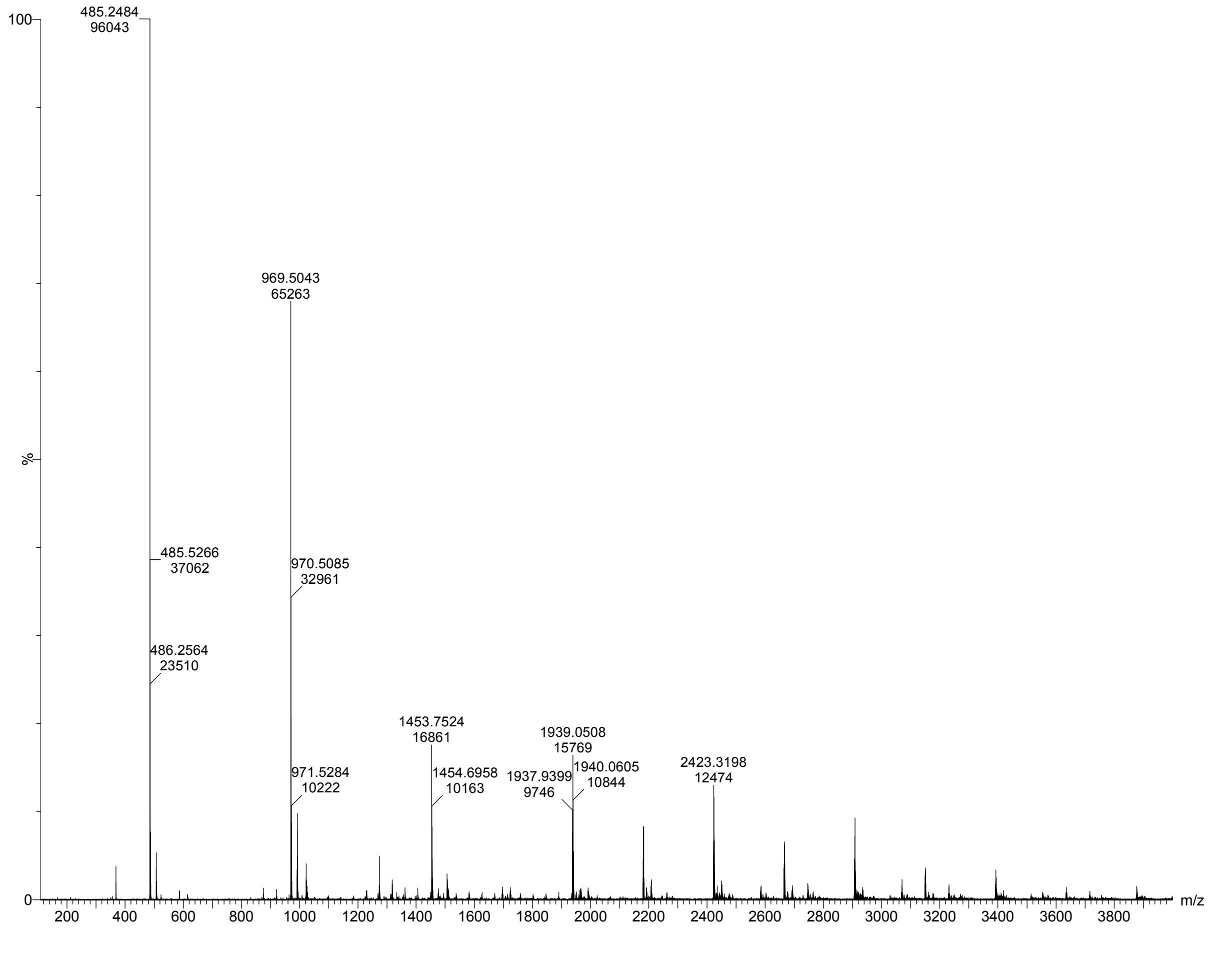

(*S*)-Glu-A8/B264 Compound **5 fluo**

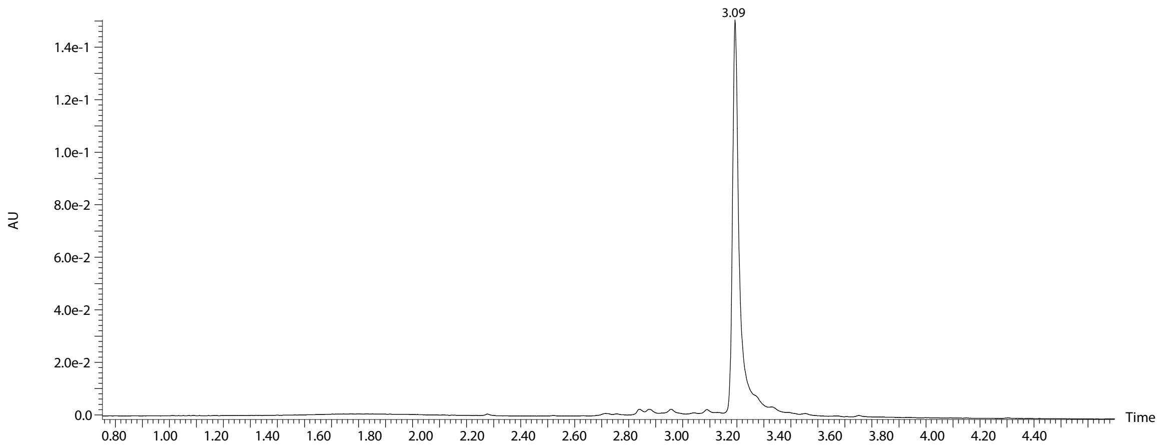

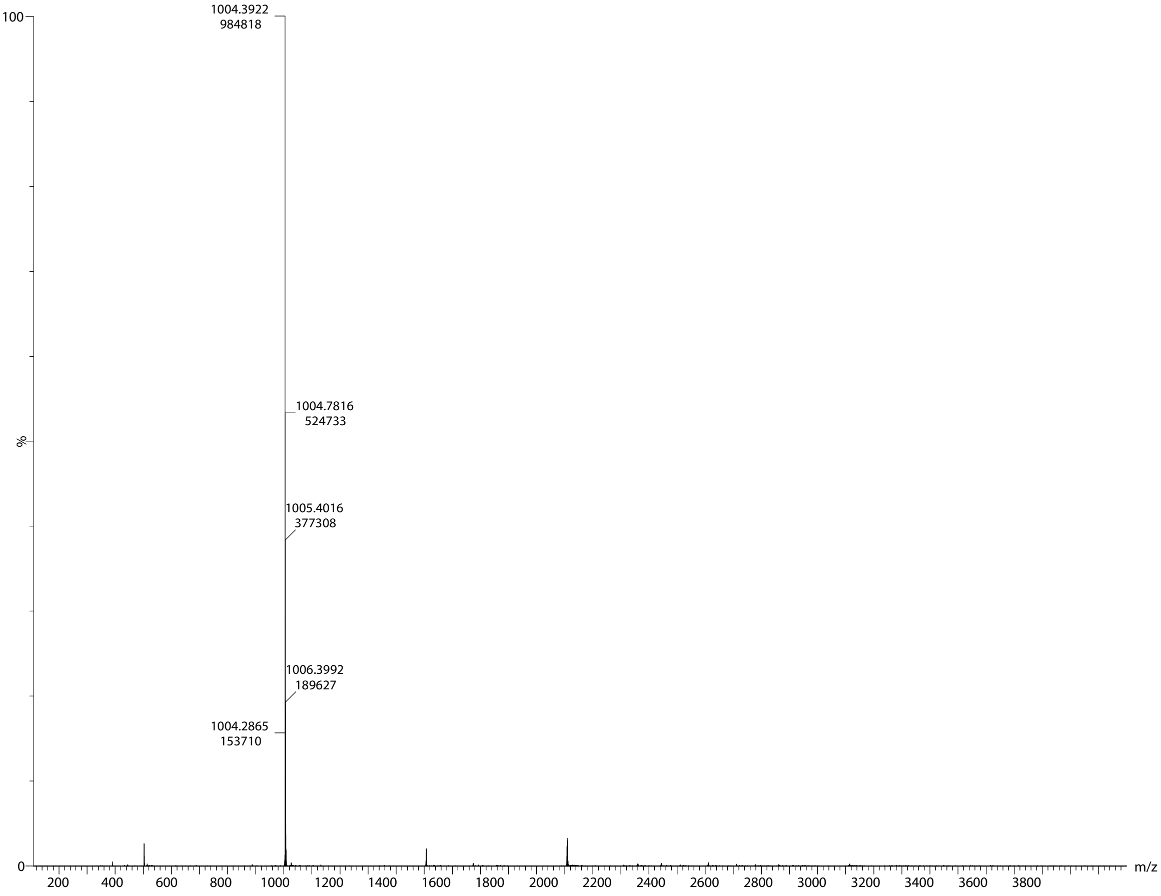

(***R***)**-Glu-A20/B611** Compound **6**

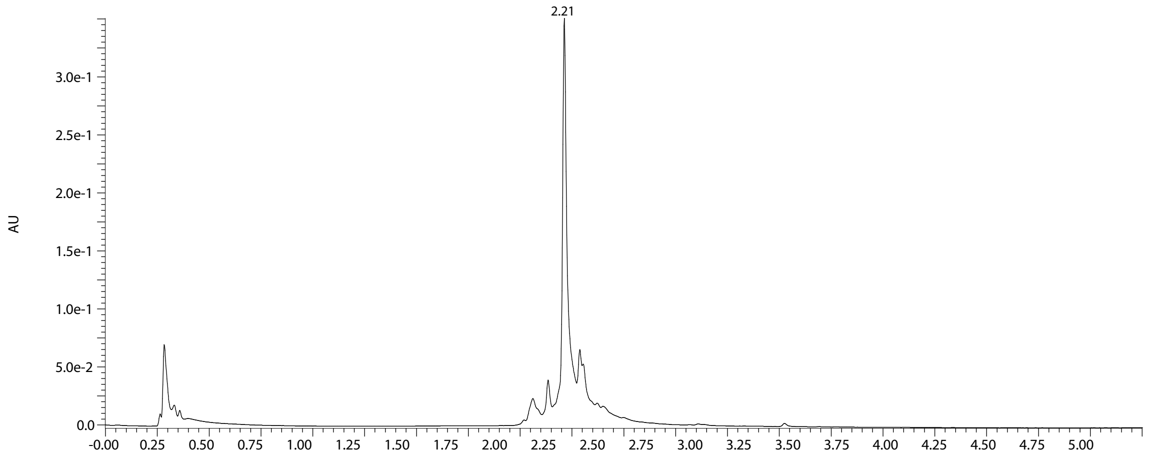

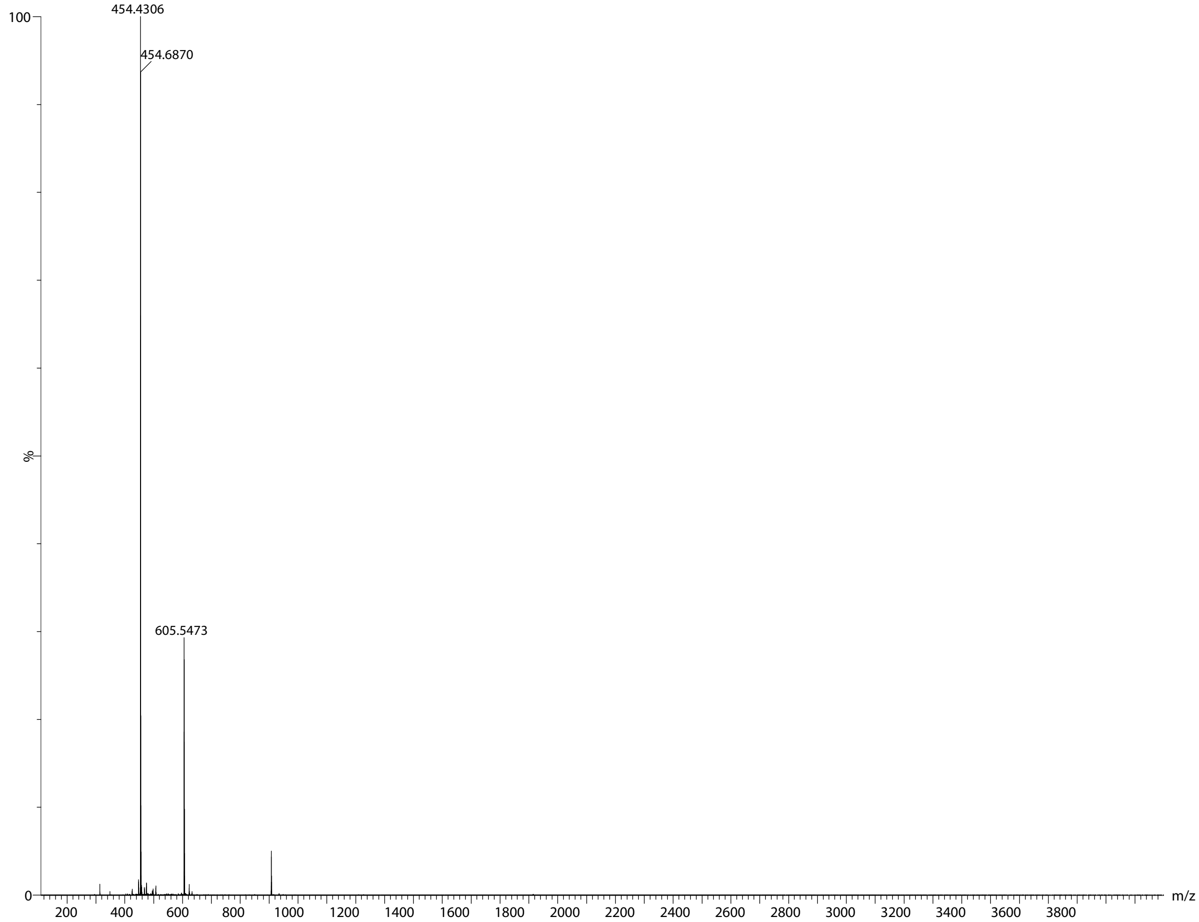

(*R*)-Glu-A20/B611 Compound **6 fluo**

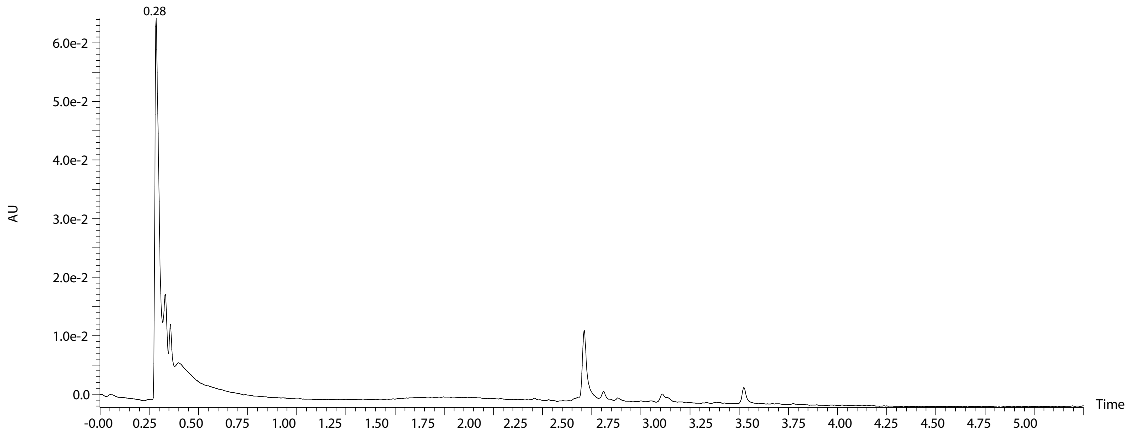

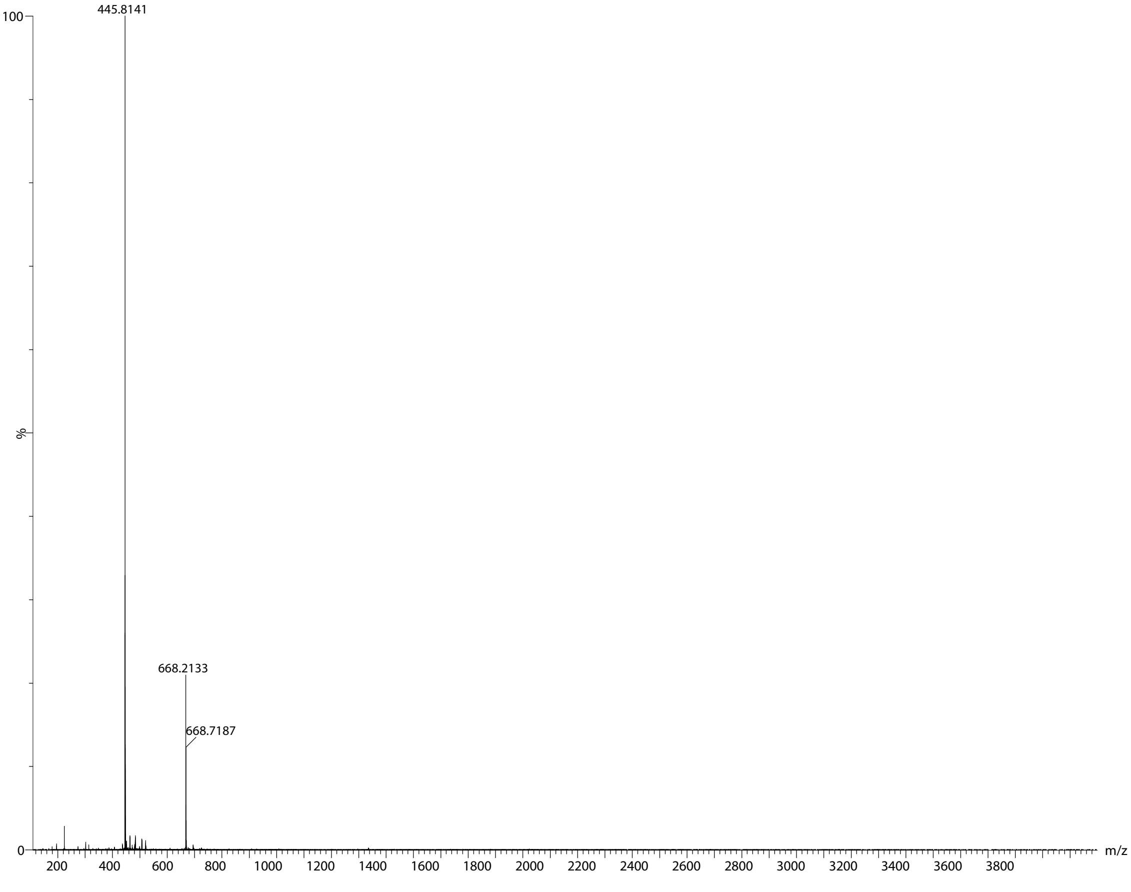

(*S*)-Glu-A20/B611 Compound **7**

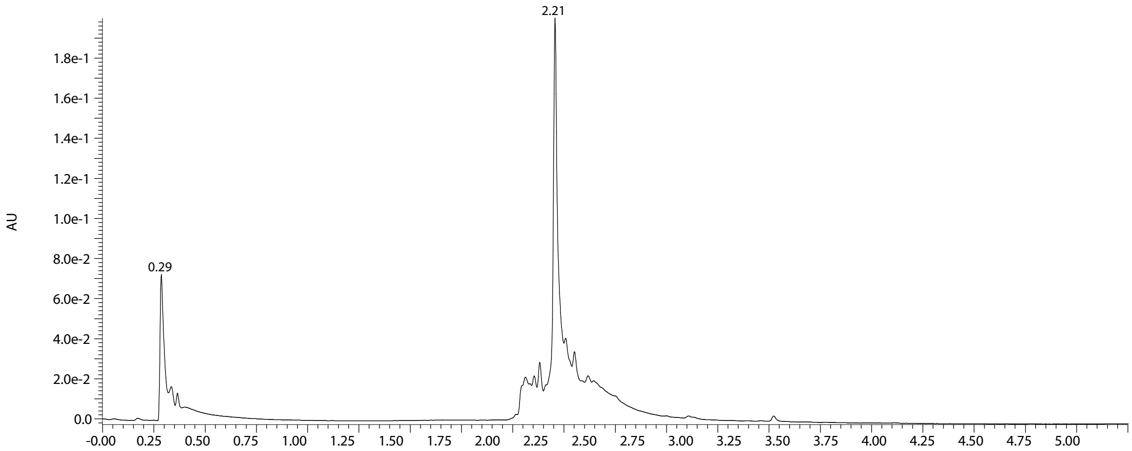

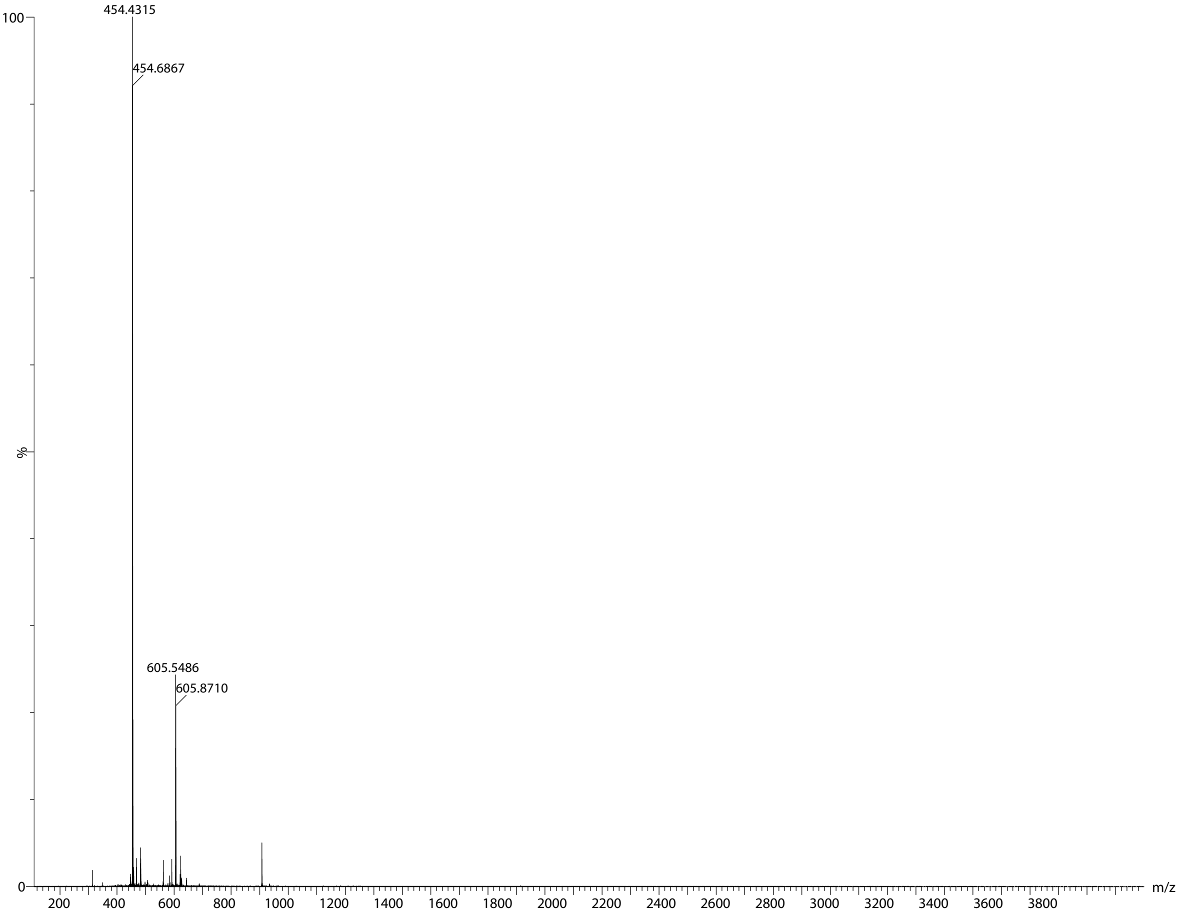

(*S*)-Glu-A20/B611 Compound **7 fluo**

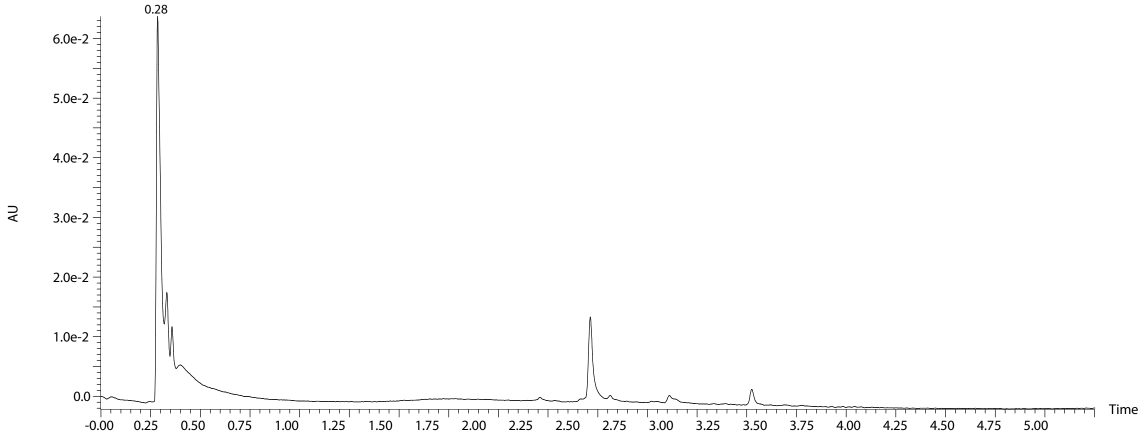

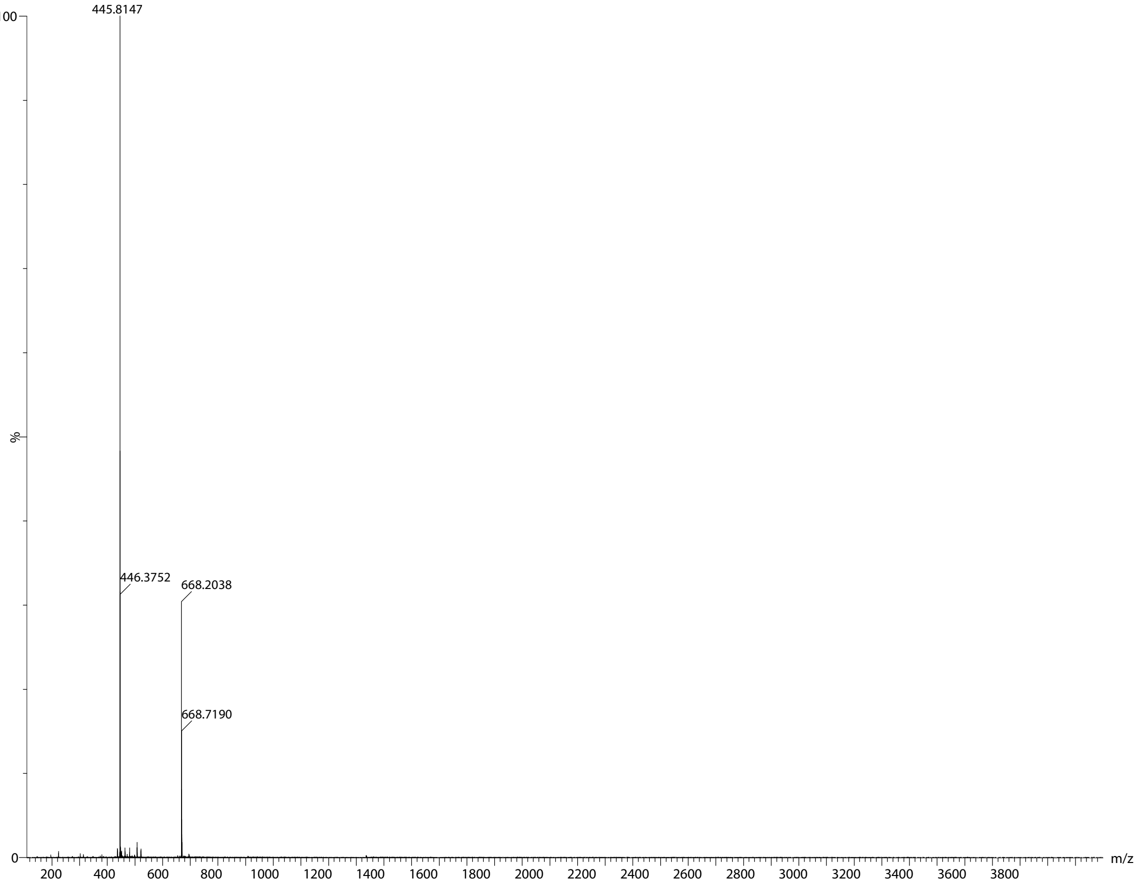

(R)-Glu-A120/B235 Compound **8**

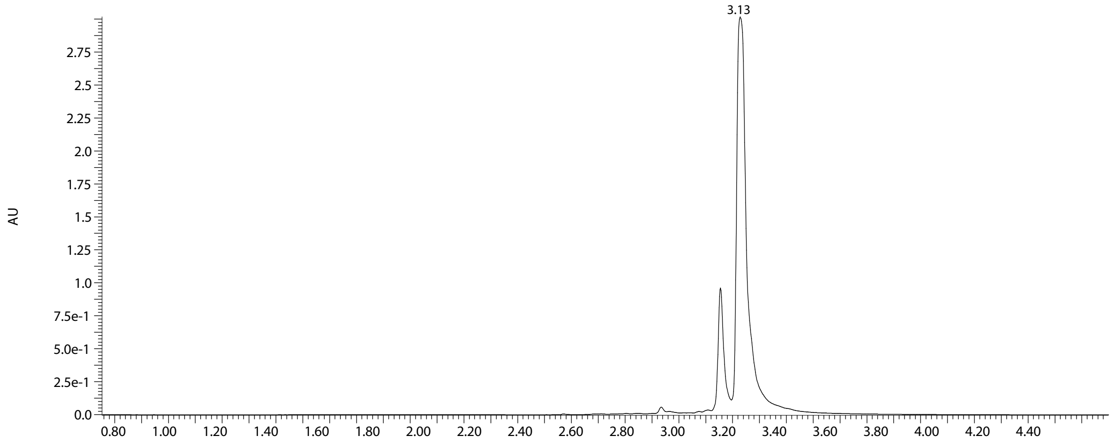

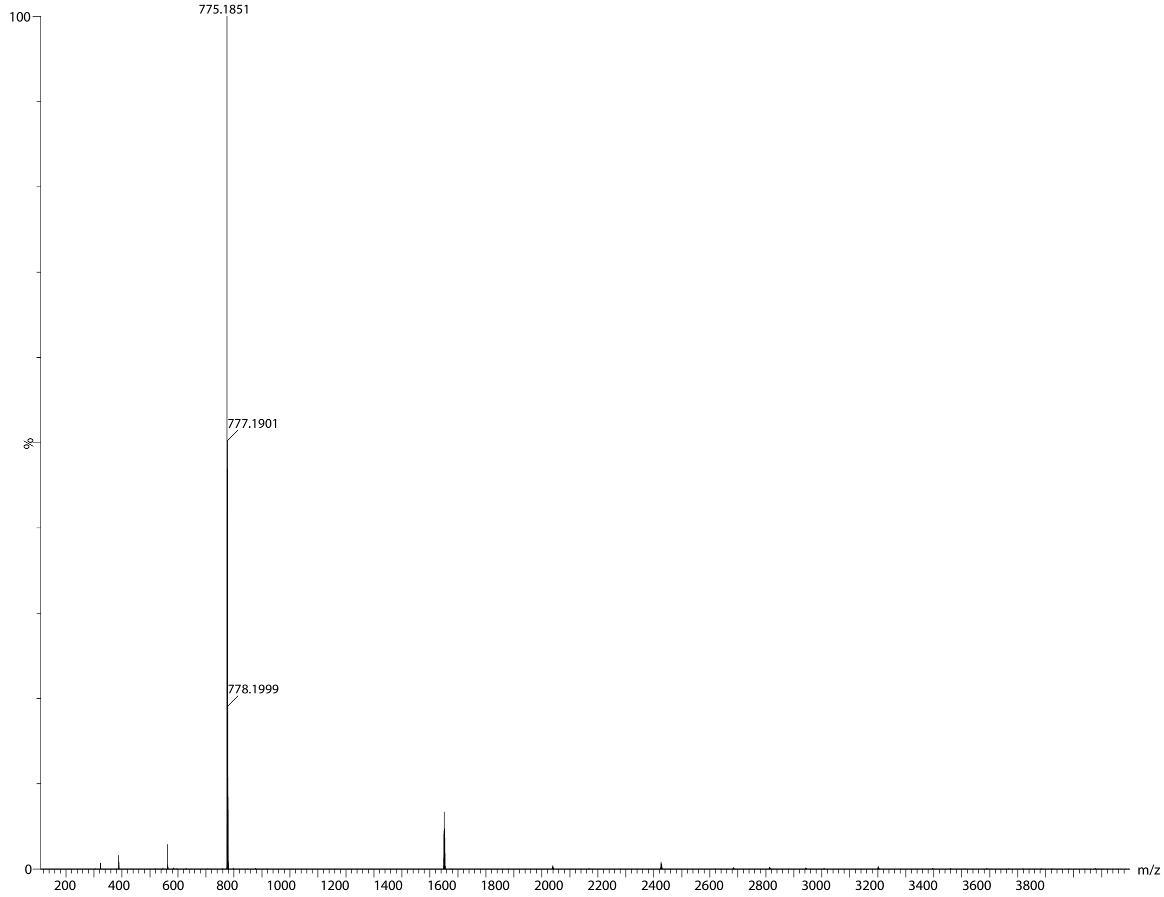

(R)-Glu-A120/B235 Compound **8 fluo**

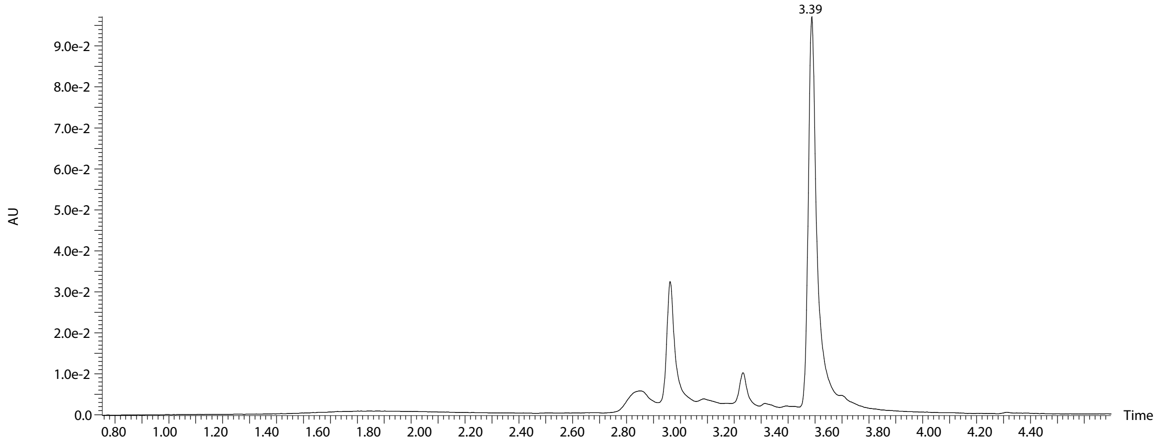

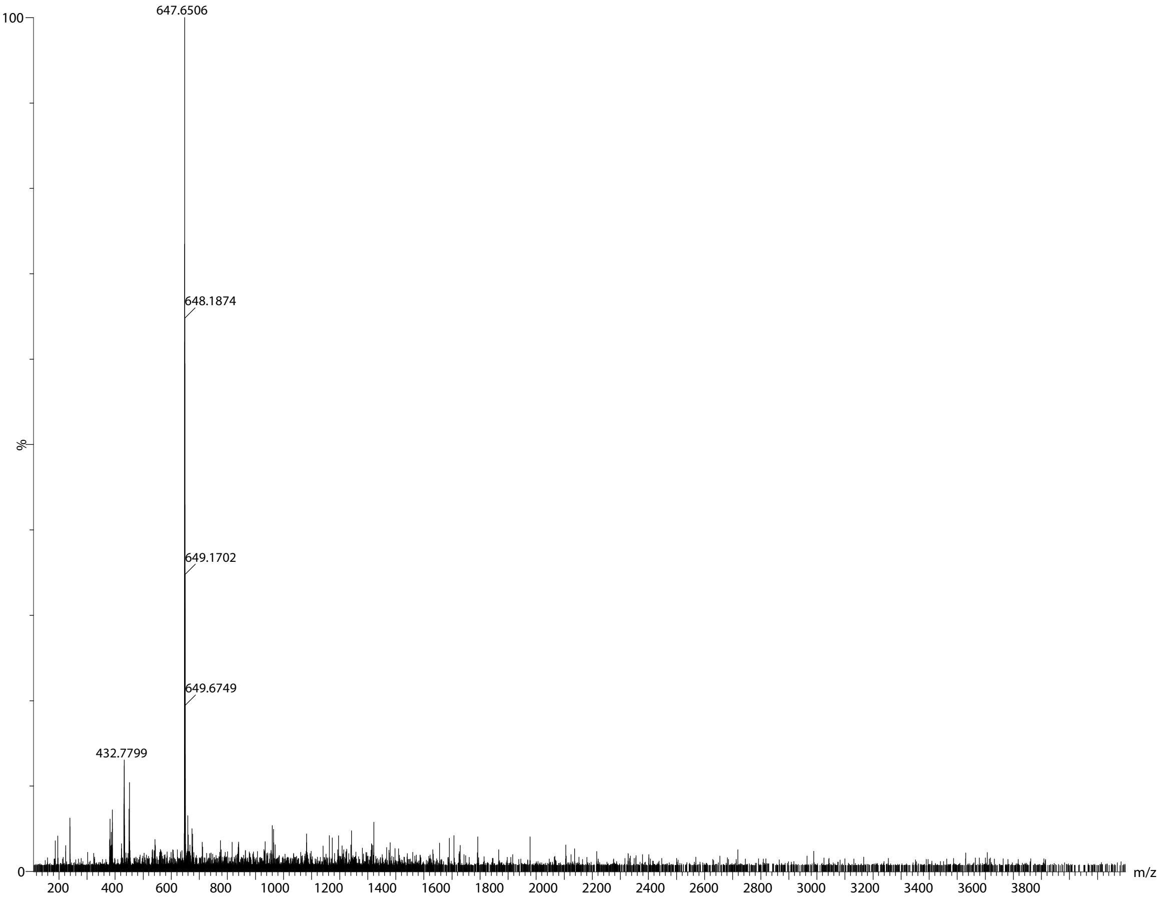

(S)-Glu-A120/B235 Compound **9**

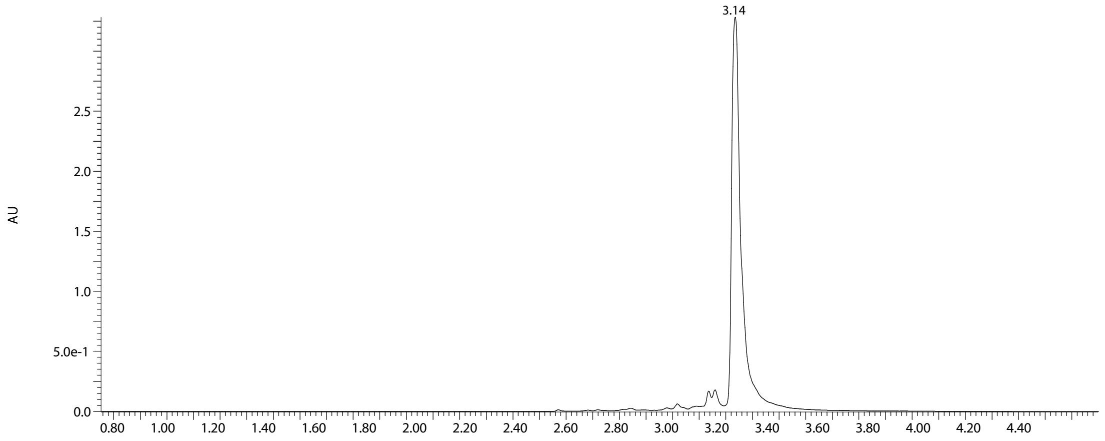

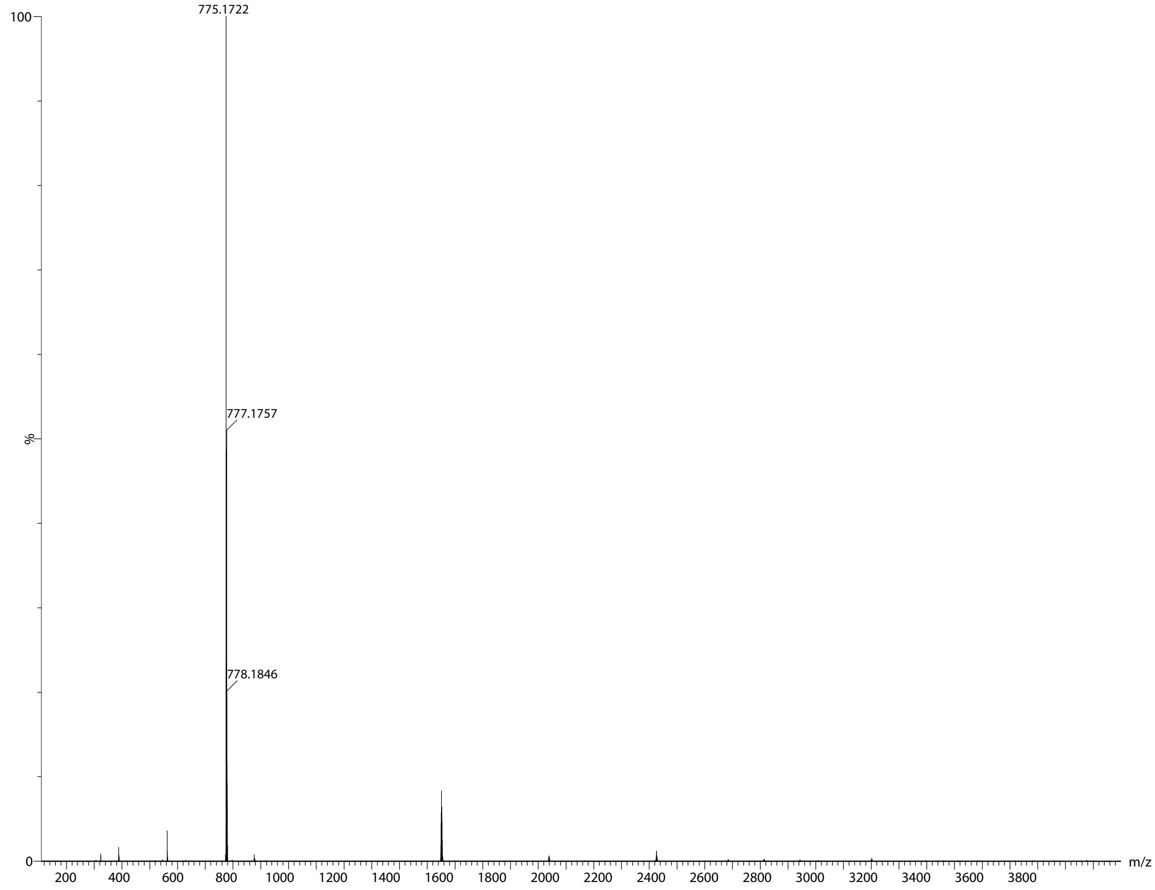

(S)-Glu-A120/B235 Compound **9 fluo**

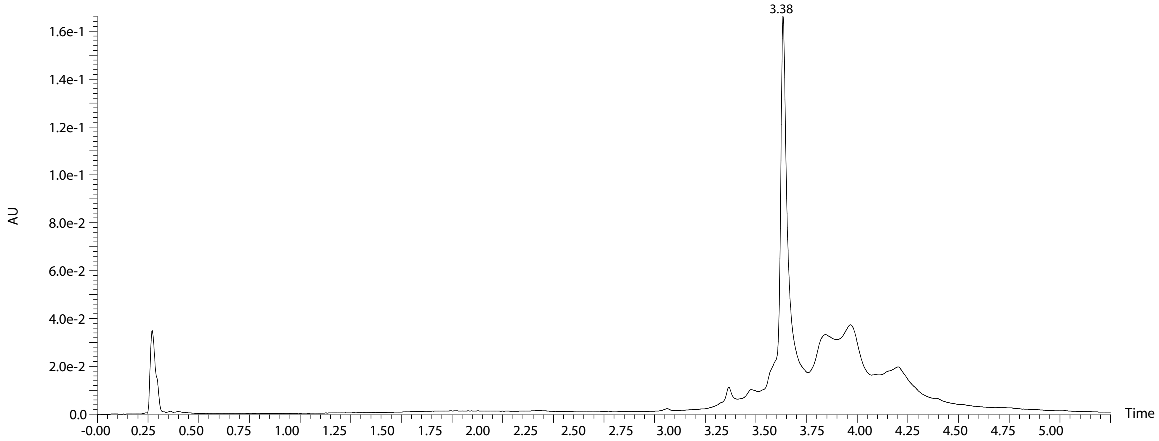

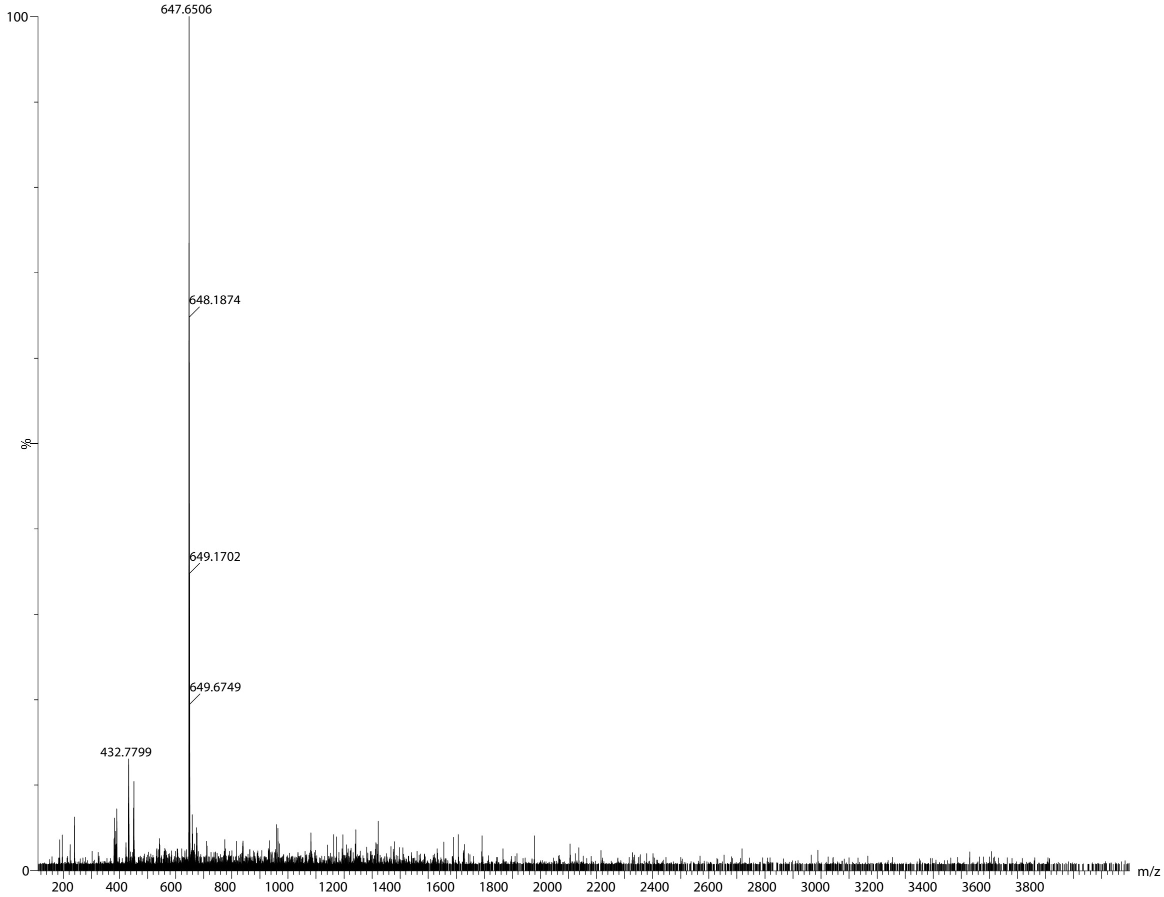

(*R*)-Glu-A86/B371 Compound **10**

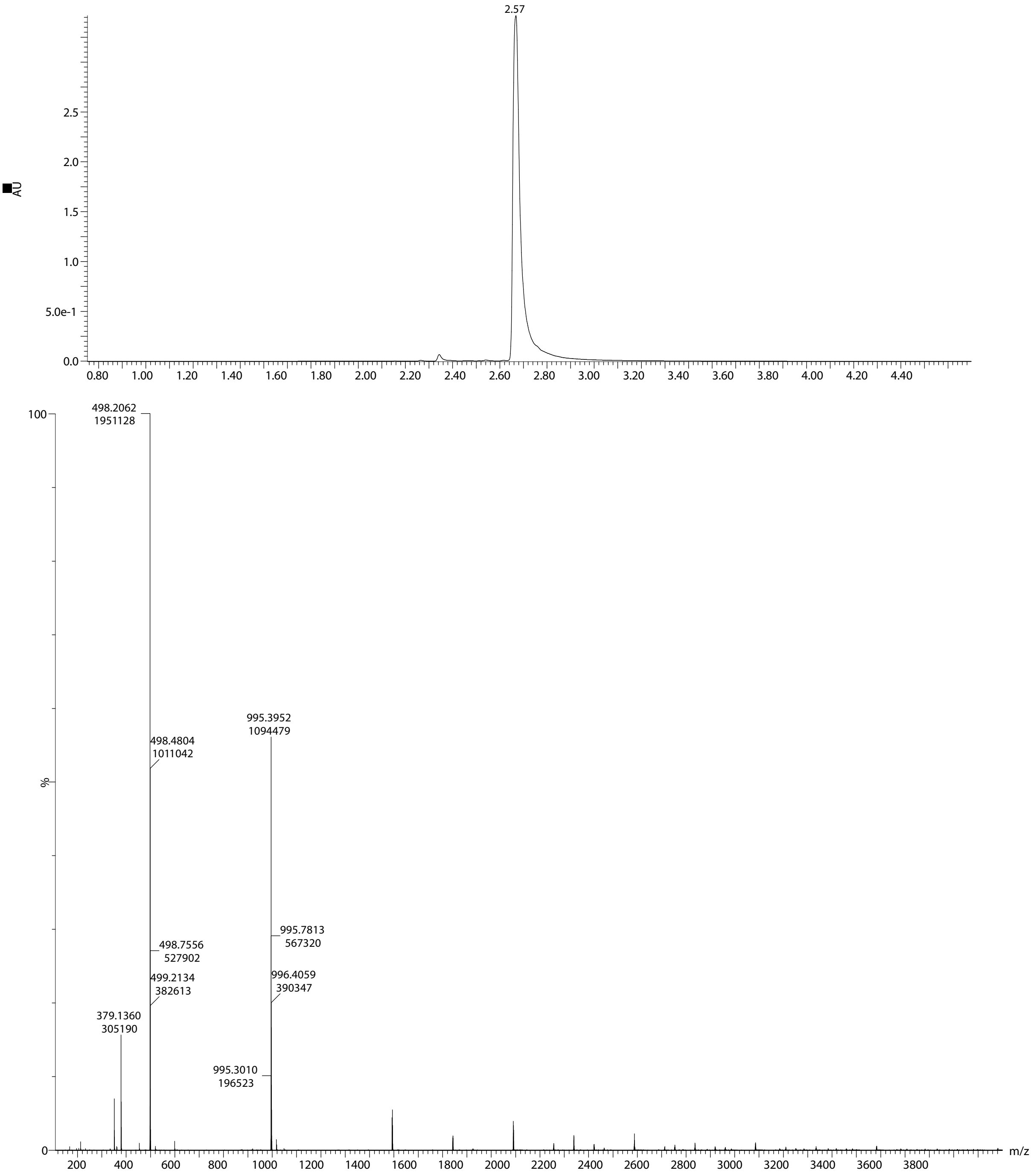

(*R*)-Glu-A86/B371 Compound **10 fluo**

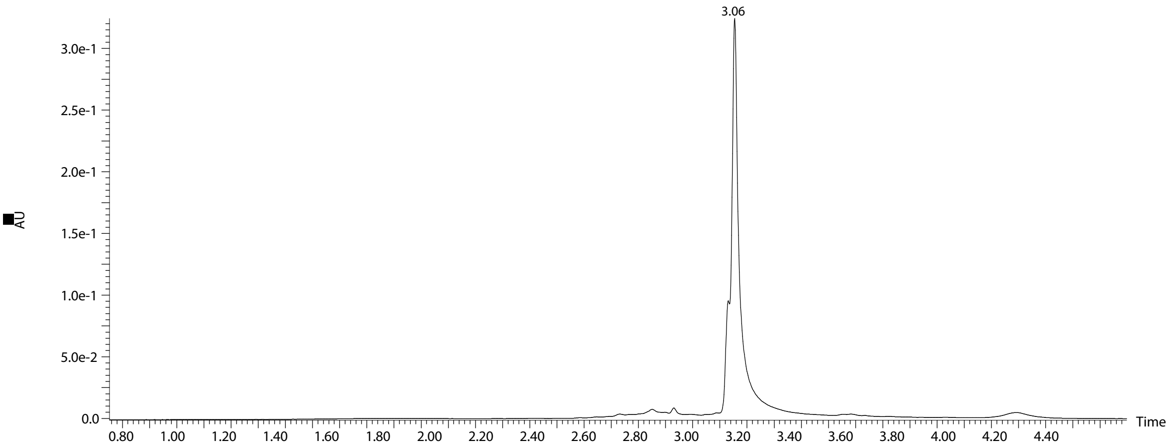

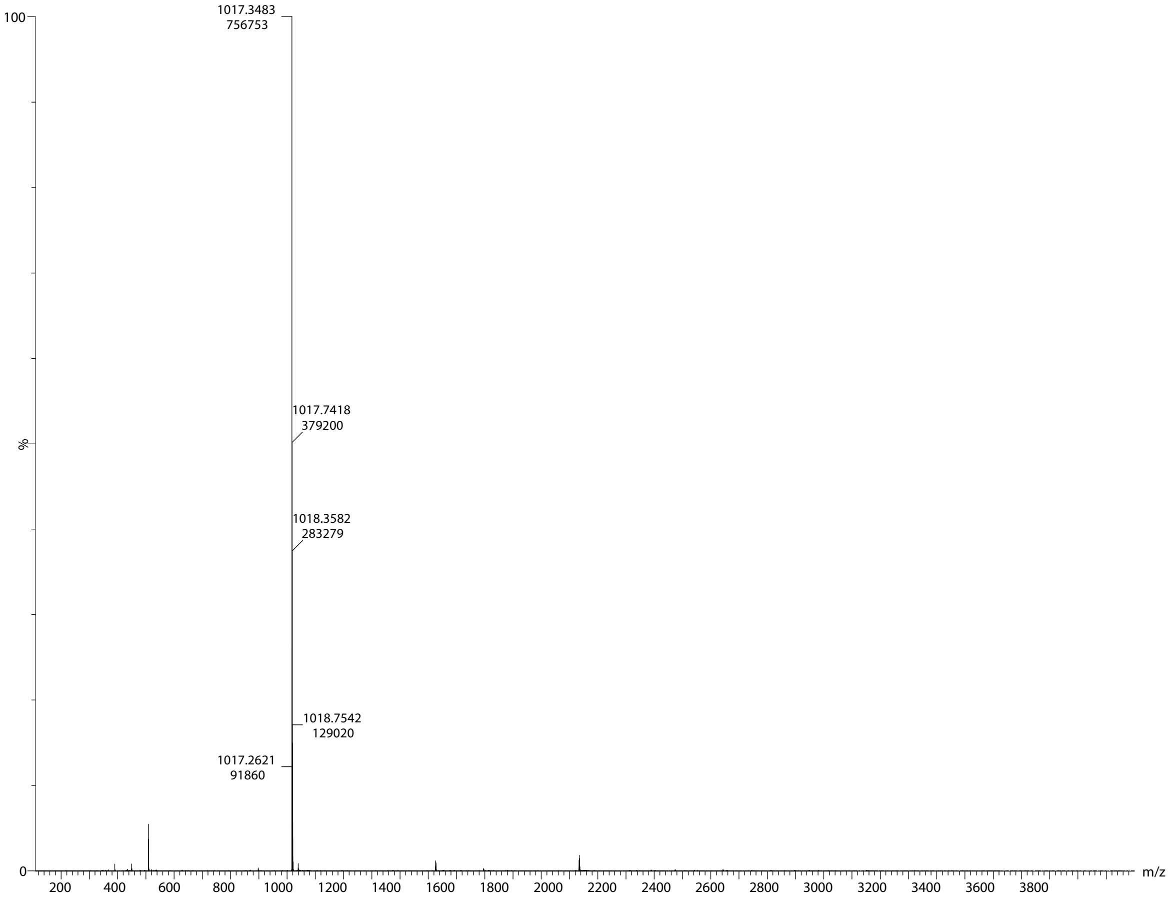

(***S***)**-Glu-A86/B371** Compound **11**

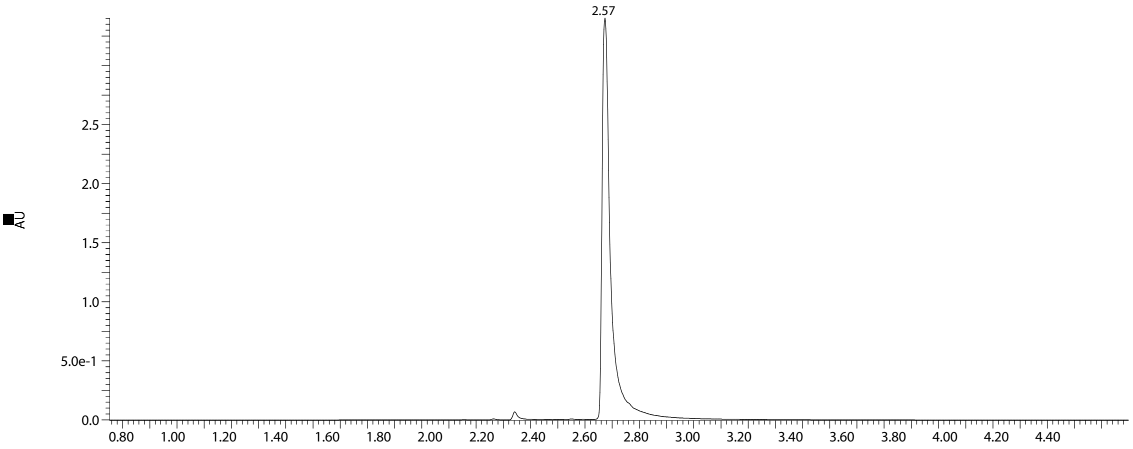

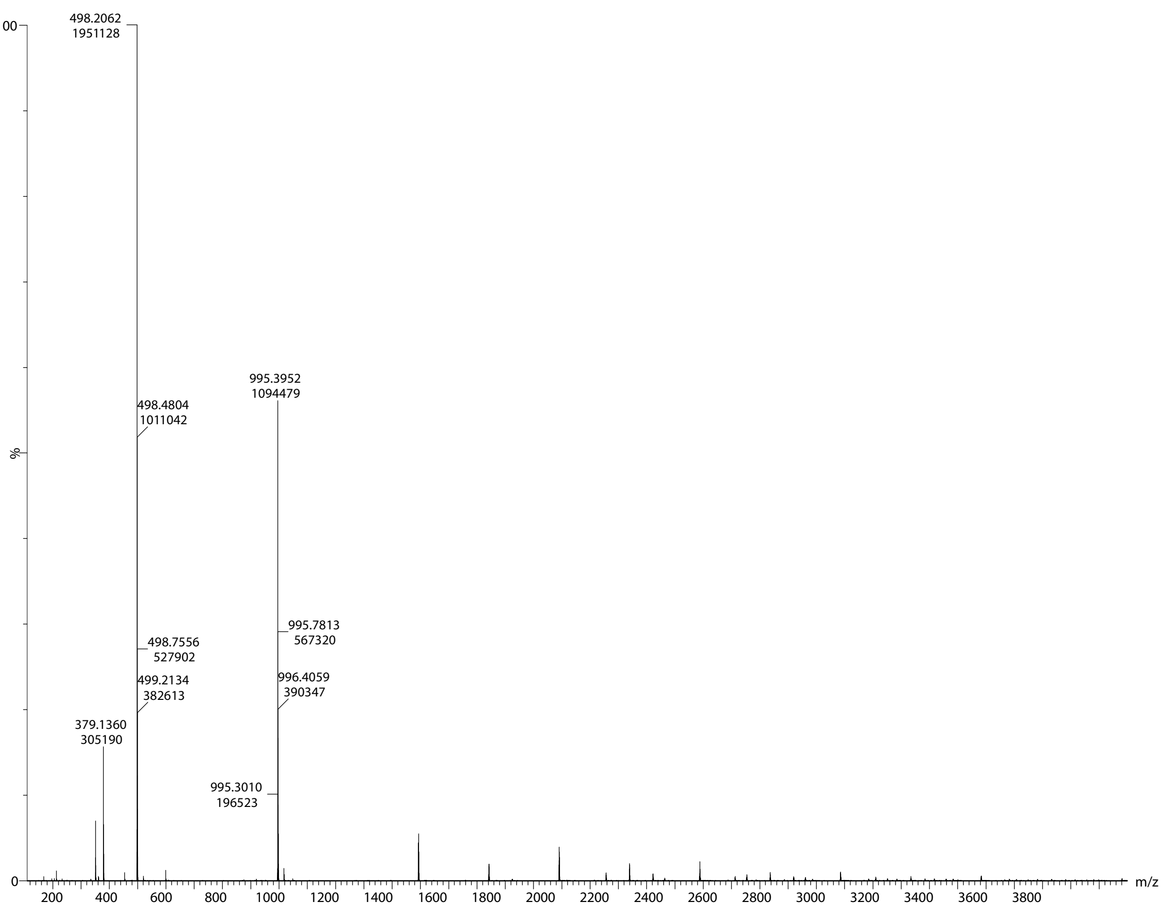

(*S*)-Glu-A86/B371 Compound **11 fluo**

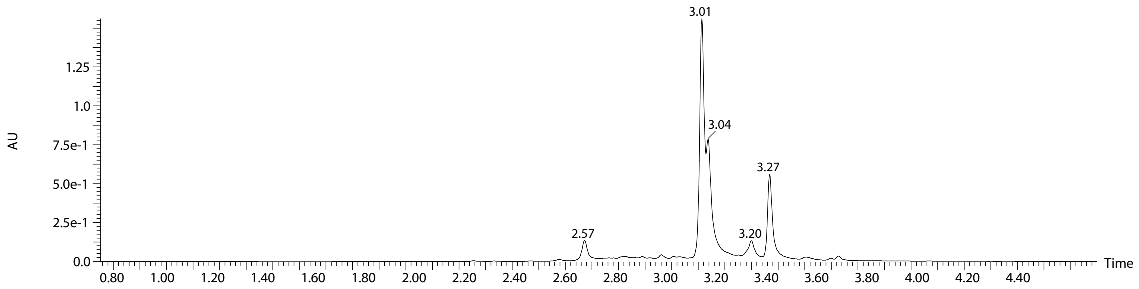

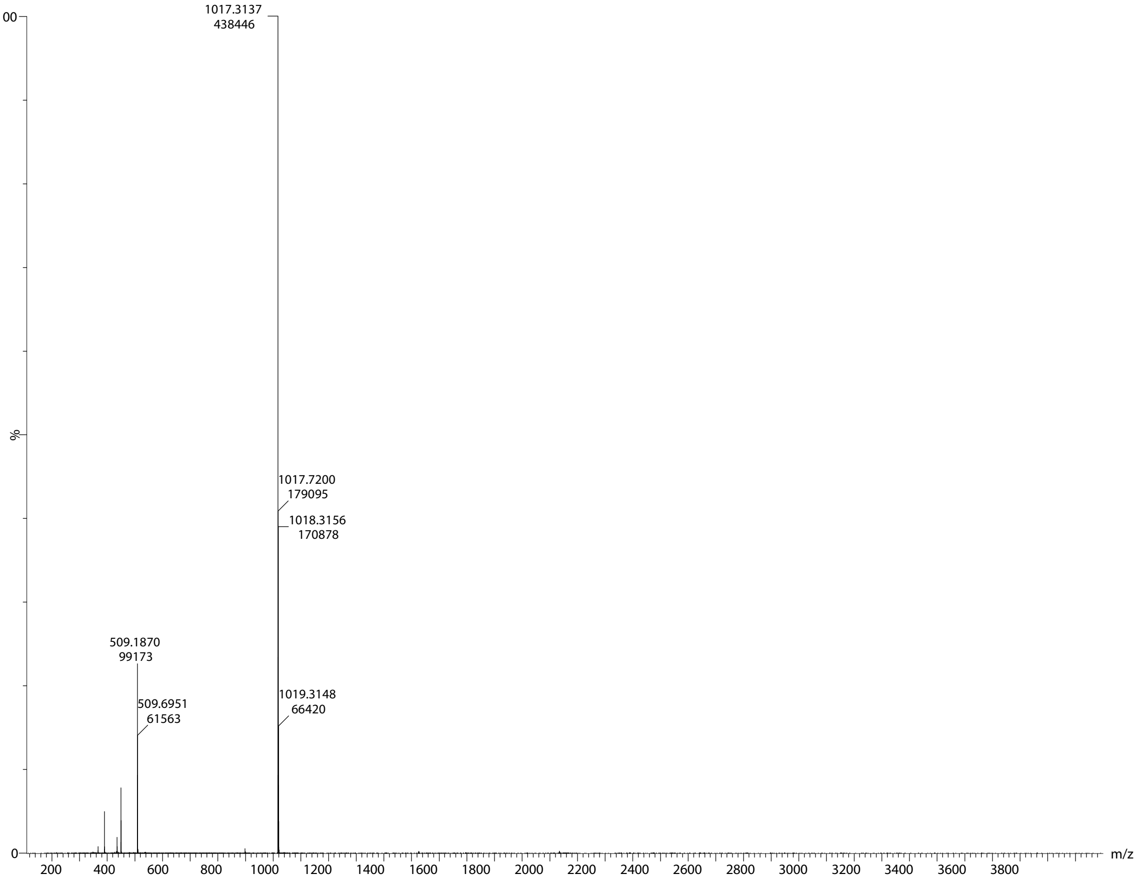

(*R*)-Glu-A146/B537 Compound **12**

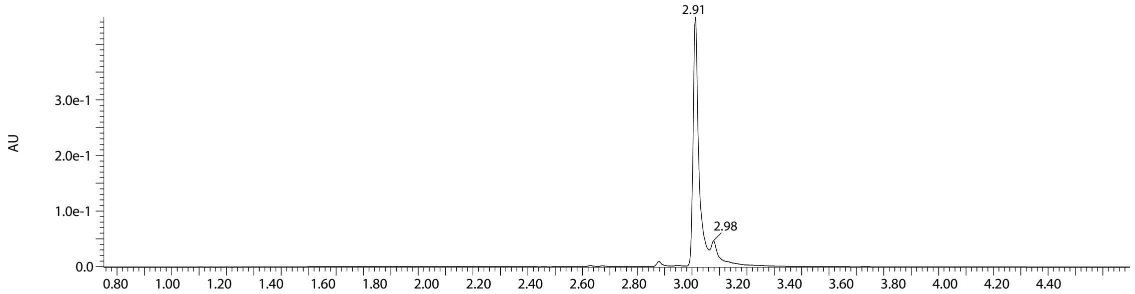

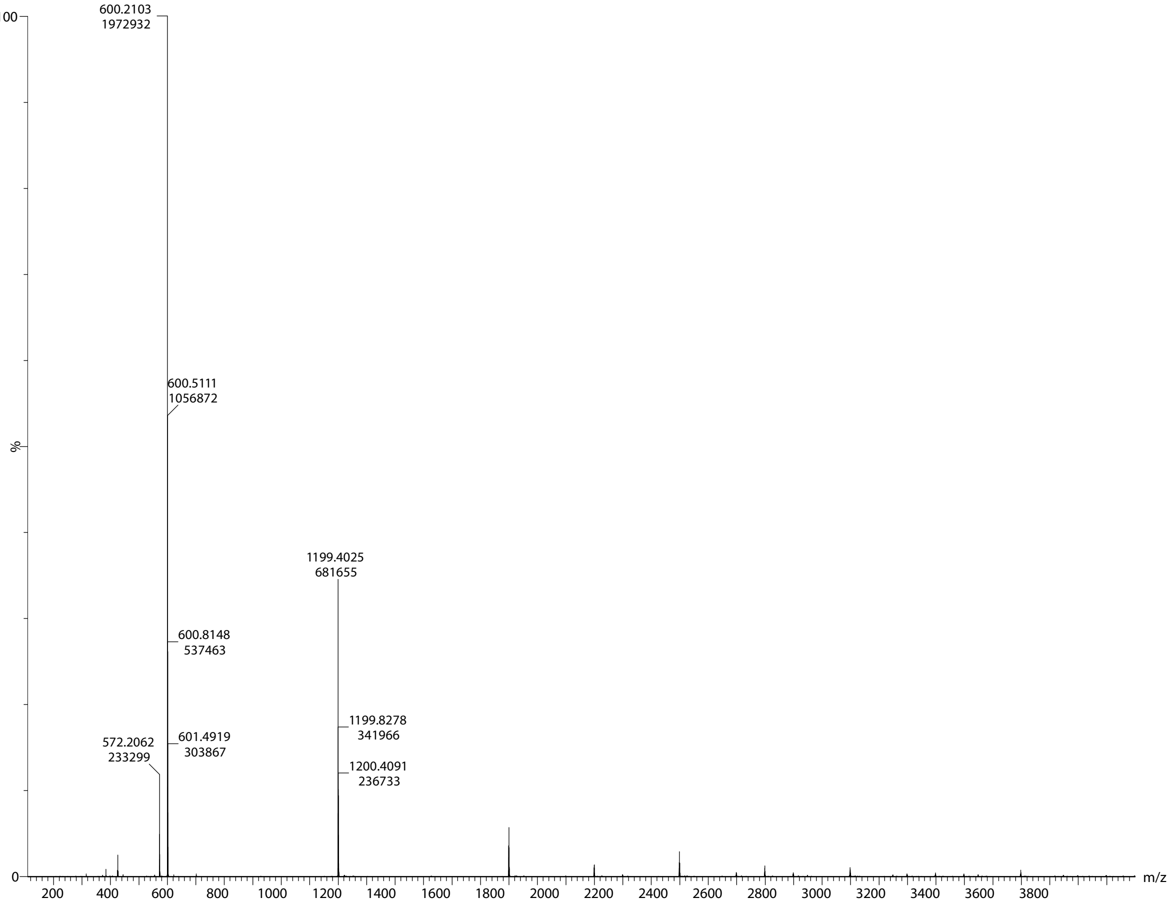

(*R*)-Glu-A146/B537 Compound **12 fluo**

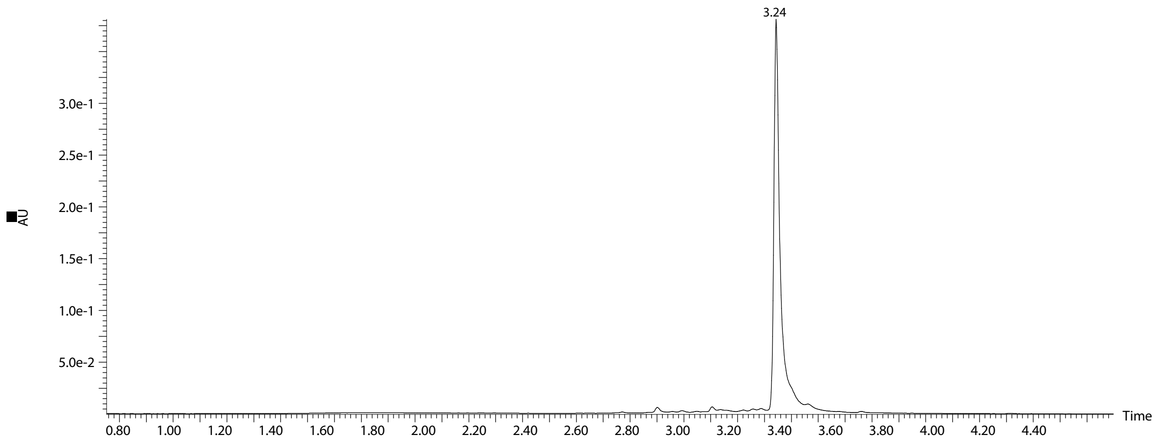

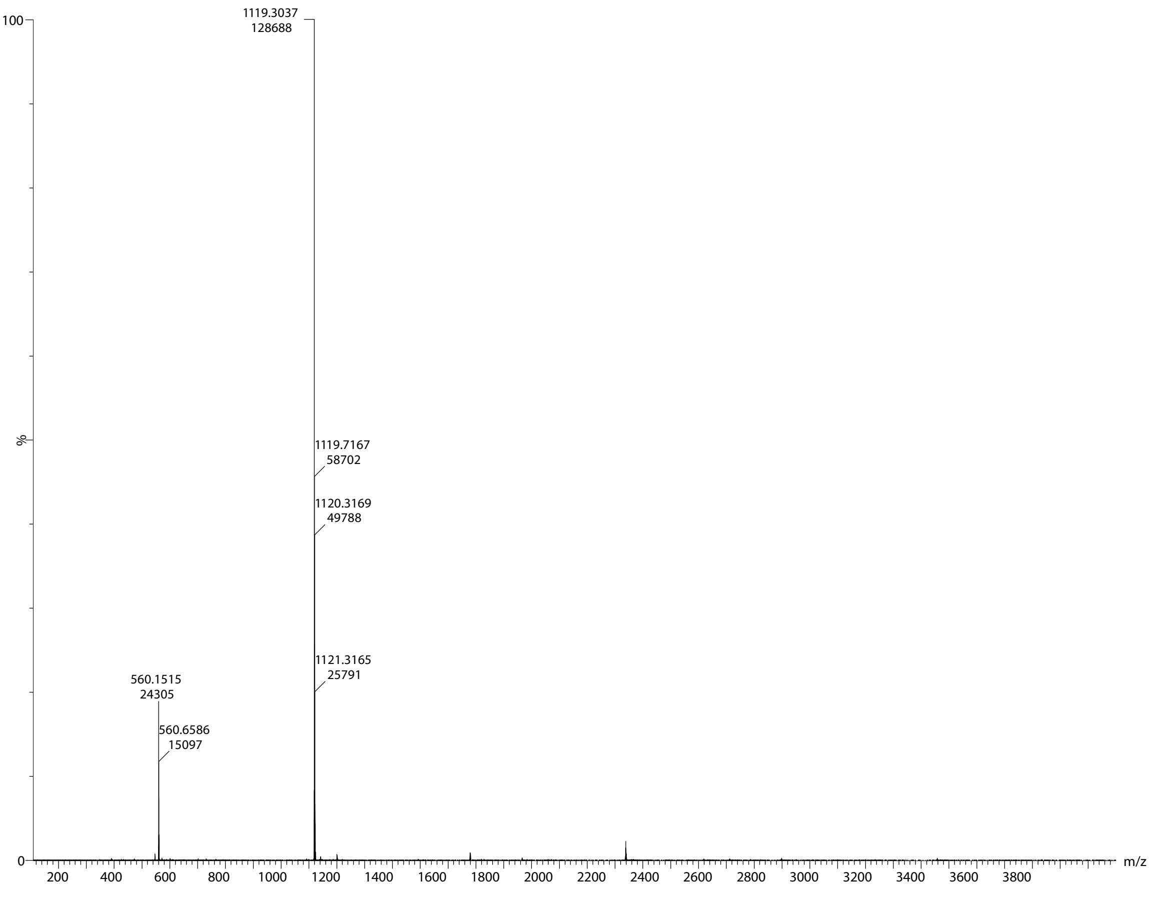

(*S*)-Glu-A146/B537 Compound **13**

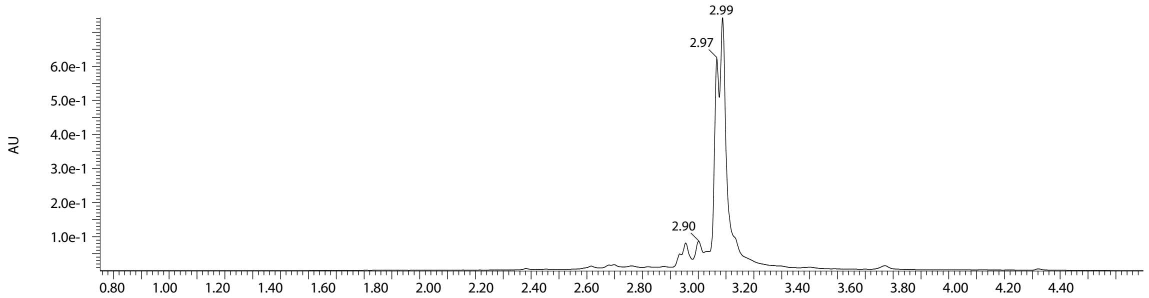

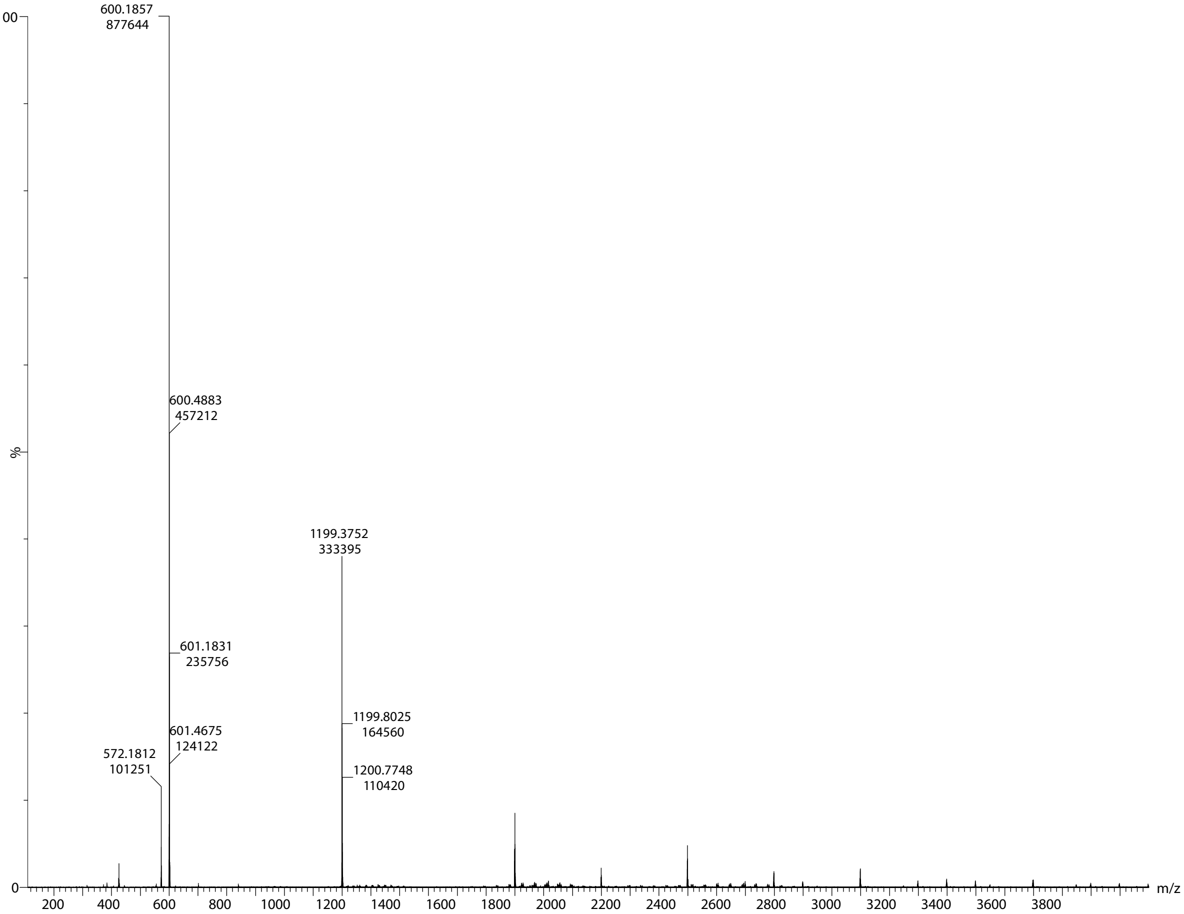

(*S*)-Glu-A146/B537 Compound **13 fluo**

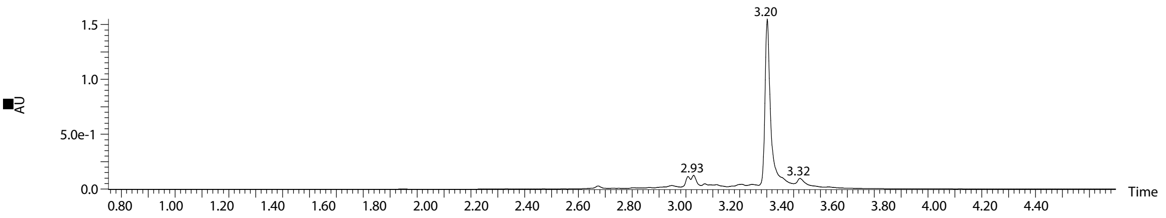

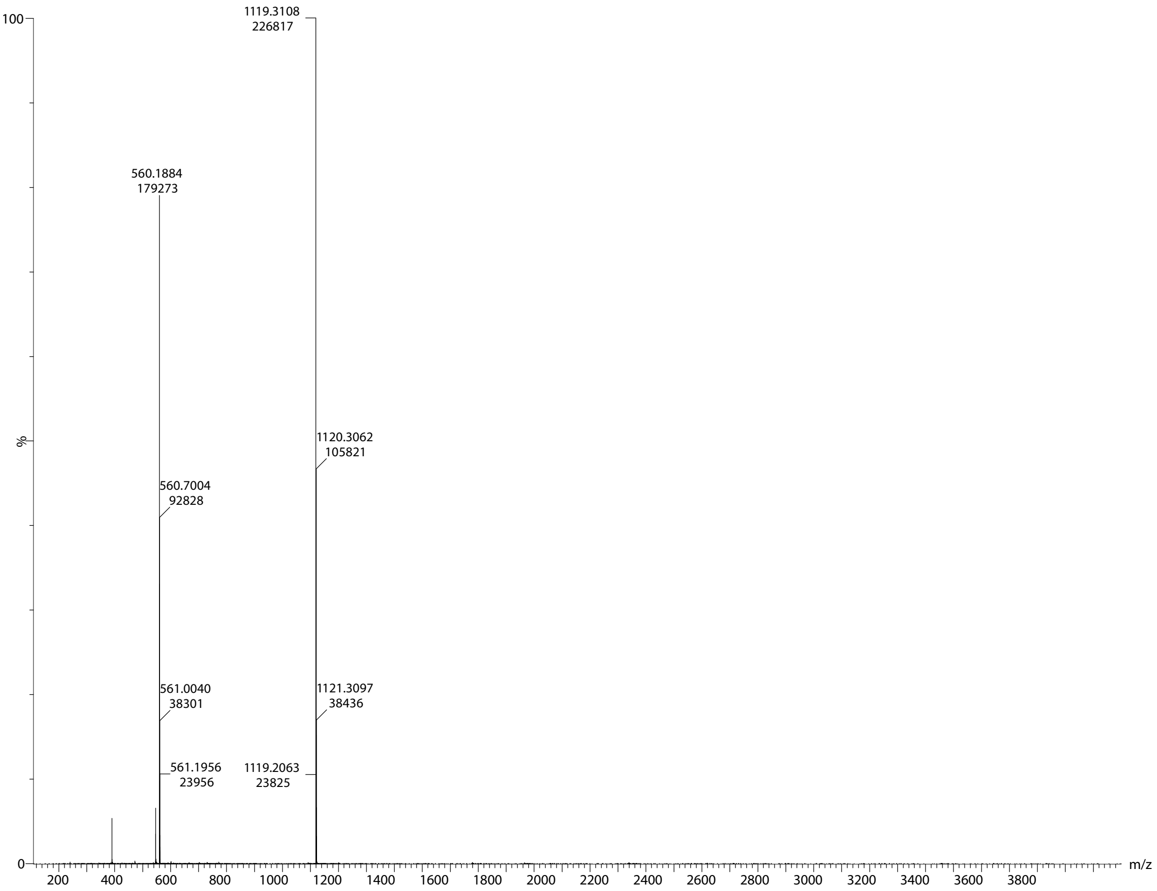

(*R*)-Glu-A245/B474 Compound **14**

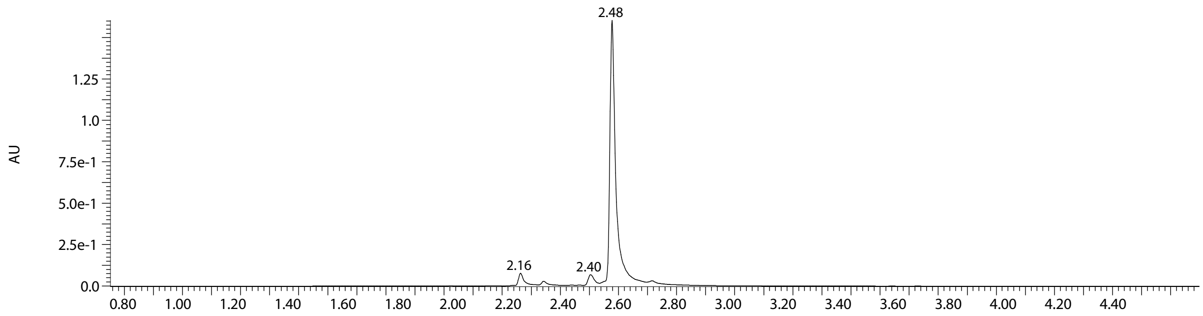

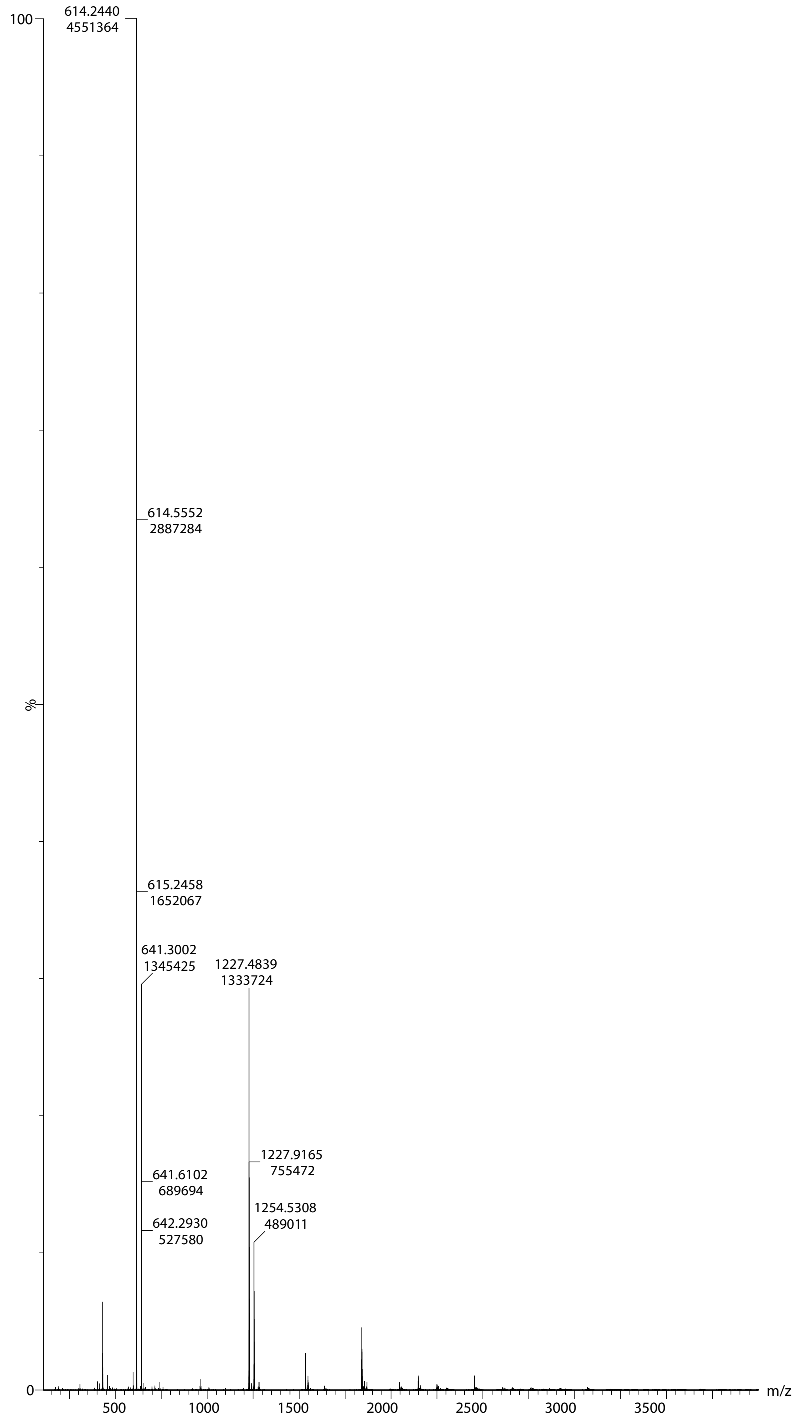

(*R*)-Glu-A245/B474 Compound **14 fluo**

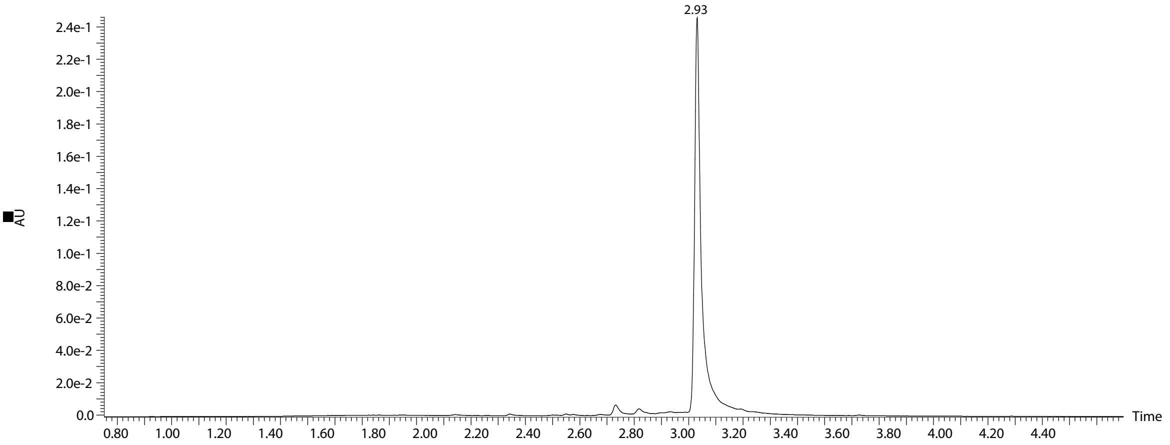

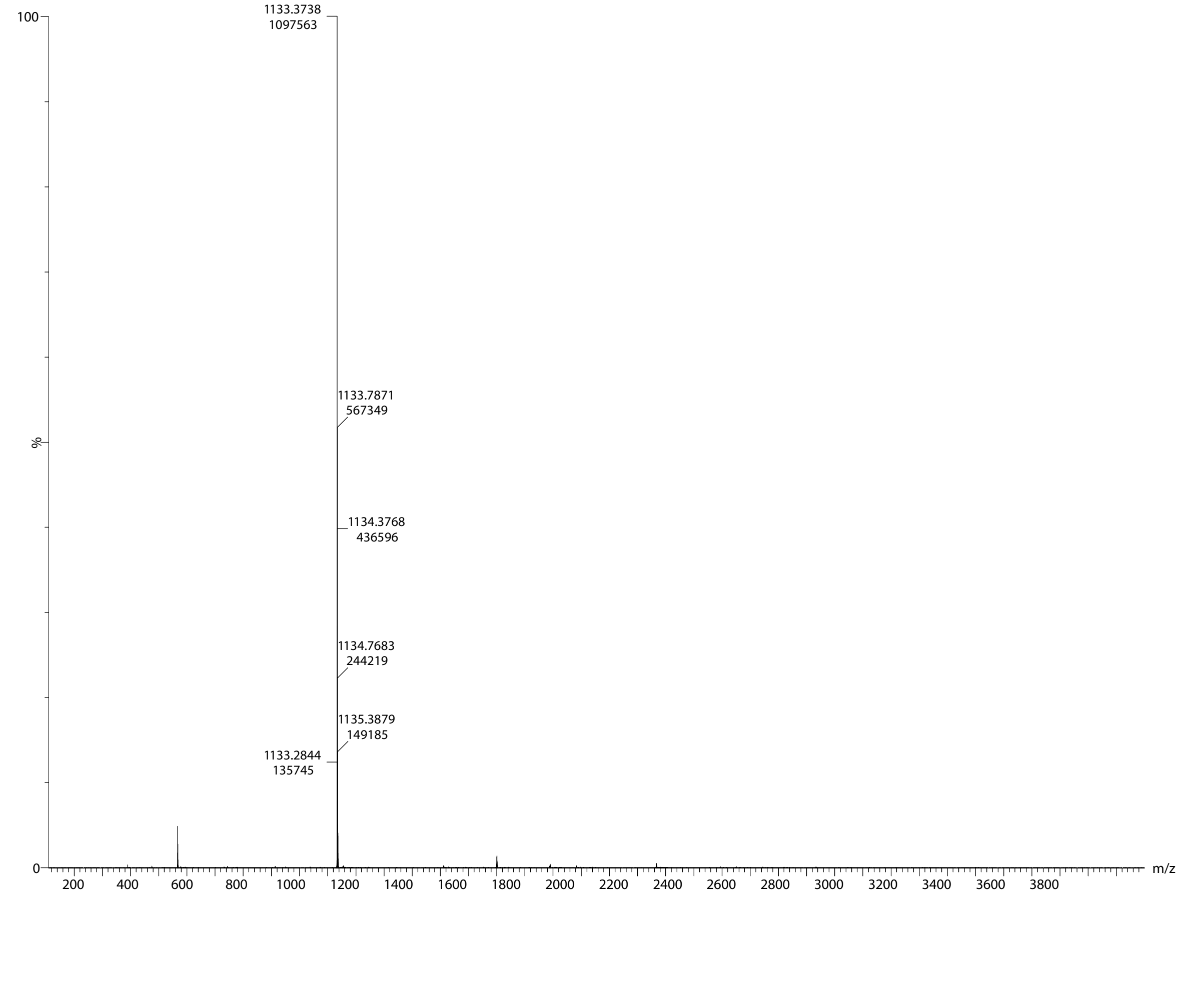

(***S***)**-Glu-A245/B474** Compound **15**

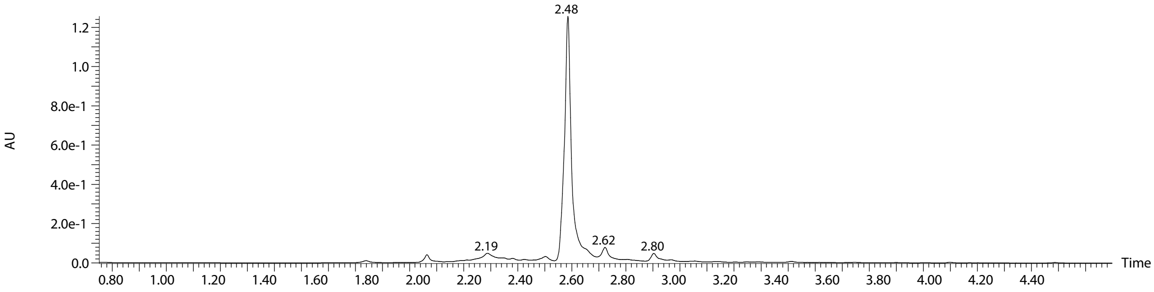

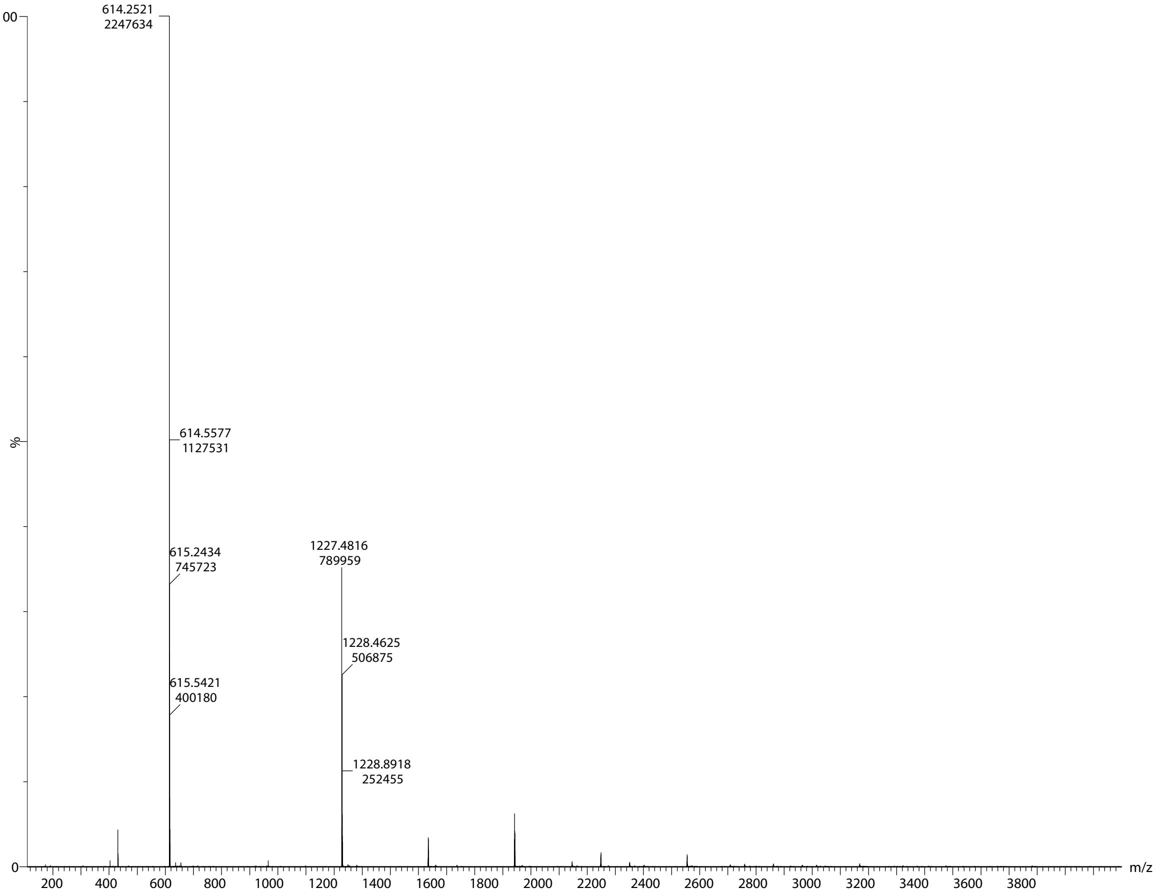

(*S*)-Glu-A245/B474 Compound **15 fluo**

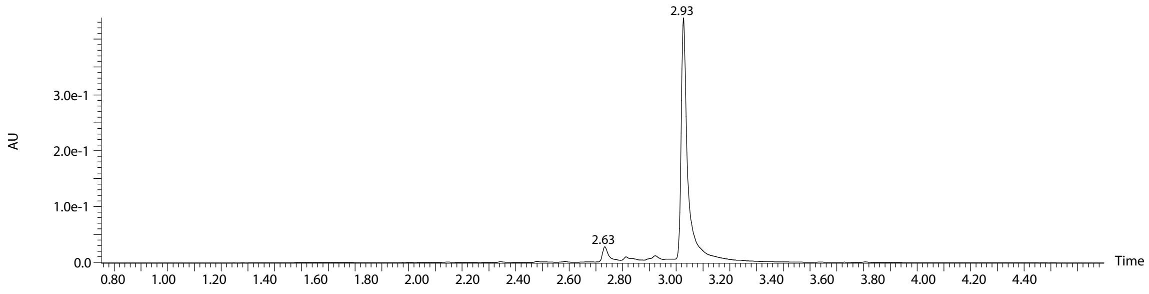

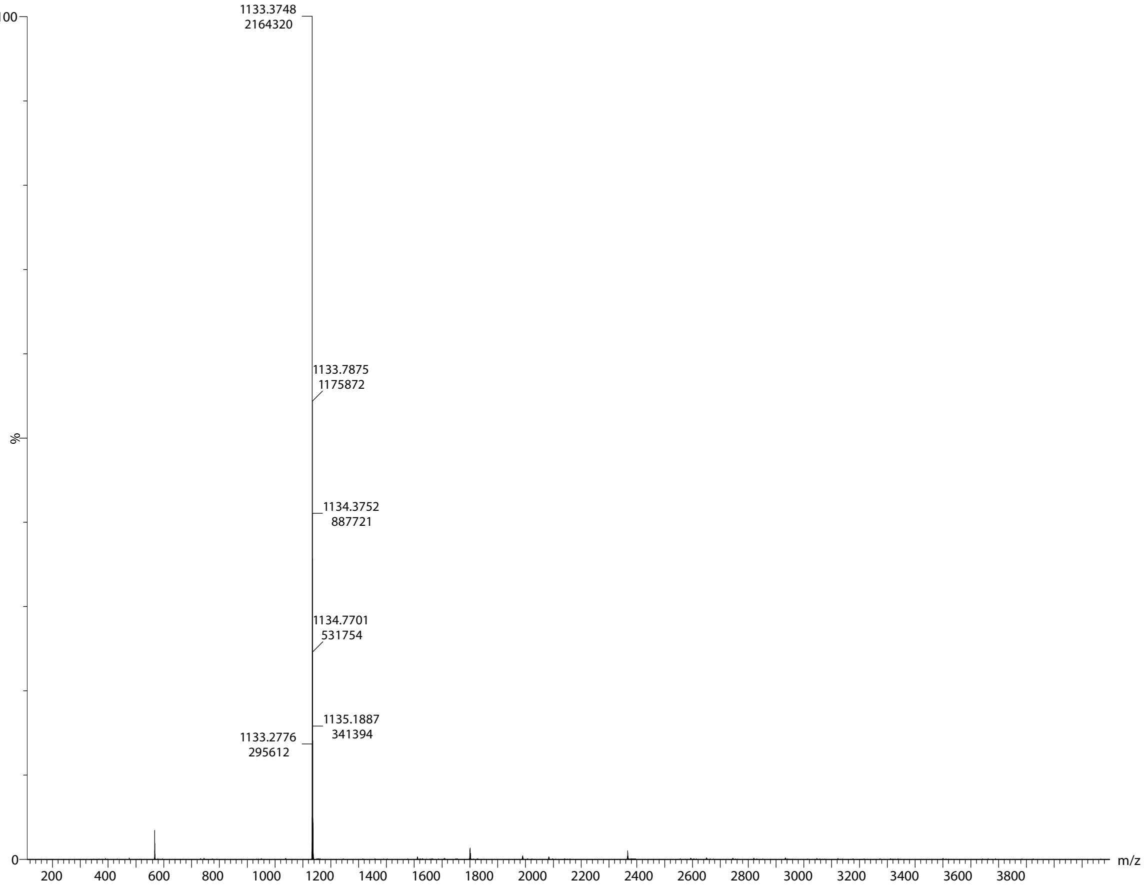

(*R*)-Glu-A200/B44 Compound **16**

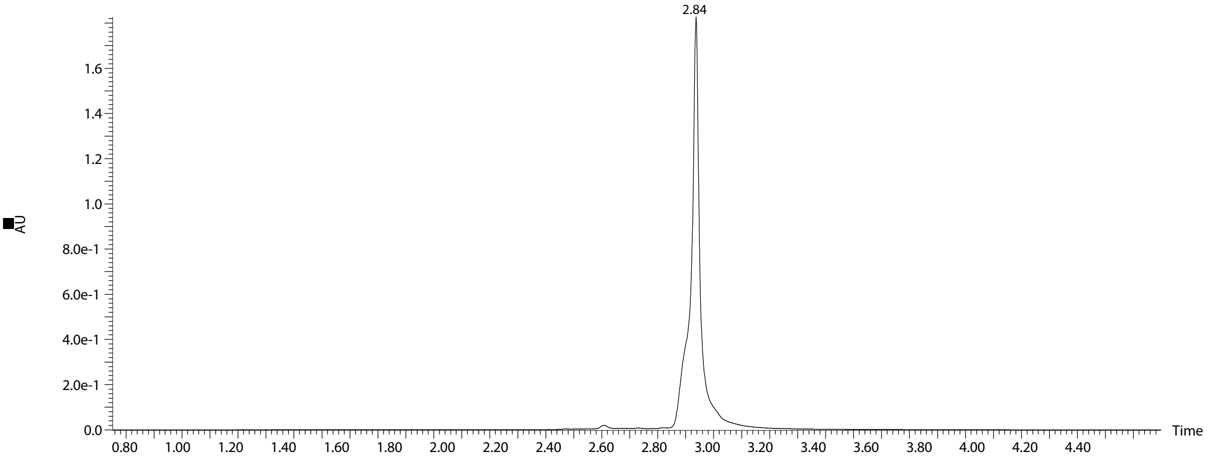

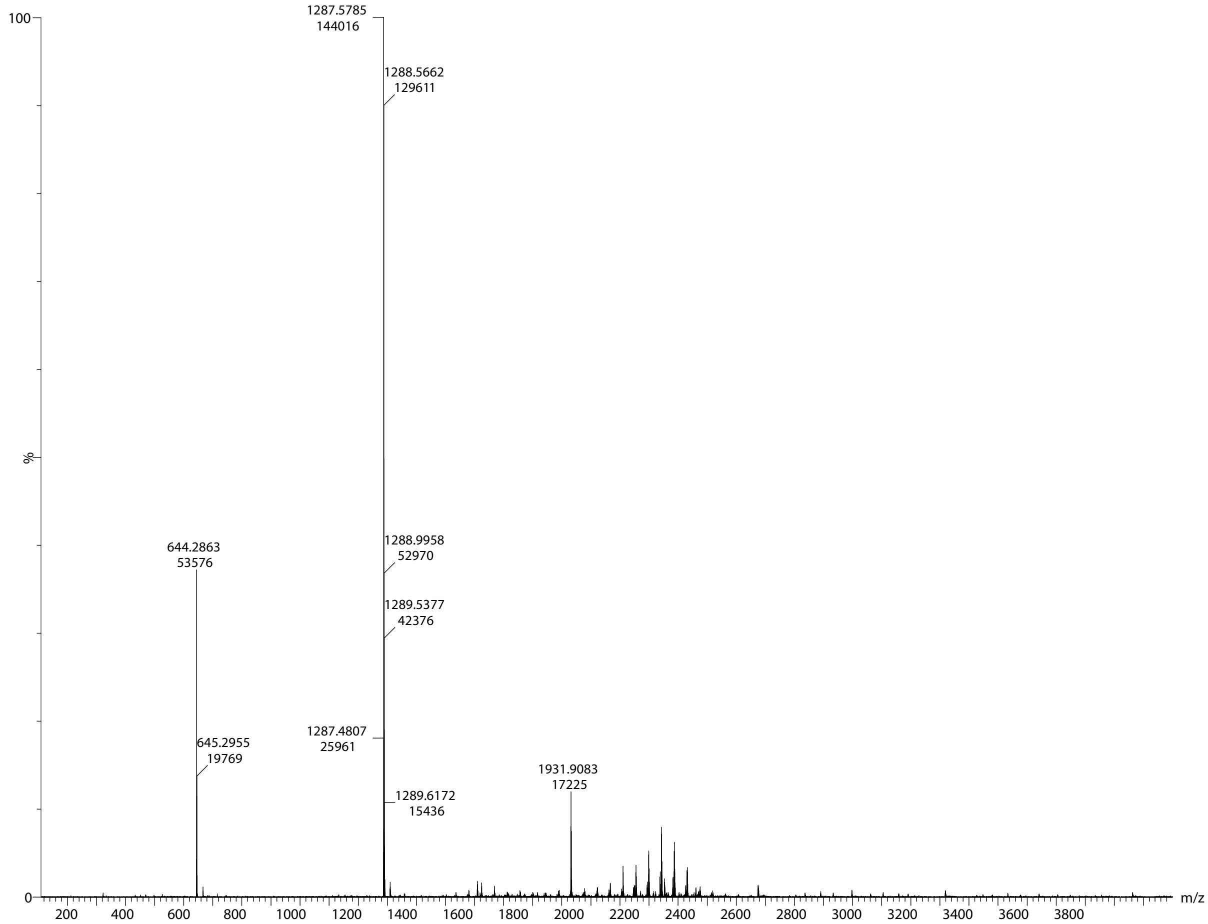

(*R*)-Glu-A200/B44 Compound **16 fluo**

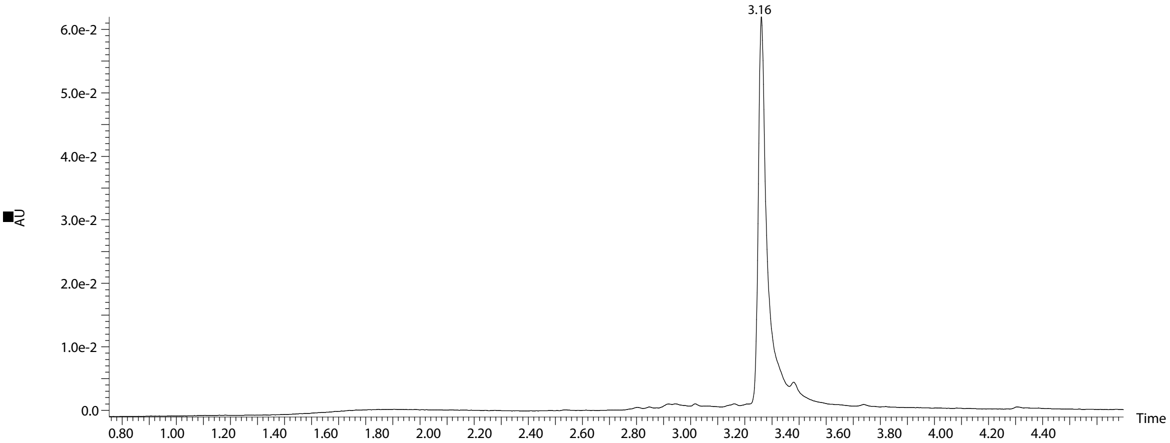

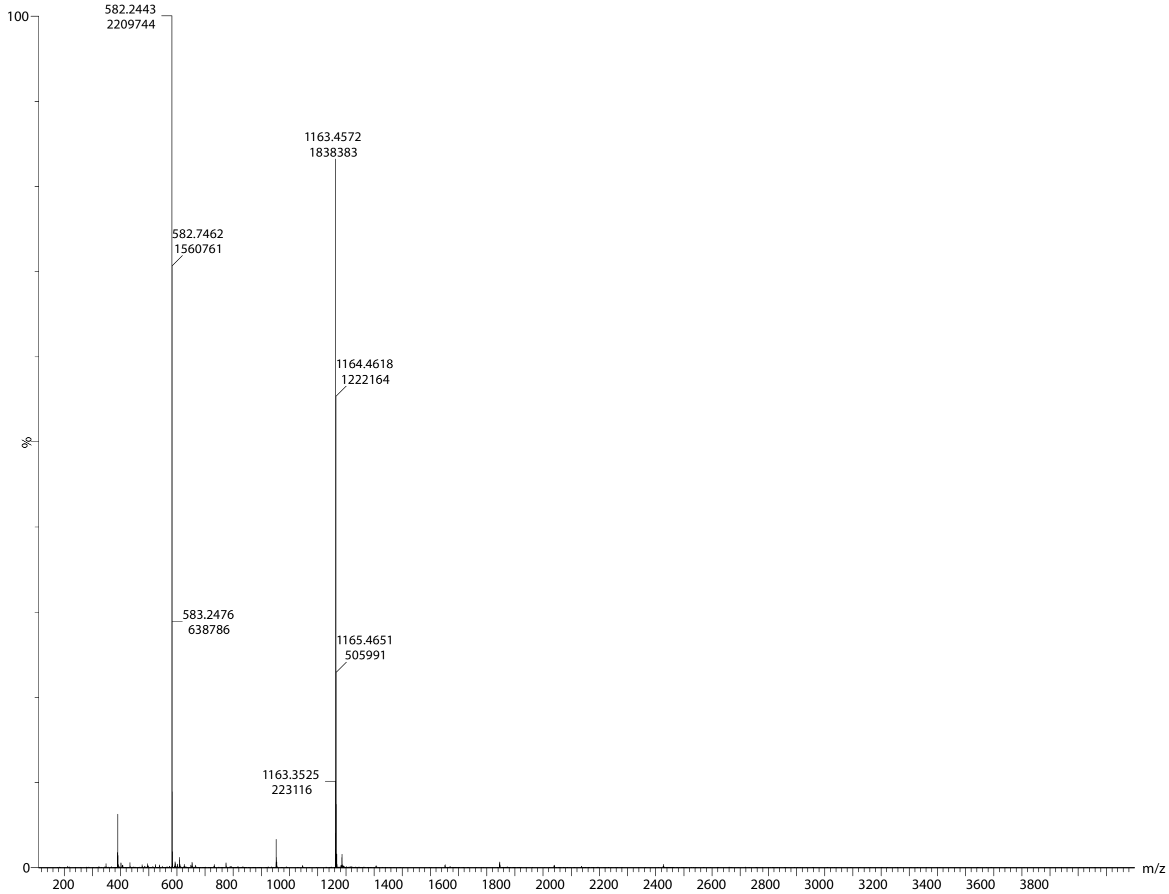

(*S*)-Glu-A200/B44 Compound **17**

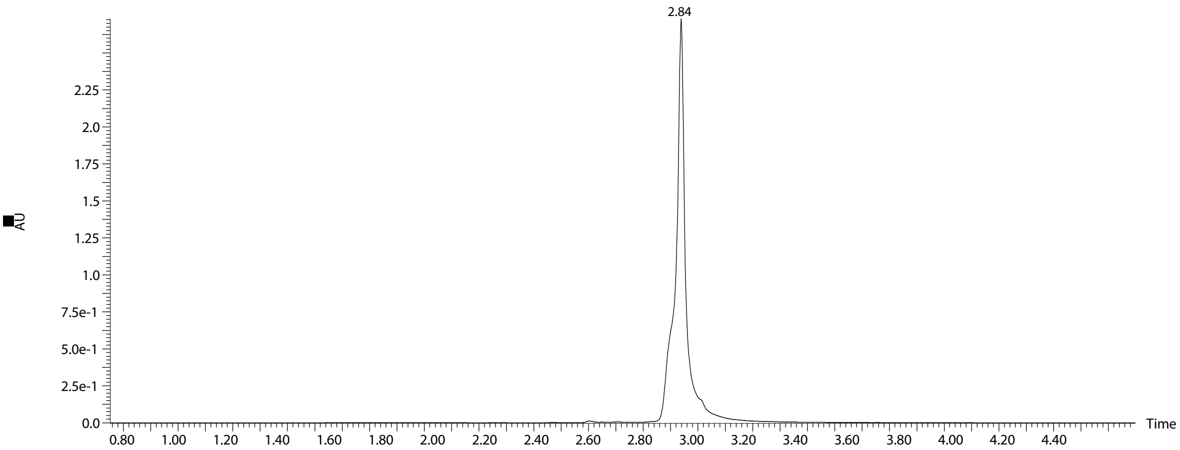

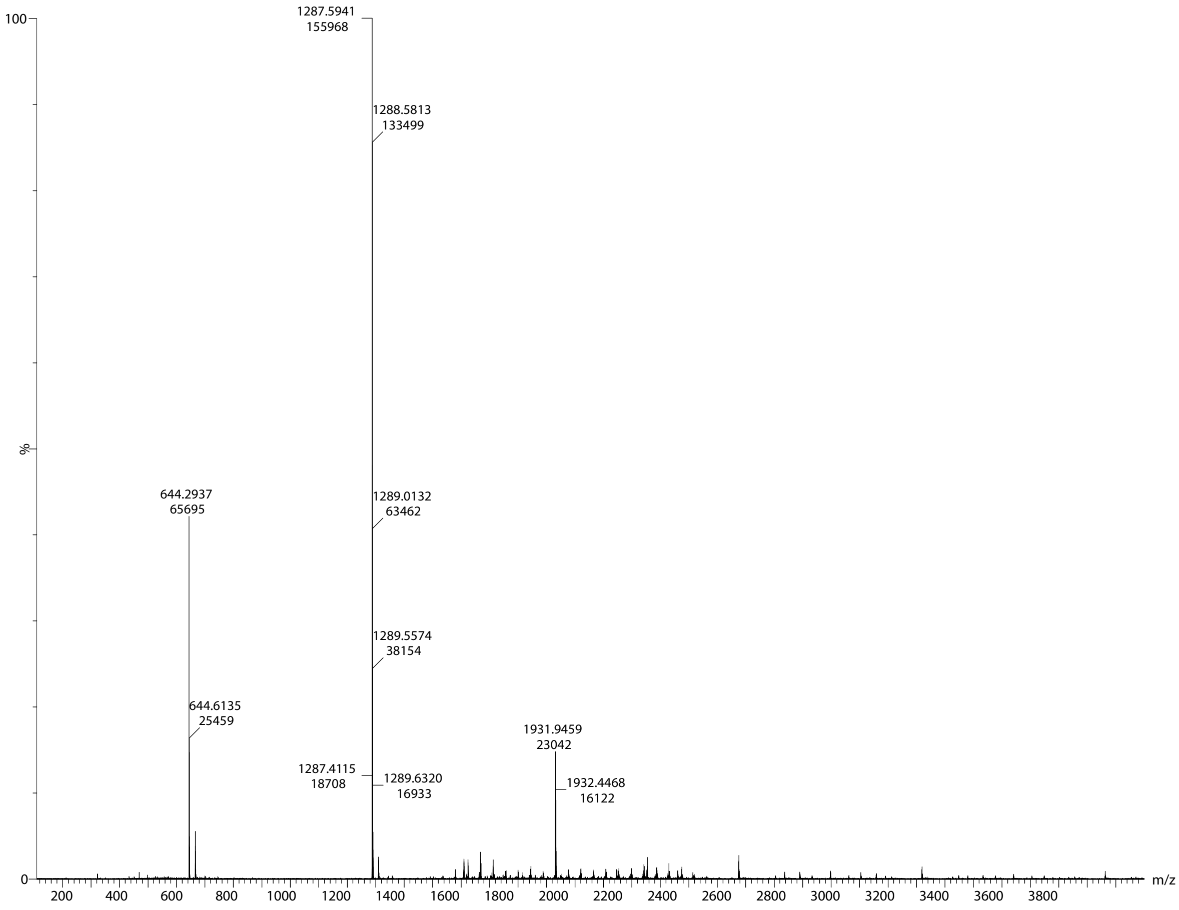

(*S*)-Glu-A200/B44 Compound **17 fluo**

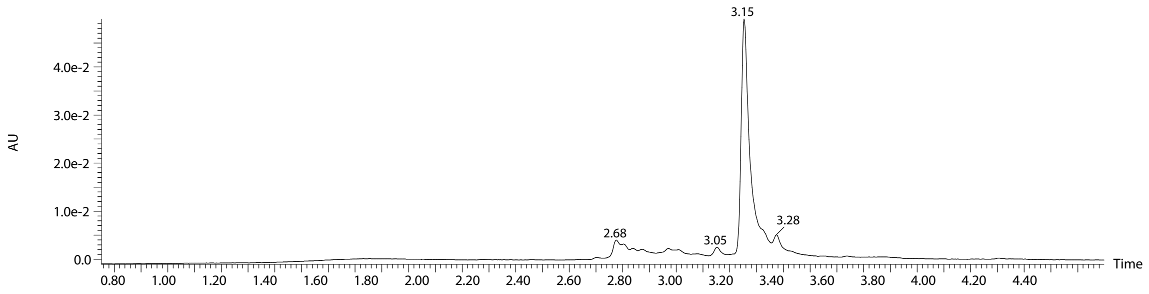

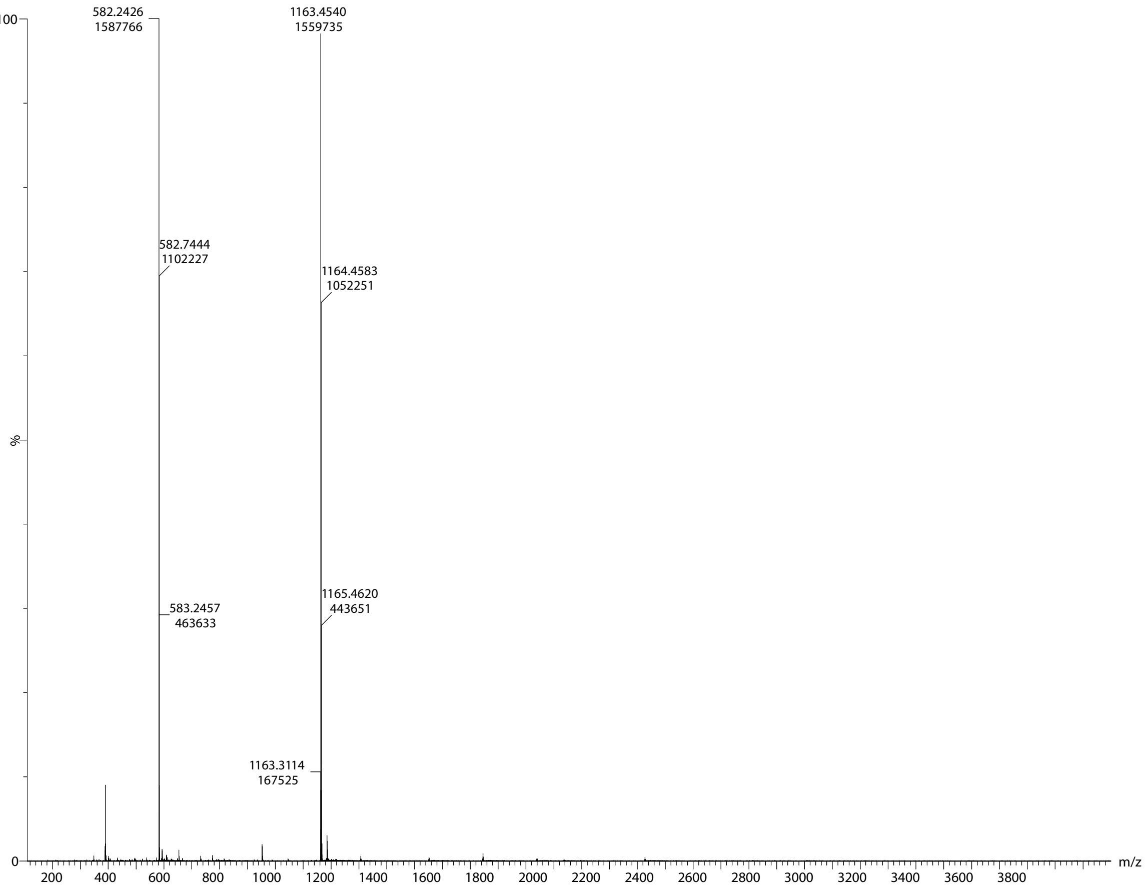

A219/B106B12DNA

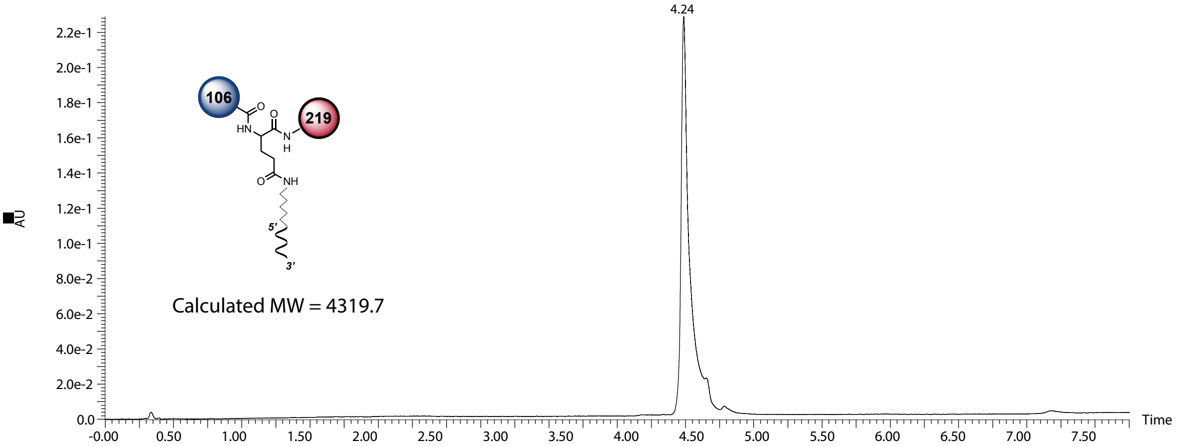

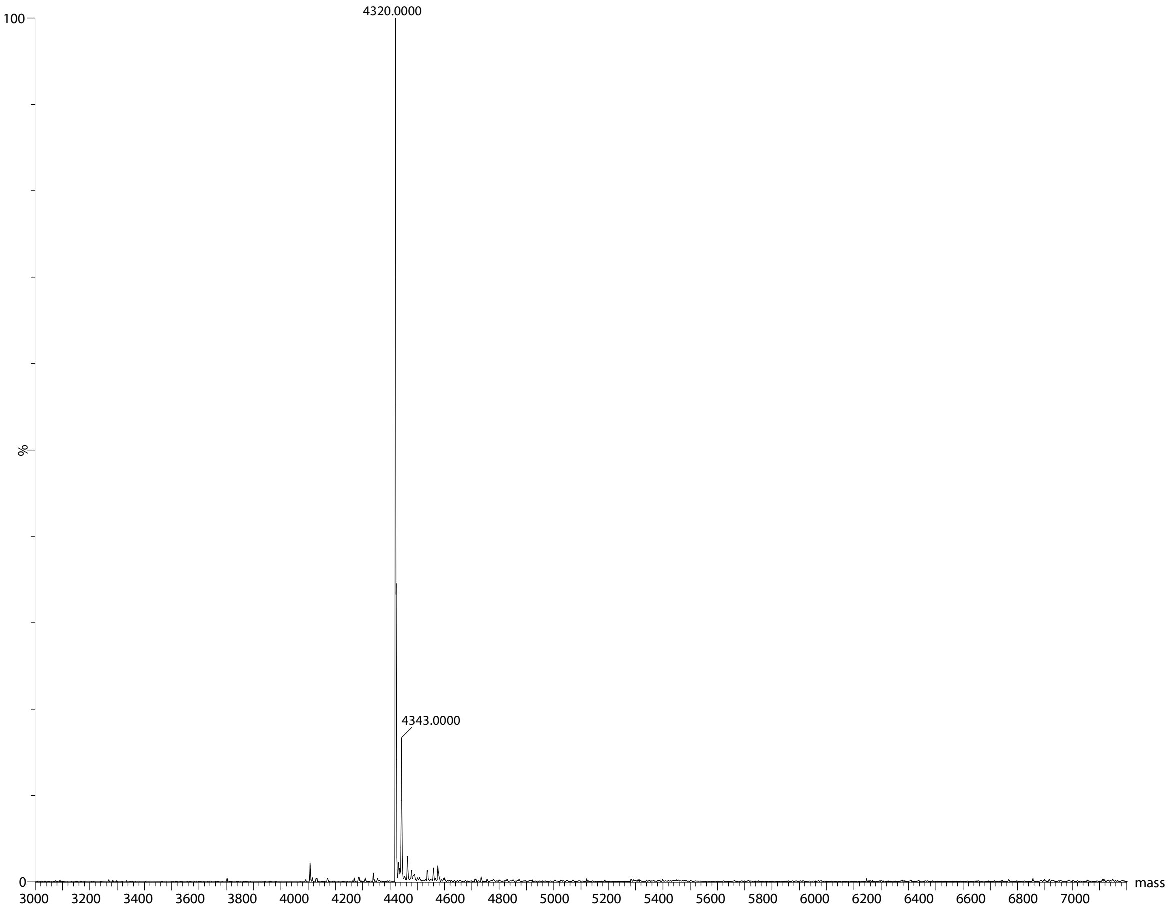

A12LNAHIT1

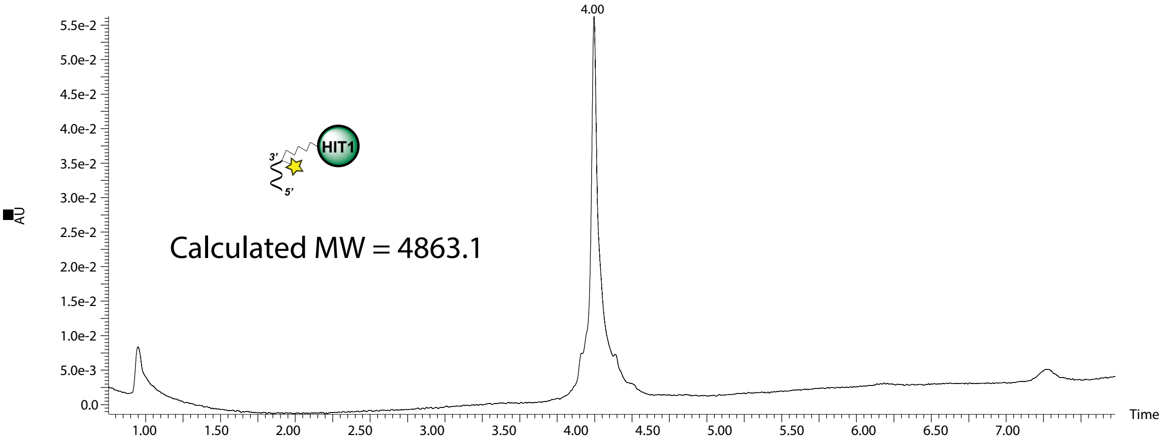

4.00
5.5e-2
5.0e-2
4.5e-2
4.0e-2
3.5e-2
3.0e-2
2.5e-2
2.0e-2
1.5e-2
1.0e-2
5.0e-3
0.0
AU
HIT1
3'
5'
Calculated MW = 4863.1
1.00
1.50
2.00
2.50
3.00
3.50
4.00
4.50
5.00
5.50
6.00
6.50
7.00
Time

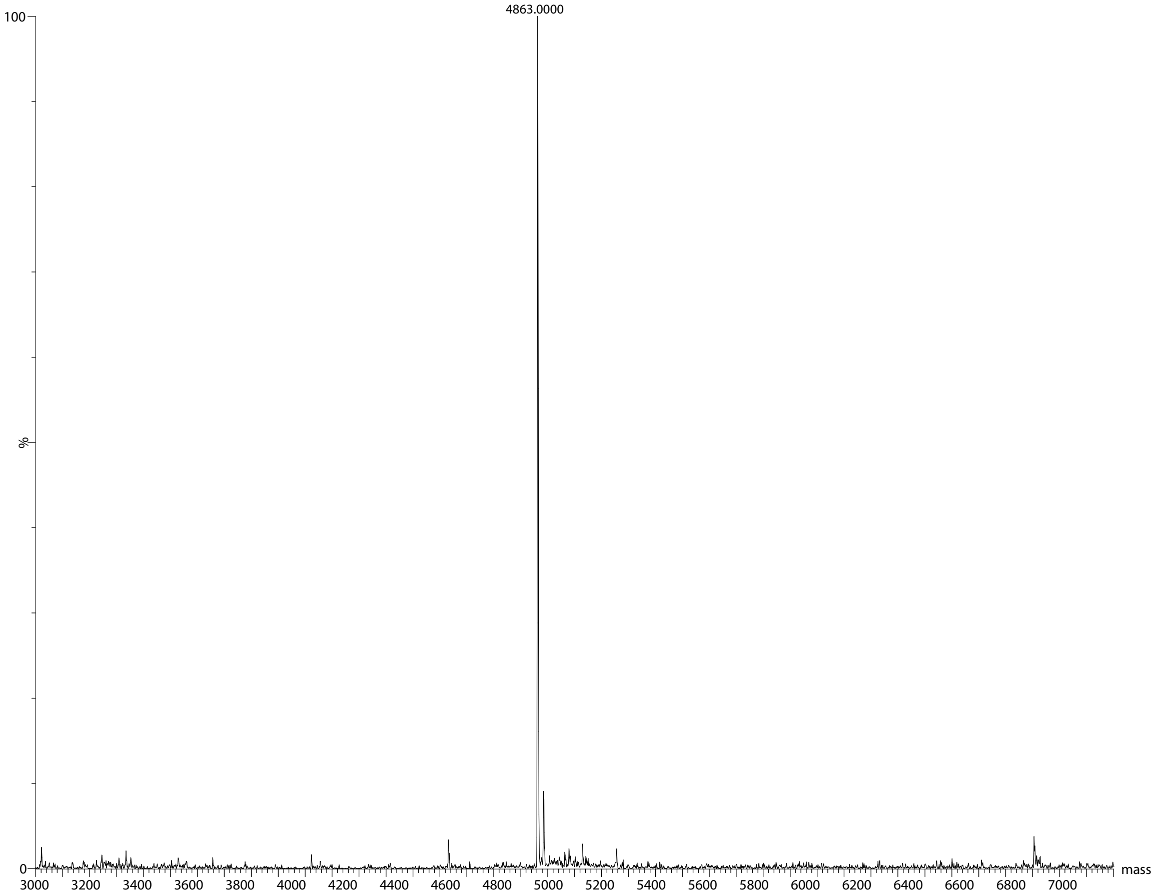

4863.0000
100
%
0
3000
3200
3400
3600
3800
4000
4200
4400
4600
4800
5000
5200
5400
5600
5800
6000
6200
6400
6600
6800
7000
mass

A24/B137B12DNA

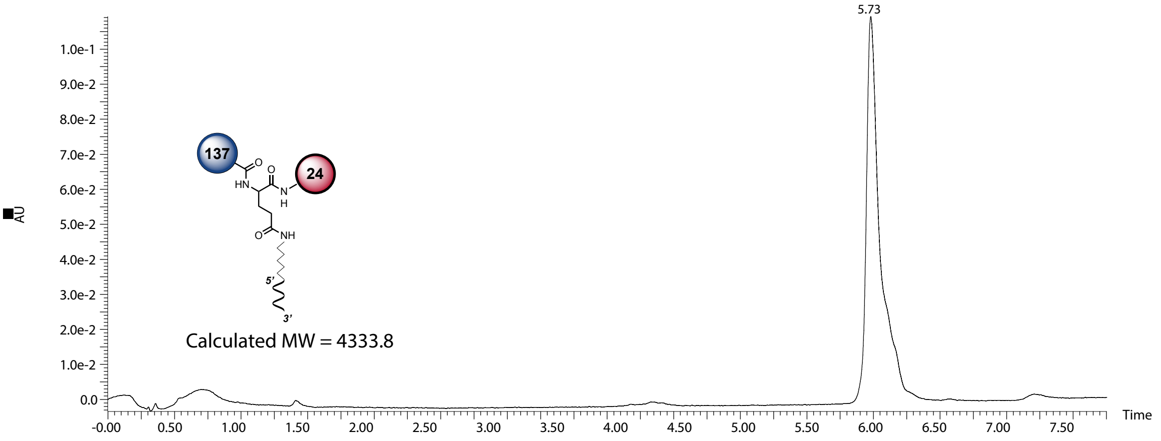

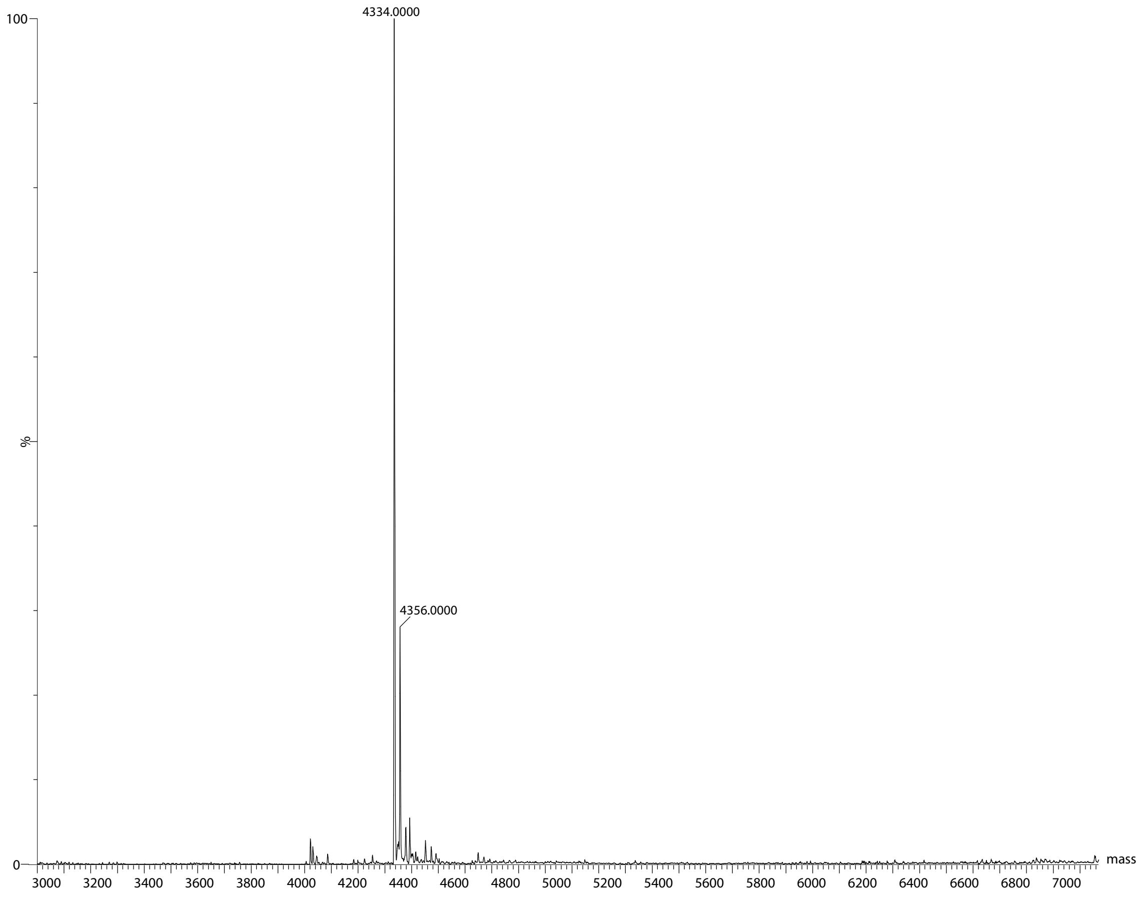

A12LNAHIT2

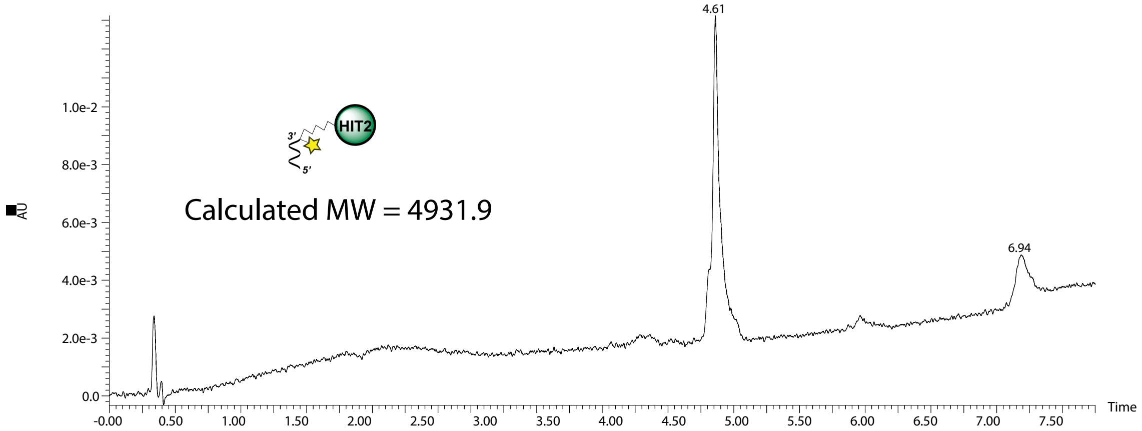
4.61
6.94
HIT2
3'
5'
Calculated MW = 4931.9
AU
1.0e-2
8.0e-3
6.0e-3
4.0e-3
2.0e-3
0.0
-0.00 0.50 1.00 1.50 2.00 2.50 3.00 3.50 4.00 4.50 5.00 5.50 6.00 6.50 7.00 7.50
Time

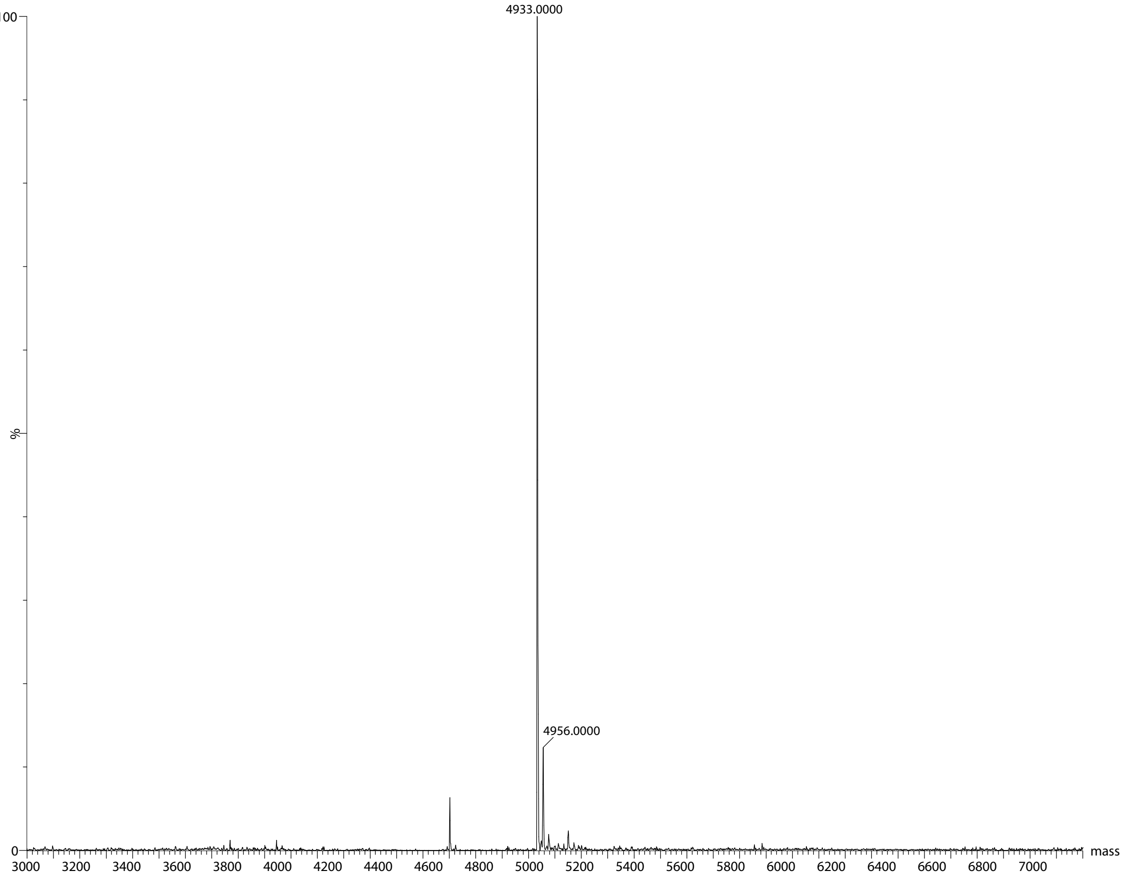
4933.0000
4956.0000
100
%
0
3000 3200 3400 3600 3800 4000 4200 4400 4600 4800 5000 5200 5400 5600 5800 6000 6200 6400 6600 6800 7000
mass

Compound **18** Allyl

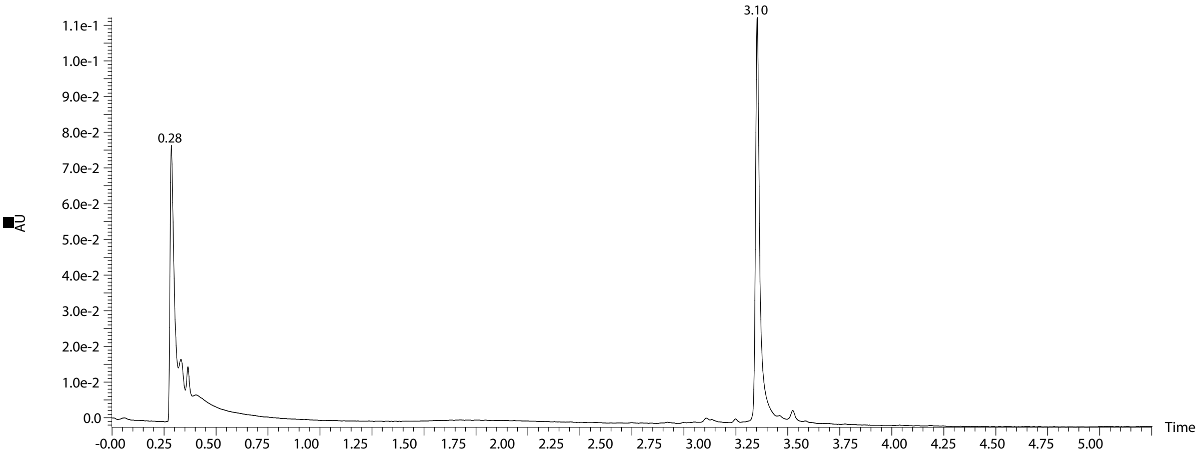

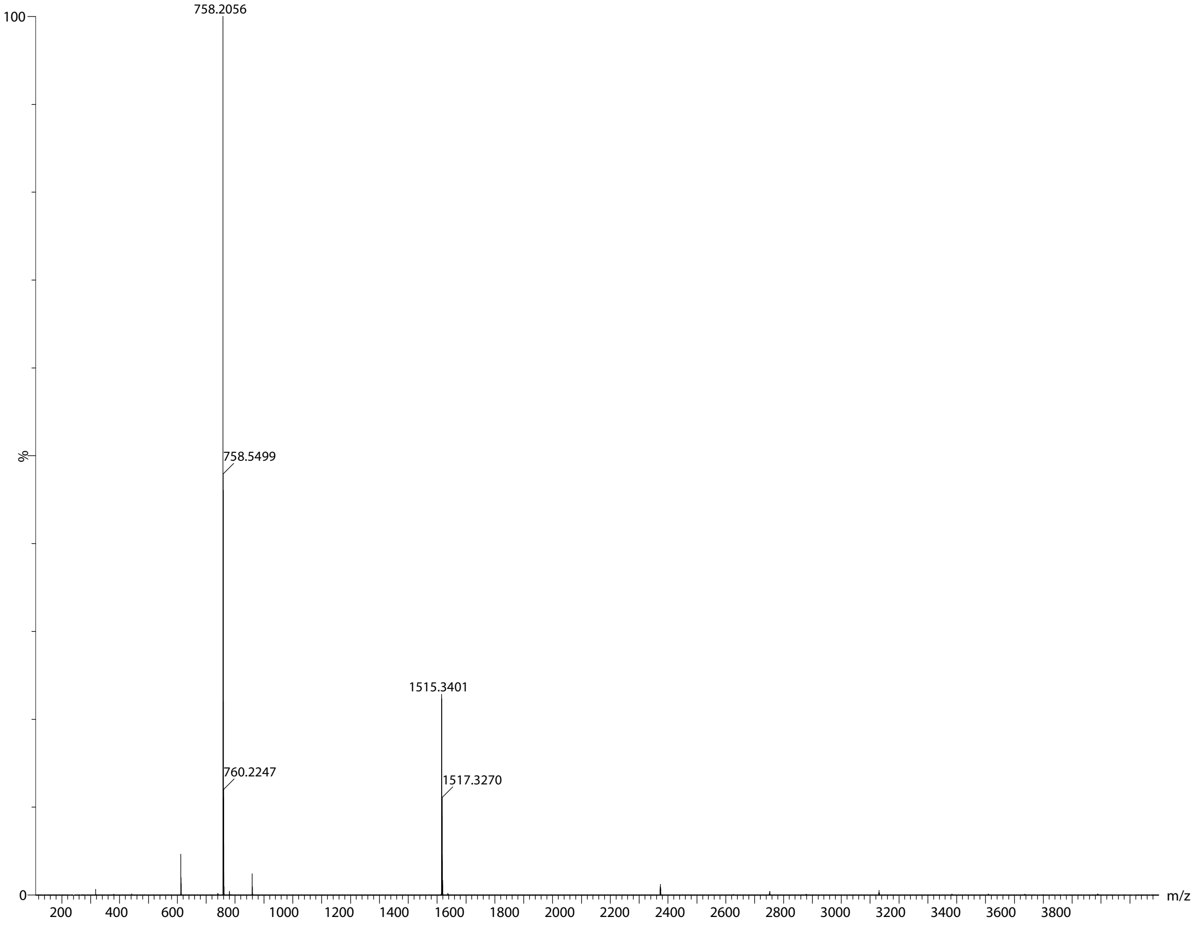

Compound **18** fluo

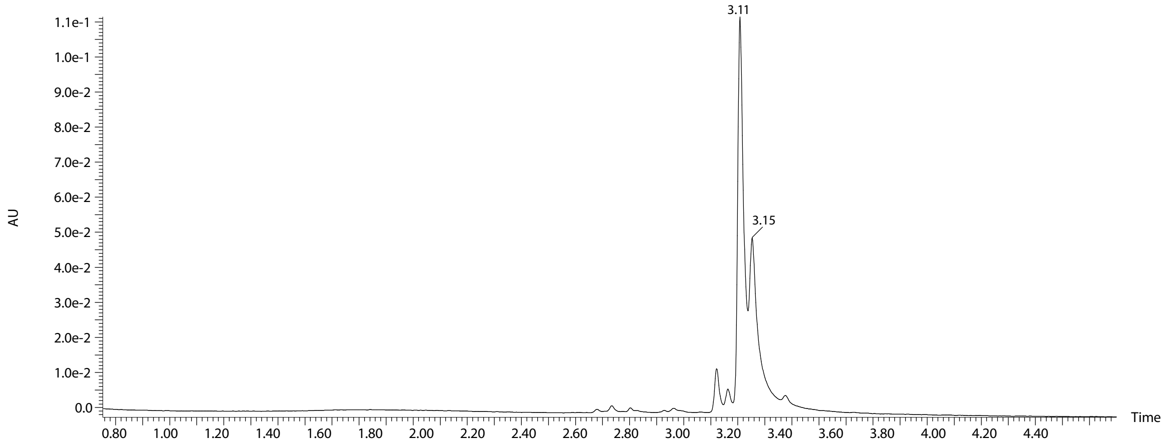

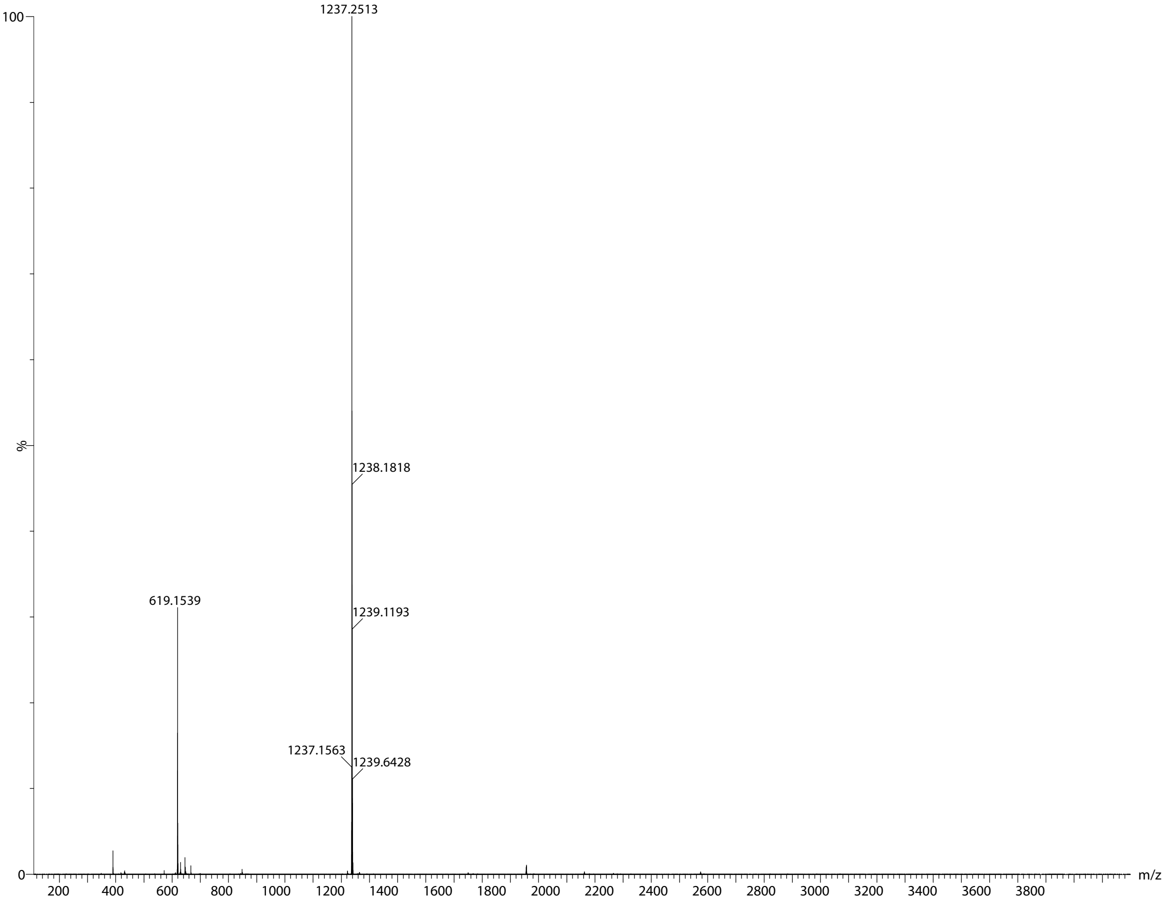

Compound **19** allyl

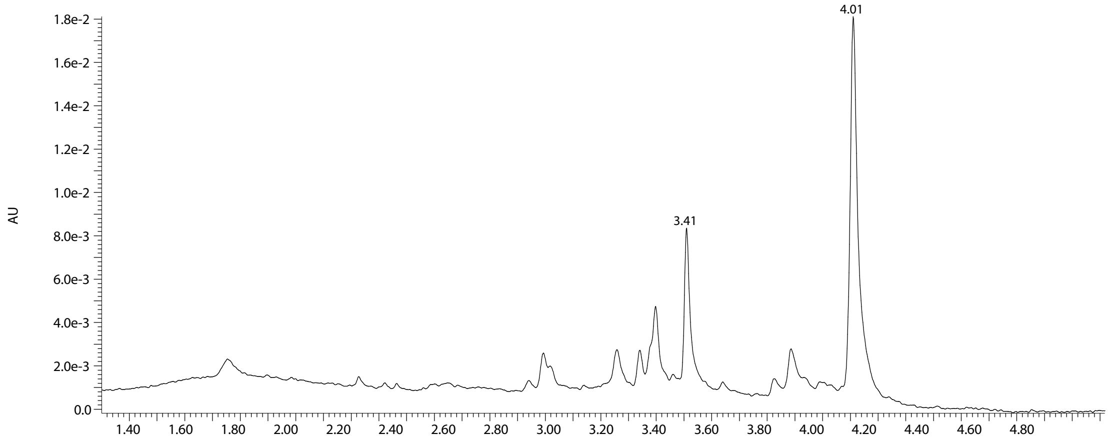

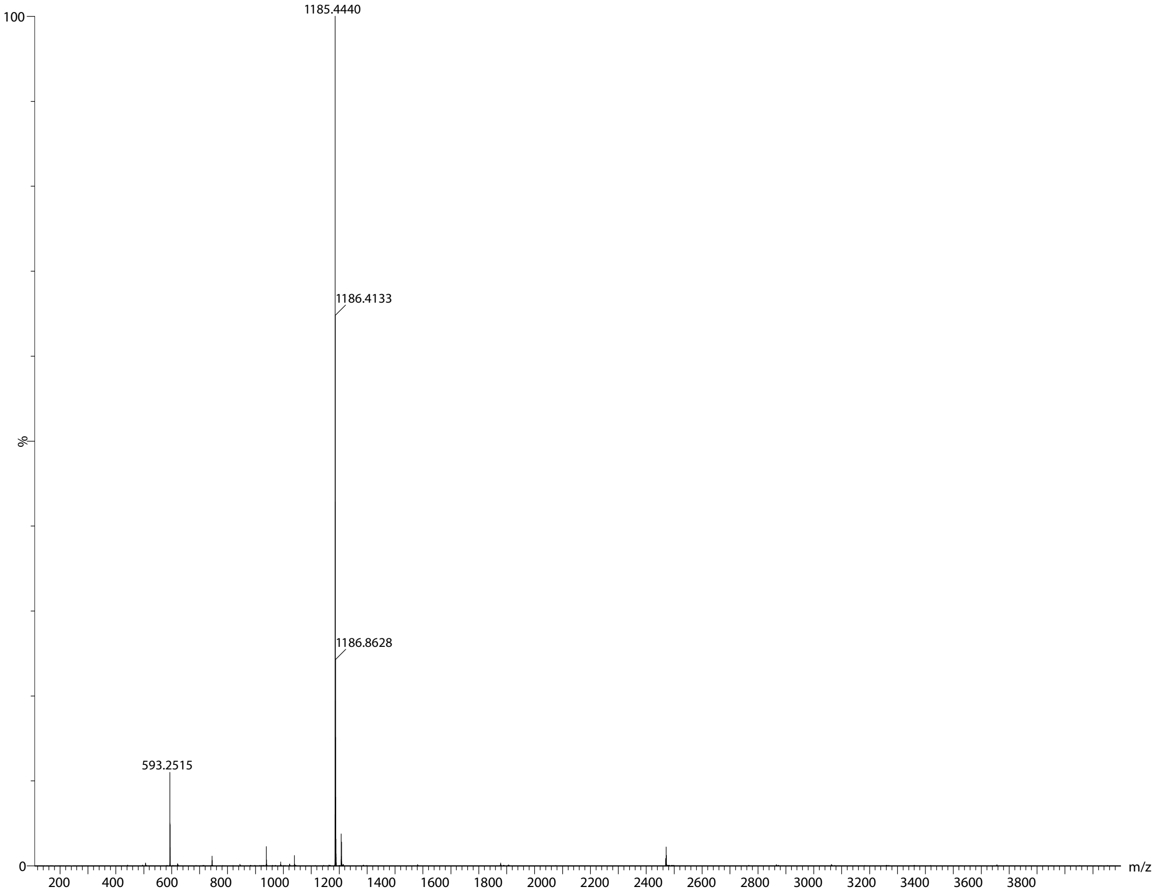

Compound **19** fluo

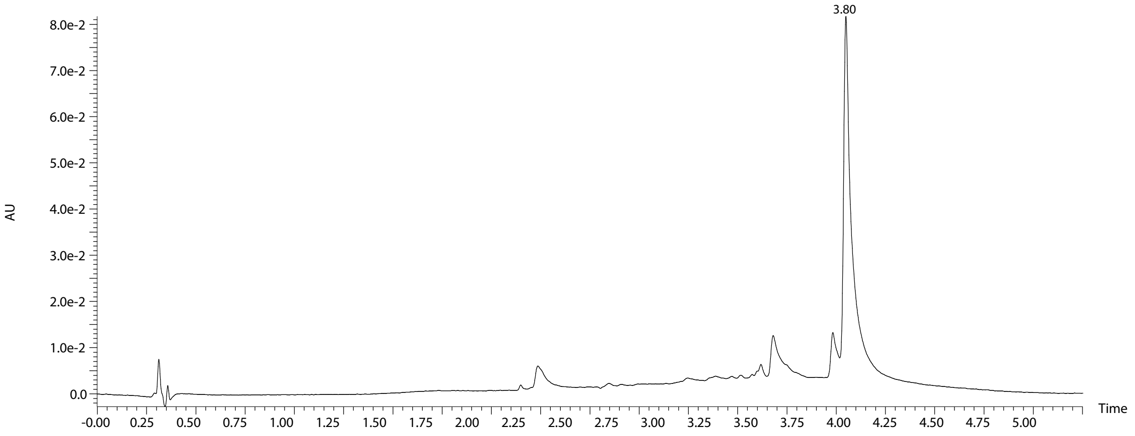

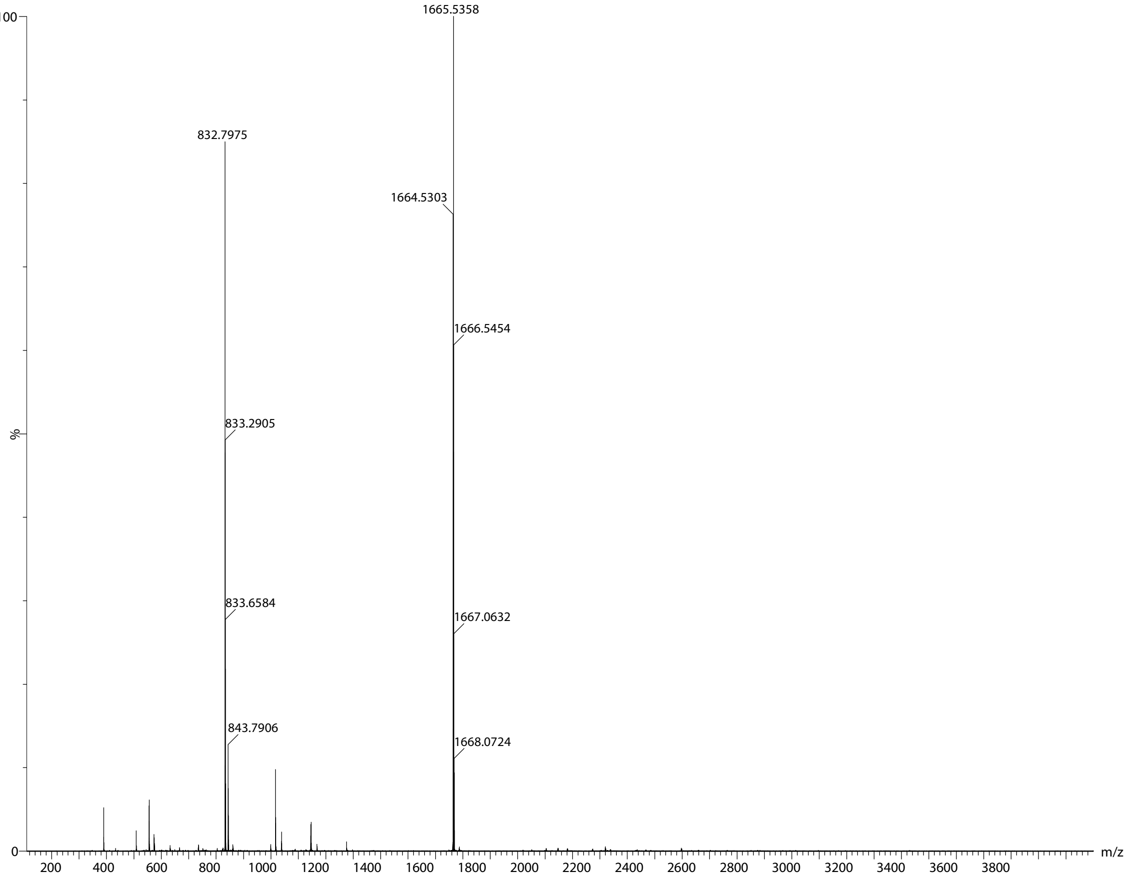

Supplement: Supplementary file 1 — Supporting Information [file ADVS-7-2001970-s001.pdf]
